# Supplementary material for: Human Adult Astrocyte Extracellular Vesicle Transcriptomics Study Identifies Specific RNAs Which Are Preferentially Secreted as EV Luminal Cargo
Source: Genes (Basel). 2023 Mar 31;14(4):853. doi: 10.3390/genes14040853 (PMC10138286; doi:10.3390/genes14040853)
Supplement: Supplementary file 1 [file genes-14-00853-s001.zip › genes-2247275-supplementary.pdf]

genes MDPI

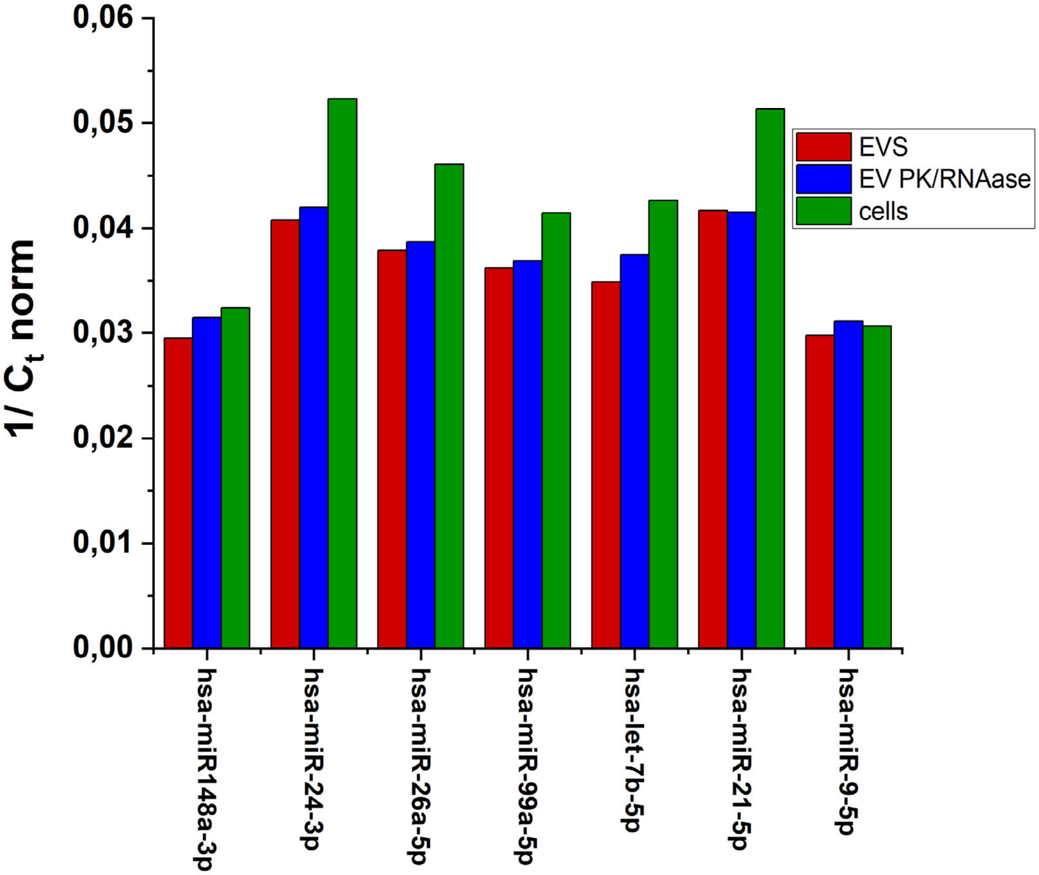

Figure S1. The total RNA from EV, EV ProteinaseK/Rnase A/T1 and astrocyte cells of another human adult astrocyte (Cell applications Inc.-882A-05) were isolated. The RT-qPCR was done using Brilliant III SYBR green. The Ct values are normalized using amount of cDNA used for qPCR and inverted to represent the relative quantification. All miRNAs tested were found to be higher in cells than EVs in the miRNA-seq data. With the exception of miR-9-5p which is highest in ProteinaseK/RNase A/T1 treated EVs, all the miRNA tested were preferentially cellular.

Method:
Cell culture and EV production: Cells were grown in manufacturer provided medium then adapted to Innoprot complete medium. EV isolation, Proteinase K /RNase treatment and RNA isolation were as described in main manuscript.
QPCR: 5µl of total RNA from EV-enriched and ProteinaseK /RNAseA/T1 treated fractions and 100 mg total RNA from cells were used for cDNA synthesis with Qiagen miRCURY cDNA synthesis kit, (no spike-in added).
Before QPCR cDNA yield were measured with Qubits 1xDNA kit (ThermoFisher). CDNA was diluted 1:40 and SYBR greenIII master mix (Agilent) was used with CFX96 thermal cycler (BioRAD), cycling conditions according to recommendation for LNA-primer (40 cycles). We corrected Ct values with amount of cDNA.

GeneGlobe ID: miRCURY LNA PCR primers
YP00204230 has-miR-21-5p
YP00204513 hsa-miR-9-5p
YP00204750 hsa-miRlet-7b-5p
YP00204260 has-24-3p
YP00206023 hsa-26a-5p
YP00204521 has-99a-5p
YP00205867 hsa-148a-3p

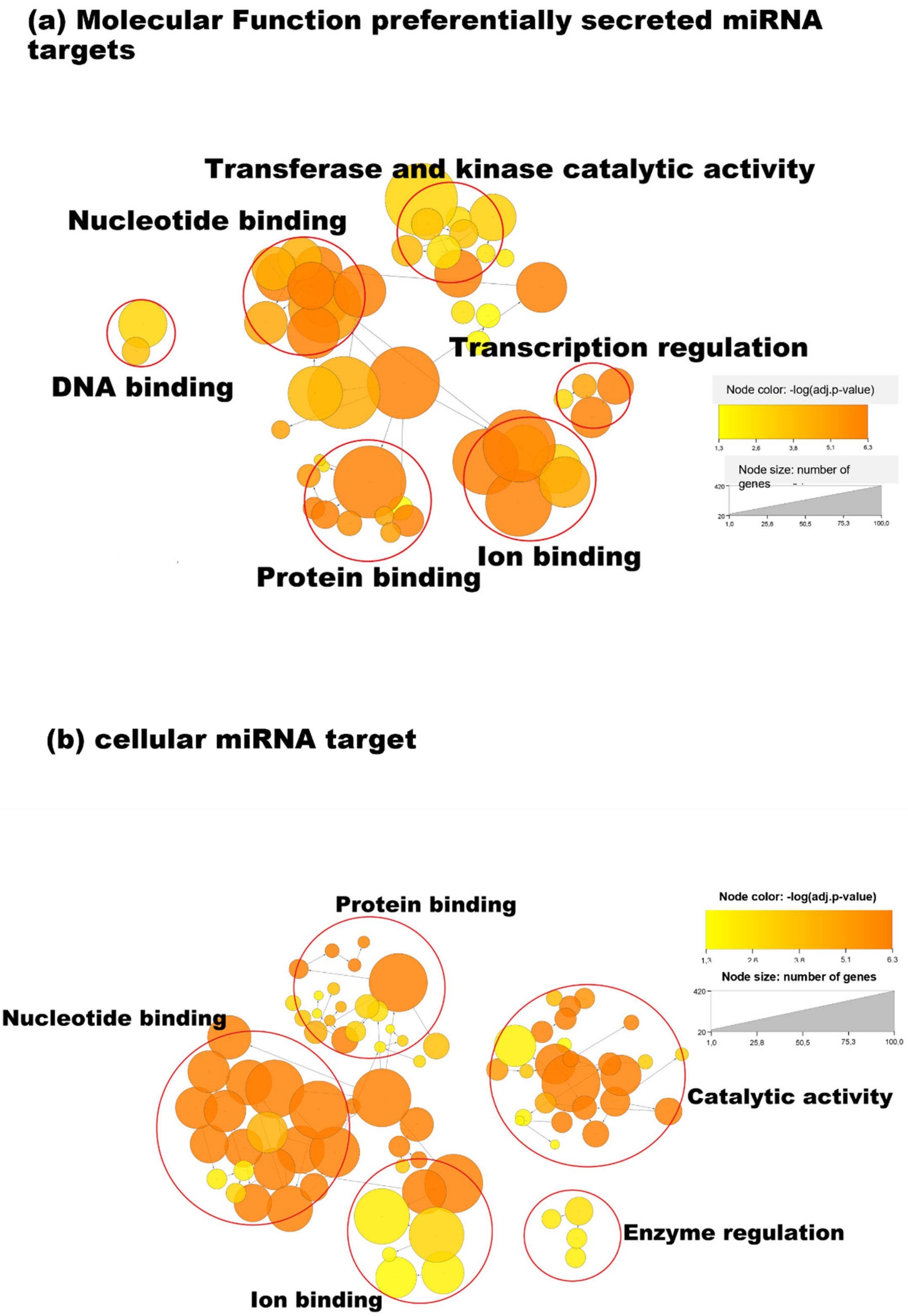

Figure S2. GO molecular function of (a) preferentially secreted miRNA targets (b) cellular miRNA targets.

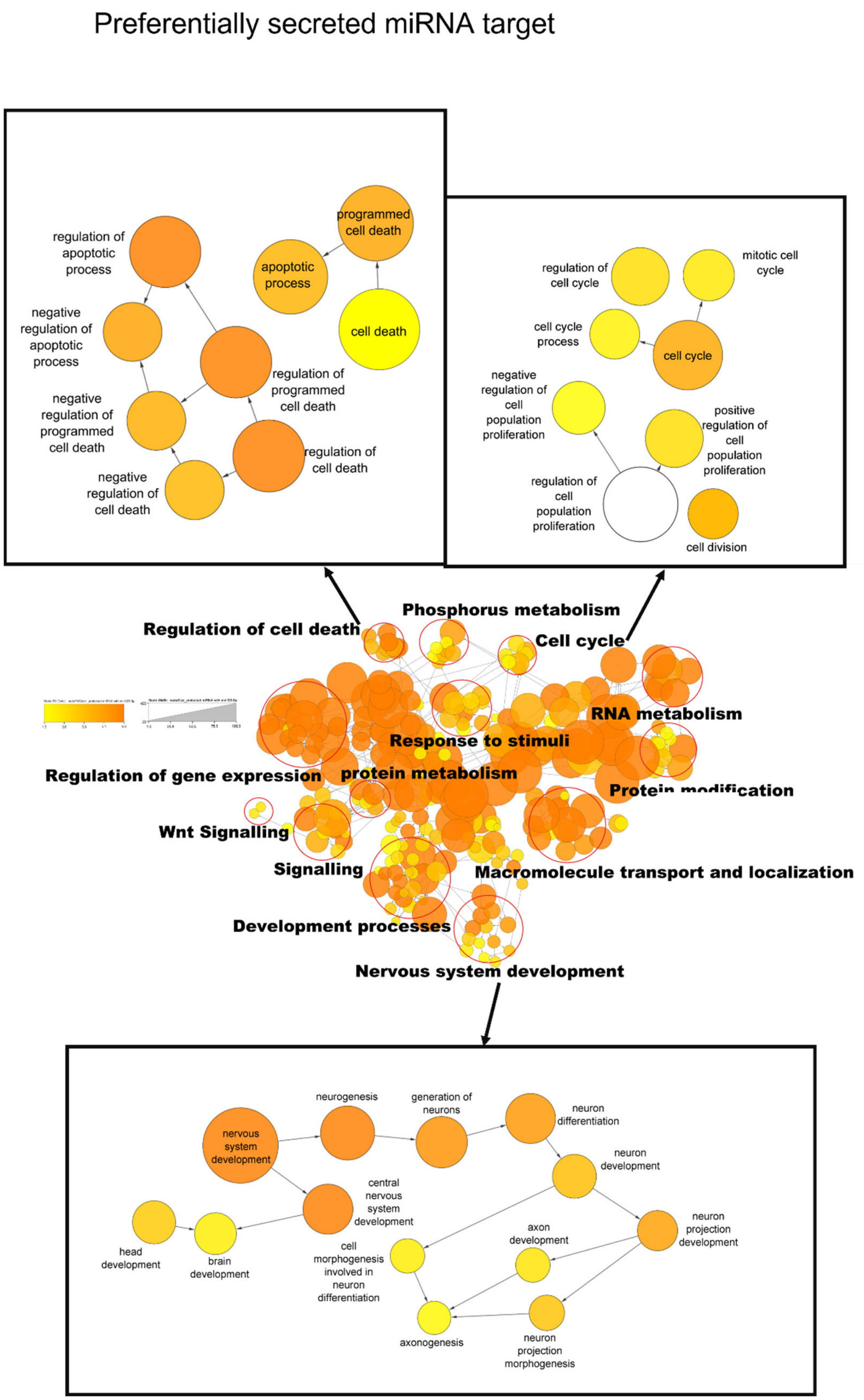

Figure S3. GO biological process of preferentially secreted miRNA targets.

Table S1. Astrocyte mRNA differentially expressed in cells switched to serum free medium for EV collection, compared to full medium with 2% FBS. 24 hour time point. (with cutoff of 0.25 -1.75 fold).

log2FoldChange	pvalue	padj	*gene_name*	gene_description
**6,024426761**	1,92E-05	9,79E-05	*PTGDR2*	prostaglandin D2 receptor 2 [Source:HGNC Symbol;Acc:HGNC:4502]
**5,903303959**	6,44E-06	3,52E-05	*MC5R*	melanocortin 5 receptor [Source:HGNC Symbol;Acc:HGNC:6933]
**5,57609863**	0,00015648	0,000689992	*SGSM1*	small G protein signaling modulator 1 [Source:HGNC Symbol;Acc:HGNC:29410]
**5,498784631**	0,000225193	0,000961242	*C2CD4A*	C2 calcium dependent domain containing 4A [Source:HGNC Symbol;Acc:HGNC:33627]
**5,355542255**	7,46E-05	0,000348818	*MMP1*	matrix metallopeptidase 1 [Source:HGNC Symbol;Acc:HGNC:7155]
**5,330793359**	0,000491287	0,001975532	*DKK4*	dickkopf WNT signaling pathway inhibitor 4 [Source:HGNC Symbol;Acc:HGNC:2894]
**5,273937633**	0,00011772	0,000532014	*WISP2*	WNT1 inducible signaling pathway protein 2 [Source:HGNC Symbol;Acc:HGNC:12770]
**5,17603373**	0,000950909	0,003610515	*CCDC141*	coiled-coil domain containing 141 [Source:HGNC Symbol;Acc:HGNC:26821]
**5,046335776**	0,000308441	0,001285262	*MLXIPL*	MLX interacting protein like [Source:HGNC Symbol;Acc:HGNC:12744]
**4,893743879**	0,000684637	0,002685845	*ABCA6*	ATP binding cassette subfamily A member 6 [Source:HGNC Symbol;Acc:HGNC:36]
**4,833093918**	0,000671547	0,002639133	*MYO3B*	myosin IIIB [Source:HGNC Symbol;Acc:HGNC:15576]
**4,61322166**	0,001664657	0,005997833	*NR0B1*	nuclear receptor subfamily 0 group B member 1 [Source:HGNC Symbol;Acc:HGNC:7960]
**4,209464233**	8,72E-49	3,79E-47	*BRINP2*	BMP/retinoic acid inducible neural specific 2 [Source:HGNC Symbol;Acc:HGNC:13746]
**4,14395554**	3,14E-56	1,63E-54	*OLIG1*	oligodendrocyte transcription factor 1 [Source:HGNC Symbol;Acc:HGNC:16983]
**4,098260245**	9,88E-10	8,12E-09	*VIPR2*	vasoactive intestinal peptide receptor 2 [Source:HGNC Symbol;Acc:HGNC:12695]
**4,045152398**	0,010859416	0,031915468	*GADL1*	glutamate decarboxylase like 1 [Source:HGNC Symbol;Acc:HGNC:27949]
**4,040260302**	0	0	*CALB1*	calbindin 1 [Source:HGNC Symbol;Acc:HGNC:1434]
**3,884674476**	6,39E-56	3,29E-54	*ADRA2B*	adrenoceptor alpha 2B [Source:HGNC Symbol;Acc:HGNC:282]
**3,851778745**	2,95E-164	8,54E-162	*MAP2K6*	mitogen-activated protein kinase kinase 6 [Source:HGNC Symbol;Acc:HGNC:6846]
**3,781991829**	1,02E-07	6,96E-07	*CA4*	carbonic anhydrase 4 [Source:HGNC Symbol;Acc:HGNC:1375]
**3,779053021**	2,56E-20	4,18E-19	*PLXDC1*	plexin domain containing 1 [Source:HGNC Symbol;Acc:HGNC:20945]
**3,755619602**	7,33E-05	0,00034326	*GABRB1*	gamma-aminobutyric acid type A receptor beta1 subunit [Source:HGNC Symbol;Acc:HGNC:4081]
**3,755154428**	6,81E-10	5,69E-09	*SNCB*	synuclein beta [Source:HGNC Symbol;Acc:HGNC:11140]
**3,737647022**	0,000573558	0,002276636	*MYRFL*	myelin regulatory factor like [Source:HGNC Symbol;Acc:HGNC:26316]
**3,706728864**	2,64E-29	6,28E-28	*OLIG2*	oligodendrocyte transcription factor 2 [Source:HGNC Symbol;Acc:HGNC:9398]
**3,687092192**	1,63E-05	8,42E-05	*PRRG3*	proline rich and Gla domain 3 [Source:HGNC Symbol;Acc:HGNC:30798]
**3,674343799**	1,56E-05	8,06E-05	*SHH*	sonic hedgehog [Source:HGNC Symbol;Acc:HGNC:10848]
**3,558490156**	1,65E-12	1,73E-11	*CCK*	cholecystokinin [Source:HGNC Symbol;Acc:HGNC:1569]
**3,532473069**	2,64E-10	2,29E-09	*ERICH3*	glutamate rich 3 [Source:HGNC Symbol;Acc:HGNC:25346]
**3,522316194**	3,80E-52	1,76E-50	*ADCYAP1R1*	ADCYAP receptor type I [Source:HGNC Symbol;Acc:HGNC:242]
**3,479945055**	8,96E-11	8,03E-10	*CA10*	carbonic anhydrase 10 [Source:HGNC Symbol;Acc:HGNC:1369]

**3,47310001**	0,000579658	0,002298965	*NEU4*	neuraminidase 4 [Source:HGNC Symbol;Acc:HGNC:21328]
**3,453308079**	5,16E-26	1,08E-24	*TMPRSS2*	transmembrane serine protease 2 [Source:HGNC Symbol;Acc:HGNC:11876]
**3,444824636**	9,45E-59	5,22E-57	*CPNE5*	copine 5 [Source:HGNC Symbol;Acc:HGNC:2318]
**3,35543286**	0,003626332	0,012114408	*VWC2*	von Willebrand factor C domain containing 2 [Source:HGNC Symbol;Acc:HGNC:30200]
**3,343667605**	8,47E-09	6,42E-08	*PCDHAC1*	protocadherin alpha subfamily C, 1 [Source:HGNC Symbol;Acc:HGNC:8676]
**3,330909049**	3,40E-24	6,62E-23	*NTSR1*	neurotensin receptor 1 [Source:HGNC Symbol;Acc:HGNC:8039]
**3,297461375**	0,017957989	0,049324545	*DSCAM*	DS cell adhesion molecule [Source:HGNC Symbol;Acc:HGNC:3039]
**3,275274791**	1,07E-42	3,93E-41	*SLC7A8*	solute carrier family 7 member 8 [Source:HGNC Symbol;Acc:HGNC:11066]
**3,248694225**	5,79E-24	1,12E-22	*DRD2*	dopamine receptor D2 [Source:HGNC Symbol;Acc:HGNC:3023]
**3,230315602**	1,26E-27	2,79E-26	*RUBCNL*	RUN and cysteine rich domain containing beclin 1 interacting protein like [Source:HGNC Symbol;Acc:HGNC:20420]
**3,214713593**	0	0	*SCG2*	secretogranin II [Source:HGNC Symbol;Acc:HGNC:10575]
**3,19837455**	0,000203752	0,0008775	*CBLN4*	cerebellin 4 precursor [Source:HGNC Symbol;Acc:HGNC:16231]
**3,164646177**	0,007588926	0,023328164	*CDH7*	cadherin 7 [Source:HGNC Symbol;Acc:HGNC:1766]
**3,151079499**	8,36E-25	1,66E-23	*HES5*	hes family bHLH transcription factor 5 [Source:HGNC Symbol;Acc:HGNC:19764]
**3,078845866**	0,00021207	0,00091009	*TACR1*	tachykinin receptor 1 [Source:HGNC Symbol;Acc:HGNC:11526]
**3,078516119**	2,75E-28	6,28E-27	*SEMA3E*	semaphorin 3E [Source:HGNC Symbol;Acc:HGNC:10727]
**3,057703774**	0,00025532	0,001079371	*RIMBP3*	RIMS binding protein 3 [Source:HGNC Symbol;Acc:HGNC:29344]
**3,056063664**	8,17E-12	8,00E-11	*CAMK1G*	calcium/calmodulin dependent protein kinase IG [Source:HGNC Symbol;Acc:HGNC:14585]
**3,018105459**	0,01310987	0,037595705	*KCNK9*	potassium two pore domain channel subfamily K member 9 [Source:HGNC Symbol;Acc:HGNC:6283]
**3,011969235**	0,011212875	0,032844839	*OSR2*	odd-skipped related transciption factor 2 [Source:HGNC Symbol;Acc:HGNC:15830]
**2,960519906**	0,002149355	0,007576818	*GHRHR*	growth hormone releasing hormone receptor [Source:HGNC Symbol;Acc:HGNC:4266]
**2,9593304**	0,013546609	0,038722271	*PRSS2*	serine protease 2 [Source:HGNC Symbol;Acc:HGNC:9483]
**2,94845674**	1,17E-30	2,94E-29	*MAP1LC3C*	microtubule associated protein 1 light chain 3 gamma [Source:HGNC Symbol;Acc:HGNC:13353]
**2,944404114**	3,84E-21	6,54E-20	*DPP4*	dipeptidyl peptidase 4 [Source:HGNC Symbol;Acc:HGNC:3009]
**2,93635343**	0,01441231	0,040788288	*KLHL31*	kelch like family member 31 [Source:HGNC Symbol;Acc:HGNC:21353]
**2,930383925**	1,90E-81	1,64E-79	*UNC5D*	unc-5 netrin receptor D [Source:HGNC Symbol;Acc:HGNC:18634]
**2,899302525**	5,73E-30	1,40E-28	*PTGS2*	prostaglandin-endoperoxide synthase 2 [Source:HGNC Symbol;Acc:HGNC:9605]
**2,895304522**	3,44E-31	8,83E-30	*LRRC10B*	leucine rich repeat containing 10B [Source:HGNC Symbol;Acc:HGNC:37215]
**2,886060061**	3,12E-90	3,17E-88	*ST8SIA5*	ST8 alpha-N-acetyl-neuraminide alpha-2,8-sialyltransferase 5 [Source:HGNC Symbol;Acc:HGNC:17827]
**2,884381878**	2,47E-07	1,61E-06	*PTGDS*	prostaglandin D2 synthase [Source:HGNC Symbol;Acc:HGNC:9592]
**2,87982284**	8,84E-34	2,47E-32	*GRIK1*	glutamate ionotropic receptor kainate type subunit 1 [Source:HGNC Symbol;Acc:HGNC:4579]
**2,874737775**	4,48E-55	2,25E-53	*G0S2*	G0/G1 switch 2 [Source:HGNC Symbol;Acc:HGNC:30229]
**2,838098672**	6,86E-69	4,64E-67	*RHOU*	ras homolog family member U [Source:HGNC Symbol;Acc:HGNC:17794]

**2,788879402**	0,006771367	0,021119439	*SYN2*	synapsin II [Source:HGNC Symbol;Acc:HGNC:11495]
**2,778078029**	9,77E-06	5,21E-05	*AMBP*	alpha-1-microglobulin/bikunin precursor [Source:HGNC Symbol;Acc:HGNC:453]
**2,762765686**	7,74E-30	1,88E-28	*SMOC1*	SPARC related modular calcium binding 1 [Source:HGNC Symbol;Acc:HGNC:20318]
**2,755090286**	2,90E-162	8,04E-160	*STC1*	stanniocalcin 1 [Source:HGNC Symbol;Acc:HGNC:11373]
**2,753224756**	1,13E-12	1,20E-11	*DCC*	DCC netrin 1 receptor [Source:HGNC Symbol;Acc:HGNC:2701]
**2,724127654**	0,00675692	0,021084546	*DAPL1*	death associated protein like 1 [Source:HGNC Symbol;Acc:HGNC:21490]
**2,706964818**	0,000859108	0,003300689	*SLITRK6*	SLIT and NTRK like family member 6 [Source:HGNC Symbol;Acc:HGNC:23503]
**2,704979722**	7,17E-12	7,04E-11	*AMER2*	APC membrane recruitment protein 2 [Source:HGNC Symbol;Acc:HGNC:26360]
**2,695473656**	0,001006019	0,003801908	*SPOCK3*	SPARC (osteonectin), cwcv and kazal like domains proteoglycan 3 [Source:HGNC Symbol;Acc:HGNC:13565]
**2,693742104**	3,05E-32	8,13E-31	*C1QL2*	complement C1q like 2 [Source:HGNC Symbol;Acc:HGNC:24181]
**2,68909377**	6,88E-08	4,78E-07	*CSMD1*	CUB and Sushi multiple domains 1 [Source:HGNC Symbol;Acc:HGNC:14026]
**2,687866482**	2,03E-61	1,21E-59	*ESR2*	estrogen receptor 2 [Source:HGNC Symbol;Acc:HGNC:3468]
**2,677415442**	1,58E-07	1,05E-06	*KCNK15*	potassium two pore domain channel subfamily K member 15 [Source:HGNC Symbol;Acc:HGNC:13814]
**2,675222128**	2,00E-100	2,42E-98	*TMCC3*	transmembrane and coiled-coil domain family 3 [Source:HGNC Symbol;Acc:HGNC:29199]
**2,663419437**	0,007980571	0,024381449	*CACNA1I*	calcium voltage-gated channel subunit alpha1 I [Source:HGNC Symbol;Acc:HGNC:1396]
**2,647521207**	1,54E-23	2,88E-22	*TINCR*	TINCR ubiquitin domain containing [Source:HGNC Symbol;Acc:HGNC:14607]
**2,64142465**	0,001352295	0,004975851	*IRS4*	insulin receptor substrate 4 [Source:HGNC Symbol;Acc:HGNC:6128]
**2,633430502**	2,79E-100	3,35E-98	*TNFRSF1B*	TNF receptor superfamily member 1B [Source:HGNC Symbol;Acc:HGNC:11917]
**2,620474971**	3,36E-07	2,15E-06	*FRK*	fyn related Src family tyrosine kinase [Source:HGNC Symbol;Acc:HGNC:3955]
**2,620454518**	1,62E-12	1,69E-11	*PLPPR5*	phospholipid phosphatase related 5 [Source:HGNC Symbol;Acc:HGNC:31703]
**2,612789469**	0,001636597	0,005905506	*SBK3*	SH3 domain binding kinase family member 3 [Source:HGNC Symbol;Acc:HGNC:44121]
**2,602122154**	0,000168054	0,000736839	*BTBD16*	BTB domain containing 16 [Source:HGNC Symbol;Acc:HGNC:26340]
**2,580283541**	1,52E-09	1,23E-08	*GSX2*	GS homeobox 2 [Source:HGNC Symbol;Acc:HGNC:24959]
**2,568098313**	2,63E-243	1,76E-240	*NOG*	noggin [Source:HGNC Symbol;Acc:HGNC:7866]
**2,567963015**	7,56E-212	3,41E-209	*FAM181B*	family with sequence similarity 181 member B [Source:HGNC Symbol;Acc:HGNC:28512]
**2,567398036**	3,94E-07	2,50E-06	*DLX2*	distal-less homeobox 2 [Source:HGNC Symbol;Acc:HGNC:2915]
**2,56515218**	6,72E-32	1,77E-30	*CHGB*	chromogranin B [Source:HGNC Symbol;Acc:HGNC:1930]
**2,56226494**	1,53E-23	2,86E-22	*WNT4*	Wnt family member 4 [Source:HGNC Symbol;Acc:HGNC:12783]
**2,552369648**	0,003127187	0,010616899	*NAIP*	NLR family apoptosis inhibitory protein [Source:HGNC Symbol;Acc:HGNC:7634]
**2,537634347**	0,000510474	0,002044213	*ALDH8A1*	aldehyde dehydrogenase 8 family member A1 [Source:HGNC Symbol;Acc:HGNC:15471]
**2,525657239**	2,05E-297	2,66E-294	*GAS1*	growth arrest specific 1 [Source:HGNC Symbol;Acc:HGNC:4165]
**2,50784542**	0,00118288	0,00440172	*RAET1G*	retinoic acid early transcript 1G [Source:HGNC Symbol;Acc:HGNC:16795]
**2,507442214**	1,50E-09	1,21E-08	*CREG2*	cellular repressor of E1A stimulated genes 2 [Source:HGNC Symbol;Acc:HGNC:14272]

**2,507403176**	0,000502524	0,002014874	*CLIC2*	chloride intracellular channel 2 [Source:HGNC Symbol;Acc:HGNC:2063]
**2,486474496**	2,15E-08	1,57E-07	*AKR1B10*	aldo-keto reductase family 1 member B10 [Source:HGNC Symbol;Acc:HGNC:382]
**2,477912904**	1,93E-09	1,55E-08	*KRT15*	keratin 15 [Source:HGNC Symbol;Acc:HGNC:6421]
**2,475602791**	3,79E-07	2,42E-06	*AKR1C2*	aldo-keto reductase family 1 member C2 [Source:HGNC Symbol;Acc:HGNC:385]
**2,474043433**	5,91E-06	3,25E-05	*NRROS*	negative regulator of reactive oxygen species [Source:HGNC Symbol;Acc:HGNC:24613]
**2,469592816**	0,001366562	0,005023584	*CHRNA9*	cholinergic receptor nicotinic alpha 9 subunit [Source:HGNC Symbol;Acc:HGNC:14079]
**2,466172504**	1,77E-33	4,89E-32	*NPNT*	nephronectin [Source:HGNC Symbol;Acc:HGNC:27405]
**2,464424131**	7,65E-07	4,71E-06	*LINGO1*	leucine rich repeat and Ig domain containing 1 [Source:HGNC Symbol;Acc:HGNC:21205]
**2,450467735**	1,04E-58	5,76E-57	*ARRB1*	arrestin beta 1 [Source:HGNC Symbol;Acc:HGNC:711]
**2,435321367**	0,000827091	0,003193498	*SPTSSB*	serine palmitoyltransferase small subunit B [Source:HGNC Symbol;Acc:HGNC:24045]
**2,429492985**	0,011171318	0,032752773	*SLC30A10*	solute carrier family 30 member 10 [Source:HGNC Symbol;Acc:HGNC:25355]
**2,429464184**	6,43E-29	1,51E-27	*RAB27B*	RAB27B, member RAS oncogene family [Source:HGNC Symbol;Acc:HGNC:9767]
**2,42797404**	0,00018181	0,000792319	*SYT6*	synaptotagmin 6 [Source:HGNC Symbol;Acc:HGNC:18638]
**2,408173228**	0,006827211	0,021279931	*RASL12*	RAS like family 12 [Source:HGNC Symbol;Acc:HGNC:30289]
**2,405692873**	0,002632383	0,009081603	*GRM8*	glutamate metabotropic receptor 8 [Source:HGNC Symbol;Acc:HGNC:4600]
**2,40315562**	0,000112672	0,000510747	*FGL2*	fibrinogen like 2 [Source:HGNC Symbol;Acc:HGNC:3696]
**2,398162913**	1,63E-15	2,05E-14	*SLC16A6*	solute carrier family 16 member 6 [Source:HGNC Symbol;Acc:HGNC:10927]
**2,396274038**	7,01E-10	5,84E-09	*CPLX2*	complexin 2 [Source:HGNC Symbol;Acc:HGNC:2310]
**2,395614518**	7,11E-54	3,45E-52	*ADAMTS18*	ADAM metallopeptidase with thrombospondin type 1 motif 18 [Source:HGNC Symbol;Acc:HGNC:17110]
**2,386863723**	5,12E-05	0,000246627	*CCDC177*	coiled-coil domain containing 177 [Source:HGNC Symbol;Acc:HGNC:23243]
**2,366648689**	0,00121265	0,004501281	*SLC52A1*	solute carrier family 52 member 1 [Source:HGNC Symbol;Acc:HGNC:30225]
**2,366307084**	1,75E-225	9,46E-223	*LIMD1*	LIM domains containing 1 [Source:HGNC Symbol;Acc:HGNC:6612]
**2,365925083**	0,0002856	0,001195763	*SPATA12*	spermatogenesis associated 12 [Source:HGNC Symbol;Acc:HGNC:23221]
**2,350387438**	2,65E-53	1,27E-51	*AQP1*	aquaporin 1 (Colton blood group) [Source:HGNC Symbol;Acc:HGNC:633]
**2,348101631**	4,88E-59	2,72E-57	*RGS16*	regulator of G protein signaling 16 [Source:HGNC Symbol;Acc:HGNC:9997]
**2,335387634**	0,002035622	0,007208627	*ARL14EPL*	ADP ribosylation factor like GTPase 14 effector protein like [Source:HGNC Symbol;Acc:HGNC:44201]
**2,334365129**	0,006420691	0,020207577	*FRMPD2*	FERM and PDZ domain containing 2 [Source:HGNC Symbol;Acc:HGNC:28572]
**2,318824472**	0,012454415	0,035981892	*GPIHBP1*	glycosylphosphatidylinositol anchored high density lipoprotein binding protein 1 [Source:HGNC Symbol;Acc:HGNC:24945]
**2,309681996**	0,016523514	0,045985275	*C2CD4B*	C2 calcium dependent domain containing 4B [Source:HGNC Symbol;Acc:HGNC:33628]
**2,299480224**	6,71E-05	0,000316832	*KCNK12*	potassium two pore domain channel subfamily K member 12 [Source:HGNC Symbol;Acc:HGNC:6274]
**2,278977939**	0,000116532	0,000527139	*LRRTM3*	leucine rich repeat transmembrane neuronal 3 [Source:HGNC Symbol;Acc:HGNC:19410]
**2,278670962**	0,000373207	0,001533767	*EVI2A*	ecotropic viral integration site 2A [Source:HGNC Symbol;Acc:HGNC:3499]
**2,270680972**	2,93E-41	1,02E-39	*IL13RA2*	interleukin 13 receptor subunit alpha 2 [Source:HGNC Symbol;Acc:HGNC:5975]

**2,264732151**	2,81E-36	8,57E-35	*PROX1*	prospero homeobox 1 [Source:HGNC Symbol;Acc:HGNC:9459]
**2,263936899**	1,57E-130	2,96E-128	*FERMT1*	fermitin family member 1 [Source:HGNC Symbol;Acc:HGNC:15889]
**2,26029233**	2,64E-288	2,69E-285	*ATP1A2*	ATPase Na+/K+ transporting subunit alpha 2 [Source:HGNC Symbol;Acc:HGNC:800]
**2,2578222**	4,27E-05	0,000208152	*RFX8*	RFX family member 8, lacking RFX DNA binding domain [Source:HGNC Symbol;Acc:HGNC:37253]
**2,252872618**	2,05E-28	4,73E-27	*GPR158*	G protein-coupled receptor 158 [Source:HGNC Symbol;Acc:HGNC:23689]
**2,242220883**	3,59E-75	2,69E-73	*ADA*	adenosine deaminase [Source:HGNC Symbol;Acc:HGNC:186]
**2,239559328**	0,004371482	0,014311254	*C3orf80*	chromosome 3 open reading frame 80 [Source:HGNC Symbol;Acc:HGNC:40048]
**2,233555958**	2,49E-18	3,70E-17	*TMC1*	transmembrane channel like 1 [Source:HGNC Symbol;Acc:HGNC:16513]
**2,220901727**	8,79E-14	1,01E-12	*RIMBP2*	RIMS binding protein 2 [Source:HGNC Symbol;Acc:HGNC:30339]
**2,219449381**	3,42E-05	0,00016902	*SERTM1*	serine rich and transmembrane domain containing 1 [Source:HGNC Symbol;Acc:HGNC:33792]
**2,2039749**	1,13E-58	6,18E-57	*EOMES*	eomesodermin [Source:HGNC Symbol;Acc:HGNC:3372]
**2,197850114**	2,92E-37	9,18E-36	*RGS2*	regulator of G protein signaling 2 [Source:HGNC Symbol;Acc:HGNC:9998]
**2,194116655**	9,20E-05	0,000424681	*IL1A*	interleukin 1 alpha [Source:HGNC Symbol;Acc:HGNC:5991]
**2,174582314**	2,28E-22	4,06E-21	*CXXC4*	CXXC finger protein 4 [Source:HGNC Symbol;Acc:HGNC:24593]
**2,170781632**	8,16E-09	6,20E-08	*TTC39A*	tetratricopeptide repeat domain 39A [Source:HGNC Symbol;Acc:HGNC:18657]
**2,163489435**	1,23E-17	1,77E-16	*BAIAP3*	BAI1 associated protein 3 [Source:HGNC Symbol;Acc:HGNC:948]
**2,160695815**	1,39E-21	2,39E-20	*FREM2*	FRAS1 related extracellular matrix protein 2 [Source:HGNC Symbol;Acc:HGNC:25396]
**2,157362705**	3,19E-20	5,17E-19	*CDH23*	cadherin related 23 [Source:HGNC Symbol;Acc:HGNC:13733]
**2,151320251**	6,92E-08	4,81E-07	*ENKUR*	enkurin, TRPC channel interacting protein [Source:HGNC Symbol;Acc:HGNC:28388]
**2,151072686**	5,14E-08	3,62E-07	*CLEC2B*	C-type lectin domain family 2 member B [Source:HGNC Symbol;Acc:HGNC:2053]
**2,144979001**	9,15E-46	3,65E-44	*PTHLH*	parathyroid hormone like hormone [Source:HGNC Symbol;Acc:HGNC:9607]
**2,144559036**	0,000197185	0,000852972	*ABCA9*	ATP binding cassette subfamily A member 9 [Source:HGNC Symbol;Acc:HGNC:39]
**2,139450081**	1,35E-210	5,94E-208	*PPP3CA*	protein phosphatase 3 catalytic subunit alpha [Source:HGNC Symbol;Acc:HGNC:9314]
**2,138003722**	3,76E-05	0,000184549	*ERBB3*	erb-b2 receptor tyrosine kinase 3 [Source:HGNC Symbol;Acc:HGNC:3431]
**2,134847106**	2,35E-05	0,0001187	*NHLRC4*	NHL repeat containing 4 [Source:HGNC Symbol;Acc:HGNC:26700]
**2,133557789**	0,000244415	0,001036975	*GABRA2*	gamma-aminobutyric acid type A receptor alpha2 subunit [Source:HGNC Symbol;Acc:HGNC:4076]
**2,131783742**	4,44E-44	1,69E-42	*TNFRSF11B*	TNF receptor superfamily member 11b [Source:HGNC Symbol;Acc:HGNC:11909]
**2,127909217**	0,000476156	0,001920258	*IL1RAPL1*	interleukin 1 receptor accessory protein like 1 [Source:HGNC Symbol;Acc:HGNC:5996]
**2,125701482**	3,51E-59	1,96E-57	*SPON1*	spondin 1 [Source:HGNC Symbol;Acc:HGNC:11252]
**2,116544701**	2,30E-103	2,88E-101	*CXCR4*	C-X-C motif chemokine receptor 4 [Source:HGNC Symbol;Acc:HGNC:2561]
**2,108999558**	1,53E-115	2,30E-113	*ETV1*	ETS variant 1 [Source:HGNC Symbol;Acc:HGNC:3490]
**2,105471049**	0,003485767	0,011685058	*CYP19A1*	cytochrome P450 family 19 subfamily A member 1 [Source:HGNC Symbol;Acc:HGNC:2594]

**2,103553174**	5,68E-58	3,07E-56	*ENTPD3*	ectonucleoside triphosphate diphosphohydrolase 3 [Source:HGNC Symbol;Acc:HGNC:3365]
**2,098499092**	8,06E-126	1,37E-123	*OSBP2*	oxysterol binding protein 2 [Source:HGNC Symbol;Acc:HGNC:8504]
**2,08953537**	3,79E-21	6,46E-20	*ARHGAP28*	Rho GTPase activating protein 28 [Source:HGNC Symbol;Acc:HGNC:25509]
**2,088444855**	8,40E-17	1,15E-15	*PTPRT*	protein tyrosine phosphatase, receptor type T [Source:HGNC Symbol;Acc:HGNC:9682]
**2,086605919**	4,80E-14	5,58E-13	*KIAA0040*	KIAA0040 [Source:HGNC Symbol;Acc:HGNC:28950]
**2,085319848**	1,72E-07	1,14E-06	*B3GNT7*	UDP-GlcNAc:betaGal beta-1,3-N-acetylglucosaminyltransferase 7 [Source:HGNC Symbol;Acc:HGNC:18811]
**2,075322946**	3,38E-05	0,000167021	*PLEKHD1*	pleckstrin homology and coiled-coil domain containing D1 [Source:HGNC Symbol;Acc:HGNC:20148]
**2,072026825**	9,14E-80	7,61E-78	*ALK*	ALK receptor tyrosine kinase [Source:HGNC Symbol;Acc:HGNC:427]
**2,07097734**	3,40E-44	1,29E-42	*HTRA3*	HtrA serine peptidase 3 [Source:HGNC Symbol;Acc:HGNC:30406]
**2,068370946**	2,10E-142	4,62E-140	*PCDH1*	protocadherin 1 [Source:HGNC Symbol;Acc:HGNC:8655]
**2,06589293**	3,08E-15	3,84E-14	*LEMD1*	LEM domain containing 1 [Source:HGNC Symbol;Acc:HGNC:18725]
**2,044750702**	4,00E-07	2,54E-06	*B3GAT2*	beta-1,3-glucuronyltransferase 2 [Source:HGNC Symbol;Acc:HGNC:922]
**2,040245763**	6,56E-21	1,10E-19	*HDC*	histidine decarboxylase [Source:HGNC Symbol;Acc:HGNC:4855]
**2,039677852**	3,36E-13	3,71E-12	*MYH15*	myosin heavy chain 15 [Source:HGNC Symbol;Acc:HGNC:31073]
**2,039304152**	8,72E-140	1,84E-137	*HMOX1*	heme oxygenase 1 [Source:HGNC Symbol;Acc:HGNC:5013]
**2,036192413**	3,00E-74	2,22E-72	*ESPN*	espin [Source:HGNC Symbol;Acc:HGNC:13281]
**2,035776565**	6,31E-15	7,68E-14	*TLE6*	transducin like enhancer of split 6 [Source:HGNC Symbol;Acc:HGNC:30788]
**2,026590352**	6,98E-56	3,57E-54	*KCNF1*	potassium voltage-gated channel modifier subfamily F member 1 [Source:HGNC Symbol;Acc:HGNC:6246]
**2,025666927**	1,07E-43	4,02E-42	*SLC2A13*	solute carrier family 2 member 13 [Source:HGNC Symbol;Acc:HGNC:15956]
**2,020644524**	5,36E-10	4,51E-09	*FOSB*	FosB proto-oncogene, AP-1 transcription factor subunit [Source:HGNC Symbol;Acc:HGNC:3797]
**2,015298148**	1,94E-09	1,56E-08	*PYGM*	glycogen phosphorylase, muscle associated [Source:HGNC Symbol;Acc:HGNC:9726]
**2,00502089**	8,06E-28	1,80E-26	*AKR1C1*	aldo-keto reductase family 1 member C1 [Source:HGNC Symbol;Acc:HGNC:384]
**1,999664274**	4,81E-35	1,41E-33	*MED12L*	mediator complex subunit 12 like [Source:HGNC Symbol;Acc:HGNC:16050]
**1,985477044**	2,38E-09	1,89E-08	*PHEX*	phosphate regulating endopeptidase homolog X-linked [Source:HGNC Symbol;Acc:HGNC:8918]
**1,982691123**	1,68E-39	5,60E-38	*HAP1*	huntingtin associated protein 1 [Source:HGNC Symbol;Acc:HGNC:4812]
**1,982437505**	0,009315179	0,027921605	*IGFL4*	IGF like family member 4 [Source:HGNC Symbol;Acc:HGNC:32931]
**1,979696573**	2,36E-118	3,60E-116	*CACNA1A*	calcium voltage-gated channel subunit alpha1 A [Source:HGNC Symbol;Acc:HGNC:1388]
**1,977999272**	2,36E-57	1,26E-55	*LAMB3*	laminin subunit beta 3 [Source:HGNC Symbol;Acc:HGNC:6490]
**1,974691007**	1,15E-16	1,56E-15	*NGEF*	neuronal guanine nucleotide exchange factor [Source:HGNC Symbol;Acc:HGNC:7807]
**1,969342012**	8,39E-08	5,76E-07	*INKA2*	inka box actin regulator 2 [Source:HGNC Symbol;Acc:HGNC:28045]
**1,968877502**	6,30E-82	5,45E-80	*GMPR*	guanosine monophosphate reductase [Source:HGNC Symbol;Acc:HGNC:4376]
**1,962532834**	1,63E-26	3,49E-25	*NEK10*	NIMA related kinase 10 [Source:HGNC Symbol;Acc:HGNC:18592]

**1,955799386**	6,19E-31	1,57E-29	*CRYM*	crystallin mu [Source:HGNC Symbol;Acc:HGNC:2418]
**1,945421056**	4,10E-07	2,60E-06	*KCNE3*	potassium voltage-gated channel subfamily E regulatory subunit 3 [Source:HGNC Symbol;Acc:HGNC:6243]
**1,944935024**	5,60E-07	3,50E-06	*ALOX5*	arachidonate 5-lipoxygenase [Source:HGNC Symbol;Acc:HGNC:435]
**1,944081926**	1,54E-10	1,36E-09	*DDIT4L*	DNA damage inducible transcript 4 like [Source:HGNC Symbol;Acc:HGNC:30555]
**1,939395801**	2,60E-06	1,50E-05	*PAK6*	p21 (RAC1) activated kinase 6 [Source:HGNC Symbol;Acc:HGNC:16061]
**1,939255231**	5,94E-06	3,27E-05	*SLC26A9*	solute carrier family 26 member 9 [Source:HGNC Symbol;Acc:HGNC:14469]
**1,938339464**	2,82E-05	0,000141164	*MYH7*	myosin heavy chain 7 [Source:HGNC Symbol;Acc:HGNC:7577]
**1,932942107**	5,24E-05	0,000252048	*C5orf49*	chromosome 5 open reading frame 49 [Source:HGNC Symbol;Acc:HGNC:27028]
**1,918794456**	9,27E-60	5,29E-58	*SYNE3*	spectrin repeat containing nuclear envelope family member 3 [Source:HGNC Symbol;Acc:HGNC:19861]
**1,917938312**	7,13E-221	3,64E-218	*FAM84A*	family with sequence similarity 84 member A [Source:HGNC Symbol;Acc:HGNC:20743]
**1,913831182**	1,69E-51	7,75E-50	*HEY1*	hes related family bHLH transcription factor with YRPW motif 1 [Source:HGNC Symbol;Acc:HGNC:4880]
**1,912717359**	6,71E-29	1,58E-27	*PRKCQ*	protein kinase C theta [Source:HGNC Symbol;Acc:HGNC:9410]
**1,91239052**	2,69E-72	1,95E-70	*LMO2*	LIM domain only 2 [Source:HGNC Symbol;Acc:HGNC:6642]
**1,90328142**	3,06E-101	3,81E-99	*C11orf87*	chromosome 11 open reading frame 87 [Source:HGNC Symbol;Acc:HGNC:33788]
**1,902527453**	3,05E-25	6,18E-24	*TRIL*	TLR4 interactor with leucine rich repeats [Source:HGNC Symbol;Acc:HGNC:22200]
**1,90043919**	4,45E-201	1,76E-198	*COL9A3*	collagen type IX alpha 3 chain [Source:HGNC Symbol;Acc:HGNC:2219]
**1,897735213**	0,004578588	0,014930612	*KIAA1257*	KIAA1257 [Source:HGNC Symbol;Acc:HGNC:29231]
**1,897477354**	1,17E-08	8,75E-08	*PCDHAC2*	protocadherin alpha subfamily C, 2 [Source:HGNC Symbol;Acc:HGNC:8677]
**1,896388867**	0,01227589	0,035556107	*GRIP2*	glutamate receptor interacting protein 2 [Source:HGNC Symbol;Acc:HGNC:23841]
**1,885938319**	1,87E-53	8,93E-52	*SERPINF1*	serpin family F member 1 [Source:HGNC Symbol;Acc:HGNC:8824]
**1,885451536**	3,53E-148	8,05E-146	*TMEM132B*	transmembrane protein 132B [Source:HGNC Symbol;Acc:HGNC:29397]
**1,877282577**	3,07E-06	1,75E-05	*KCNK10*	potassium two pore domain channel subfamily K member 10 [Source:HGNC Symbol;Acc:HGNC:6273]
**1,877161717**	1,22E-16	1,65E-15	*PDK4*	pyruvate dehydrogenase kinase 4 [Source:HGNC Symbol;Acc:HGNC:8812]
**1,871552274**	9,54E-18	1,38E-16	*EML6*	echinoderm microtubule associated protein like 6 [Source:HGNC Symbol;Acc:HGNC:35412]
**1,86699806**	7,69E-28	1,72E-26	*RCOR2*	REST corepressor 2 [Source:HGNC Symbol;Acc:HGNC:27455]
**1,8616223**	1,79E-45	7,07E-44	*COBL*	cordon-bleu WH2 repeat protein [Source:HGNC Symbol;Acc:HGNC:22199]
**1,858500986**	0,000170367	0,000745973	*ABCG1*	ATP binding cassette subfamily G member 1 [Source:HGNC Symbol;Acc:HGNC:73]
**1,858244488**	0,006148459	0,019439066	*KCNN1*	potassium calcium-activated channel subfamily N member 1 [Source:HGNC Symbol;Acc:HGNC:6290]
**1,857645733**	1,10E-95	1,23E-93	*RASD1*	ras related dexamethasone induced 1 [Source:HGNC Symbol;Acc:HGNC:15828]
**1,857370635**	0,014592796	0,041196943	*NHLH2*	nescient helix-loop-helix 2 [Source:HGNC Symbol;Acc:HGNC:7818]
**1,851555256**	2,46E-92	2,60E-90	*APCDD1L*	APC down-regulated 1 like [Source:HGNC Symbol;Acc:HGNC:26892]
**1,833988705**	4,64E-71	3,30E-69	*TENT5A*	terminal nucleotidyltransferase 5A [Source:HGNC Symbol;Acc:HGNC:18345]

**1,829776632**	0,002363208	0,008249761	*GPR146*	G protein-coupled receptor 146 [Source:HGNC Symbol;Acc:HGNC:21718]
**1,82765654**	8,92E-128	1,59E-125	*GCLC*	glutamate-cysteine ligase catalytic subunit [Source:HGNC Symbol;Acc:HGNC:4311]
**1,824259381**	2,28E-10	1,99E-09	*PARM1*	prostate androgen-regulated mucin-like protein 1 [Source:HGNC Symbol;Acc:HGNC:24536]
**1,82374317**	1,10E-14	1,32E-13	*CACNB2*	calcium voltage-gated channel auxiliary subunit beta 2 [Source:HGNC Symbol;Acc:HGNC:1402]
**1,822235223**	3,37E-111	4,70E-109	*PLEKHA1*	pleckstrin homology domain containing A1 [Source:HGNC Symbol;Acc:HGNC:14335]
**1,821640223**	2,83E-08	2,04E-07	*C1orf94*	chromosome 1 open reading frame 94 [Source:HGNC Symbol;Acc:HGNC:28250]
**1,812857796**	1,18E-07	7,94E-07	*ADAMTSL2*	ADAMTS like 2 [Source:HGNC Symbol;Acc:HGNC:14631]
**1,808498156**	6,98E-131	1,33E-128	*COLGALT2*	collagen beta(1-O)galactosyltransferase 2 [Source:HGNC Symbol;Acc:HGNC:16790]
**1,807670379**	4,19E-06	2,34E-05	*HERC5*	HECT and RLD domain containing E3 ubiquitin protein ligase 5 [Source:HGNC Symbol;Acc:HGNC:24368]
**1,804688103**	2,49E-27	5,46E-26	*MT3*	metallothionein 3 [Source:HGNC Symbol;Acc:HGNC:7408]
**1,799185754**	0,013647592	0,038947818	*KCNN2*	potassium calcium-activated channel subfamily N member 2 [Source:HGNC Symbol;Acc:HGNC:6291]
**1,798454864**	0,003663706	0,012224531	*TMEM132C*	transmembrane protein 132C [Source:HGNC Symbol;Acc:HGNC:25436]
**1,796294095**	1,65E-10	1,45E-09	*ANO1*	anoctamin 1 [Source:HGNC Symbol;Acc:HGNC:21625]
**1,793705932**	0,013821602	0,039374928	*SYPL2*	synaptophysin like 2 [Source:HGNC Symbol;Acc:HGNC:27638]
**1,793459517**	3,13E-152	7,89E-150	*DRAXIN*	dorsal inhibitory axon guidance protein [Source:HGNC Symbol;Acc:HGNC:25054]
**1,791363208**	0,000138207	0,000615854	*LRCOL1*	leucine rich colipase like 1 [Source:HGNC Symbol;Acc:HGNC:44160]
**1,787353752**	9,03E-06	4,84E-05	*ARHGAP20*	Rho GTPase activating protein 20 [Source:HGNC Symbol;Acc:HGNC:18357]
**1,786192004**	3,58E-28	8,11E-27	*OSGIN1*	oxidative stress induced growth inhibitor 1 [Source:HGNC Symbol;Acc:HGNC:30093]
**1,785785156**	9,77E-60	5,56E-58	*SPRY1*	sprouty RTK signaling antagonist 1 [Source:HGNC Symbol;Acc:HGNC:11269]
**1,775991989**	4,48E-84	4,08E-82	*SOX13*	SRY-box 13 [Source:HGNC Symbol;Acc:HGNC:11192]
**1,774677848**	4,76E-15	5,85E-14	*PLA2G3*	phospholipase A2 group III [Source:HGNC Symbol;Acc:HGNC:17934]
**1,774322645**	0,000104758	0,000477915	*PENK*	proenkephalin [Source:HGNC Symbol;Acc:HGNC:8831]
**1,772608388**	1,06E-06	6,46E-06	*COL21A1*	collagen type XXI alpha 1 chain [Source:HGNC Symbol;Acc:HGNC:17025]
**1,769517367**	2,20E-06	1,28E-05	*CRB1*	crumbs 1, cell polarity complex component [Source:HGNC Symbol;Acc:HGNC:2343]
**1,768534852**	6,15E-53	2,93E-51	*GRIK2*	glutamate ionotropic receptor kainate type subunit 2 [Source:HGNC Symbol;Acc:HGNC:4580]
**1,76813416**	8,83E-208	3,81E-205	*SERINC5*	serine incorporator 5 [Source:HGNC Symbol;Acc:HGNC:18825]
**1,768091553**	0,001231227	0,004565868	*SPATA6L*	spermatogenesis associated 6 like [Source:HGNC Symbol;Acc:HGNC:25472]
**1,766403911**	1,82E-44	6,97E-43	*FAXDC2*	fatty acid hydroxylase domain containing 2 [Source:HGNC Symbol;Acc:HGNC:1334]
**1,765551579**	0,001742807	0,006249246	*TXLNB*	taxilin beta [Source:HGNC Symbol;Acc:HGNC:21617]
**1,762513124**	0,010185596	0,030191182	*SLC16A11*	solute carrier family 16 member 11 [Source:HGNC Symbol;Acc:HGNC:23093]
**1,761692966**	1,45E-20	2,40E-19	*RASSF10*	Ras association domain family member 10 [Source:HGNC Symbol;Acc:HGNC:33984]
**1,760200871**	1,19E-205	5,01E-203	*HMGA1*	high mobility group AT-hook 1 [Source:HGNC Symbol;Acc:HGNC:5010]
**1,759329346**	1,06E-15	1,35E-14	*HES2*	hes family bHLH transcription factor 2 [Source:HGNC Symbol;Acc:HGNC:16005]

**1,757731011**	4,01E-98	4,72E-96	*APCDD1*	APC down-regulated 1 [Source:HGNC Symbol;Acc:HGNC:15718]
**1,74930107**	0,000104773	0,000477915	*SFN*	stratifin [Source:HGNC Symbol;Acc:HGNC:10773]
**1,742389784**	0,006766317	0,02110708	*ANKRD20A2*	ankyrin repeat domain 20 family member A2 [Source:HGNC Symbol;Acc:HGNC:31979]
**1,735389548**	8,65E-34	2,42E-32	*CALCRL*	calcitonin receptor like receptor [Source:HGNC Symbol;Acc:HGNC:16709]
**1,733449606**	3,02E-06	1,72E-05	*CLDN3*	claudin 3 [Source:HGNC Symbol;Acc:HGNC:2045]
**1,730138002**	2,91E-05	0,000145272	*ABCD2*	ATP binding cassette subfamily D member 2 [Source:HGNC Symbol;Acc:HGNC:66]
**1,714582758**	1,92E-36	5,92E-35	*FOXN2*	forkhead box N2 [Source:HGNC Symbol;Acc:HGNC:5281]
**1,713668784**	8,16E-05	0,000379286	*TMEM100*	transmembrane protein 100 [Source:HGNC Symbol;Acc:HGNC:25607]
**1,712514651**	0,000184929	0,000804289	*ENTPD8*	ectonucleoside triphosphate diphosphohydrolase 8 [Source:HGNC Symbol;Acc:HGNC:24860]
**1,710252936**	1,10E-20	1,83E-19	*GADD45G*	growth arrest and DNA damage inducible gamma [Source:HGNC Symbol;Acc:HGNC:4097]
**1,708390182**	1,42E-05	7,37E-05	*ADCY8*	adenylate cyclase 8 [Source:HGNC Symbol;Acc:HGNC:239]
**1,707047792**	6,01E-05	0,000286159	*COL23A1*	collagen type XXIII alpha 1 chain [Source:HGNC Symbol;Acc:HGNC:22990]
**1,702353803**	2,10E-11	1,99E-10	*ATP8A2*	ATPase phospholipid transporting 8A2 [Source:HGNC Symbol;Acc:HGNC:13533]
**1,700247582**	0,006557772	0,020575606	*SNHG28*	small nucleolar RNA host gene 28 [Source:NCBI gene;Acc:284677]
**1,69868229**	6,13E-79	5,00E-77	*COL22A1*	collagen type XXII alpha 1 chain [Source:HGNC Symbol;Acc:HGNC:22989]
**1,696394568**	1,56E-10	1,38E-09	*NOV*	nephroblastoma overexpressed [Source:HGNC Symbol;Acc:HGNC:7885]
**1,694842384**	0,012841085	0,036912174	*FHAD1*	forkhead associated phosphopeptide binding domain 1 [Source:HGNC Symbol;Acc:HGNC:29408]
**1,691304772**	1,73E-05	8,87E-05	*STXBP5L*	syntaxin binding protein 5 like [Source:HGNC Symbol;Acc:HGNC:30757]
**1,6871501**	1,32E-16	1,78E-15	*ST8SIA4*	ST8 alpha-N-acetyl-neuraminide alpha-2,8-sialyltransferase 4 [Source:HGNC Symbol;Acc:HGNC:10871]
**1,684511896**	0,000947047	0,003600078	*RGS8*	regulator of G protein signaling 8 [Source:HGNC Symbol;Acc:HGNC:16810]
**1,683025245**	1,19E-16	1,60E-15	*DIRAS2*	DIRAS family GTPase 2 [Source:HGNC Symbol;Acc:HGNC:19323]
**1,677051666**	7,24E-25	1,45E-23	*RGS17*	regulator of G protein signaling 17 [Source:HGNC Symbol;Acc:HGNC:14088]
**1,673686591**	0,000864936	0,003320465	*PDZK1*	PDZ domain containing 1 [Source:HGNC Symbol;Acc:HGNC:8821]
**1,654795733**	2,27E-203	9,16E-201	*NQO1*	NAD(P)H quinone dehydrogenase 1 [Source:HGNC Symbol;Acc:HGNC:2874]
**1,649102828**	8,79E-11	7,88E-10	*CACNA1G*	calcium voltage-gated channel subunit alpha1 G [Source:HGNC Symbol;Acc:HGNC:1394]
**1,649088286**	2,75E-27	6,03E-26	*TMEM154*	transmembrane protein 154 [Source:HGNC Symbol;Acc:HGNC:26489]
**1,648562685**	4,80E-14	5,58E-13	*CCDC102B*	coiled-coil domain containing 102B [Source:HGNC Symbol;Acc:HGNC:26295]
**1,644827442**	2,63E-12	2,71E-11	*GCK*	glucokinase [Source:HGNC Symbol;Acc:HGNC:4195]
**1,641725232**	7,48E-21	1,25E-19	*PCDHGC5*	protocadherin gamma subfamily C, 5 [Source:HGNC Symbol;Acc:HGNC:8718]
**1,641183378**	5,96E-59	3,31E-57	*SEZ6L*	seizure related 6 homolog like [Source:HGNC Symbol;Acc:HGNC:10763]
**1,637200138**	9,56E-11	8,54E-10	*SLC9A9*	solute carrier family 9 member A9 [Source:HGNC Symbol;Acc:HGNC:20653]
**1,636728383**	9,08E-76	6,89E-74	*PCDH19*	protocadherin 19 [Source:HGNC Symbol;Acc:HGNC:14270]

**1,636494366**	8,57E-81	7,26E-79	*PITPNC1*	phosphatidylinositol transfer protein cytoplasmic 1 [Source:HGNC Symbol;Acc:HGNC:21045]
**1,635746519**	0,003902943	0,01293604	*SLC22A18AS*	solute carrier family 22 member 18 antisense [Source:HGNC Symbol;Acc:HGNC:10965]
**1,633332147**	4,27E-25	8,58E-24	*PCSK9*	proprotein convertase subtilisin/kexin type 9 [Source:HGNC Symbol;Acc:HGNC:20001]
**1,6329103**	0,001006484	0,003802926	*DNAAF3*	dynein axonemal assembly factor 3 [Source:HGNC Symbol;Acc:HGNC:30492]
**1,632891006**	4,54E-129	8,30E-127	*BMP2*	bone morphogenetic protein 2 [Source:HGNC Symbol;Acc:HGNC:1069]
**1,631825248**	6,81E-12	6,71E-11	*FABP3*	fatty acid binding protein 3 [Source:HGNC Symbol;Acc:HGNC:3557]
**1,629875655**	1,72E-21	2,96E-20	*AMPD3*	adenosine monophosphate deaminase 3 [Source:HGNC Symbol;Acc:HGNC:470]
**1,626112882**	2,60E-44	9,90E-43	*CA11*	carbonic anhydrase 11 [Source:HGNC Symbol;Acc:HGNC:1370]
**1,625304713**	2,23E-127	3,91E-125	*CA12*	carbonic anhydrase 12 [Source:HGNC Symbol;Acc:HGNC:1371]
**1,624720727**	7,94E-06	4,29E-05	*BCO1*	beta-carotene oxygenase 1 [Source:HGNC Symbol;Acc:HGNC:13815]
**1,624260134**	1,71E-85	1,61E-83	*PDGFRA*	platelet derived growth factor receptor alpha [Source:HGNC Symbol;Acc:HGNC:8803]
**1,624234331**	0,005443789	0,01741546	*GABRR2*	gamma-aminobutyric acid type A receptor rho2 subunit [Source:HGNC Symbol;Acc:HGNC:4091]
**1,621329815**	1,29E-53	6,21E-52	*C1QTNF1*	C1q and TNF related 1 [Source:HGNC Symbol;Acc:HGNC:14324]
**1,616012266**	7,84E-89	7,80E-87	*KCNN4*	potassium calcium-activated channel subfamily N member 4 [Source:HGNC Symbol;Acc:HGNC:6293]
**1,615419429**	7,55E-238	4,58E-235	*IGFBP5*	insulin like growth factor binding protein 5 [Source:HGNC Symbol;Acc:HGNC:5474]
**1,61379757**	6,20E-15	7,55E-14	*RORB*	RAR related orphan receptor B [Source:HGNC Symbol;Acc:HGNC:10259]
**1,612929425**	4,19E-08	2,96E-07	*RENBP*	renin binding protein [Source:HGNC Symbol;Acc:HGNC:9959]
**1,612834338**	1,19E-158	3,12E-156	*ZNF436*	zinc finger protein 436 [Source:HGNC Symbol;Acc:HGNC:20814]
**1,612718123**	0,000188807	0,000819869	*KANK3*	KN motif and ankyrin repeat domains 3 [Source:HGNC Symbol;Acc:HGNC:24796]
**1,609905406**	8,04E-23	1,46E-21	*PANX2*	pannexin 2 [Source:HGNC Symbol;Acc:HGNC:8600]
**1,609609652**	6,14E-80	5,13E-78	*MAMDC2*	MAM domain containing 2 [Source:HGNC Symbol;Acc:HGNC:23673]
**1,607647554**	0,000551585	0,002196155	*GAS2*	growth arrest specific 2 [Source:HGNC Symbol;Acc:HGNC:4167]
**1,604579354**	5,93E-06	3,26E-05	*JAKMIP3*	Janus kinase and microtubule interacting protein 3 [Source:HGNC Symbol;Acc:HGNC:23523]
**1,600422591**	2,37E-12	2,46E-11	*CTXND1*	cortexin domain containing 1 [Source:HGNC Symbol;Acc:HGNC:50507]
**1,596268359**	1,87E-108	2,49E-106	*ZIC1*	Zic family member 1 [Source:HGNC Symbol;Acc:HGNC:12872]
**1,59219338**	0	0	*TSC22D1*	TSC22 domain family member 1 [Source:HGNC Symbol;Acc:HGNC:16826]
**1,590068003**	5,07E-86	4,82E-84	*TACC2*	transforming acidic coiled-coil containing protein 2 [Source:HGNC Symbol;Acc:HGNC:11523]
**1,580358544**	0,012998803	0,037328383	*FAM110C*	family with sequence similarity 110 member C [Source:HGNC Symbol;Acc:HGNC:33340]
**1,576350678**	3,26E-46	1,32E-44	*NOVA1*	NOVA alternative splicing regulator 1 [Source:HGNC Symbol;Acc:HGNC:7886]
**1,573202315**	0,014969395	0,042106983	*ASIC4*	acid sensing ion channel subunit family member 4 [Source:HGNC Symbol;Acc:HGNC:21263]
**1,569731971**	8,07E-20	1,29E-18	*NMNAT2*	nicotinamide nucleotide adenylyltransferase 2 [Source:HGNC Symbol;Acc:HGNC:16789]

**1,569026811**	0,008437604	0,025636472	*SLC11A1*	solute carrier family 11 member 1 [Source:HGNC Symbol;Acc:HGNC:10907]
**1,565903964**	4,04E-32	1,07E-30	*TMEM51*	transmembrane protein 51 [Source:HGNC Symbol;Acc:HGNC:25488]
**1,564862121**	0,00118247	0,004401038	*CCDC17*	coiled-coil domain containing 17 [Source:HGNC Symbol;Acc:HGNC:26574]
**1,564047331**	3,05E-141	6,51E-139	*KITLG*	KIT ligand [Source:HGNC Symbol;Acc:HGNC:6343]
**1,563162998**	1,50E-05	7,78E-05	*ANGPTL1*	angiopoietin like 1 [Source:HGNC Symbol;Acc:HGNC:489]
**1,560913299**	3,04E-06	1,73E-05	*ASGR1*	asialoglycoprotein receptor 1 [Source:HGNC Symbol;Acc:HGNC:742]
**1,559696107**	0,000172079	0,000752959	*FUT9*	fucosyltransferase 9 [Source:HGNC Symbol;Acc:HGNC:4020]
**1,554154774**	1,41E-150	3,41E-148	*ETV5*	ETS variant 5 [Source:HGNC Symbol;Acc:HGNC:3494]
**1,553913089**	0,006968397	0,021643521	*CLDND2*	claudin domain containing 2 [Source:HGNC Symbol;Acc:HGNC:28511]
**1,553141359**	8,75E-71	6,17E-69	*SATB1*	SATB homeobox 1 [Source:HGNC Symbol;Acc:HGNC:10541]
**1,549685298**	1,21E-15	1,54E-14	*LNX1*	ligand of numb-protein X 1 [Source:HGNC Symbol;Acc:HGNC:6657]
**1,547222436**	8,98E-85	8,22E-83	*EFR3B*	EFR3 homolog B [Source:HGNC Symbol;Acc:HGNC:29155]
**1,546036553**	1,49E-06	8,88E-06	*PIP5K1B*	phosphatidylinositol-4-phosphate 5-kinase type 1 beta [Source:HGNC Symbol;Acc:HGNC:8995]
**1,543701099**	3,68E-08	2,63E-07	*COL17A1*	collagen type XVII alpha 1 chain [Source:HGNC Symbol;Acc:HGNC:2194]
**1,538302011**	1,15E-12	1,22E-11	*RHBDL3*	rhomboid like 3 [Source:HGNC Symbol;Acc:HGNC:16502]
**1,537882283**	0,002865245	0,009811706	*CD226*	CD226 molecule [Source:HGNC Symbol;Acc:HGNC:16961]
**1,537734052**	5,57E-63	3,43E-61	*ADM*	adrenomedullin [Source:HGNC Symbol;Acc:HGNC:259]
**1,533960308**	5,07E-08	3,57E-07	*SPSB4*	splA/ryanodine receptor domain and SOCS box containing 4 [Source:HGNC Symbol;Acc:HGNC:30630]
**1,531108895**	5,51E-54	2,69E-52	*DUSP6*	dual specificity phosphatase 6 [Source:HGNC Symbol;Acc:HGNC:3072]
**1,529650011**	2,53E-29	6,05E-28	*P2RX6*	purinergic receptor P2X 6 [Source:HGNC Symbol;Acc:HGNC:8538]
**1,52690349**	1,54E-82	1,36E-80	*ABCA1*	ATP binding cassette subfamily A member 1 [Source:HGNC Symbol;Acc:HGNC:29]
**1,525000531**	1,08E-07	7,30E-07	*ZMAT4*	zinc finger matrin-type 4 [Source:HGNC Symbol;Acc:HGNC:25844]
**1,51891044**	5,00E-07	3,14E-06	*THEMIS2*	thymocyte selection associated family member 2 [Source:HGNC Symbol;Acc:HGNC:16839]
**1,51271283**	0,008454324	0,025679231	*SLC22A1*	solute carrier family 22 member 1 [Source:HGNC Symbol;Acc:HGNC:10963]
**1,510863842**	0,003448136	0,011567393	*HEY2*	hes related family bHLH transcription factor with YRPW motif 2 [Source:HGNC Symbol;Acc:HGNC:4881]
**1,507622888**	7,54E-29	1,77E-27	*ANKRD44*	ankyrin repeat domain 44 [Source:HGNC Symbol;Acc:HGNC:25259]
**1,504535177**	2,81E-05	0,000140586	*N4BP2L1*	NEDD4 binding protein 2 like 1 [Source:HGNC Symbol;Acc:HGNC:25037]
**1,502455442**	1,25E-247	9,67E-245	*ITPRID2*	ITPR interacting domain containing 2 [Source:HGNC Symbol;Acc:HGNC:11319]
**1,498646386**	0,010660542	0,031397552	*C2orf50*	chromosome 2 open reading frame 50 [Source:HGNC Symbol;Acc:HGNC:26324]
**1,496928807**	6,82E-14	7,84E-13	*RGCC*	regulator of cell cycle [Source:HGNC Symbol;Acc:HGNC:20369]
**1,496022236**	6,26E-07	3,89E-06	*SH3TC1*	SH3 domain and tetratricopeptide repeats 1 [Source:HGNC Symbol;Acc:HGNC:26009]
**1,492918839**	9,03E-12	8,81E-11	*SUSD4*	sushi domain containing 4 [Source:HGNC Symbol;Acc:HGNC:25470]

**1,492713698**	4,82E-12	4,82E-11	*MCTP1*	multiple C2 and transmembrane domain containing 1 [Source:HGNC Symbol;Acc:HGNC:26183]
**1,48929263**	1,23E-08	9,18E-08	*HIST1H3H*	histone cluster 1 H3 family member h [Source:HGNC Symbol;Acc:HGNC:4775]
**1,48670599**	5,32E-12	5,30E-11	*TCEA3*	transcription elongation factor A3 [Source:HGNC Symbol;Acc:HGNC:11615]
**1,484332025**	0,000798106	0,003090193	*ZDHHC22*	zinc finger DHHC-type containing 22 [Source:HGNC Symbol;Acc:HGNC:20106]
**1,479349493**	2,98E-08	2,14E-07	*NEUROG2*	neurogenin 2 [Source:HGNC Symbol;Acc:HGNC:13805]
**1,478308769**	8,09E-05	0,000376278	*ZCCHC12*	zinc finger CCHC-type containing 12 [Source:HGNC Symbol;Acc:HGNC:27273]
**1,475953144**	2,83E-148	6,54E-146	*LRIG1*	leucine rich repeats and immunoglobulin like domains 1 [Source:HGNC Symbol;Acc:HGNC:17360]
**1,475583804**	0,004820494	0,01562494	*FER1L6*	fer-1 like family member 6 [Source:HGNC Symbol;Acc:HGNC:28065]
**1,471815932**	1,07E-07	7,29E-07	*MOB3B*	MOB kinase activator 3B [Source:HGNC Symbol;Acc:HGNC:23825]
**1,469951074**	4,89E-36	1,47E-34	*KCNB1*	potassium voltage-gated channel subfamily B member 1 [Source:HGNC Symbol;Acc:HGNC:6231]
**1,468960923**	0,000130562	0,000585822	*PTGFR*	prostaglandin F receptor [Source:HGNC Symbol;Acc:HGNC:9600]
**1,459108705**	5,87E-62	3,55E-60	*SNED1*	sushi, nidogen and EGF like domains 1 [Source:HGNC Symbol;Acc:HGNC:24696]
**1,456453516**	9,26E-12	9,03E-11	*IL6R*	interleukin 6 receptor [Source:HGNC Symbol;Acc:HGNC:6019]
**1,456023492**	2,42E-205	9,98E-203	*MSI2*	musashi RNA binding protein 2 [Source:HGNC Symbol;Acc:HGNC:18585]
**1,452724651**	1,06E-212	4,90E-210	*SPP1*	secreted phosphoprotein 1 [Source:HGNC Symbol;Acc:HGNC:11255]
**1,45100342**	4,79E-84	4,34E-82	*RFFL*	ring finger and FYVE like domain containing E3 ubiquitin protein ligase [Source:HGNC Symbol;Acc:HGNC:24821]
**1,450490929**	3,41E-110	4,63E-108	*ARHGEF6*	Rac/Cdc42 guanine nucleotide exchange factor 6 [Source:HGNC Symbol;Acc:HGNC:685]
**1,448455073**	0,000161236	0,000709493	*TCERG1L*	transcription elongation regulator 1 like [Source:HGNC Symbol;Acc:HGNC:23533]
**1,442347222**	2,84E-20	4,63E-19	*NDNF*	neuron derived neurotrophic factor [Source:HGNC Symbol;Acc:HGNC:26256]
**1,439349981**	0,001443382	0,005276992	*SH3GL2*	SH3 domain containing GRB2 like 2, endophilin A1 [Source:HGNC Symbol;Acc:HGNC:10831]
**1,438390628**	5,02E-26	1,05E-24	*SLC14A1*	solute carrier family 14 member 1 (Kidd blood group) [Source:HGNC Symbol;Acc:HGNC:10918]
**1,432726131**	0,000286877	0,001200329	*KIAA1211L*	KIAA1211 like [Source:HGNC Symbol;Acc:HGNC:33454]
**1,431859918**	3,20E-109	4,28E-107	*ITPKB*	inositol-trisphosphate 3-kinase B [Source:HGNC Symbol;Acc:HGNC:6179]
**1,429118807**	1,29E-56	6,80E-55	*CHST11*	carbohydrate sulfotransferase 11 [Source:HGNC Symbol;Acc:HGNC:17422]
**1,428241033**	6,92E-52	3,18E-50	*DOCK4*	dedicator of cytokinesis 4 [Source:HGNC Symbol;Acc:HGNC:19192]
**1,427621342**	1,46E-67	9,68E-66	*ASTN1*	astrotactin 1 [Source:HGNC Symbol;Acc:HGNC:773]
**1,423582803**	1,84E-08	1,35E-07	*CPAMD8*	C3 and PZP like, alpha-2-macroglobulin domain containing 8 [Source:HGNC Symbol;Acc:HGNC:23228]
**1,422996291**	1,25E-47	5,29E-46	*RGS20*	regulator of G protein signaling 20 [Source:HGNC Symbol;Acc:HGNC:14600]
**1,421725003**	6,63E-41	2,29E-39	*GEM*	GTP binding protein overexpressed in skeletal muscle [Source:HGNC Symbol;Acc:HGNC:4234]
**1,421122652**	0,016992867	0,047028503	*TBC1D30*	TBC1 domain family member 30 [Source:HGNC Symbol;Acc:HGNC:29164]
**1,420498186**	3,48E-60	2,01E-58	*GMDS*	GDP-mannose 4,6-dehydratase [Source:HGNC Symbol;Acc:HGNC:4369]
**1,420295036**	1,05E-07	7,16E-07	*TNXB*	tenascin XB [Source:HGNC Symbol;Acc:HGNC:11976]
**1,419468927**	0,000198802	0,000859109	*ADAMTS4*	ADAM metallopeptidase with thrombospondin type 1 motif 4 [Source:HGNC Symbol;Acc:HGNC:220]

**1,417640731**	2,03E-12	2,12E-11	*RBM47*	RNA binding motif protein 47 [Source:HGNC Symbol;Acc:HGNC:30358]
**1,417540985**	5,54E-18	8,11E-17	*IFI44L*	interferon induced protein 44 like [Source:HGNC Symbol;Acc:HGNC:17817]
**1,417134541**	0,006665358	0,020862572	*LHFPL3*	LHFPL tetraspan subfamily member 3 [Source:HGNC Symbol;Acc:HGNC:6589]
**1,417029113**	2,51E-28	5,74E-27	*NPTX2*	neuronal pentraxin 2 [Source:HGNC Symbol;Acc:HGNC:7953]
**1,416065781**	0,002464787	0,008573518	*FNDC11*	fibronectin type III domain containing 11 [Source:HGNC Symbol;Acc:HGNC:28764]
**1,414215634**	2,72E-200	1,05E-197	*HTRA1*	HtrA serine peptidase 1 [Source:HGNC Symbol;Acc:HGNC:9476]
**1,413948511**	2,97E-12	3,04E-11	*THSD7A*	thrombospondin type 1 domain containing 7A [Source:HGNC Symbol;Acc:HGNC:22207]
**1,412482303**	0,008887038	0,026813099	*TH*	tyrosine hydroxylase [Source:HGNC Symbol;Acc:HGNC:11782]
**1,410427948**	1,15E-60	6,73E-59	*CSDC2*	cold shock domain containing C2 [Source:HGNC Symbol;Acc:HGNC:30359]
**1,409864889**	3,78E-121	5,96E-119	*ADD3*	adducin 3 [Source:HGNC Symbol;Acc:HGNC:245]
**1,409265894**	0,001552363	0,005635083	*ADAMTS19*	ADAM metallopeptidase with thrombospondin type 1 motif 19 [Source:HGNC Symbol;Acc:HGNC:17111]
**1,406210198**	2,44E-98	2,89E-96	*BTG1*	BTG anti-proliferation factor 1 [Source:HGNC Symbol;Acc:HGNC:1130]
**1,402520967**	5,33E-70	3,68E-68	*TRIM9*	tripartite motif containing 9 [Source:HGNC Symbol;Acc:HGNC:16288]
**1,400284885**	1,29E-05	6,75E-05	*WNT11*	Wnt family member 11 [Source:HGNC Symbol;Acc:HGNC:12776]
**1,398631659**	0,004949674	0,016003561	*CACNA1D*	calcium voltage-gated channel subunit alpha1 D [Source:HGNC Symbol;Acc:HGNC:1391]
**1,39166917**	4,87E-05	0,000235226	*HCN1*	hyperpolarization activated cyclic nucleotide gated potassium channel 1 [Source:HGNC Symbol;Acc:HGNC:4845]
**1,38845543**	1,14E-11	1,10E-10	*ERC2*	ELKS/RAB6-interacting/CAST family member 2 [Source:HGNC Symbol;Acc:HGNC:31922]
**1,384516966**	0,000645461	0,002545899	*CDNF*	cerebral dopamine neurotrophic factor [Source:HGNC Symbol;Acc:HGNC:24913]
**1,382834412**	7,73E-21	1,29E-19	*TMEM63C*	transmembrane protein 63C [Source:HGNC Symbol;Acc:HGNC:23787]
**1,382672126**	3,30E-12	3,36E-11	*MGAT4A*	alpha-1,3-mannosyl-glycoprotein 4-beta-N-acetylglucosaminyltransferase A [Source:HGNC Symbol;Acc:HGNC:7047]
**1,380779662**	1,70E-115	2,54E-113	*SRPX*	sushi repeat containing protein X-linked [Source:HGNC Symbol;Acc:HGNC:11309]
**1,380564643**	2,61E-07	1,70E-06	*ADGRB1*	adhesion G protein-coupled receptor B1 [Source:HGNC Symbol;Acc:HGNC:943]
**1,380233113**	1,91E-95	2,11E-93	*UBASH3B*	ubiquitin associated and SH3 domain containing B [Source:HGNC Symbol;Acc:HGNC:29884]
**1,380067719**	0,014636572	0,041302502	*STUM*	stum, mechanosensory transduction mediator homolog [Source:HGNC Symbol;Acc:HGNC:30491]
**1,378842714**	4,11E-79	3,38E-77	*SPRY4*	sprouty RTK signaling antagonist 4 [Source:HGNC Symbol;Acc:HGNC:15533]
**1,377976038**	2,02E-45	7,95E-44	*BCL2L11*	BCL2 like 11 [Source:HGNC Symbol;Acc:HGNC:994]
**1,376714453**	4,87E-06	2,70E-05	*KCNQ3*	potassium voltage-gated channel subfamily Q member 3 [Source:HGNC Symbol;Acc:HGNC:6297]
**1,375685203**	3,93E-06	2,21E-05	*MERTK*	MER proto-oncogene, tyrosine kinase [Source:HGNC Symbol;Acc:HGNC:7027]
**1,374940309**	5,88E-14	6,80E-13	*EFNA5*	ephrin A5 [Source:HGNC Symbol;Acc:HGNC:3225]
**1,374605581**	7,86E-08	5,42E-07	*VAV3*	vav guanine nucleotide exchange factor 3 [Source:HGNC Symbol;Acc:HGNC:12659]
**1,372915098**	0,00020084	0,00086592	*LRRK2*	leucine rich repeat kinase 2 [Source:HGNC Symbol;Acc:HGNC:18618]

**1,370505662**	2,12E-51	9,68E-50	*HIVEP3*	human immunodeficiency virus type I enhancer binding protein 3 [Source:HGNC Symbol;Acc:HGNC:13561]
**1,369217915**	1,37E-16	1,84E-15	*TMEM163*	transmembrane protein 163 [Source:HGNC Symbol;Acc:HGNC:25380]
**1,364784467**	7,23E-30	1,76E-28	*RRAGD*	Ras related GTP binding D [Source:HGNC Symbol;Acc:HGNC:19903]
**1,363407717**	7,33E-17	1,01E-15	*PLCD4*	phospholipase C delta 4 [Source:HGNC Symbol;Acc:HGNC:9062]
**1,362937078**	7,23E-09	5,52E-08	*UBXN10*	UBX domain protein 10 [Source:HGNC Symbol;Acc:HGNC:26354]
**1,362768794**	0,000940677	0,003582177	*PVRIG*	PVR related immunoglobulin domain containing [Source:HGNC Symbol;Acc:HGNC:32190]
**1,361612321**	9,28E-09	7,02E-08	*RAB3IL1*	RAB3A interacting protein like 1 [Source:HGNC Symbol;Acc:HGNC:9780]
**1,360020803**	2,06E-09	1,64E-08	*KLHDC8A*	kelch domain containing 8A [Source:HGNC Symbol;Acc:HGNC:25573]
**1,359701203**	0,0010047	0,003798405	*EYA1*	EYA transcriptional coactivator and phosphatase 1 [Source:HGNC Symbol;Acc:HGNC:3519]
**1,356209195**	4,77E-14	5,55E-13	*IQCN*	IQ motif containing N [Source:HGNC Symbol;Acc:HGNC:29350]
**1,353775132**	1,86E-26	3,96E-25	*MCF2L*	MCF,2 cell line derived transforming sequence like [Source:HGNC Symbol;Acc:HGNC:14576]
**1,352119757**	3,54E-24	6,88E-23	*DLGAP1*	DLG associated protein 1 [Source:HGNC Symbol;Acc:HGNC:2905]
**1,351622946**	0,010691552	0,031469779	*C7*	complement C7 [Source:HGNC Symbol;Acc:HGNC:1346]
**1,349862955**	2,09E-27	4,61E-26	*TFPI2*	tissue factor pathway inhibitor 2 [Source:HGNC Symbol;Acc:HGNC:11761]
**1,349393715**	2,52E-09	2,01E-08	*ST6GALNAC5*	ST6 N-acetylgalactosaminide alpha-2,6-sialyltransferase 5 [Source:HGNC Symbol;Acc:HGNC:19342]
**1,347481007**	5,38E-84	4,86E-82	*TCN2*	transcobalamin 2 [Source:HGNC Symbol;Acc:HGNC:11653]
**1,346312571**	8,59E-12	8,40E-11	*METTL7B*	methyltransferase like 7B [Source:HGNC Symbol;Acc:HGNC:28276]
**1,345163949**	5,54E-22	9,70E-21	*GLCCI1*	glucocorticoid induced 1 [Source:HGNC Symbol;Acc:HGNC:18713]
**1,342476835**	1,36E-05	7,11E-05	*YPEL4*	yippee like 4 [Source:HGNC Symbol;Acc:HGNC:18328]
**1,338988145**	2,52E-28	5,76E-27	*FAM49A*	family with sequence similarity 49 member A [Source:HGNC Symbol;Acc:HGNC:25373]
**1,337346656**	2,62E-17	3,70E-16	*GPNMB*	glycoprotein nmb [Source:HGNC Symbol;Acc:HGNC:4462]
**1,33495626**	0,00728276	0,022508239	*SLCO4C1*	solute carrier organic anion transporter family member 4C1 [Source:HGNC Symbol;Acc:HGNC:23612]
**1,334495427**	0,005892807	0,018716174	*ATP2A3*	ATPase sarcoplasmic/endoplasmic reticulum Ca2+ transporting 3 [Source:HGNC Symbol;Acc:HGNC:813]
**1,334054653**	5,28E-31	1,34E-29	*INSM1*	INSM transcriptional repressor 1 [Source:HGNC Symbol;Acc:HGNC:6090]
**1,333479684**	1,27E-20	2,10E-19	*GPAT3*	glycerol-3-phosphate acyltransferase 3 [Source:HGNC Symbol;Acc:HGNC:28157]
**1,332368694**	8,23E-14	9,43E-13	*ATP8A1*	ATPase phospholipid transporting 8A1 [Source:HGNC Symbol;Acc:HGNC:13531]
**1,331072716**	0,000924773	0,00352854	*RIBC1*	RIB43A domain with coiled-coils 1 [Source:HGNC Symbol;Acc:HGNC:26537]
**1,330213982**	0,001320121	0,004864845	*ASXL3*	ASXL transcriptional regulator 3 [Source:HGNC Symbol;Acc:HGNC:29357]
**1,329826833**	8,86E-149	2,07E-146	*ARHGAP21*	Rho GTPase activating protein 21 [Source:HGNC Symbol;Acc:HGNC:23725]
**1,329652578**	4,38E-58	2,37E-56	*PAK3*	p21 (RAC1) activated kinase 3 [Source:HGNC Symbol;Acc:HGNC:8592]
**1,329329765**	7,51E-11	6,77E-10	*ALDH1A1*	aldehyde dehydrogenase 1 family member A1 [Source:HGNC Symbol;Acc:HGNC:402]
**1,326948378**	0,00082051	0,003169349	*CHST9*	carbohydrate sulfotransferase 9 [Source:HGNC Symbol;Acc:HGNC:19898]

**1,326726726**	0,000126783	0,000570184	*GRHL3*	grainyhead like transcription factor 3 [Source:HGNC Symbol;Acc:HGNC:25839]
**1,32412287**	8,05E-05	0,000374976	*ATP13A4*	ATPase 13A4 [Source:HGNC Symbol;Acc:HGNC:25422]
**1,322168728**	4,35E-11	4,02E-10	*PLXNC1*	plexin C1 [Source:HGNC Symbol;Acc:HGNC:9106]
**1,318806668**	4,24E-05	0,000207035	*SYTL1*	synaptotagmin like 1 [Source:HGNC Symbol;Acc:HGNC:15584]
**1,318167895**	4,52E-06	2,52E-05	*GDF10*	growth differentiation factor 10 [Source:HGNC Symbol;Acc:HGNC:4215]
**1,315990691**	1,57E-14	1,87E-13	*MYO5B*	myosin VB [Source:HGNC Symbol;Acc:HGNC:7603]
**1,315586242**	0,009239158	0,0277344	*NKX6-2*	NK6 homeobox 2 [Source:HGNC Symbol;Acc:HGNC:19321]
**1,315581919**	0,011533126	0,033676172	*HOXB8*	homeobox B8 [Source:HGNC Symbol;Acc:HGNC:5119]
**1,312657673**	6,53E-45	2,54E-43	*NDRG1*	N-myc downstream regulated 1 [Source:HGNC Symbol;Acc:HGNC:7679]
**1,310654043**	3,21E-19	5,01E-18	*CHST1*	carbohydrate sulfotransferase 1 [Source:HGNC Symbol;Acc:HGNC:1969]
**1,309767563**	1,54E-07	1,02E-06	*ACER2*	alkaline ceramidase 2 [Source:HGNC Symbol;Acc:HGNC:23675]
**1,309517474**	0,00196135	0,006963402	*LRRTM4*	leucine rich repeat transmembrane neuronal 4 [Source:HGNC Symbol;Acc:HGNC:19411]
**1,309373701**	2,38E-27	5,24E-26	*CREBRF*	CREB3 regulatory factor [Source:HGNC Symbol;Acc:HGNC:24050]
**1,30927508**	1,16E-09	9,43E-09	*DBP*	D-box binding PAR bZIP transcription factor [Source:HGNC Symbol;Acc:HGNC:2697]
**1,308107364**	7,73E-09	5,88E-08	*CACNA1E*	calcium voltage-gated channel subunit alpha1 E [Source:HGNC Symbol;Acc:HGNC:1392]
**1,307308605**	1,29E-57	6,93E-56	*LSAMP*	limbic system associated membrane protein [Source:HGNC Symbol;Acc:HGNC:6705]
**1,305429232**	3,25E-13	3,59E-12	*DNAH9*	dynein axonemal heavy chain 9 [Source:HGNC Symbol;Acc:HGNC:2953]
**1,303046694**	9,50E-37	2,94E-35	*SH3BP5*	SH3 domain binding protein 5 [Source:HGNC Symbol;Acc:HGNC:10827]
**1,302287131**	1,57E-68	1,05E-66	*SOCS6*	suppressor of cytokine signaling 6 [Source:HGNC Symbol;Acc:HGNC:16833]
**1,299889823**	1,25E-65	8,15E-64	*HES6*	hes family bHLH transcription factor 6 [Source:HGNC Symbol;Acc:HGNC:18254]
**1,29766008**	1,93E-55	9,84E-54	*CTNNBIP1*	catenin beta interacting protein 1 [Source:HGNC Symbol;Acc:HGNC:16913]
**1,297352146**	1,64E-07	1,09E-06	*NMNAT3*	nicotinamide nucleotide adenylyltransferase 3 [Source:HGNC Symbol;Acc:HGNC:20989]
**1,297239144**	0,013075572	0,037502889	*FGD3*	FYVE, RhoGEF and PH domain containing 3 [Source:HGNC Symbol;Acc:HGNC:16027]
**1,296920947**	5,06E-51	2,30E-49	*MXI1*	MAX interactor 1, dimerization protein [Source:HGNC Symbol;Acc:HGNC:7534]
**1,296096447**	2,10E-31	5,43E-30	*ENTPD2*	ectonucleoside triphosphate diphosphohydrolase 2 [Source:HGNC Symbol;Acc:HGNC:3364]
**1,295387942**	2,55E-16	3,38E-15	*TMEM35A*	transmembrane protein 35A [Source:HGNC Symbol;Acc:HGNC:25864]
**1,294155516**	0,000501096	0,002009977	*GJA3*	gap junction protein alpha 3 [Source:HGNC Symbol;Acc:HGNC:4277]
**1,294000624**	5,16E-06	2,85E-05	*RASEF*	RAS and EF-hand domain containing [Source:HGNC Symbol;Acc:HGNC:26464]
**1,292996161**	0,000488059	0,001963773	*MISP3*	MISP family member 3 [Source:HGNC Symbol;Acc:HGNC:26963]
**1,292448629**	5,24E-06	2,90E-05	*C15orf62*	chromosome 15 open reading frame 62 [Source:HGNC Symbol;Acc:HGNC:34489]
**1,290105432**	1,13E-07	7,64E-07	*RDH5*	retinol dehydrogenase 5 [Source:HGNC Symbol;Acc:HGNC:9940]
**1,288321652**	0,007619284	0,023405883	*SMPD3*	sphingomyelin phosphodiesterase 3 [Source:HGNC Symbol;Acc:HGNC:14240]
**1,288016933**	3,27E-62	1,98E-60	*RGMA*	repulsive guidance molecule BMP co-receptor a [Source:HGNC Symbol;Acc:HGNC:30308]

**1,28422181**	1,84E-17	2,63E-16	*SLC1A1*	solute carrier family 1 member 1 [Source:HGNC Symbol;Acc:HGNC:10939]
**1,282084481**	2,73E-29	6,49E-28	*SYTL5*	synaptotagmin like 5 [Source:HGNC Symbol;Acc:HGNC:15589]
**1,276296298**	0,001651129	0,00595462	*PGF*	placental growth factor [Source:HGNC Symbol;Acc:HGNC:8893]
**1,274672805**	9,16E-11	8,20E-10	*CASKIN1*	CASK interacting protein 1 [Source:HGNC Symbol;Acc:HGNC:20879]
**1,272399302**	1,73E-93	1,86E-91	*JMJD1C*	jumonji domain containing 1C [Source:HGNC Symbol;Acc:HGNC:12313]
**1,272015023**	1,75E-56	9,13E-55	*TNFRSF21*	TNF receptor superfamily member 21 [Source:HGNC Symbol;Acc:HGNC:13469]
**1,270707778**	4,21E-21	7,16E-20	*LRRTM2*	leucine rich repeat transmembrane neuronal 2 [Source:HGNC Symbol;Acc:HGNC:19409]
**1,270254724**	3,29E-97	3,80E-95	*ALDH3A2*	aldehyde dehydrogenase 3 family member A2 [Source:HGNC Symbol;Acc:HGNC:403]
**1,269682881**	0,003387487	0,011392994	*EPCAM*	epithelial cell adhesion molecule [Source:HGNC Symbol;Acc:HGNC:11529]
**1,266723378**	1,04E-07	7,10E-07	*CTTNBP2*	cortactin binding protein 2 [Source:HGNC Symbol;Acc:HGNC:15679]
**1,266692621**	0,000894748	0,003422049	*MCF2*	MCF,2 cell line derived transforming sequence [Source:HGNC Symbol;Acc:HGNC:6940]
**1,265719489**	2,96E-13	3,27E-12	*OGDHL*	oxoglutarate dehydrogenase like [Source:HGNC Symbol;Acc:HGNC:25590]
**1,265422118**	7,38E-31	1,87E-29	*TOX3*	TOX high mobility group box family member 3 [Source:HGNC Symbol;Acc:HGNC:11972]
**1,264817592**	4,68E-15	5,76E-14	*HGD*	homogentisate 1,2-dioxygenase [Source:HGNC Symbol;Acc:HGNC:4892]
**1,263297912**	1,69E-11	1,61E-10	*SELPLG*	selectin P ligand [Source:HGNC Symbol;Acc:HGNC:10722]
**1,263159636**	0,002434534	0,008480446	*KCNH3*	potassium voltage-gated channel subfamily H member 3 [Source:HGNC Symbol;Acc:HGNC:6252]
**1,261871838**	1,49E-167	4,66E-165	*TIMP1*	TIMP metallopeptidase inhibitor 1 [Source:HGNC Symbol;Acc:HGNC:11820]
**1,259252154**	1,43E-90	1,48E-88	*METTL7A*	methyltransferase like 7A [Source:HGNC Symbol;Acc:HGNC:24550]
**1,257068891**	3,44E-111	4,77E-109	*SPRY2*	sprouty RTK signaling antagonist 2 [Source:HGNC Symbol;Acc:HGNC:11270]
**1,252682836**	8,27E-08	5,69E-07	*RGR*	retinal G protein coupled receptor [Source:HGNC Symbol;Acc:HGNC:9990]
**1,252405072**	5,31E-95	5,82E-93	*DECR1*	2,4-dienoyl-CoA reductase 1 [Source:HGNC Symbol;Acc:HGNC:2753]
**1,251995377**	6,99E-86	6,62E-84	*AIF1L*	allograft inflammatory factor 1 like [Source:HGNC Symbol;Acc:HGNC:28904]
**1,251391535**	2,19E-14	2,60E-13	*RAB26*	RAB26, member RAS oncogene family [Source:HGNC Symbol;Acc:HGNC:14259]
**1,251093835**	4,57E-11	4,20E-10	*HIST1H2BG*	histone cluster 1 H2B family member g [Source:HGNC Symbol;Acc:HGNC:4746]
**1,250562286**	1,25E-48	5,38E-47	*ADGRL4*	adhesion G protein-coupled receptor L4 [Source:HGNC Symbol;Acc:HGNC:20822]
**1,248624048**	3,56E-136	7,20E-134	*CHST2*	carbohydrate sulfotransferase 2 [Source:HGNC Symbol;Acc:HGNC:1970]
**1,246266381**	5,58E-13	6,08E-12	*BMF*	Bcl2 modifying factor [Source:HGNC Symbol;Acc:HGNC:24132]
**1,245147428**	5,54E-15	6,77E-14	*ACSS1*	acyl-CoA synthetase short chain family member 1 [Source:HGNC Symbol;Acc:HGNC:16091]
**1,24467941**	2,67E-23	4,95E-22	*NTNG2*	netrin G2 [Source:HGNC Symbol;Acc:HGNC:14288]
**1,242172565**	0,010594849	0,031237259	*GAL*	galanin and GMAP prepropeptide [Source:HGNC Symbol;Acc:HGNC:4114]
**1,240067917**	1,09E-49	4,86E-48	*RTL5*	retrotransposon Gag like 5 [Source:HGNC Symbol;Acc:HGNC:29430]

**1,234173199**	2,14E-45	8,44E-44	*KIF13B*	kinesin family member 13B [Source:HGNC Symbol;Acc:HGNC:14405]
**1,233072538**	5,72E-08	4,00E-07	*GHR*	growth hormone receptor [Source:HGNC Symbol;Acc:HGNC:4263]
**1,232897907**	0,00043069	0,001750359	*GRASP*	general receptor for phosphoinositides 1 associated scaffold protein [Source:HGNC Symbol;Acc:HGNC:18707]
**1,232779446**	7,34E-20	1,17E-18	*ADCY1*	adenylate cyclase 1 [Source:HGNC Symbol;Acc:HGNC:232]
**1,231546815**	0,004310824	0,014147383	*IQCD*	IQ motif containing D [Source:HGNC Symbol;Acc:HGNC:25168]
**1,229874786**	4,59E-42	1,64E-40	*NT5DC1*	5'-nucleotidase domain containing 1 [Source:HGNC Symbol;Acc:HGNC:21556]
**1,222936486**	3,37E-09	2,65E-08	*NHSL2*	NHS like 2 [Source:HGNC Symbol;Acc:HGNC:33737]
**1,221249108**	3,75E-12	3,79E-11	*NCAM2*	neural cell adhesion molecule 2 [Source:HGNC Symbol;Acc:HGNC:7657]
**1,220990933**	3,08E-06	1,76E-05	*CCNA1*	cyclin A1 [Source:HGNC Symbol;Acc:HGNC:1577]
**1,217227512**	1,05E-07	7,12E-07	*GAD1*	glutamate decarboxylase 1 [Source:HGNC Symbol;Acc:HGNC:4092]
**1,215852973**	2,08E-38	6,73E-37	*ACSS2*	acyl-CoA synthetase short chain family member 2 [Source:HGNC Symbol;Acc:HGNC:15814]
**1,214670462**	0,009255022	0,027759926	*OSCAR*	osteoclast associated, immunoglobulin-like receptor [Source:HGNC Symbol;Acc:HGNC:29960]
**1,213793155**	2,03E-42	7,37E-41	*TIAM1*	T cell lymphoma invasion and metastasis 1 [Source:HGNC Symbol;Acc:HGNC:11805]
**1,207445145**	1,45E-14	1,73E-13	*STK32B*	serine/threonine kinase 32B [Source:HGNC Symbol;Acc:HGNC:14217]
**1,206049846**	2,59E-07	1,69E-06	*YPEL1*	yippee like 1 [Source:HGNC Symbol;Acc:HGNC:12845]
**1,20002522**	5,17E-06	2,86E-05	*FNDC5*	fibronectin type III domain containing 5 [Source:HGNC Symbol;Acc:HGNC:20240]
**1,198559831**	9,37E-32	2,45E-30	*GNG2*	G protein subunit gamma 2 [Source:HGNC Symbol;Acc:HGNC:4404]
**1,198160217**	0,004978426	0,016088481	*NDST3*	N-deacetylase and N-sulfotransferase 3 [Source:HGNC Symbol;Acc:HGNC:7682]
**1,197554909**	8,31E-07	5,10E-06	*SMIM10L2A*	small integral membrane protein 10 like 2A [Source:HGNC Symbol;Acc:HGNC:34499]
**1,197300531**	5,02E-33	1,37E-31	*YPEL3*	yippee like 3 [Source:HGNC Symbol;Acc:HGNC:18327]
**1,196039792**	1,03E-52	4,89E-51	*LRRC17*	leucine rich repeat containing 17 [Source:HGNC Symbol;Acc:HGNC:16895]
**1,194394533**	0,010190409	0,030195876	*AGTR1*	angiotensin II receptor type 1 [Source:HGNC Symbol;Acc:HGNC:336]
**1,192784567**	6,49E-65	4,18E-63	*POSTN*	periostin [Source:HGNC Symbol;Acc:HGNC:16953]
**1,191525689**	4,68E-16	6,08E-15	*PTPRN*	protein tyrosine phosphatase, receptor type N [Source:HGNC Symbol;Acc:HGNC:9676]
**1,190637256**	1,95E-19	3,08E-18	*EDNRA*	endothelin receptor type A [Source:HGNC Symbol;Acc:HGNC:3179]
**1,190315172**	6,66E-12	6,58E-11	*PDE3A*	phosphodiesterase 3A [Source:HGNC Symbol;Acc:HGNC:8778]
**1,189328016**	0,000109709	0,000498249	*ANKRD53*	ankyrin repeat domain 53 [Source:HGNC Symbol;Acc:HGNC:25691]
**1,188396454**	5,37E-11	4,91E-10	*RFTN2*	raftlin family member 2 [Source:HGNC Symbol;Acc:HGNC:26402]
**1,187504504**	1,22E-15	1,55E-14	*PCDH17*	protocadherin 17 [Source:HGNC Symbol;Acc:HGNC:14267]
**1,187223179**	9,07E-29	2,12E-27	*IL33*	interleukin 33 [Source:HGNC Symbol;Acc:HGNC:16028]
**1,18501184**	1,65E-19	2,61E-18	*RNF128*	ring finger protein 128, E3 ubiquitin protein ligase [Source:HGNC Symbol;Acc:HGNC:21153]
**1,177595958**	5,57E-05	0,000266519	*ANKRD36C*	ankyrin repeat domain 36C [Source:HGNC Symbol;Acc:HGNC:32946]
**1,177031117**	8,35E-18	1,22E-16	*ETV4*	ETS variant 4 [Source:HGNC Symbol;Acc:HGNC:3493]
**1,176865822**	3,39E-60	1,96E-58	*OAF*	out at first homolog [Source:HGNC Symbol;Acc:HGNC:28752]

**1,175753252**	7,98E-09	6,07E-08	*ZIC4*	Zic family member 4 [Source:HGNC Symbol;Acc:HGNC:20393]
**1,175273238**	4,09E-11	3,81E-10	*ARHGAP24*	Rho GTPase activating protein 24 [Source:HGNC Symbol;Acc:HGNC:25361]
**1,173530508**	2,46E-31	6,34E-30	*KIF26B*	kinesin family member 26B [Source:HGNC Symbol;Acc:HGNC:25484]
**1,173309918**	1,21E-06	7,28E-06	*PODN*	podocan [Source:HGNC Symbol;Acc:HGNC:23174]
**1,172636033**	4,78E-49	2,10E-47	*SASH1*	SAM and SH3 domain containing 1 [Source:HGNC Symbol;Acc:HGNC:19182]
**1,172362989**	5,66E-29	1,34E-27	*COQ8A*	coenzyme Q8A [Source:HGNC Symbol;Acc:HGNC:16812]
**1,170684323**	0,003628177	0,012118485	*PTGER1*	prostaglandin E receptor 1 [Source:HGNC Symbol;Acc:HGNC:9593]
**1,169095674**	3,86E-10	3,30E-09	*AK5*	adenylate kinase 5 [Source:HGNC Symbol;Acc:HGNC:365]
**1,168530829**	8,01E-153	2,07E-150	*SMS*	spermine synthase [Source:HGNC Symbol;Acc:HGNC:11123]
**1,166412125**	7,11E-07	4,40E-06	*ICA1*	islet cell autoantigen 1 [Source:HGNC Symbol;Acc:HGNC:5343]
**1,164265203**	9,27E-15	1,12E-13	*SHROOM2*	shroom family member 2 [Source:HGNC Symbol;Acc:HGNC:630]
**1,163192405**	0,005036993	0,01625339	*EGR3*	early growth response 3 [Source:HGNC Symbol;Acc:HGNC:3240]
**1,162876507**	7,48E-07	4,62E-06	*ITGA8*	integrin subunit alpha 8 [Source:HGNC Symbol;Acc:HGNC:6144]
**1,162345016**	1,22E-07	8,21E-07	*ABCA5*	ATP binding cassette subfamily A member 5 [Source:HGNC Symbol;Acc:HGNC:35]
**1,160359112**	3,62E-26	7,64E-25	*PTCH1*	patched 1 [Source:HGNC Symbol;Acc:HGNC:9585]
**1,159522838**	1,31E-12	1,38E-11	*XKR4*	XK related 4 [Source:HGNC Symbol;Acc:HGNC:29394]
**1,158188867**	6,84E-90	6,87E-88	*CAMK2N1*	calcium/calmodulin dependent protein kinase II inhibitor 1 [Source:HGNC Symbol;Acc:HGNC:24190]
**1,157827631**	3,01E-11	2,83E-10	*TMC7*	transmembrane channel like 7 [Source:HGNC Symbol;Acc:HGNC:23000]
**1,156887334**	2,36E-08	1,72E-07	*SELENOP*	selenoprotein P [Source:HGNC Symbol;Acc:HGNC:10751]
**1,152674789**	3,39E-29	8,06E-28	*ABHD6*	abhydrolase domain containing 6 [Source:HGNC Symbol;Acc:HGNC:21398]
**1,151974172**	3,23E-118	4,89E-116	*TCF12*	transcription factor 12 [Source:HGNC Symbol;Acc:HGNC:11623]
**1,151681166**	0,014854253	0,041834213	*AQP2*	aquaporin 2 [Source:HGNC Symbol;Acc:HGNC:634]
**1,151258584**	0,001224063	0,004541037	*AZGP1*	alpha-2-glycoprotein 1, zinc-binding [Source:HGNC Symbol;Acc:HGNC:910]
**1,149904809**	1,42E-08	1,06E-07	*ANKRD35*	ankyrin repeat domain 35 [Source:HGNC Symbol;Acc:HGNC:26323]
**1,149687398**	2,75E-59	1,55E-57	*DTNA*	dystrobrevin alpha [Source:HGNC Symbol;Acc:HGNC:3057]
**1,149227224**	0,003645368	0,012167529	*DLX1*	distal-less homeobox 1 [Source:HGNC Symbol;Acc:HGNC:2914]
**1,149009742**	5,49E-09	4,23E-08	*MYCL*	MYCL proto-oncogene, bHLH transcription factor [Source:HGNC Symbol;Acc:HGNC:7555]
**1,148547823**	1,31E-26	2,82E-25	*SP8*	Sp8 transcription factor [Source:HGNC Symbol;Acc:HGNC:19196]
**1,148303519**	4,43E-05	0,000215272	*CADM2*	cell adhesion molecule 2 [Source:HGNC Symbol;Acc:HGNC:29849]
**1,146247723**	3,98E-14	4,66E-13	*PCDHGC4*	protocadherin gamma subfamily C, 4 [Source:HGNC Symbol;Acc:HGNC:8717]
**1,14557331**	0,011187259	0,032794554	*INO80B*	INO80 complex subunit B [Source:HGNC Symbol;Acc:HGNC:13324]
**1,14463039**	5,98E-32	1,58E-30	*SMIM3*	small integral membrane protein 3 [Source:HGNC Symbol;Acc:HGNC:30248]
**1,144252955**	0,014461079	0,04089053	*ETV2*	ETS variant 2 [Source:HGNC Symbol;Acc:HGNC:3491]
**1,143565666**	2,08E-07	1,37E-06	*HIST1H2BC*	histone cluster 1 H2B family member c [Source:HGNC Symbol;Acc:HGNC:4757]

**1,142796122**	3,48E-25	7,01E-24	*SLC1A2*	solute carrier family 1 member 2 [Source:HGNC Symbol;Acc:HGNC:10940]
**1,142401962**	4,09E-19	6,35E-18	*PLCL2*	phospholipase C like 2 [Source:HGNC Symbol;Acc:HGNC:9064]
**1,141029288**	7,34E-25	1,46E-23	*MEGF10*	multiple EGF like domains 10 [Source:HGNC Symbol;Acc:HGNC:29634]
**1,139966397**	1,77E-09	1,42E-08	*ADAM22*	ADAM metallopeptidase domain 22 [Source:HGNC Symbol;Acc:HGNC:201]
**1,139815633**	1,37E-05	7,14E-05	*C2orf92*	chromosome 2 open reading frame 92 [Source:HGNC Symbol;Acc:HGNC:49272]
**1,138834779**	3,90E-55	1,96E-53	*FDXR*	ferredoxin reductase [Source:HGNC Symbol;Acc:HGNC:3642]
**1,137560137**	0,011648939	0,033978553	*LDHD*	lactate dehydrogenase D [Source:HGNC Symbol;Acc:HGNC:19708]
**1,137456229**	6,04E-127	1,04E-124	*G6PD*	glucose-6-phosphate dehydrogenase [Source:HGNC Symbol;Acc:HGNC:4057]
**1,134622126**	0,000225473	0,000962077	*DLG2*	discs large MAGUK scaffold protein 2 [Source:HGNC Symbol;Acc:HGNC:2901]
**1,13119947**	3,31E-05	0,000163932	*SLC16A14*	solute carrier family 16 member 14 [Source:HGNC Symbol;Acc:HGNC:26417]
**1,12904793**	0,000949081	0,003605691	*TGFBR3*	transforming growth factor beta receptor 3 [Source:HGNC Symbol;Acc:HGNC:11774]
**1,128550904**	0,017683012	0,04868159	*HIST1H2AI*	histone cluster 1 H2A family member i [Source:HGNC Symbol;Acc:HGNC:4725]
**1,128493302**	3,24E-07	2,08E-06	*DUSP15*	dual specificity phosphatase 15 [Source:HGNC Symbol;Acc:HGNC:16236]
**1,127670281**	1,96E-07	1,29E-06	*PTPRH*	protein tyrosine phosphatase, receptor type H [Source:HGNC Symbol;Acc:HGNC:9672]
**1,126491164**	1,16E-32	3,15E-31	*CNTNAP2*	contactin associated protein like 2 [Source:HGNC Symbol;Acc:HGNC:13830]
**1,121062122**	0,000448426	0,001816355	*TSPAN7*	tetraspanin 7 [Source:HGNC Symbol;Acc:HGNC:11854]
**1,114906403**	0,001656226	0,005969672	*ZSWIM5*	zinc finger SWIM-type containing 5 [Source:HGNC Symbol;Acc:HGNC:29299]
**1,113970219**	2,36E-111	3,32E-109	*CORO2B*	coronin 2B [Source:HGNC Symbol;Acc:HGNC:2256]
**1,111435908**	2,66E-100	3,21E-98	*EGFR*	epidermal growth factor receptor [Source:HGNC Symbol;Acc:HGNC:3236]
**1,111039792**	1,91E-28	4,40E-27	*RIN1*	Ras and Rab interactor 1 [Source:HGNC Symbol;Acc:HGNC:18749]
**1,110161171**	3,72E-08	2,65E-07	*PPP1R14C*	protein phosphatase 1 regulatory inhibitor subunit 14C [Source:HGNC Symbol;Acc:HGNC:14952]
**1,105956128**	3,45E-32	9,19E-31	*PIK3IP1*	phosphoinositide-3-kinase interacting protein 1 [Source:HGNC Symbol;Acc:HGNC:24942]
**1,105375418**	0,003205512	0,010841087	*ITPKA*	inositol-trisphosphate 3-kinase A [Source:HGNC Symbol;Acc:HGNC:6178]
**1,104179636**	1,42E-09	1,15E-08	*FEZF2*	FEZ family zinc finger 2 [Source:HGNC Symbol;Acc:HGNC:13506]
**1,101124162**	0,002737734	0,009408264	*LCN12*	lipocalin 12 [Source:HGNC Symbol;Acc:HGNC:28733]
**1,100766699**	0,001542299	0,005604842	*SLC13A4*	solute carrier family 13 member 4 [Source:HGNC Symbol;Acc:HGNC:15827]
**1,099564057**	0,011238416	0,032908225	*RAB33A*	RAB33A, member RAS oncogene family [Source:HGNC Symbol;Acc:HGNC:9773]
**1,09895401**	4,44E-166	1,32E-163	*FTL*	ferritin light chain [Source:HGNC Symbol;Acc:HGNC:3999]
**1,097428068**	2,90E-20	4,72E-19	*ORAI3*	ORAI calcium release-activated calcium modulator 3 [Source:HGNC Symbol;Acc:HGNC:28185]
**1,095963003**	2,04E-14	2,42E-13	*ST3GAL5*	ST3 beta-galactoside alpha-2,3-sialyltransferase 5 [Source:HGNC Symbol;Acc:HGNC:10872]
**1,094891295**	2,32E-21	3,99E-20	*RETREG1*	reticulophagy regulator 1 [Source:HGNC Symbol;Acc:HGNC:25964]
**1,091093159**	8,50E-54	4,12E-52	*TM7SF2*	transmembrane 7 superfamily member 2 [Source:HGNC Symbol;Acc:HGNC:11863]
**1,091023375**	3,28E-07	2,10E-06	*GGN*	gametogenetin [Source:HGNC Symbol;Acc:HGNC:18869]

**1,089323542**	1,17E-14	1,41E-13	*STK32A*	serine/threonine kinase 32A [Source:HGNC Symbol;Acc:HGNC:28317]
**1,088029839**	0,006544519	0,020540667	*CCDC30*	coiled-coil domain containing 30 [Source:HGNC Symbol;Acc:HGNC:26103]
**1,086591765**	0,002050976	0,007256381	*SMIM1*	small integral membrane protein 1 (Vel blood group) [Source:HGNC Symbol;Acc:HGNC:44204]
**1,084565878**	9,28E-06	4,96E-05	*TENM1*	teneurin transmembrane protein 1 [Source:HGNC Symbol;Acc:HGNC:8117]
**1,083975814**	0,012208992	0,035388751	*UNC79*	unc-79 homolog, NALCN channel complex subunit [Source:HGNC Symbol;Acc:HGNC:19966]
**1,083966179**	1,02E-21	1,78E-20	*NBPF14*	NBPF member 14 [Source:HGNC Symbol;Acc:HGNC:25232]
**1,083933343**	2,20E-123	3,59E-121	*TRIB2*	tribbles pseudokinase 2 [Source:HGNC Symbol;Acc:HGNC:30809]
**1,081377706**	2,68E-36	8,19E-35	*GPR153*	G protein-coupled receptor 153 [Source:HGNC Symbol;Acc:HGNC:23618]
**1,080765115**	2,54E-16	3,36E-15	*HIST1H3E*	histone cluster 1 H3 family member e [Source:HGNC Symbol;Acc:HGNC:4769]
**1,080086821**	0,00064224	0,002534738	*FAM222A*	family with sequence similarity 222 member A [Source:HGNC Symbol;Acc:HGNC:25915]
**1,079663456**	6,52E-14	7,51E-13	*KCNE4*	potassium voltage-gated channel subfamily E regulatory subunit 4 [Source:HGNC Symbol;Acc:HGNC:6244]
**1,078799022**	9,64E-76	7,28E-74	*EFHD2*	EF-hand domain family member D2 [Source:HGNC Symbol;Acc:HGNC:28670]
**1,078738696**	2,68E-06	1,54E-05	*CES4A*	carboxylesterase 4A [Source:HGNC Symbol;Acc:HGNC:26741]
**1,077883593**	3,19E-12	3,26E-11	*CECR2*	CECR2, histone acetyl-lysine reader [Source:HGNC Symbol;Acc:HGNC:1840]
**1,07767622**	5,20E-05	0,000250485	*NBPF10*	NBPF member 10 [Source:HGNC Symbol;Acc:HGNC:31992]
**1,076931174**	6,35E-43	2,35E-41	*VWA5A*	von Willebrand factor A domain containing 5A [Source:HGNC Symbol;Acc:HGNC:6658]
**1,076754097**	4,48E-11	4,13E-10	*CDH5*	cadherin 5 [Source:HGNC Symbol;Acc:HGNC:1764]
**1,076228632**	5,44E-13	5,94E-12	*GSAP*	gamma-secretase activating protein [Source:HGNC Symbol;Acc:HGNC:28042]
**1,075092654**	2,55E-12	2,63E-11	*MYO15B*	myosin XVB [Source:HGNC Symbol;Acc:HGNC:14083]
**1,074459158**	1,36E-21	2,35E-20	*MINDY1*	MINDY lysine 48 deubiquitinase 1 [Source:HGNC Symbol;Acc:HGNC:25648]
**1,072851107**	0,000163902	0,000720263	*PLLP*	plasmolipin [Source:HGNC Symbol;Acc:HGNC:18553]
**1,071912588**	9,94E-121	1,54E-118	*GRB10*	growth factor receptor bound protein 10 [Source:HGNC Symbol;Acc:HGNC:4564]
**1,069752128**	1,71E-09	1,37E-08	*PPM1H*	protein phosphatase, Mg2+/Mn2+ dependent 1H [Source:HGNC Symbol;Acc:HGNC:18583]
**1,069146225**	1,00E-71	7,22E-70	*STEAP3*	STEAP3 metalloreductase [Source:HGNC Symbol;Acc:HGNC:24592]
**1,069141394**	2,76E-38	8,91E-37	*SLC2A12*	solute carrier family 2 member 12 [Source:HGNC Symbol;Acc:HGNC:18067]
**1,068510434**	4,45E-39	1,46E-37	*TPCN1*	two pore segment channel 1 [Source:HGNC Symbol;Acc:HGNC:18182]
**1,066849845**	3,20E-17	4,50E-16	*STX1A*	syntaxin 1A [Source:HGNC Symbol;Acc:HGNC:11433]
**1,065353572**	1,93E-25	3,94E-24	*DKK1*	dickkopf WNT signaling pathway inhibitor 1 [Source:HGNC Symbol;Acc:HGNC:2891]
**1,064617475**	0,003517423	0,011783035	*AGAP2*	ArfGAP with GTPase domain, ankyrin repeat and PH domain 2 [Source:HGNC Symbol;Acc:HGNC:16921]
**1,062721696**	9,29E-10	7,66E-09	*GJB2*	gap junction protein beta 2 [Source:HGNC Symbol;Acc:HGNC:4284]
**1,061543107**	1,06E-38	3,45E-37	*KLHL24*	kelch like family member 24 [Source:HGNC Symbol;Acc:HGNC:25947]
**1,060891932**	1,17E-91	1,22E-89	*LGALS3*	galectin 3 [Source:HGNC Symbol;Acc:HGNC:6563]
**1,06078305**	3,34E-06	1,89E-05	*SERPINI1*	serpin family I member 1 [Source:HGNC Symbol;Acc:HGNC:8943]

**1,059334687**	3,75E-17	5,24E-16	*GRIN2A*	glutamate ionotropic receptor NMDA type subunit 2A [Source:HGNC Symbol;Acc:HGNC:4585]
**1,058816088**	4,49E-52	2,08E-50	*RTN1*	reticulon 1 [Source:HGNC Symbol;Acc:HGNC:10467]
**1,058018902**	4,56E-09	3,54E-08	*INSYN2*	inhibitory synaptic factor 2A [Source:HGNC Symbol;Acc:HGNC:33859]
**1,057459157**	2,26E-25	4,60E-24	*CSMD2*	CUB and Sushi multiple domains 2 [Source:HGNC Symbol;Acc:HGNC:19290]
**1,05456154**	1,64E-61	9,82E-60	*MYORG*	myogenesis regulating glycosidase (putative) [Source:HGNC Symbol;Acc:HGNC:19918]
**1,053569525**	0,000208735	0,000896772	*BHMT2*	betaine--homocysteine S-methyltransferase 2 [Source:HGNC Symbol;Acc:HGNC:1048]
**1,052665662**	2,94E-87	2,85E-85	*SEMA6A*	semaphorin 6A [Source:HGNC Symbol;Acc:HGNC:10738]
**1,052389029**	1,32E-96	1,50E-94	*TMEM158*	transmembrane protein 158 (gene/pseudogene) [Source:HGNC Symbol;Acc:HGNC:30293]
**1,052077717**	0,000283469	0,001187609	*HEXIM2*	hexamethylene bisacetamide inducible 2 [Source:HGNC Symbol;Acc:HGNC:28591]
**1,050927162**	1,53E-16	2,05E-15	*NRN1*	neuritin 1 [Source:HGNC Symbol;Acc:HGNC:17972]
**1,050903392**	2,92E-16	3,85E-15	*ABCB9*	ATP binding cassette subfamily B member 9 [Source:HGNC Symbol;Acc:HGNC:50]
**1,049219916**	3,69E-19	5,74E-18	*ZNF521*	zinc finger protein 521 [Source:HGNC Symbol;Acc:HGNC:24605]
**1,048232034**	6,92E-09	5,29E-08	*DISP1*	dispatched RND transporter family member 1 [Source:HGNC Symbol;Acc:HGNC:19711]
**1,047619486**	0,014455782	0,040887465	*PRRT1*	proline rich transmembrane protein 1 [Source:HGNC Symbol;Acc:HGNC:13943]
**1,046786418**	3,61E-12	3,65E-11	*TLE2*	transducin like enhancer of split 2 [Source:HGNC Symbol;Acc:HGNC:11838]
**1,046523025**	1,20E-49	5,33E-48	*TSPAN13*	tetraspanin 13 [Source:HGNC Symbol;Acc:HGNC:21643]
**1,04437855**	8,54E-93	9,11E-91	*SQSTM1*	sequestosome 1 [Source:HGNC Symbol;Acc:HGNC:11280]
**1,04327586**	1,72E-17	2,47E-16	*EPHB3*	EPH receptor B3 [Source:HGNC Symbol;Acc:HGNC:3394]
**1,042477509**	6,84E-26	1,43E-24	*PPL*	periplakin [Source:HGNC Symbol;Acc:HGNC:9273]
**1,040064941**	6,36E-05	0,000301685	*PLA2G4A*	phospholipase A2 group IVA [Source:HGNC Symbol;Acc:HGNC:9035]
**1,03990424**	4,03E-15	4,97E-14	*ADCY2*	adenylate cyclase 2 [Source:HGNC Symbol;Acc:HGNC:233]
**1,038804904**	5,73E-108	7,52E-106	*ARL4C*	ADP ribosylation factor like GTPase 4C [Source:HGNC Symbol;Acc:HGNC:698]
**1,038776882**	0,009491086	0,028379662	*WDR88*	WD repeat domain 88 [Source:HGNC Symbol;Acc:HGNC:26999]
**1,038745423**	9,81E-11	8,74E-10	*PHF21B*	PHD finger protein 21B [Source:HGNC Symbol;Acc:HGNC:25161]
**1,037373659**	3,62E-60	2,08E-58	*NETO2*	neuropilin and tolloid like 2 [Source:HGNC Symbol;Acc:HGNC:14644]
**1,036707692**	4,40E-19	6,81E-18	*RNF144A*	ring finger protein 144A [Source:HGNC Symbol;Acc:HGNC:20457]
**1,034563565**	4,04E-128	7,25E-126	*PYGB*	glycogen phosphorylase B [Source:HGNC Symbol;Acc:HGNC:9723]
**1,034258947**	1,42E-06	8,48E-06	*RASSF9*	Ras association domain family member 9 [Source:HGNC Symbol;Acc:HGNC:15739]
**1,034179164**	0,016406269	0,045687354	*MUM1L1*	MUM1 like 1 [Source:HGNC Symbol;Acc:HGNC:26583]
**1,034157123**	5,26E-06	2,91E-05	*CD38*	CD38 molecule [Source:HGNC Symbol;Acc:HGNC:1667]
**1,033413719**	1,69E-07	1,12E-06	*SRCIN1*	SRC kinase signaling inhibitor 1 [Source:HGNC Symbol;Acc:HGNC:29506]
**1,032848983**	0,001158362	0,004320425	*TMEM119*	transmembrane protein 119 [Source:HGNC Symbol;Acc:HGNC:27884]
**1,03165744**	0,004870098	0,015769917	*IMPG2*	interphotoreceptor matrix proteoglycan 2 [Source:HGNC Symbol;Acc:HGNC:18362]
**1,026601214**	8,04E-06	4,34E-05	*SLC24A3*	solute carrier family 24 member 3 [Source:HGNC Symbol;Acc:HGNC:10977]
**1,025758989**	3,94E-06	2,21E-05	*ZDHHC23*	zinc finger DHHC-type containing 23 [Source:HGNC Symbol;Acc:HGNC:28654]

**1,0251778**	1,69E-05	8,70E-05	*BEAN1*	brain expressed associated with NEDD4 1 [Source:HGNC Symbol;Acc:HGNC:24160]
**1,022326966**	4,12E-10	3,51E-09	*MAML3*	mastermind like transcriptional coactivator 3 [Source:HGNC Symbol;Acc:HGNC:16272]
**1,022013314**	1,60E-11	1,54E-10	*MANSC1*	MANSC domain containing 1 [Source:HGNC Symbol;Acc:HGNC:25505]
**1,021734134**	0,000539701	0,002152815	*GLUD2*	glutamate dehydrogenase 2 [Source:HGNC Symbol;Acc:HGNC:4336]
**1,021110866**	0,000330857	0,00137017	*SNAP91*	synaptosome associated protein 91 [Source:HGNC Symbol;Acc:HGNC:14986]
**1,02058436**	3,59E-71	2,56E-69	*ATP1B3*	ATPase Na+/K+ transporting subunit beta 3 [Source:HGNC Symbol;Acc:HGNC:806]
**1,019989404**	0,01664779	0,046258164	*FERMT3*	fermitin family member 3 [Source:HGNC Symbol;Acc:HGNC:23151]
**1,018692981**	1,01E-12	1,08E-11	*MTSS1*	MTSS1, I-BAR domain containing [Source:HGNC Symbol;Acc:HGNC:20443]
**1,017564513**	6,82E-09	5,22E-08	*TUBB4A*	tubulin beta 4A class IVa [Source:HGNC Symbol;Acc:HGNC:20774]
**1,016499087**	2,11E-26	4,48E-25	*ITGA10*	integrin subunit alpha 10 [Source:HGNC Symbol;Acc:HGNC:6135]
**1,01632038**	0,004269402	0,014021325	*POU3F1*	POU class 3 homeobox 1 [Source:HGNC Symbol;Acc:HGNC:9214]
**1,016094341**	1,66E-14	1,98E-13	*STON1*	stonin 1 [Source:HGNC Symbol;Acc:HGNC:17003]
**1,015357411**	6,73E-17	9,24E-16	*PIR*	pirin [Source:HGNC Symbol;Acc:HGNC:30048]
**1,01392513**	5,78E-33	1,58E-31	*HBP1*	HMG-box transcription factor 1 [Source:HGNC Symbol;Acc:HGNC:23200]
**1,013724983**	4,16E-06	2,33E-05	*KBTBD3*	kelch repeat and BTB domain containing 3 [Source:HGNC Symbol;Acc:HGNC:22934]
**1,013223588**	9,13E-26	1,90E-24	*SORL1*	sortilin related receptor 1 [Source:HGNC Symbol;Acc:HGNC:11185]
**1,012667835**	4,57E-11	4,20E-10	*LGALS9*	galectin 9 [Source:HGNC Symbol;Acc:HGNC:6570]
**1,012311809**	5,45E-20	8,79E-19	*PPP1R3B*	protein phosphatase 1 regulatory subunit 3B [Source:HGNC Symbol;Acc:HGNC:14942]
**1,011240609**	0,012304181	0,035606165	*FAM155B*	family with sequence similarity 155 member B [Source:HGNC Symbol;Acc:HGNC:30701]
**1,010551856**	1,31E-13	1,49E-12	*FAM117B*	family with sequence similarity 117 member B [Source:HGNC Symbol;Acc:HGNC:14440]
**1,009738057**	0,002772735	0,009513382	*ADAM11*	ADAM metallopeptidase domain 11 [Source:HGNC Symbol;Acc:HGNC:189]
**1,007362493**	6,11E-92	6,40E-90	*ITGA7*	integrin subunit alpha 7 [Source:HGNC Symbol;Acc:HGNC:6143]
**1,007062067**	0,001055385	0,003970686	*KLF4*	Kruppel like factor 4 [Source:HGNC Symbol;Acc:HGNC:6348]
**1,00540237**	1,62E-56	8,51E-55	*FEM1C*	fem-1 homolog C [Source:HGNC Symbol;Acc:HGNC:16933]
**1,005375529**	1,14E-77	9,05E-76	*BTG2*	BTG anti-proliferation factor 2 [Source:HGNC Symbol;Acc:HGNC:1131]
**1,004371305**	0,003441434	0,011550409	*TM6SF1*	transmembrane 6 superfamily member 1 [Source:HGNC Symbol;Acc:HGNC:11860]
**1,003741632**	1,72E-21	2,96E-20	*GABBR2*	gamma-aminobutyric acid type B receptor subunit 2 [Source:HGNC Symbol;Acc:HGNC:4507]
**1,002522162**	9,46E-06	5,05E-05	*ANXA9*	annexin A9 [Source:HGNC Symbol;Acc:HGNC:547]
**1,002212511**	0,013875064	0,039509829	*RAB37*	RAB37, member RAS oncogene family [Source:HGNC Symbol;Acc:HGNC:30268]
**1,000919706**	3,22E-06	1,83E-05	*ARHGAP27*	Rho GTPase activating protein 27 [Source:HGNC Symbol;Acc:HGNC:31813]
**1,000220682**	3,65E-05	0,000179349	*RASSF5*	Ras association domain family member 5 [Source:HGNC Symbol;Acc:HGNC:17609]
**0,999840735**	7,29E-25	1,45E-23	*OSTF1*	osteoclast stimulating factor 1 [Source:HGNC Symbol;Acc:HGNC:8510]
**0,99983964**	0,005055799	0,016308649	*NOTCH2NLA*	notch 2 N-terminal like A [Source:HGNC Symbol;Acc:HGNC:31862]

**0,99971526**	5,88E-05	0,000280319	*SARDH*	sarcosine dehydrogenase [Source:HGNC Symbol;Acc:HGNC:10536]
**0,999280114**	0,003566675	0,011931545	*CYP4X1*	cytochrome P450 family 4 subfamily X member 1 [Source:HGNC Symbol;Acc:HGNC:20244]
**0,997335038**	0,000850975	0,003275943	*PNMA6A*	PNMA family member 6A [Source:HGNC Symbol;Acc:HGNC:28248]
**0,996764522**	4,25E-25	8,56E-24	*CREB5*	cAMP responsive element binding protein 5 [Source:HGNC Symbol;Acc:HGNC:16844]
**0,995745544**	3,61E-08	2,58E-07	*TNFRSF14*	TNF receptor superfamily member 14 [Source:HGNC Symbol;Acc:HGNC:11912]
**0,995248391**	0,011513746	0,033634767	*INSC*	INSC, spindle orientation adaptor protein [Source:HGNC Symbol;Acc:HGNC:33116]
**0,994037973**	0,007547416	0,023215273	*SNCG*	synuclein gamma [Source:HGNC Symbol;Acc:HGNC:11141]
**0,993893049**	0,000235831	0,001003406	*GCNA*	germ cell nuclear acidic peptidase [Source:HGNC Symbol;Acc:HGNC:15805]
**0,993490423**	1,15E-16	1,56E-15	*GALNT6*	polypeptide N-acetylgalactosaminyltransferase 6 [Source:HGNC Symbol;Acc:HGNC:4128]
**0,993391489**	2,19E-14	2,60E-13	*KCNAB2*	potassium voltage-gated channel subfamily A regulatory beta subunit 2 [Source:HGNC Symbol;Acc:HGNC:6229]
**0,991901529**	2,09E-85	1,95E-83	*TSKU*	tsukushi, small leucine rich proteoglycan [Source:HGNC Symbol;Acc:HGNC:28850]
**0,991774152**	0,016868897	0,046772085	*RUNDC3A*	RUN domain containing 3A [Source:HGNC Symbol;Acc:HGNC:16984]
**0,987309162**	0,000119848	0,000541003	*NCALD*	neurocalcin delta [Source:HGNC Symbol;Acc:HGNC:7655]
**0,985456865**	6,60E-61	3,88E-59	*SOX21*	SRY-box 21 [Source:HGNC Symbol;Acc:HGNC:11197]
**0,985253728**	0,004716211	0,015327882	*EXOC3L1*	exocyst complex component 3 like 1 [Source:HGNC Symbol;Acc:HGNC:27540]
**0,98493856**	0,017753196	0,048850118	*SNORC*	secondary ossification center associated regulator of chondrocyte maturation [Source:HGNC Symbol;Acc:HGNC:33763]
**0,984780496**	2,37E-11	2,24E-10	*TBC1D8*	TBC1 domain family member 8 [Source:HGNC Symbol;Acc:HGNC:17791]
**0,984422097**	0,000816308	0,003155005	*AC007906,2*	p53-regulated lncRNA 1 [Source:NCBI gene;Acc:105371267]
**0,984049471**	6,29E-05	0,000298299	*TMEM37*	transmembrane protein 37 [Source:HGNC Symbol;Acc:HGNC:18216]
**0,982754933**	8,18E-15	9,93E-14	*RNF112*	ring finger protein 112 [Source:HGNC Symbol;Acc:HGNC:12968]
**0,980278619**	0,011765583	0,034251861	*WAS*	Wiskott-Aldrich syndrome [Source:HGNC Symbol;Acc:HGNC:12731]
**0,979899144**	1,83E-13	2,05E-12	*ATP2B2*	ATPase plasma membrane Ca2+ transporting 2 [Source:HGNC Symbol;Acc:HGNC:815]
**0,979666834**	2,69E-11	2,53E-10	*SCX*	scleraxis bHLH transcription factor [Source:HGNC Symbol;Acc:HGNC:32322]
**0,978786201**	1,03E-75	7,72E-74	*PCDHGC3*	protocadherin gamma subfamily C, 3 [Source:HGNC Symbol;Acc:HGNC:8716]
**0,978096068**	0,002529499	0,008762487	*LAT2*	linker for activation of T cells family member 2 [Source:HGNC Symbol;Acc:HGNC:12749]
**0,977960414**	1,12E-15	1,43E-14	*PLEKHF1*	pleckstrin homology and FYVE domain containing 1 [Source:HGNC Symbol;Acc:HGNC:20764]
**0,975455768**	4,75E-38	1,52E-36	*UBALD2*	UBA like domain containing 2 [Source:HGNC Symbol;Acc:HGNC:28438]
**0,975010194**	1,09E-11	1,06E-10	*HIST3H2A*	histone cluster 3 H2A [Source:HGNC Symbol;Acc:HGNC:20507]
**0,974762946**	6,22E-07	3,87E-06	*GPER1*	G protein-coupled estrogen receptor 1 [Source:HGNC Symbol;Acc:HGNC:4485]
**0,974343633**	2,51E-05	0,000126612	*KLLN*	killin, p53 regulated DNA replication inhibitor [Source:HGNC Symbol;Acc:HGNC:37212]
**0,972260497**	9,27E-05	0,000427743	*NFATC2*	nuclear factor of activated T cells 2 [Source:HGNC Symbol;Acc:HGNC:7776]

**0,972032265**	4,20E-42	1,51E-40	*SLC7A11*	solute carrier family 7 member 11 [Source:HGNC Symbol;Acc:HGNC:11059]
**0,971951669**	1,07E-43	4,02E-42	*ELOVL6*	ELOVL fatty acid elongase 6 [Source:HGNC Symbol;Acc:HGNC:15829]
**0,969400744**	0,008721725	0,026367625	*GSTM5*	glutathione S-transferase mu 5 [Source:HGNC Symbol;Acc:HGNC:4637]
**0,969318724**	5,42E-49	2,37E-47	*GDF11*	growth differentiation factor 11 [Source:HGNC Symbol;Acc:HGNC:4216]
**0,968810294**	1,55E-07	1,04E-06	*MAPK10*	mitogen-activated protein kinase 10 [Source:HGNC Symbol;Acc:HGNC:6872]
**0,967046656**	4,02E-40	1,36E-38	*TXNIP*	thioredoxin interacting protein [Source:HGNC Symbol;Acc:HGNC:16952]
**0,966465916**	0,001297269	0,004787906	*DCAF12L2*	DDB1 and CUL4 associated factor 12 like 2 [Source:HGNC Symbol;Acc:HGNC:32950]
**0,966226445**	5,38E-27	1,17E-25	*GRK5*	G protein-coupled receptor kinase 5 [Source:HGNC Symbol;Acc:HGNC:4544]
**0,964354692**	0,004847054	0,015701754	*LBHD1*	LBH domain containing 1 [Source:HGNC Symbol;Acc:HGNC:28351]
**0,964107716**	4,30E-12	4,33E-11	*ARHGAP26*	Rho GTPase activating protein 26 [Source:HGNC Symbol;Acc:HGNC:17073]
**0,961137039**	2,22E-27	4,90E-26	*KLHL4*	kelch like family member 4 [Source:HGNC Symbol;Acc:HGNC:6355]
**0,958636988**	5,15E-57	2,74E-55	*PTPRZ1*	protein tyrosine phosphatase, receptor type Z1 [Source:HGNC Symbol;Acc:HGNC:9685]
**0,958326398**	3,48E-09	2,73E-08	*ACOX2*	acyl-CoA oxidase 2 [Source:HGNC Symbol;Acc:HGNC:120]
**0,955963594**	2,31E-05	0,000116786	*FOXS1*	forkhead box S1 [Source:HGNC Symbol;Acc:HGNC:3735]
**0,955135187**	7,44E-06	4,04E-05	*RGS6*	regulator of G protein signaling 6 [Source:HGNC Symbol;Acc:HGNC:10002]
**0,954394718**	3,54E-35	1,04E-33	*ZNF395*	zinc finger protein 395 [Source:HGNC Symbol;Acc:HGNC:18737]
**0,954081404**	1,04E-35	3,08E-34	*SLC6A6*	solute carrier family 6 member 6 [Source:HGNC Symbol;Acc:HGNC:11052]
**0,952270955**	1,65E-33	4,57E-32	*ARRDC3*	arrestin domain containing 3 [Source:HGNC Symbol;Acc:HGNC:29263]
**0,951955159**	3,51E-36	1,06E-34	*NAV2*	neuron navigator 2 [Source:HGNC Symbol;Acc:HGNC:15997]
**0,951674013**	0,000106125	0,000483441	*KCNC3*	potassium voltage-gated channel subfamily C member 3 [Source:HGNC Symbol;Acc:HGNC:6235]
**0,950276517**	1,38E-05	7,19E-05	*B3GALT4*	beta-1,3-galactosyltransferase 4 [Source:HGNC Symbol;Acc:HGNC:919]
**0,948529133**	2,12E-90	2,17E-88	*TLE1*	transducin like enhancer of split 1 [Source:HGNC Symbol;Acc:HGNC:11837]
**0,948286792**	3,10E-37	9,70E-36	*KAT6B*	lysine acetyltransferase 6B [Source:HGNC Symbol;Acc:HGNC:17582]
**0,948013592**	3,12E-23	5,76E-22	*SLC4A8*	solute carrier family 4 member 8 [Source:HGNC Symbol;Acc:HGNC:11034]
**0,947473085**	2,03E-12	2,12E-11	*PAQR8*	progestin and adipoQ receptor family member 8 [Source:HGNC Symbol;Acc:HGNC:15708]
**0,945432472**	1,07E-09	8,73E-09	*SLC47A1*	solute carrier family 47 member 1 [Source:HGNC Symbol;Acc:HGNC:25588]
**0,944072133**	6,40E-58	3,45E-56	*SLC5A3*	solute carrier family 5 member 3 [Source:HGNC Symbol;Acc:HGNC:11038]
**0,943742584**	1,85E-07	1,22E-06	*MAFB*	MAF bZIP transcription factor B [Source:HGNC Symbol;Acc:HGNC:6408]
**0,941571685**	0,014050726	0,039933859	*FOXP2*	forkhead box P2 [Source:HGNC Symbol;Acc:HGNC:13875]
**0,941386655**	1,78E-20	2,93E-19	*PTPN4*	protein tyrosine phosphatase, non-receptor type 4 [Source:HGNC Symbol;Acc:HGNC:9656]
**0,939500679**	1,40E-25	2,89E-24	*MTHFR*	methylenetetrahydrofolate reductase [Source:HGNC Symbol;Acc:HGNC:7436]

**0,939117686**	4,76E-09	3,70E-08	*HIST1H2BD*	histone cluster 1 H2B family member d [Source:HGNC Symbol;Acc:HGNC:4747]
**0,939062243**	1,76E-16	2,36E-15	*GLDC*	glycine decarboxylase [Source:HGNC Symbol;Acc:HGNC:4313]
**0,937974643**	2,70E-06	1,55E-05	*HSD17B8*	hydroxysteroid 17-beta dehydrogenase 8 [Source:HGNC Symbol;Acc:HGNC:3554]
**0,937590462**	6,34E-09	4,86E-08	*LRRC4*	leucine rich repeat containing 4 [Source:HGNC Symbol;Acc:HGNC:15586]
**0,93699009**	0,000144459	0,000641462	*BEND5*	BEN domain containing 5 [Source:HGNC Symbol;Acc:HGNC:25668]
**0,935817007**	0,000282494	0,001184547	*OPRL1*	opioid related nociceptin receptor 1 [Source:HGNC Symbol;Acc:HGNC:8155]
**0,934851968**	0,000246379	0,001044622	*LRRN4CL*	LRRN4 C-terminal like [Source:HGNC Symbol;Acc:HGNC:33724]
**0,934538995**	0,000482616	0,001944693	*ARPP21*	cAMP regulated phosphoprotein 21 [Source:HGNC Symbol;Acc:HGNC:16968]
**0,934404508**	2,69E-06	1,54E-05	*HID1*	HID1 domain containing [Source:HGNC Symbol;Acc:HGNC:15736]
**0,934194434**	0,004108271	0,013521896	*GLTPD2*	glycolipid transfer protein domain containing 2 [Source:HGNC Symbol;Acc:HGNC:33756]
**0,934057484**	1,36E-28	3,16E-27	*RCAN2*	regulator of calcineurin 2 [Source:HGNC Symbol;Acc:HGNC:3041]
**0,932879063**	5,70E-27	1,24E-25	*KCTD13*	potassium channel tetramerization domain containing 13 [Source:HGNC Symbol;Acc:HGNC:22234]
**0,932348234**	4,38E-23	8,03E-22	*C1orf226*	chromosome 1 open reading frame 226 [Source:HGNC Symbol;Acc:HGNC:34351]
**0,930670659**	4,24E-81	3,63E-79	*IDS*	iduronate 2-sulfatase [Source:HGNC Symbol;Acc:HGNC:5389]
**0,930500402**	0,001168086	0,004352513	*ABCA8*	ATP binding cassette subfamily A member 8 [Source:HGNC Symbol;Acc:HGNC:38]
**0,929552863**	1,74E-08	1,28E-07	*IFIT2*	interferon induced protein with tetratricopeptide repeats 2 [Source:HGNC Symbol;Acc:HGNC:5409]
**0,927267515**	4,09E-15	5,04E-14	*RTTN*	rotatin [Source:HGNC Symbol;Acc:HGNC:18654]
**0,92698137**	3,16E-12	3,23E-11	*CHRNB1*	cholinergic receptor nicotinic beta 1 subunit [Source:HGNC Symbol;Acc:HGNC:1961]
**0,921504285**	0,010628238	0,031316669	*SNAI3*	snail family transcriptional repressor 3 [Source:HGNC Symbol;Acc:HGNC:18411]
**0,920535076**	9,03E-36	2,69E-34	*ARHGEF3*	Rho guanine nucleotide exchange factor 3 [Source:HGNC Symbol;Acc:HGNC:683]
**0,91619036**	3,15E-36	9,59E-35	*FAM102A*	family with sequence similarity 102 member A [Source:HGNC Symbol;Acc:HGNC:31419]
**0,915812524**	0,00116174	0,004331358	*RIMS4*	regulating synaptic membrane exocytosis 4 [Source:HGNC Symbol;Acc:HGNC:16183]
**0,914776942**	0,001432955	0,005244799	*FAM189A1*	family with sequence similarity 189 member A1 [Source:HGNC Symbol;Acc:HGNC:29075]
**0,914397818**	0,007471462	0,023003522	*MTUS1*	microtubule associated scaffold protein 1 [Source:HGNC Symbol;Acc:HGNC:29789]
**0,912813691**	3,56E-05	0,000175568	*UNC80*	unc-80 homolog, NALCN channel complex subunit [Source:HGNC Symbol;Acc:HGNC:26582]
**0,912660033**	0,004726035	0,015357239	*FAM78B*	family with sequence similarity 78 member B [Source:HGNC Symbol;Acc:HGNC:13495]
**0,912146452**	0,006207662	0,019600697	*MAGIX*	MAGI family member, X-linked [Source:HGNC Symbol;Acc:HGNC:30006]
**0,911953536**	3,34E-14	3,93E-13	*ABTB1*	ankyrin repeat and BTB domain containing 1 [Source:HGNC Symbol;Acc:HGNC:18275]
**0,911785679**	2,20E-30	5,51E-29	*ADGRE5*	adhesion G protein-coupled receptor E5 [Source:HGNC Symbol;Acc:HGNC:1711]
**0,911462037**	6,82E-78	5,49E-76	*PDLIM4*	PDZ and LIM domain 4 [Source:HGNC Symbol;Acc:HGNC:16501]
**0,910853069**	4,62E-06	2,57E-05	*CREB3L4*	cAMP responsive element binding protein 3 like 4 [Source:HGNC Symbol;Acc:HGNC:18854]
**0,909142768**	8,63E-13	9,23E-12	*ARTN*	artemin [Source:HGNC Symbol;Acc:HGNC:727]

**0,908744841**	1,11E-08	8,35E-08	*PDZD2*	PDZ domain containing 2 [Source:HGNC Symbol;Acc:HGNC:18486]
**0,908120271**	0,000508365	0,002036187	*KLHL3*	kelch like family member 3 [Source:HGNC Symbol;Acc:HGNC:6354]
**0,905131466**	3,16E-11	2,95E-10	*DNER*	delta/notch like EGF repeat containing [Source:HGNC Symbol;Acc:HGNC:24456]
**0,904227837**	4,00E-05	0,000195895	*SIGLEC15*	sialic acid binding Ig like lectin 15 [Source:HGNC Symbol;Acc:HGNC:27596]
**0,90355069**	2,31E-46	9,44E-45	*SDK1*	sidekick cell adhesion molecule 1 [Source:HGNC Symbol;Acc:HGNC:19307]
**0,903530107**	1,24E-56	6,56E-55	*MKNK2*	MAP kinase interacting serine/threonine kinase 2 [Source:HGNC Symbol;Acc:HGNC:7111]
**0,902864373**	1,79E-06	1,06E-05	*REEP1*	receptor accessory protein 1 [Source:HGNC Symbol;Acc:HGNC:25786]
**0,90217437**	3,54E-94	3,86E-92	*GSN*	gelsolin [Source:HGNC Symbol;Acc:HGNC:4620]
**0,901031705**	0,001522155	0,005540449	*TG*	thyroglobulin [Source:HGNC Symbol;Acc:HGNC:11764]
**0,900457838**	5,10E-138	1,05E-135	*TKT*	transketolase [Source:HGNC Symbol;Acc:HGNC:11834]
**0,89975045**	4,89E-07	3,08E-06	*NOXA1*	NADPH oxidase activator 1 [Source:HGNC Symbol;Acc:HGNC:10668]
**0,898395274**	1,88E-62	1,15E-60	*IP6K1*	inositol hexakisphosphate kinase 1 [Source:HGNC Symbol;Acc:HGNC:18360]
**0,898221891**	1,35E-15	1,71E-14	*KCNJ2*	potassium voltage-gated channel subfamily J member 2 [Source:HGNC Symbol;Acc:HGNC:6263]
**0,898172232**	7,54E-07	4,65E-06	*ACACB*	acetyl-CoA carboxylase beta [Source:HGNC Symbol;Acc:HGNC:85]
**0,897306013**	5,31E-08	3,73E-07	*NLGN3*	neuroligin 3 [Source:HGNC Symbol;Acc:HGNC:14289]
**0,896945456**	3,83E-15	4,73E-14	*PIP4P2*	phosphatidylinositol-4,5-bisphosphate 4-phosphatase 2 [Source:HGNC Symbol;Acc:HGNC:25452]
**0,896684145**	3,58E-12	3,63E-11	*PPM1L*	protein phosphatase, Mg2+/Mn2+ dependent 1L [Source:HGNC Symbol;Acc:HGNC:16381]
**0,896309178**	4,06E-06	2,28E-05	*SPATA7*	spermatogenesis associated 7 [Source:HGNC Symbol;Acc:HGNC:20423]
**0,895132601**	1,01E-42	3,72E-41	*CNIH3*	cornichon family AMPA receptor auxiliary protein 3 [Source:HGNC Symbol;Acc:HGNC:26802]
**0,894087364**	4,81E-19	7,42E-18	*ADGRL3*	adhesion G protein-coupled receptor L3 [Source:HGNC Symbol;Acc:HGNC:20974]
**0,893419802**	8,75E-06	4,70E-05	*PTGES*	prostaglandin E synthase [Source:HGNC Symbol;Acc:HGNC:9599]
**0,891838341**	0,000154633	0,000682466	*EBF1*	early B cell factor 1 [Source:HGNC Symbol;Acc:HGNC:3126]
**0,890506502**	9,31E-05	0,000429244	*CBX7*	chromobox 7 [Source:HGNC Symbol;Acc:HGNC:1557]
**0,889592339**	4,22E-07	2,67E-06	*METTL27*	methyltransferase like 27 [Source:HGNC Symbol;Acc:HGNC:19068]
**0,889054087**	1,26E-19	2,00E-18	*MARCH8*	membrane associated ring-CH-type finger 8 [Source:HGNC Symbol;Acc:HGNC:23356]
**0,888620368**	1,51E-63	9,44E-62	*DHCR7*	7-dehydrocholesterol reductase [Source:HGNC Symbol;Acc:HGNC:2860]
**0,887117004**	1,94E-49	8,60E-48	*GBE1*	1,4-alpha-glucan branching enzyme 1 [Source:HGNC Symbol;Acc:HGNC:4180]
**0,886623888**	2,22E-06	1,29E-05	*BEX2*	brain expressed X-linked 2 [Source:HGNC Symbol;Acc:HGNC:30933]
**0,886365263**	0,000921773	0,003518473	*APOBEC3G*	apolipoprotein B mRNA editing enzyme catalytic subunit 3G [Source:HGNC Symbol;Acc:HGNC:17357]
**0,885923003**	1,17E-07	7,93E-07	*PITPNM3*	PITPNM family member 3 [Source:HGNC Symbol;Acc:HGNC:21043]
**0,884834017**	8,98E-14	1,03E-12	*ANKRA2*	ankyrin repeat family A member 2 [Source:HGNC Symbol;Acc:HGNC:13208]
**0,884767465**	0,000831083	0,003206361	*CLDN4*	claudin 4 [Source:HGNC Symbol;Acc:HGNC:2046]
**0,884501519**	0,003970583	0,013119894	*C6orf15*	chromosome 6 open reading frame 15 [Source:HGNC Symbol;Acc:HGNC:13927]
**0,883832623**	1,35E-06	8,11E-06	*NR1D1*	nuclear receptor subfamily 1 group D member 1 [Source:HGNC Symbol;Acc:HGNC:7962]

**0,883468285**	8,92E-14	1,02E-12	*QPCT*	glutaminyl-peptide cyclotransferase [Source:HGNC Symbol;Acc:HGNC:9753]
**0,882350512**	1,60E-13	1,80E-12	*STN1*	STN1, CST complex subunit [Source:HGNC Symbol;Acc:HGNC:26200]
**0,882192554**	5,91E-12	5,85E-11	*CASP8*	caspase 8 [Source:HGNC Symbol;Acc:HGNC:1509]
**0,881089928**	2,41E-19	3,78E-18	*B3GNT2*	UDP-GlcNAc:betaGal beta-1,3-N-acetylglucosaminyltransferase 2 [Source:HGNC Symbol;Acc:HGNC:15629]
**0,879590358**	4,66E-22	8,18E-21	*ACSS3*	acyl-CoA synthetase short chain family member 3 [Source:HGNC Symbol;Acc:HGNC:24723]
**0,875524426**	2,75E-20	4,49E-19	*PC*	pyruvate carboxylase [Source:HGNC Symbol;Acc:HGNC:8636]
**0,874283669**	1,58E-05	8,16E-05	*PCDH18*	protocadherin 18 [Source:HGNC Symbol;Acc:HGNC:14268]
**0,873535821**	8,00E-42	2,84E-40	*TWF2*	twinfilin actin binding protein 2 [Source:HGNC Symbol;Acc:HGNC:9621]
**0,873111821**	6,39E-41	2,21E-39	*MGAT5*	alpha-1,6-mannosylglycoprotein 6-beta-N-acetylglucosaminyltransferase [Source:HGNC Symbol;Acc:HGNC:7049]
**0,872249926**	3,92E-79	3,24E-77	*FDFT1*	farnesyl-diphosphate farnesyltransferase 1 [Source:HGNC Symbol;Acc:HGNC:3629]
**0,871408027**	5,75E-10	4,84E-09	*FOXO4*	forkhead box O4 [Source:HGNC Symbol;Acc:HGNC:7139]
**0,869220365**	2,93E-61	1,73E-59	*IGSF8*	immunoglobulin superfamily member 8 [Source:HGNC Symbol;Acc:HGNC:17813]
**0,869182071**	6,59E-24	1,26E-22	*KIF21A*	kinesin family member 21A [Source:HGNC Symbol;Acc:HGNC:19349]
**0,868534472**	7,57E-16	9,71E-15	*MAF*	MAF bZIP transcription factor [Source:HGNC Symbol;Acc:HGNC:6776]
**0,867901766**	8,44E-27	1,82E-25	*WNT7B*	Wnt family member 7B [Source:HGNC Symbol;Acc:HGNC:12787]
**0,867723427**	6,19E-05	0,000294212	*TMEM121B*	transmembrane protein 121B [Source:HGNC Symbol;Acc:HGNC:1844]
**0,866537166**	2,94E-07	1,90E-06	*DLL1*	delta like canonical Notch ligand 1 [Source:HGNC Symbol;Acc:HGNC:2908]
**0,866167332**	0,001208374	0,00448884	*NR4A1*	nuclear receptor subfamily 4 group A member 1 [Source:HGNC Symbol;Acc:HGNC:7980]
**0,86407509**	1,73E-39	5,77E-38	*PLPP3*	phospholipid phosphatase 3 [Source:HGNC Symbol;Acc:HGNC:9229]
**0,863861081**	0,000318908	0,001324925	*BTBD8*	BTB domain containing 8 [Source:HGNC Symbol;Acc:HGNC:21019]
**0,863067769**	5,78E-10	4,86E-09	*TNFRSF10C*	TNF receptor superfamily member 10c [Source:HGNC Symbol;Acc:HGNC:11906]
**0,862961293**	2,01E-06	1,18E-05	*CPNE4*	copine 4 [Source:HGNC Symbol;Acc:HGNC:2317]
**0,862227897**	0,000480436	0,001936312	*DMRTA1*	DMRT like family A1 [Source:HGNC Symbol;Acc:HGNC:13826]
**0,861274418**	0,013609063	0,038883609	*TAF7L*	TATA-box binding protein associated factor 7 like [Source:HGNC Symbol;Acc:HGNC:11548]
**0,861072582**	5,00E-05	0,000240905	*TRPM3*	transient receptor potential cation channel subfamily M member 3 [Source:HGNC Symbol;Acc:HGNC:17992]
**0,860862511**	3,20E-06	1,82E-05	*SPNS2*	sphingolipid transporter 2 [Source:HGNC Symbol;Acc:HGNC:26992]
**0,859534506**	3,68E-45	1,44E-43	*PGPEP1*	pyroglutamyl-peptidase I [Source:HGNC Symbol;Acc:HGNC:13568]
**0,858720692**	1,34E-65	8,74E-64	*EXTL3*	exostosin like glycosyltransferase 3 [Source:HGNC Symbol;Acc:HGNC:3518]
**0,857174647**	1,03E-08	7,74E-08	*PDE4B*	phosphodiesterase 4B [Source:HGNC Symbol;Acc:HGNC:8781]
**0,856359525**	0,011288819	0,033032393	*SEPT1*	septin 1 [Source:HGNC Symbol;Acc:HGNC:2879]
**0,856140051**	1,01E-14	1,22E-13	*FBXL20*	F-box and leucine rich repeat protein 20 [Source:HGNC Symbol;Acc:HGNC:24679]
**0,855749799**	6,85E-30	1,67E-28	*GSR*	glutathione-disulfide reductase [Source:HGNC Symbol;Acc:HGNC:4623]

**0,855490983**	6,67E-37	2,08E-35	*PREX1*	phosphatidylinositol-3,4,5-trisphosphate dependent Rac exchange factor 1 [Source:HGNC Symbol;Acc:HGNC:32594]
**0,854884303**	7,49E-07	4,62E-06	*SLC27A3*	solute carrier family 27 member 3 [Source:HGNC Symbol;Acc:HGNC:10997]
**0,854212475**	5,18E-08	3,65E-07	*ALDH5A1*	aldehyde dehydrogenase 5 family member A1 [Source:HGNC Symbol;Acc:HGNC:408]
**0,850411471**	1,16E-22	2,08E-21	*B3GNT5*	UDP-GlcNAc:betaGal beta-1,3-N-acetylglucosaminyltransferase 5 [Source:HGNC Symbol;Acc:HGNC:15684]
**0,849170387**	0,000101441	0,000464177	*REEP6*	receptor accessory protein 6 [Source:HGNC Symbol;Acc:HGNC:30078]
**0,848527619**	2,64E-55	1,34E-53	*FRMD4A*	FERM domain containing 4A [Source:HGNC Symbol;Acc:HGNC:25491]
**0,847923526**	0,000143436	0,000637105	*C1orf56*	chromosome 1 open reading frame 56 [Source:HGNC Symbol;Acc:HGNC:26045]
**0,847285275**	3,94E-35	1,16E-33	*DLK1*	delta like non-canonical Notch ligand 1 [Source:HGNC Symbol;Acc:HGNC:2907]
**0,844276549**	1,28E-25	2,65E-24	*DNM1*	dynamin 1 [Source:HGNC Symbol;Acc:HGNC:2972]
**0,84387085**	2,66E-30	6,63E-29	*SAT1*	spermidine/spermine N1-acetyltransferase 1 [Source:HGNC Symbol;Acc:HGNC:10540]
**0,843780944**	4,22E-40	1,43E-38	*PMEPA1*	prostate transmembrane protein, androgen induced 1 [Source:HGNC Symbol;Acc:HGNC:14107]
**0,843551756**	8,66E-29	2,03E-27	*SIK3*	SIK family kinase 3 [Source:HGNC Symbol;Acc:HGNC:29165]
**0,842588358**	0,000411046	0,001678257	*ANKRD24*	ankyrin repeat domain 24 [Source:HGNC Symbol;Acc:HGNC:29424]
**0,841830314**	1,59E-06	9,48E-06	*CYP27A1*	cytochrome P450 family 27 subfamily A member 1 [Source:HGNC Symbol;Acc:HGNC:2605]
**0,841180213**	4,32E-19	6,70E-18	*CAVIN3*	caveolae associated protein 3 [Source:HGNC Symbol;Acc:HGNC:9400]
**0,840867154**	1,69E-59	9,54E-58	*FMNL2*	formin like 2 [Source:HGNC Symbol;Acc:HGNC:18267]
**0,838315114**	9,82E-05	0,000450541	*CMTM8*	CKLF like MARVEL transmembrane domain containing 8 [Source:HGNC Symbol;Acc:HGNC:19179]
**0,837809308**	0,001990776	0,007058835	*CHRNA1*	cholinergic receptor nicotinic alpha 1 subunit [Source:HGNC Symbol;Acc:HGNC:1955]
**0,837087499**	7,87E-19	1,20E-17	*SLC12A6*	solute carrier family 12 member 6 [Source:HGNC Symbol;Acc:HGNC:10914]
**0,836437113**	0,007746901	0,023761082	*STARD8*	StAR related lipid transfer domain containing 8 [Source:HGNC Symbol;Acc:HGNC:19161]
**0,835596364**	6,15E-26	1,29E-24	*CD82*	CD82 molecule [Source:HGNC Symbol;Acc:HGNC:6210]
**0,835584892**	1,89E-13	2,12E-12	*FMN2*	formin 2 [Source:HGNC Symbol;Acc:HGNC:14074]
**0,833846619**	5,71E-12	5,67E-11	*ANGPTL2*	angiopoietin like 2 [Source:HGNC Symbol;Acc:HGNC:490]
**0,833540091**	3,42E-07	2,19E-06	*TDRKH*	tudor and KH domain containing [Source:HGNC Symbol;Acc:HGNC:11713]
**0,832658976**	5,78E-05	0,000276083	*BCHE*	butyrylcholinesterase [Source:HGNC Symbol;Acc:HGNC:983]
**0,832313034**	1,61E-18	2,43E-17	*MMP16*	matrix metallopeptidase 16 [Source:HGNC Symbol;Acc:HGNC:7162]
**0,829921444**	3,86E-35	1,13E-33	*ABHD2*	abhydrolase domain containing 2 [Source:HGNC Symbol;Acc:HGNC:18717]
**0,829782472**	0,006534107	0,020511304	*RTN4R*	reticulon 4 receptor [Source:HGNC Symbol;Acc:HGNC:18601]
**0,829495736**	2,21E-05	0,000112072	*OTUD1*	OTU deubiquitinase 1 [Source:HGNC Symbol;Acc:HGNC:27346]
**0,827755783**	0,005534412	0,017673325	*INKA1*	inka box actin regulator 1 [Source:HGNC Symbol;Acc:HGNC:32480]
**0,827442107**	9,98E-06	5,31E-05	*SEMA6D*	semaphorin 6D [Source:HGNC Symbol;Acc:HGNC:16770]
**0,827432719**	1,27E-11	1,22E-10	*PCYT1B*	phosphate cytidylyltransferase 1, choline, beta [Source:HGNC Symbol;Acc:HGNC:8755]

**0,827009765**	3,96E-08	2,81E-07	*KCNA2*	potassium voltage-gated channel subfamily A member 2 [Source:HGNC Symbol;Acc:HGNC:6220]
**0,826190382**	1,71E-10	1,50E-09	*ISLR*	immunoglobulin superfamily containing leucine rich repeat [Source:HGNC Symbol;Acc:HGNC:6133]
**0,824765286**	0,00018437	0,000802394	*ENPP5*	ectonucleotide pyrophosphatase/phosphodiesterase 5 (putative) [Source:HGNC Symbol;Acc:HGNC:13717]
**0,824681151**	5,46E-12	5,44E-11	*NAT8L*	N-acetyltransferase 8 like [Source:HGNC Symbol;Acc:HGNC:26742]
**0,824612547**	6,48E-12	6,40E-11	*GSTZ1*	glutathione S-transferase zeta 1 [Source:HGNC Symbol;Acc:HGNC:4643]
**0,824587289**	2,04E-07	1,34E-06	*NR1H3*	nuclear receptor subfamily 1 group H member 3 [Source:HGNC Symbol;Acc:HGNC:7966]
**0,824432302**	1,59E-09	1,28E-08	*PTPRN2*	protein tyrosine phosphatase, receptor type N2 [Source:HGNC Symbol;Acc:HGNC:9677]
**0,824250817**	0,015887459	0,044431768	*USP2*	ubiquitin specific peptidase 2 [Source:HGNC Symbol;Acc:HGNC:12618]
**0,823722158**	0,008659456	0,026228407	*PALM3*	paralemmin 3 [Source:HGNC Symbol;Acc:HGNC:33274]
**0,82248195**	1,04E-06	6,35E-06	*CALHM2*	calcium homeostasis modulator family member 2 [Source:HGNC Symbol;Acc:HGNC:23493]
**0,822397186**	8,98E-18	1,31E-16	*WBP1*	WW domain binding protein 1 [Source:HGNC Symbol;Acc:HGNC:12737]
**0,821425183**	1,57E-22	2,82E-21	*SMOX*	spermine oxidase [Source:HGNC Symbol;Acc:HGNC:15862]
**0,821404505**	2,40E-07	1,57E-06	*PKDCC*	protein kinase domain containing, cytoplasmic [Source:HGNC Symbol;Acc:HGNC:25123]
**0,821138733**	1,61E-08	1,19E-07	*BMP7*	bone morphogenetic protein 7 [Source:HGNC Symbol;Acc:HGNC:1074]
**0,820112706**	6,01E-06	3,30E-05	*TMEM171*	transmembrane protein 171 [Source:HGNC Symbol;Acc:HGNC:27031]
**0,820021465**	5,80E-25	1,16E-23	*FXYD6*	FXYD domain containing ion transport regulator 6 [Source:HGNC Symbol;Acc:HGNC:4030]
**0,819950133**	0,000299714	0,001251076	*TTYH2*	tweety family member 2 [Source:HGNC Symbol;Acc:HGNC:13877]
**0,819320557**	1,33E-14	1,60E-13	*BTBD11*	BTB domain containing 11 [Source:HGNC Symbol;Acc:HGNC:23844]
**0,818724041**	4,57E-07	2,88E-06	*EEPD1*	endonuclease/exonuclease/phosphatase family domain containing 1 [Source:HGNC Symbol;Acc:HGNC:22223]
**0,815843263**	1,50E-05	7,76E-05	*SLC6A15*	solute carrier family 6 member 15 [Source:HGNC Symbol;Acc:HGNC:13621]
**0,814621049**	0,012063435	0,03501915	*DSC3*	desmocollin 3 [Source:HGNC Symbol;Acc:HGNC:3037]
**0,814049906**	1,94E-22	3,47E-21	*BAZ2B*	bromodomain adjacent to zinc finger domain 2B [Source:HGNC Symbol;Acc:HGNC:963]
**0,814047536**	1,86E-05	9,53E-05	*ATP6V1G2*	ATPase H+ transporting V1 subunit G2 [Source:HGNC Symbol;Acc:HGNC:862]
**0,81224391**	1,20E-40	4,11E-39	*ABHD4*	abhydrolase domain containing 4 [Source:HGNC Symbol;Acc:HGNC:20154]
**0,811866933**	4,79E-11	4,40E-10	*ITPR1*	inositol 1,4,5-trisphosphate receptor type 1 [Source:HGNC Symbol;Acc:HGNC:6180]
**0,811531723**	1,49E-13	1,68E-12	*GAL3ST4*	galactose-3-O-sulfotransferase 4 [Source:HGNC Symbol;Acc:HGNC:24145]
**0,811462025**	8,36E-36	2,50E-34	*ZMIZ1*	zinc finger MIZ-type containing 1 [Source:HGNC Symbol;Acc:HGNC:16493]
**0,811034957**	9,13E-17	1,24E-15	*ANGPT1*	angiopoietin 1 [Source:HGNC Symbol;Acc:HGNC:484]
**0,81088226**	1,69E-29	4,06E-28	*SLC43A2*	solute carrier family 43 member 2 [Source:HGNC Symbol;Acc:HGNC:23087]
**0,810587405**	1,60E-06	9,51E-06	*CYS1*	cystin 1 [Source:HGNC Symbol;Acc:HGNC:18525]
**0,809350565**	4,10E-36	1,24E-34	*PTK2*	protein tyrosine kinase 2 [Source:HGNC Symbol;Acc:HGNC:9611]
**0,80747601**	5,20E-14	6,02E-13	*GPRASP2*	G protein-coupled receptor associated sorting protein 2 [Source:HGNC Symbol;Acc:HGNC:25169]
**0,806445253**	0,000144516	0,000641462	*TBX18*	T-box 18 [Source:HGNC Symbol;Acc:HGNC:11595]

**0,806250912**	1,43E-34	4,14E-33	*RAMP1*	receptor activity modifying protein 1 [Source:HGNC Symbol;Acc:HGNC:9843]
**0,806061538**	1,65E-26	3,52E-25	*LDLRAD3*	low density lipoprotein receptor class A domain containing 3 [Source:HGNC Symbol;Acc:HGNC:27046]
**0,805820997**	7,18E-55	3,59E-53	*TSPAN14*	tetraspanin 14 [Source:HGNC Symbol;Acc:HGNC:23303]
**0,804980758**	4,72E-07	2,97E-06	*PLD1*	phospholipase D1 [Source:HGNC Symbol;Acc:HGNC:9067]
**0,80380571**	0,015723032	0,044022676	*ISG20*	interferon stimulated exonuclease gene 20 [Source:HGNC Symbol;Acc:HGNC:6130]
**0,803731811**	8,08E-06	4,36E-05	*MTERF2*	mitochondrial transcription termination factor 2 [Source:HGNC Symbol;Acc:HGNC:30779]
**0,803616155**	1,30E-07	8,71E-07	*ZNF844*	zinc finger protein 844 [Source:HGNC Symbol;Acc:HGNC:25932]
**0,803245351**	1,22E-36	3,78E-35	*PRCP*	prolylcarboxypeptidase [Source:HGNC Symbol;Acc:HGNC:9344]
**0,802580555**	4,61E-12	4,62E-11	*F11R*	F11 receptor [Source:HGNC Symbol;Acc:HGNC:14685]
**0,800070371**	1,05E-69	7,25E-68	*MMP14*	matrix metallopeptidase 14 [Source:HGNC Symbol;Acc:HGNC:7160]
**0,799849725**	2,41E-40	8,20E-39	*FZD3*	frizzled class receptor 3 [Source:HGNC Symbol;Acc:HGNC:4041]
**0,799363196**	0,01437973	0,040731504	*MMEL1*	membrane metalloendopeptidase like 1 [Source:HGNC Symbol;Acc:HGNC:14668]
**0,7992161**	1,67E-32	4,51E-31	*ATP2B1*	ATPase plasma membrane Ca2+ transporting 1 [Source:HGNC Symbol;Acc:HGNC:814]
**0,799053203**	3,88E-13	4,27E-12	*GNG11*	G protein subunit gamma 11 [Source:HGNC Symbol;Acc:HGNC:4403]
**0,798464232**	4,91E-10	4,15E-09	*CCDC28A*	coiled-coil domain containing 28A [Source:HGNC Symbol;Acc:HGNC:21098]
**0,798375561**	1,87E-07	1,23E-06	*THNSL1*	threonine synthase like 1 [Source:HGNC Symbol;Acc:HGNC:26160]
**0,798106916**	0,002313861	0,008094969	*PIEZO2*	piezo type mechanosensitive ion channel component 2 [Source:HGNC Symbol;Acc:HGNC:26270]
**0,797607702**	2,45E-06	1,41E-05	*GLYCTK*	glycerate kinase [Source:HGNC Symbol;Acc:HGNC:24247]
**0,797285698**	3,42E-05	0,00016902	*KIZ*	kizuna centrosomal protein [Source:HGNC Symbol;Acc:HGNC:15865]
**0,796294899**	6,36E-09	4,88E-08	*IGSF11*	immunoglobulin superfamily member 11 [Source:HGNC Symbol;Acc:HGNC:16669]
**0,793868156**	0,005837892	0,018550866	*LY6G5C*	lymphocyte antigen 6 family member G5C [Source:HGNC Symbol;Acc:HGNC:13932]
**0,79364638**	0,014523882	0,041038215	*ZNF490*	zinc finger protein 490 [Source:HGNC Symbol;Acc:HGNC:23705]
**0,79306847**	4,68E-16	6,08E-15	*SMAD1*	SMAD family member 1 [Source:HGNC Symbol;Acc:HGNC:6767]
**0,79259016**	0,016346592	0,045558231	*ABHD14A*	abhydrolase domain containing 14A [Source:HGNC Symbol;Acc:HGNC:24538]
**0,792295972**	1,58E-16	2,11E-15	*HMGA2*	high mobility group AT-hook 2 [Source:HGNC Symbol;Acc:HGNC:5009]
**0,792055696**	3,24E-20	5,26E-19	*SESN1*	sestrin 1 [Source:HGNC Symbol;Acc:HGNC:21595]
**0,791958853**	0,000684752	0,002685845	*RILP*	Rab interacting lysosomal protein [Source:HGNC Symbol;Acc:HGNC:30266]
**0,791949207**	1,65E-14	1,97E-13	*IQSEC2*	IQ motif and Sec7 domain 2 [Source:HGNC Symbol;Acc:HGNC:29059]
**0,79194848**	6,89E-05	0,000324586	*SLC15A2*	solute carrier family 15 member 2 [Source:HGNC Symbol;Acc:HGNC:10921]
**0,791912587**	0,000136094	0,000607835	*INPP5D*	inositol polyphosphate-5-phosphatase D [Source:HGNC Symbol;Acc:HGNC:6079]
**0,791651254**	8,01E-06	4,33E-05	*REPS2*	RALBP1 associated Eps domain containing 2 [Source:HGNC Symbol;Acc:HGNC:9963]
**0,791561693**	5,37E-09	4,15E-08	*TMEFF2*	transmembrane protein with EGF like and two follistatin like domains 2 [Source:HGNC Symbol;Acc:HGNC:11867]

**0,790299033**	8,44E-08	5,79E-07	*CDK18*	cyclin dependent kinase 18 [Source:HGNC Symbol;Acc:HGNC:8751]
**0,789736352**	1,43E-17	2,05E-16	*MAP3K4*	mitogen-activated protein kinase kinase kinase 4 [Source:HGNC Symbol;Acc:HGNC:6856]
**0,789680361**	1,33E-05	6,95E-05	*POPDC3*	popeye domain containing 3 [Source:HGNC Symbol;Acc:HGNC:17649]
**0,789329545**	4,90E-21	8,30E-20	*ARMCX1*	armadillo repeat containing X-linked 1 [Source:HGNC Symbol;Acc:HGNC:18073]
**0,788822187**	2,85E-19	4,46E-18	*JARID2*	jumonji and AT-rich interaction domain containing 2 [Source:HGNC Symbol;Acc:HGNC:6196]
**0,786680808**	1,91E-05	9,75E-05	*FOS*	Fos proto-oncogene, AP-1 transcription factor subunit [Source:HGNC Symbol;Acc:HGNC:3796]
**0,785598409**	3,11E-87	3,00E-85	*PSAP*	prosaposin [Source:HGNC Symbol;Acc:HGNC:9498]
**0,784098487**	0,01275439	0,036706466	*KANTR*	KDM5C adjacent transcript [Source:HGNC Symbol;Acc:HGNC:49510]
**0,782317923**	0,016036671	0,044781296	*PPP1R1B*	protein phosphatase 1 regulatory inhibitor subunit 1B [Source:HGNC Symbol;Acc:HGNC:9287]
**0,781560759**	0,000307206	0,001280696	*LRAT*	lecithin retinol acyltransferase [Source:HGNC Symbol;Acc:HGNC:6685]
**0,781274136**	1,63E-11	1,56E-10	*RFX3*	regulatory factor X3 [Source:HGNC Symbol;Acc:HGNC:9984]
**0,780835825**	4,80E-41	1,67E-39	*JUP*	junction plakoglobin [Source:HGNC Symbol;Acc:HGNC:6207]
**0,780323833**	1,74E-42	6,32E-41	*PLTP*	phospholipid transfer protein [Source:HGNC Symbol;Acc:HGNC:9093]
**0,779308501**	1,01E-09	8,27E-09	*RGS10*	regulator of G protein signaling 10 [Source:HGNC Symbol;Acc:HGNC:9992]
**0,778871594**	2,17E-17	3,09E-16	*FLCN*	folliculin [Source:HGNC Symbol;Acc:HGNC:27310]
**0,778621283**	1,22E-16	1,65E-15	*FAM171B*	family with sequence similarity 171 member B [Source:HGNC Symbol;Acc:HGNC:29412]
**0,777565831**	0,012902199	0,037071376	*RAB38*	RAB38, member RAS oncogene family [Source:HGNC Symbol;Acc:HGNC:9776]
**0,777423809**	4,71E-11	4,33E-10	*PLPP2*	phospholipid phosphatase 2 [Source:HGNC Symbol;Acc:HGNC:9230]
**0,777046682**	0,010575482	0,031184897	*NKD1*	naked cuticle homolog 1 [Source:HGNC Symbol;Acc:HGNC:17045]
**0,776073547**	3,06E-08	2,20E-07	*CRYBG1*	crystallin beta-gamma domain containing 1 [Source:HGNC Symbol;Acc:HGNC:356]
**0,775714265**	4,42E-10	3,75E-09	*PTCHD4*	patched domain containing 4 [Source:HGNC Symbol;Acc:HGNC:21345]
**0,775212569**	0,005956091	0,018901702	*ST3GAL6*	ST3 beta-galactoside alpha-2,3-sialyltransferase 6 [Source:HGNC Symbol;Acc:HGNC:18080]
**0,772961919**	1,13E-09	9,24E-09	*NOS1AP*	nitric oxide synthase 1 adaptor protein [Source:HGNC Symbol;Acc:HGNC:16859]
**0,772745114**	4,12E-12	4,15E-11	*DENND4A*	DENN domain containing 4A [Source:HGNC Symbol;Acc:HGNC:24321]
**0,772473691**	3,83E-05	0,000187949	*GSTO2*	glutathione S-transferase omega 2 [Source:HGNC Symbol;Acc:HGNC:23064]
**0,772065405**	0,003947381	0,013058793	*COL15A1*	collagen type XV alpha 1 chain [Source:HGNC Symbol;Acc:HGNC:2192]
**0,771541656**	0,000554444	0,002205727	*PCLO*	piccolo presynaptic cytomatrix protein [Source:HGNC Symbol;Acc:HGNC:13406]
**0,769847858**	1,69E-29	4,06E-28	*CALCOCO1*	calcium binding and coiled-coil domain 1 [Source:HGNC Symbol;Acc:HGNC:29306]
**0,768045984**	2,71E-05	0,000135903	*WIPF3*	WAS/WASL interacting protein family member 3 [Source:HGNC Symbol;Acc:HGNC:22004]
**0,767933385**	1,43E-35	4,23E-34	*ZFAND6*	zinc finger AN1-type containing 6 [Source:HGNC Symbol;Acc:HGNC:30164]
**0,767678238**	4,03E-10	3,44E-09	*SLC22A18*	solute carrier family 22 member 18 [Source:HGNC Symbol;Acc:HGNC:10964]
**0,766638683**	0,000417536	0,001703322	*EPHX2*	epoxide hydrolase 2 [Source:HGNC Symbol;Acc:HGNC:3402]

**0,766168182**	4,44E-63	2,75E-61	*WWTR1*	WW domain containing transcription regulator 1 [Source:HGNC Symbol;Acc:HGNC:24042]
**0,764861319**	4,26E-05	0,000207777	*KBTBD11*	kelch repeat and BTB domain containing 11 [Source:HGNC Symbol;Acc:HGNC:29104]
**0,763353057**	1,03E-23	1,95E-22	*SRPX2*	sushi repeat containing protein X-linked 2 [Source:HGNC Symbol;Acc:HGNC:30668]
**0,761904963**	1,19E-10	1,06E-09	*UAP1L1*	UDP-N-acetylglucosamine pyrophosphorylase 1 like 1 [Source:HGNC Symbol;Acc:HGNC:28082]
**0,760735166**	3,49E-38	1,12E-36	*ADAMTS14*	ADAM metallopeptidase with thrombospondin type 1 motif 14 [Source:HGNC Symbol;Acc:HGNC:14899]
**0,760490883**	3,87E-09	3,03E-08	*GPCPD1*	glycerophosphocholine phosphodiesterase 1 [Source:HGNC Symbol;Acc:HGNC:26957]
**0,760435533**	1,05E-22	1,90E-21	*SLC6A9*	solute carrier family 6 member 9 [Source:HGNC Symbol;Acc:HGNC:11056]
**0,759357334**	8,91E-48	3,78E-46	*POU3F2*	POU class 3 homeobox 2 [Source:HGNC Symbol;Acc:HGNC:9215]
**0,758894385**	8,88E-15	1,07E-13	*SMIM29*	small integral membrane protein 29 [Source:HGNC Symbol;Acc:HGNC:1340]
**0,758810821**	0,011033574	0,03238807	*AATK*	apoptosis associated tyrosine kinase [Source:HGNC Symbol;Acc:HGNC:21]
**0,757692949**	0,000107716	0,000489769	*RORA*	RAR related orphan receptor A [Source:HGNC Symbol;Acc:HGNC:10258]
**0,757214072**	1,71E-05	8,81E-05	*SECTM1*	secreted and transmembrane 1 [Source:HGNC Symbol;Acc:HGNC:10707]
**0,757073017**	0,000796018	0,003084569	*HACD4*	3-hydroxyacyl-CoA dehydratase 4 [Source:HGNC Symbol;Acc:HGNC:20920]
**0,755860258**	3,24E-31	8,33E-30	*NEU1*	neuraminidase 1 [Source:HGNC Symbol;Acc:HGNC:7758]
**0,755612862**	4,97E-10	4,20E-09	*CDKN1C*	cyclin dependent kinase inhibitor 1C [Source:HGNC Symbol;Acc:HGNC:1786]
**0,755292438**	3,09E-45	1,22E-43	*NUMA1*	nuclear mitotic apparatus protein 1 [Source:HGNC Symbol;Acc:HGNC:8059]
**0,755164967**	0,000272774	0,00114701	*GGT1*	gamma-glutamyltransferase 1 [Source:HGNC Symbol;Acc:HGNC:4250]
**0,75471107**	4,47E-05	0,000217482	*ENOX1*	ecto-NOX disulfide-thiol exchanger 1 [Source:HGNC Symbol;Acc:HGNC:25474]
**0,754196833**	1,04E-12	1,11E-11	*FAH*	fumarylacetoacetate hydrolase [Source:HGNC Symbol;Acc:HGNC:3579]
**0,75369057**	1,33E-12	1,40E-11	*TCF7*	transcription factor 7 [Source:HGNC Symbol;Acc:HGNC:11639]
**0,753595318**	2,85E-50	1,28E-48	*MYO1B*	myosin IB [Source:HGNC Symbol;Acc:HGNC:7596]
**0,753235836**	1,61E-42	5,86E-41	*AKR1B1*	aldo-keto reductase family 1 member B [Source:HGNC Symbol;Acc:HGNC:381]
**0,752529517**	9,58E-33	2,60E-31	*TP53INP1*	tumor protein p53 inducible nuclear protein 1 [Source:HGNC Symbol;Acc:HGNC:18022]
**0,751179228**	4,99E-06	2,77E-05	*CADPS2*	calcium dependent secretion activator 2 [Source:HGNC Symbol;Acc:HGNC:16018]
**0,750255239**	1,72E-57	9,17E-56	*TSPAN9*	tetraspanin 9 [Source:HGNC Symbol;Acc:HGNC:21640]
**0,750197518**	3,60E-27	7,83E-26	*ITPRIP*	inositol 1,4,5-trisphosphate receptor interacting protein [Source:HGNC Symbol;Acc:HGNC:29370]
**0,749947986**	7,95E-25	1,58E-23	*SRGAP3*	SLIT-ROBO Rho GTPase activating protein 3 [Source:HGNC Symbol;Acc:HGNC:19744]
**0,748107991**	2,90E-09	2,29E-08	*NPTX1*	neuronal pentraxin 1 [Source:HGNC Symbol;Acc:HGNC:7952]
**0,747200334**	1,25E-10	1,11E-09	*ERMAP*	erythroblast membrane associated protein (Scianna blood group) [Source:HGNC Symbol;Acc:HGNC:15743]
**0,746297028**	8,43E-06	4,54E-05	*TMEM53*	transmembrane protein 53 [Source:HGNC Symbol;Acc:HGNC:26186]
**0,745866185**	0,012599794	0,036344078	*SLC47A2*	solute carrier family 47 member 2 [Source:HGNC Symbol;Acc:HGNC:26439]
**0,745575076**	6,63E-05	0,000313073	*ZBTB20*	zinc finger and BTB domain containing 20 [Source:HGNC Symbol;Acc:HGNC:13503]

**0,745392304**	1,21E-25	2,50E-24	*SV2A*	synaptic vesicle glycoprotein 2A [Source:HGNC Symbol;Acc:HGNC:20566]
**0,745343617**	2,14E-17	3,05E-16	*KIAA1147*	KIAA1147 [Source:HGNC Symbol;Acc:HGNC:29472]
**0,745234067**	6,93E-41	2,39E-39	*SVIL*	supervillin [Source:HGNC Symbol;Acc:HGNC:11480]
**0,745180985**	0,002755715	0,009461677	*PNPLA7*	patatin like phospholipase domain containing 7 [Source:HGNC Symbol;Acc:HGNC:24768]
**0,744142841**	0,009981578	0,029668021	*SMIM10L2B*	small integral membrane protein 10 like 2B [Source:HGNC Symbol;Acc:HGNC:34500]
**0,743669639**	2,11E-10	1,85E-09	*HHAT*	hedgehog acyltransferase [Source:HGNC Symbol;Acc:HGNC:18270]
**0,743644174**	0,001107643	0,004148799	*PRR36*	proline rich 36 [Source:HGNC Symbol;Acc:HGNC:26172]
**0,743433944**	2,61E-13	2,90E-12	*TGFBI*	transforming growth factor beta induced [Source:HGNC Symbol;Acc:HGNC:11771]
**0,743118541**	2,53E-12	2,61E-11	*SLC35E4*	solute carrier family 35 member E4 [Source:HGNC Symbol;Acc:HGNC:17058]
**0,742818723**	2,33E-16	3,10E-15	*DSEL*	dermatan sulfate epimerase like [Source:HGNC Symbol;Acc:HGNC:18144]
**0,742406763**	1,36E-22	2,45E-21	*UNC5B*	unc-5 netrin receptor B [Source:HGNC Symbol;Acc:HGNC:12568]
**0,742341997**	6,66E-21	1,12E-19	*PGAP1*	post-GPI attachment to proteins 1 [Source:HGNC Symbol;Acc:HGNC:25712]
**0,742044221**	1,77E-17	2,53E-16	*SLC25A37*	solute carrier family 25 member 37 [Source:HGNC Symbol;Acc:HGNC:29786]
**0,742010092**	1,88E-18	2,81E-17	*CASTOR3*	CASTOR family member 3 [Source:HGNC Symbol;Acc:HGNC:29954]
**0,740904539**	0,000841214	0,003242223	*OSR1*	odd-skipped related transciption factor 1 [Source:HGNC Symbol;Acc:HGNC:8111]
**0,740340204**	0,001306461	0,004820914	*ZNF792*	zinc finger protein 792 [Source:HGNC Symbol;Acc:HGNC:24751]
**0,740046098**	5,90E-11	5,36E-10	*ACVR2A*	activin A receptor type 2A [Source:HGNC Symbol;Acc:HGNC:173]
**0,739455064**	6,92E-05	0,000325665	*ANO4*	anoctamin 4 [Source:HGNC Symbol;Acc:HGNC:23837]
**0,739101062**	4,10E-08	2,91E-07	*ZNF667*	zinc finger protein 667 [Source:HGNC Symbol;Acc:HGNC:28854]
**0,738581313**	0,004686215	0,015243158	*ALOX12*	arachidonate 12-lipoxygenase, 12S type [Source:HGNC Symbol;Acc:HGNC:429]
**0,738538158**	3,10E-07	2,00E-06	*NBEA*	neurobeachin [Source:HGNC Symbol;Acc:HGNC:7648]
**0,737426038**	0,002132828	0,007522657	*TMEM86A*	transmembrane protein 86A [Source:HGNC Symbol;Acc:HGNC:26890]
**0,736978294**	4,65E-06	2,59E-05	*C6orf226*	chromosome 6 open reading frame 226 [Source:HGNC Symbol;Acc:HGNC:34431]
**0,736935899**	2,21E-15	2,77E-14	*NHS*	NHS actin remodeling regulator [Source:HGNC Symbol;Acc:HGNC:7820]
**0,736865302**	0,004883259	0,015807259	*CACNG8*	calcium voltage-gated channel auxiliary subunit gamma 8 [Source:HGNC Symbol;Acc:HGNC:13628]
**0,735780649**	1,14E-21	1,97E-20	*ANKRD10*	ankyrin repeat domain 10 [Source:HGNC Symbol;Acc:HGNC:20265]
**0,734884312**	1,86E-35	5,50E-34	*PTPRG*	protein tyrosine phosphatase, receptor type G [Source:HGNC Symbol;Acc:HGNC:9671]
**0,734789816**	3,27E-17	4,58E-16	*NADK2*	NAD kinase 2, mitochondrial [Source:HGNC Symbol;Acc:HGNC:26404]
**0,733534988**	3,22E-36	9,78E-35	*PIK3R1*	phosphoinositide-3-kinase regulatory subunit 1 [Source:HGNC Symbol;Acc:HGNC:8979]
**0,733047334**	1,42E-13	1,60E-12	*FAM214A*	family with sequence similarity 214 member A [Source:HGNC Symbol;Acc:HGNC:25609]
**0,732998415**	1,79E-14	2,13E-13	*FNIP1*	folliculin interacting protein 1 [Source:HGNC Symbol;Acc:HGNC:29418]
**0,732377669**	8,50E-29	1,99E-27	*HDAC5*	histone deacetylase 5 [Source:HGNC Symbol;Acc:HGNC:14068]
**0,731777678**	2,45E-17	3,47E-16	*SCRN2*	secernin 2 [Source:HGNC Symbol;Acc:HGNC:30381]
**0,731702084**	7,07E-16	9,09E-15	*CROT*	carnitine O-octanoyltransferase [Source:HGNC Symbol;Acc:HGNC:2366]

**0,731033759**	7,17E-49	3,12E-47	*TPP1*	tripeptidyl peptidase 1 [Source:HGNC Symbol;Acc:HGNC:2073]
**0,730868618**	0,009267235	0,027787385	*ELFN1*	extracellular leucine rich repeat and fibronectin type III domain containing 1 [Source:HGNC Symbol;Acc:HGNC:33154]
**0,728938932**	3,48E-34	9,88E-33	*CCND2*	cyclin D2 [Source:HGNC Symbol;Acc:HGNC:1583]
**0,728012407**	2,04E-10	1,79E-09	*ECM1*	extracellular matrix protein 1 [Source:HGNC Symbol;Acc:HGNC:3153]
**0,727663304**	1,02E-17	1,48E-16	*TYRO3*	TYRO3 protein tyrosine kinase [Source:HGNC Symbol;Acc:HGNC:12446]
**0,726869863**	8,69E-15	1,05E-13	*HSD17B14*	hydroxysteroid 17-beta dehydrogenase 14 [Source:HGNC Symbol;Acc:HGNC:23238]
**0,726700611**	9,57E-23	1,73E-21	*PCMTD2*	protein-L-isoaspartate (D-aspartate) O-methyltransferase domain containing 2 [Source:HGNC Symbol;Acc:HGNC:15882]
**0,726248287**	0,004002409	0,013209311	*CSRNP3*	cysteine and serine rich nuclear protein 3 [Source:HGNC Symbol;Acc:HGNC:30729]
**0,724124929**	1,62E-33	4,50E-32	*APOBEC3C*	apolipoprotein B mRNA editing enzyme catalytic subunit 3C [Source:HGNC Symbol;Acc:HGNC:17353]
**0,723810036**	0,000105505	0,000480845	*CLMN*	calmin [Source:HGNC Symbol;Acc:HGNC:19972]
**0,723581561**	1,01E-08	7,64E-08	*FBLN2*	fibulin 2 [Source:HGNC Symbol;Acc:HGNC:3601]
**0,722725655**	0,000153952	0,000679769	*ACVR2B*	activin A receptor type 2B [Source:HGNC Symbol;Acc:HGNC:174]
**0,722659017**	0,000889969	0,003408473	*FRAT1*	FRAT1, WNT signaling pathway regulator [Source:HGNC Symbol;Acc:HGNC:3944]
**0,721553792**	1,06E-40	3,65E-39	*ANPEP*	alanyl aminopeptidase, membrane [Source:HGNC Symbol;Acc:HGNC:500]
**0,720630896**	2,07E-22	3,68E-21	*SLC27A1*	solute carrier family 27 member 1 [Source:HGNC Symbol;Acc:HGNC:10995]
**0,720204837**	3,17E-15	3,93E-14	*HDAC4*	histone deacetylase 4 [Source:HGNC Symbol;Acc:HGNC:14063]
**0,720043504**	8,53E-26	1,78E-24	*SPRED2*	sprouty related EVH1 domain containing 2 [Source:HGNC Symbol;Acc:HGNC:17722]
**0,719927961**	9,04E-21	1,51E-19	*CXCL12*	C-X-C motif chemokine ligand 12 [Source:HGNC Symbol;Acc:HGNC:10672]
**0,718143363**	2,38E-05	0,000120357	*CRACR2B*	calcium release activated channel regulator 2B [Source:HGNC Symbol;Acc:HGNC:28703]
**0,717909569**	0,005374202	0,017226919	*CCDC88B*	coiled-coil domain containing 88B [Source:HGNC Symbol;Acc:HGNC:26757]
**0,716919432**	8,02E-06	4,34E-05	*DRP2*	dystrophin related protein 2 [Source:HGNC Symbol;Acc:HGNC:3032]
**0,715447492**	4,55E-09	3,54E-08	*PDE4D*	phosphodiesterase 4D [Source:HGNC Symbol;Acc:HGNC:8783]
**0,713687541**	2,19E-28	5,04E-27	*AGPAT5*	1-acylglycerol-3-phosphate O-acyltransferase 5 [Source:HGNC Symbol;Acc:HGNC:20886]
**0,71338837**	1,04E-05	5,53E-05	*STAG3*	stromal antigen 3 [Source:HGNC Symbol;Acc:HGNC:11356]
**0,713296856**	1,48E-10	1,31E-09	*ALDH1L1*	aldehyde dehydrogenase 1 family member L1 [Source:HGNC Symbol;Acc:HGNC:3978]
**0,713172992**	6,29E-16	8,11E-15	*CDK19*	cyclin dependent kinase 19 [Source:HGNC Symbol;Acc:HGNC:19338]
**0,712569958**	0,00098618	0,003733479	*PRDM8*	PR/SET domain 8 [Source:HGNC Symbol;Acc:HGNC:13993]
**0,711509524**	0,000145587	0,000645333	*SLC22A4*	solute carrier family 22 member 4 [Source:HGNC Symbol;Acc:HGNC:10968]
**0,711446657**	1,43E-22	2,58E-21	*COL27A1*	collagen type XXVII alpha 1 chain [Source:HGNC Symbol;Acc:HGNC:22986]
**0,710384125**	1,04E-18	1,58E-17	*MRPS6*	mitochondrial ribosomal protein S6 [Source:HGNC Symbol;Acc:HGNC:14051]
**0,708180043**	2,59E-16	3,42E-15	*MAML2*	mastermind like transcriptional coactivator 2 [Source:HGNC Symbol;Acc:HGNC:16259]
**0,706729337**	7,74E-33	2,11E-31	*TNKS*	tankyrase [Source:HGNC Symbol;Acc:HGNC:11941]

**0,70660186**	0,002873325	0,009832436	*ARHGAP4*	Rho GTPase activating protein 4 [Source:HGNC Symbol;Acc:HGNC:674]
**0,705864609**	5,29E-15	6,50E-14	*TFPI*	tissue factor pathway inhibitor [Source:HGNC Symbol;Acc:HGNC:11760]
**0,705678894**	8,38E-14	9,60E-13	*PKIA*	cAMP-dependent protein kinase inhibitor alpha [Source:HGNC Symbol;Acc:HGNC:9017]
**0,70560377**	2,25E-08	1,64E-07	*UTY*	ubiquitously transcribed tetratricopeptide repeat containing, Y-linked [Source:HGNC Symbol;Acc:HGNC:12638]
**0,70535201**	2,83E-17	3,99E-16	*ZHX2*	zinc fingers and homeoboxes 2 [Source:HGNC Symbol;Acc:HGNC:18513]
**0,705097528**	2,52E-36	7,71E-35	*SSX2IP*	SSX family member 2 interacting protein [Source:HGNC Symbol;Acc:HGNC:16509]
**0,704979987**	1,41E-13	1,60E-12	*SPRED3*	sprouty related EVH1 domain containing 3 [Source:HGNC Symbol;Acc:HGNC:31041]
**0,704681978**	1,05E-05	5,55E-05	*ARHGEF37*	Rho guanine nucleotide exchange factor 37 [Source:HGNC Symbol;Acc:HGNC:34430]
**0,704352303**	4,89E-17	6,77E-16	*RAB13*	RAB13, member RAS oncogene family [Source:HGNC Symbol;Acc:HGNC:9762]
**0,702394616**	1,68E-10	1,47E-09	*ARID4A*	AT-rich interaction domain 4A [Source:HGNC Symbol;Acc:HGNC:9885]
**0,702317966**	3,73E-06	2,10E-05	*HSD17B11*	hydroxysteroid 17-beta dehydrogenase 11 [Source:HGNC Symbol;Acc:HGNC:22960]
**0,700222726**	2,14E-29	5,12E-28	*WSCD1*	WSC domain containing 1 [Source:HGNC Symbol;Acc:HGNC:29060]
**0,699962474**	3,17E-05	0,000157679	*BFSP1*	beaded filament structural protein 1 [Source:HGNC Symbol;Acc:HGNC:1040]
**0,699452721**	6,23E-16	8,04E-15	*HECTD2*	HECT domain E3 ubiquitin protein ligase 2 [Source:HGNC Symbol;Acc:HGNC:26736]
**0,697152918**	0,006377475	0,020081338	*LEF1*	lymphoid enhancer binding factor 1 [Source:HGNC Symbol;Acc:HGNC:6551]
**0,696866684**	0,00079788	0,003089934	*STMN4*	stathmin 4 [Source:HGNC Symbol;Acc:HGNC:16078]
**0,695944569**	4,18E-36	1,26E-34	*INSIG1*	insulin induced gene 1 [Source:HGNC Symbol;Acc:HGNC:6083]
**0,694618813**	1,40E-07	9,36E-07	*RALGDS*	ral guanine nucleotide dissociation stimulator [Source:HGNC Symbol;Acc:HGNC:9842]
**0,694063662**	1,22E-21	2,11E-20	*SIPA1L3*	signal induced proliferation associated 1 like 3 [Source:HGNC Symbol;Acc:HGNC:23801]
**0,693728715**	3,87E-05	0,000190038	*OCEL1*	occludin/ELL domain containing 1 [Source:HGNC Symbol;Acc:HGNC:26221]
**0,693093347**	1,01E-23	1,92E-22	*ANKRD28*	ankyrin repeat domain 28 [Source:HGNC Symbol;Acc:HGNC:29024]
**0,692751909**	1,73E-31	4,49E-30	*XPNPEP1*	X-prolyl aminopeptidase 1 [Source:HGNC Symbol;Acc:HGNC:12822]
**0,691822747**	2,84E-49	1,26E-47	*PBXIP1*	PBX homeobox interacting protein 1 [Source:HGNC Symbol;Acc:HGNC:21199]
**0,691677619**	6,66E-15	8,09E-14	*OTX1*	orthodenticle homeobox 1 [Source:HGNC Symbol;Acc:HGNC:8521]
**0,689789894**	2,89E-10	2,49E-09	*RND2*	Rho family GTPase 2 [Source:HGNC Symbol;Acc:HGNC:18315]
**0,689053987**	4,84E-22	8,49E-21	*CMBL*	carboxymethylenebutenolidase homolog [Source:HGNC Symbol;Acc:HGNC:25090]
**0,689016481**	1,45E-34	4,17E-33	*EMP1*	epithelial membrane protein 1 [Source:HGNC Symbol;Acc:HGNC:3333]
**0,688873381**	1,45E-05	7,52E-05	*CDH10*	cadherin 10 [Source:HGNC Symbol;Acc:HGNC:1749]
**0,687986903**	2,27E-11	2,15E-10	*TMEM108*	transmembrane protein 108 [Source:HGNC Symbol;Acc:HGNC:28451]
**0,68546501**	0,003719976	0,012389059	*CCDC171*	coiled-coil domain containing 171 [Source:HGNC Symbol;Acc:HGNC:29828]
**0,682685218**	0,012101743	0,035119848	*CHRNA7*	cholinergic receptor nicotinic alpha 7 subunit [Source:HGNC Symbol;Acc:HGNC:1960]

**0,68167931**	6,83E-12	6,73E-11	*OBSCN*	obscurin, cytoskeletal calmodulin and titin-interacting RhoGEF [Source:HGNC Symbol;Acc:HGNC:15719]
**0,681053333**	2,42E-07	1,58E-06	*PHYH*	phytanoyl-CoA 2-hydroxylase [Source:HGNC Symbol;Acc:HGNC:8940]
**0,680820222**	7,91E-32	2,08E-30	*AKIRIN2*	akirin 2 [Source:HGNC Symbol;Acc:HGNC:21407]
**0,680661648**	0,00042223	0,001721384	*NR4A2*	nuclear receptor subfamily 4 group A member 2 [Source:HGNC Symbol;Acc:HGNC:7981]
**0,680482524**	8,95E-17	1,22E-15	*IL17RC*	interleukin 17 receptor C [Source:HGNC Symbol;Acc:HGNC:18358]
**0,680181267**	0,002970615	0,010134977	*IDNK*	IDNK, gluconokinase [Source:HGNC Symbol;Acc:HGNC:31367]
**0,679914251**	1,07E-18	1,62E-17	*GLMP*	glycosylated lysosomal membrane protein [Source:HGNC Symbol;Acc:HGNC:29436]
**0,679465588**	1,10E-05	5,82E-05	*TRPV2*	transient receptor potential cation channel subfamily V member 2 [Source:HGNC Symbol;Acc:HGNC:18082]
**0,678676023**	4,96E-07	3,12E-06	*TNFRSF10A*	TNF receptor superfamily member 10a [Source:HGNC Symbol;Acc:HGNC:11904]
**0,67656147**	1,30E-13	1,47E-12	*CARD19*	caspase recruitment domain family member 19 [Source:HGNC Symbol;Acc:HGNC:28148]
**0,676371467**	0,000267614	0,001127265	*OTUB2*	OTU deubiquitinase, ubiquitin aldehyde binding 2 [Source:HGNC Symbol;Acc:HGNC:20351]
**0,676266705**	3,38E-10	2,90E-09	*FAIM2*	Fas apoptotic inhibitory molecule 2 [Source:HGNC Symbol;Acc:HGNC:17067]
**0,676169259**	1,48E-11	1,42E-10	*LONRF1*	LON peptidase N-terminal domain and ring finger 1 [Source:HGNC Symbol;Acc:HGNC:26302]
**0,675870442**	1,13E-05	5,97E-05	*C17orf58*	chromosome 17 open reading frame 58 [Source:HGNC Symbol;Acc:HGNC:27568]
**0,675641562**	1,89E-07	1,25E-06	*RNF208*	ring finger protein 208 [Source:HGNC Symbol;Acc:HGNC:25420]
**0,674965521**	0,000593589	0,002350855	*NRARP*	NOTCH regulated ankyrin repeat protein [Source:HGNC Symbol;Acc:HGNC:33843]
**0,674279756**	3,61E-06	2,04E-05	*CRYL1*	crystallin lambda 1 [Source:HGNC Symbol;Acc:HGNC:18246]
**0,673711307**	0,003926882	0,01299541	*LRRC75A*	leucine rich repeat containing 75A [Source:HGNC Symbol;Acc:HGNC:32403]
**0,672452075**	7,54E-06	4,09E-05	*TDRD3*	tudor domain containing 3 [Source:HGNC Symbol;Acc:HGNC:20612]
**0,672408636**	1,05E-06	6,36E-06	*STOX2*	storkhead box 2 [Source:HGNC Symbol;Acc:HGNC:25450]
**0,670136653**	0,001275253	0,004716517	*TIGD6*	tigger transposable element derived 6 [Source:HGNC Symbol;Acc:HGNC:18332]
**0,669799979**	6,24E-14	7,20E-13	*MPG*	N-methylpurine DNA glycosylase [Source:HGNC Symbol;Acc:HGNC:7211]
**0,669700654**	5,76E-09	4,44E-08	*AMDHD2*	amidohydrolase domain containing 2 [Source:HGNC Symbol;Acc:HGNC:24262]
**0,669305615**	8,64E-10	7,15E-09	*TCF7L2*	transcription factor 7 like 2 [Source:HGNC Symbol;Acc:HGNC:11641]
**0,6688311**	0,016985667	0,047021949	*TBX19*	T-box 19 [Source:HGNC Symbol;Acc:HGNC:11596]
**0,668734117**	0,000115184	0,00052165	*AQP5*	aquaporin 5 [Source:HGNC Symbol;Acc:HGNC:638]
**0,668513832**	0,000865133	0,003320565	*P2RY1*	purinergic receptor P2Y1 [Source:HGNC Symbol;Acc:HGNC:8539]
**0,668102035**	0,001442668	0,005275374	*CSGALNACT1*	chondroitin sulfate N-acetylgalactosaminyltransferase 1 [Source:HGNC Symbol;Acc:HGNC:24290]
**0,668000283**	9,80E-11	8,74E-10	*GAP43*	growth associated protein 43 [Source:HGNC Symbol;Acc:HGNC:4140]
**0,667769612**	4,02E-23	7,38E-22	*HPCAL1*	hippocalcin like 1 [Source:HGNC Symbol;Acc:HGNC:5145]
**0,667746073**	0,004614492	0,015035063	*IGSF9*	immunoglobulin superfamily member 9 [Source:HGNC Symbol;Acc:HGNC:18132]
**0,667731181**	3,92E-36	1,18E-34	*PFKFB3*	6-phosphofructo-2-kinase/fructose-2,6-biphosphatase 3 [Source:HGNC Symbol;Acc:HGNC:8874]

**0,666716461**	7,79E-08	5,38E-07	*INHBB*	inhibin subunit beta B [Source:HGNC Symbol;Acc:HGNC:6067]
**0,666675074**	1,12E-20	1,86E-19	*PNRC1*	proline rich nuclear receptor coactivator 1 [Source:HGNC Symbol;Acc:HGNC:17278]
**0,66666545**	0,00193526	0,006875807	*TLCD1*	TLC domain containing 1 [Source:HGNC Symbol;Acc:HGNC:25177]
**0,665683773**	2,01E-22	3,59E-21	*SCARB1*	scavenger receptor class B member 1 [Source:HGNC Symbol;Acc:HGNC:1664]
**0,664329872**	1,83E-15	2,30E-14	*EPHX1*	epoxide hydrolase 1 [Source:HGNC Symbol;Acc:HGNC:3401]
**0,664163904**	0,002746903	0,009438101	*LRRC56*	leucine rich repeat containing 56 [Source:HGNC Symbol;Acc:HGNC:25430]
**0,663953975**	0,001062243	0,003993392	*HS3ST1*	heparan sulfate-glucosamine 3-sulfotransferase 1 [Source:HGNC Symbol;Acc:HGNC:5194]
**0,663773947**	7,09E-25	1,42E-23	*JAG1*	jagged 1 [Source:HGNC Symbol;Acc:HGNC:6188]
**0,663407118**	8,95E-21	1,49E-19	*BBS2*	Bardet-Biedl syndrome 2 [Source:HGNC Symbol;Acc:HGNC:967]
**0,663040413**	2,45E-17	3,46E-16	*TGIF1*	TGFB induced factor homeobox 1 [Source:HGNC Symbol;Acc:HGNC:11776]
**0,662774011**	8,22E-51	3,73E-49	*ZFAND5*	zinc finger AN1-type containing 5 [Source:HGNC Symbol;Acc:HGNC:13008]
**0,66200806**	4,74E-07	2,99E-06	*FAM110B*	family with sequence similarity 110 member B [Source:HGNC Symbol;Acc:HGNC:28587]
**0,661913426**	3,78E-11	3,51E-10	*KIAA1671*	KIAA1671 [Source:HGNC Symbol;Acc:HGNC:29345]
**0,661182106**	2,57E-13	2,85E-12	*VAT1L*	vesicle amine transport 1 like [Source:HGNC Symbol;Acc:HGNC:29315]
**0,661002165**	9,52E-23	1,73E-21	*UBE2J1*	ubiquitin conjugating enzyme E2 J1 [Source:HGNC Symbol;Acc:HGNC:17598]
**0,659331499**	0,000156794	0,00069122	*CUBN*	cubilin [Source:HGNC Symbol;Acc:HGNC:2548]
**0,659293563**	2,94E-12	3,02E-11	*TNRC6B*	trinucleotide repeat containing 6B [Source:HGNC Symbol;Acc:HGNC:29190]
**0,659174469**	2,25E-28	5,18E-27	*FNDC3A*	fibronectin type III domain containing 3A [Source:HGNC Symbol;Acc:HGNC:20296]
**0,658859272**	1,61E-06	9,56E-06	*CPVL*	carboxypeptidase, vitellogenic like [Source:HGNC Symbol;Acc:HGNC:14399]
**0,657042332**	3,74E-13	4,11E-12	*CDCA7L*	cell division cycle associated 7 like [Source:HGNC Symbol;Acc:HGNC:30777]
**0,656534083**	1,48E-35	4,37E-34	*ZEB1*	zinc finger E-box binding homeobox 1 [Source:HGNC Symbol;Acc:HGNC:11642]
**0,656365931**	2,84E-05	0,000141819	*LRRC4C*	leucine rich repeat containing 4C [Source:HGNC Symbol;Acc:HGNC:29317]
**0,65602073**	5,41E-15	6,63E-14	*CEP57*	centrosomal protein 57 [Source:HGNC Symbol;Acc:HGNC:30794]
**0,655914212**	6,57E-14	7,56E-13	*SPATA18*	spermatogenesis associated 18 [Source:HGNC Symbol;Acc:HGNC:29579]
**0,65402624**	1,59E-19	2,52E-18	*DYRK1B*	dual specificity tyrosine phosphorylation regulated kinase 1B [Source:HGNC Symbol;Acc:HGNC:3092]
**0,653971884**	0,000164479	0,000722473	*AL365205,1*	novel transcript
**0,653963834**	8,65E-05	0,000401099	*PDE6B*	phosphodiesterase 6B [Source:HGNC Symbol;Acc:HGNC:8786]
**0,653738368**	4,17E-18	6,15E-17	*KAT7*	lysine acetyltransferase 7 [Source:HGNC Symbol;Acc:HGNC:17016]
**0,653719383**	7,65E-13	8,24E-12	*SAT2*	spermidine/spermine N1-acetyltransferase family member 2 [Source:HGNC Symbol;Acc:HGNC:23160]
**0,653612103**	2,43E-06	1,40E-05	*CASP9*	caspase 9 [Source:HGNC Symbol;Acc:HGNC:1511]
**0,653488846**	0,006285341	0,01982339	*C9orf72*	chromosome 9 open reading frame 72 [Source:HGNC Symbol;Acc:HGNC:28337]
**0,653240579**	2,01E-05	0,000102467	*ZSCAN26*	zinc finger and SCAN domain containing 26 [Source:HGNC Symbol;Acc:HGNC:12978]
**0,652827189**	2,34E-63	1,46E-61	*CD63*	CD63 molecule [Source:HGNC Symbol;Acc:HGNC:1692]

**0,652572009**	0,001324154	0,004877855	*KCNMB4*	potassium calcium-activated channel subfamily M regulatory beta subunit 4 [Source:HGNC Symbol;Acc:HGNC:6289]
**0,652116162**	0,002253309	0,007905934	*GATA3*	GATA binding protein 3 [Source:HGNC Symbol;Acc:HGNC:4172]
**0,651985854**	1,13E-06	6,85E-06	*STX3*	syntaxin 3 [Source:HGNC Symbol;Acc:HGNC:11438]
**0,651746515**	6,23E-17	8,58E-16	*ANKRD12*	ankyrin repeat domain 12 [Source:HGNC Symbol;Acc:HGNC:29135]
**0,649888337**	3,33E-08	2,39E-07	*PRRT3*	proline rich transmembrane protein 3 [Source:HGNC Symbol;Acc:HGNC:26591]
**0,649746208**	1,58E-08	1,17E-07	*IRAK2*	interleukin 1 receptor associated kinase 2 [Source:HGNC Symbol;Acc:HGNC:6113]
**0,649495444**	0,0005773	0,002290084	*ZNF16*	zinc finger protein 16 [Source:HGNC Symbol;Acc:HGNC:12947]
**0,647697123**	1,48E-25	3,05E-24	*BOC*	BOC cell adhesion associated, oncogene regulated [Source:HGNC Symbol;Acc:HGNC:17173]
**0,647071359**	3,25E-06	1,85E-05	*FN3K*	fructosamine 3 kinase [Source:HGNC Symbol;Acc:HGNC:24822]
**0,64669087**	0,000275827	0,001158842	*CYB5R2*	cytochrome b5 reductase 2 [Source:HGNC Symbol;Acc:HGNC:24376]
**0,646474035**	7,28E-16	9,35E-15	*HIST1H2AC*	histone cluster 1 H2A family member c [Source:HGNC Symbol;Acc:HGNC:4733]
**0,64497909**	0,007296672	0,022540467	*SWT1*	SWT1, RNA endoribonuclease homolog [Source:HGNC Symbol;Acc:HGNC:16785]
**0,644449407**	2,16E-18	3,22E-17	*BIN1*	bridging integrator 1 [Source:HGNC Symbol;Acc:HGNC:1052]
**0,644376257**	4,03E-08	2,86E-07	*TMEM135*	transmembrane protein 135 [Source:HGNC Symbol;Acc:HGNC:26167]
**0,644366267**	4,03E-18	5,94E-17	*GDF15*	growth differentiation factor 15 [Source:HGNC Symbol;Acc:HGNC:30142]
**0,644316808**	0,014993826	0,042151265	*SSPO*	SCO-spondin [Source:HGNC Symbol;Acc:HGNC:21998]
**0,643679527**	6,19E-19	9,51E-18	*BAIAP2*	BAI1 associated protein 2 [Source:HGNC Symbol;Acc:HGNC:947]
**0,643667439**	4,23E-12	4,26E-11	*RIN2*	Ras and Rab interactor 2 [Source:HGNC Symbol;Acc:HGNC:18750]
**0,643666869**	1,27E-05	6,66E-05	*SHF*	Src homology 2 domain containing F [Source:HGNC Symbol;Acc:HGNC:25116]
**0,64362638**	1,82E-05	9,31E-05	*GDPD2*	glycerophosphodiester phosphodiesterase domain containing 2 [Source:HGNC Symbol;Acc:HGNC:25974]
**0,643484452**	7,07E-37	2,20E-35	*GAA*	glucosidase alpha, acid [Source:HGNC Symbol;Acc:HGNC:4065]
**0,642070719**	1,82E-07	1,20E-06	*PNPLA3*	patatin like phospholipase domain containing 3 [Source:HGNC Symbol;Acc:HGNC:18590]
**0,641653212**	1,34E-09	1,09E-08	*MMP17*	matrix metallopeptidase 17 [Source:HGNC Symbol;Acc:HGNC:7163]
**0,640003997**	3,16E-47	1,32E-45	*MDK*	midkine [Source:HGNC Symbol;Acc:HGNC:6972]
**0,639544052**	1,09E-18	1,65E-17	*MGST1*	microsomal glutathione S-transferase 1 [Source:HGNC Symbol;Acc:HGNC:7061]
**0,63953718**	0,006748077	0,021070504	*EID2B*	EP300 interacting inhibitor of differentiation 2B [Source:HGNC Symbol;Acc:HGNC:26796]
**0,638886593**	2,30E-25	4,68E-24	*IGF2BP3*	insulin like growth factor 2 mRNA binding protein 3 [Source:HGNC Symbol;Acc:HGNC:28868]
**0,638785193**	1,85E-11	1,76E-10	*COL13A1*	collagen type XIII alpha 1 chain [Source:HGNC Symbol;Acc:HGNC:2190]
**0,638574453**	1,12E-15	1,43E-14	*BBC3*	BCL2 binding component 3 [Source:HGNC Symbol;Acc:HGNC:17868]
**0,636863293**	3,49E-16	4,56E-15	*JADE2*	jade family PHD finger 2 [Source:HGNC Symbol;Acc:HGNC:22984]
**0,636583462**	0,003978873	0,013138336	*SYCE1L*	synaptonemal complex central element protein 1 like [Source:HGNC Symbol;Acc:HGNC:37236]
**0,634512985**	1,67E-25	3,44E-24	*SPECC1*	sperm antigen with calponin homology and coiled-coil domains 1 [Source:HGNC Symbol;Acc:HGNC:30615]

**0,63352586**	0,018248471	0,049997153	*THBS4*	thrombospondin 4 [Source:HGNC Symbol;Acc:HGNC:11788]
**0,63337305**	6,11E-20	9,82E-19	*DNMBP*	dynamin binding protein [Source:HGNC Symbol;Acc:HGNC:30373]
**0,633052212**	7,34E-06	3,99E-05	*NBPF19*	NBPF member 19 [Source:HGNC Symbol;Acc:HGNC:31999]
**0,632908435**	8,34E-13	8,95E-12	*ACTR3B*	ARP3 actin related protein 3 homolog B [Source:HGNC Symbol;Acc:HGNC:17256]
**0,632839726**	7,44E-23	1,36E-21	*IGDCC4*	immunoglobulin superfamily DCC subclass member 4 [Source:HGNC Symbol;Acc:HGNC:13770]
**0,632708438**	2,31E-06	1,34E-05	*SFMBT2*	Scm like with four mbt domains 2 [Source:HGNC Symbol;Acc:HGNC:20256]
**0,632604723**	2,35E-35	6,95E-34	*CAMK2D*	calcium/calmodulin dependent protein kinase II delta [Source:HGNC Symbol;Acc:HGNC:1462]
**0,632168644**	0,008631768	0,026152707	*F8*	coagulation factor VIII [Source:HGNC Symbol;Acc:HGNC:3546]
**0,631337787**	4,97E-12	4,97E-11	*E2F7*	E2F transcription factor 7 [Source:HGNC Symbol;Acc:HGNC:23820]
**0,631006951**	3,70E-06	2,09E-05	*ZC3H6*	zinc finger CCCH-type containing 6 [Source:HGNC Symbol;Acc:HGNC:24762]
**0,630823321**	0,00010171	0,000465297	*PTCHD1*	patched domain containing 1 [Source:HGNC Symbol;Acc:HGNC:26392]
**0,628772467**	3,14E-20	5,10E-19	*LHFPL2*	LHFPL tetraspan subfamily member 2 [Source:HGNC Symbol;Acc:HGNC:6588]
**0,628672359**	2,82E-30	7,02E-29	*WBP2*	WW domain binding protein 2 [Source:HGNC Symbol;Acc:HGNC:12738]
**0,627402027**	6,79E-12	6,70E-11	*ARHGEF9*	Cdc42 guanine nucleotide exchange factor 9 [Source:HGNC Symbol;Acc:HGNC:14561]
**0,627151715**	8,38E-30	2,03E-28	*FOXG1*	forkhead box G1 [Source:HGNC Symbol;Acc:HGNC:3811]
**0,627119556**	4,67E-14	5,44E-13	*CNTN1*	contactin 1 [Source:HGNC Symbol;Acc:HGNC:2171]
**0,626955782**	2,98E-07	1,92E-06	*SLC35E2A*	solute carrier family 35 member E2A [Source:HGNC Symbol;Acc:HGNC:20863]
**0,626746103**	4,96E-12	4,96E-11	*SCPEP1*	serine carboxypeptidase 1 [Source:HGNC Symbol;Acc:HGNC:29507]
**0,62651502**	1,45E-24	2,86E-23	*SPRED1*	sprouty related EVH1 domain containing 1 [Source:HGNC Symbol;Acc:HGNC:20249]
**0,626079624**	4,25E-58	2,31E-56	*SCD*	stearoyl-CoA desaturase [Source:HGNC Symbol;Acc:HGNC:10571]
**0,625558484**	2,13E-33	5,89E-32	*CDKN2A*	cyclin dependent kinase inhibitor 2A [Source:HGNC Symbol;Acc:HGNC:1787]
**0,625238528**	2,18E-19	3,44E-18	*ZNF219*	zinc finger protein 219 [Source:HGNC Symbol;Acc:HGNC:13011]
**0,624175537**	0,008264503	0,025149906	*GRIN2D*	glutamate ionotropic receptor NMDA type subunit 2D [Source:HGNC Symbol;Acc:HGNC:4588]
**0,622748343**	4,34E-11	4,02E-10	*MLLT3*	MLLT3, super elongation complex subunit [Source:HGNC Symbol;Acc:HGNC:7136]
**0,622679435**	0,002045429	0,007239394	*MGP*	matrix Gla protein [Source:HGNC Symbol;Acc:HGNC:7060]
**0,621693643**	1,37E-30	3,45E-29	*SESN3*	sestrin 3 [Source:HGNC Symbol;Acc:HGNC:23060]
**0,62166453**	8,16E-08	5,62E-07	*CRIPT*	CXXC repeat containing interactor of PDZ3 domain [Source:HGNC Symbol;Acc:HGNC:14312]
**0,621249823**	1,94E-13	2,17E-12	*SMIM14*	small integral membrane protein 14 [Source:HGNC Symbol;Acc:HGNC:27321]
**0,62116837**	6,39E-19	9,81E-18	*PAX6*	paired box 6 [Source:HGNC Symbol;Acc:HGNC:8620]
**0,619835776**	5,31E-09	4,10E-08	*TK2*	thymidine kinase 2, mitochondrial [Source:HGNC Symbol;Acc:HGNC:11831]
**0,619442672**	2,44E-05	0,000123163	*RBM43*	RNA binding motif protein 43 [Source:HGNC Symbol;Acc:HGNC:24790]
**0,619381238**	0,005260631	0,016890769	*EPHX4*	epoxide hydrolase 4 [Source:HGNC Symbol;Acc:HGNC:23758]
**0,619369368**	5,70E-08	3,99E-07	*CCDC115*	coiled-coil domain containing 115 [Source:HGNC Symbol;Acc:HGNC:28178]

**0,619339157**	0,000145781	0,000646043	*FGD6*	FYVE, RhoGEF and PH domain containing 6 [Source:HGNC Symbol;Acc:HGNC:21740]
**0,619283292**	0,009124453	0,027435563	*SLC16A13*	solute carrier family 16 member 13 [Source:HGNC Symbol;Acc:HGNC:31037]
**0,618523883**	0,007158109	0,022158281	*IFI44*	interferon induced protein 44 [Source:HGNC Symbol;Acc:HGNC:16938]
**0,618257746**	5,81E-06	3,20E-05	*APOE*	apolipoprotein E [Source:HGNC Symbol;Acc:HGNC:613]
**0,617811156**	2,23E-06	1,30E-05	*EXD3*	exonuclease 3'-5' domain containing 3 [Source:HGNC Symbol;Acc:HGNC:26023]
**0,617347453**	5,23E-18	7,67E-17	*SYNGR1*	synaptogyrin 1 [Source:HGNC Symbol;Acc:HGNC:11498]
**0,616643549**	8,06E-27	1,74E-25	*IDH1*	isocitrate dehydrogenase (NADP(+)) 1, cytosolic [Source:HGNC Symbol;Acc:HGNC:5382]
**0,616557172**	1,54E-05	7,94E-05	*CARF*	calcium responsive transcription factor [Source:HGNC Symbol;Acc:HGNC:14435]
**0,616167108**	7,57E-16	9,71E-15	*HS6ST2*	heparan sulfate 6-O-sulfotransferase 2 [Source:HGNC Symbol;Acc:HGNC:19133]
**0,615997604**	2,30E-06	1,34E-05	*GAREM1*	GRB2 associated regulator of MAPK1 subtype 1 [Source:HGNC Symbol;Acc:HGNC:26136]
**0,615554686**	0,000826838	0,003193158	*ZNF350*	zinc finger protein 350 [Source:HGNC Symbol;Acc:HGNC:16656]
**0,615465002**	3,44E-11	3,21E-10	*TSPYL2*	TSPY like 2 [Source:HGNC Symbol;Acc:HGNC:24358]
**0,615263096**	7,60E-05	0,000354969	*PIRT*	phosphoinositide interacting regulator of transient receptor potential channels [Source:HGNC Symbol;Acc:HGNC:37239]
**0,615112539**	0,008670851	0,026250629	*ZNF600*	zinc finger protein 600 [Source:HGNC Symbol;Acc:HGNC:30951]
**0,614996027**	0,001452847	0,005308594	*ZNF441*	zinc finger protein 441 [Source:HGNC Symbol;Acc:HGNC:20875]
**0,614710332**	0,006776698	0,021132671	*NAP1L2*	nucleosome assembly protein 1 like 2 [Source:HGNC Symbol;Acc:HGNC:7638]
**0,614183609**	9,98E-25	1,98E-23	*MKRN1*	makorin ring finger protein 1 [Source:HGNC Symbol;Acc:HGNC:7112]
**0,613227601**	2,99E-05	0,000149106	*PLAG1*	PLAG1 zinc finger [Source:HGNC Symbol;Acc:HGNC:9045]
**0,612675718**	2,87E-08	2,07E-07	*HRH1*	histamine receptor H1 [Source:HGNC Symbol;Acc:HGNC:5182]
**0,611982796**	8,59E-05	0,000398727	*FGGY*	FGGY carbohydrate kinase domain containing [Source:HGNC Symbol;Acc:HGNC:25610]
**0,611979062**	2,99E-08	2,15E-07	*TRMT1L*	tRNA methyltransferase 1 like [Source:HGNC Symbol;Acc:HGNC:16782]
**0,611832787**	0,000484201	0,001949864	*AZIN2*	antizyme inhibitor 2 [Source:HGNC Symbol;Acc:HGNC:29957]
**0,610812446**	1,33E-38	4,31E-37	*JUN*	Jun proto-oncogene, AP-1 transcription factor subunit [Source:HGNC Symbol;Acc:HGNC:6204]
**0,610412837**	2,14E-27	4,72E-26	*FADS1*	fatty acid desaturase 1 [Source:HGNC Symbol;Acc:HGNC:3574]
**0,610158972**	1,31E-17	1,88E-16	*SCN4B*	sodium voltage-gated channel beta subunit 4 [Source:HGNC Symbol;Acc:HGNC:10592]
**0,609982406**	1,70E-05	8,75E-05	*RAB3IP*	RAB3A interacting protein [Source:HGNC Symbol;Acc:HGNC:16508]
**0,609478036**	3,85E-18	5,70E-17	*SPATA13*	spermatogenesis associated 13 [Source:HGNC Symbol;Acc:HGNC:23222]
**0,609390382**	3,89E-17	5,41E-16	*HOMER3*	homer scaffold protein 3 [Source:HGNC Symbol;Acc:HGNC:17514]
**0,609122251**	3,06E-20	4,98E-19	*COX6C*	cytochrome c oxidase subunit 6C [Source:HGNC Symbol;Acc:HGNC:2285]
**0,608444923**	5,75E-11	5,23E-10	*TUT7*	terminal uridylyl transferase 7 [Source:HGNC Symbol;Acc:HGNC:25817]
**0,608363047**	8,26E-15	1,00E-13	*SACM1L*	SAC1 like phosphatidylinositide phosphatase [Source:HGNC Symbol;Acc:HGNC:17059]
**0,60795933**	1,90E-09	1,52E-08	*NYNRIN*	NYN domain and retroviral integrase containing [Source:HGNC Symbol;Acc:HGNC:20165]

**0,607442449**	0,000107556	0,000489154	*PIGP*	phosphatidylinositol glycan anchor biosynthesis class P [Source:HGNC Symbol;Acc:HGNC:3046]
**0,607050237**	3,08E-18	4,56E-17	*DPF3*	double PHD fingers 3 [Source:HGNC Symbol;Acc:HGNC:17427]
**0,60666658**	0,00031416	0,001306599	*SLX4IP*	SLX4 interacting protein [Source:HGNC Symbol;Acc:HGNC:16225]
**0,606266641**	0,00019994	0,000862612	*TSNARE1*	t-SNARE domain containing 1 [Source:HGNC Symbol;Acc:HGNC:26437]
**0,605699431**	1,07E-07	7,27E-07	*C22orf46*	chromosome 22 open reading frame 46 [Source:HGNC Symbol;Acc:HGNC:26294]
**0,605631049**	3,04E-11	2,85E-10	*DLG3*	discs large MAGUK scaffold protein 3 [Source:HGNC Symbol;Acc:HGNC:2902]
**0,605057347**	1,48E-25	3,05E-24	*HMGCR*	3-hydroxy-3-methylglutaryl-CoA reductase [Source:HGNC Symbol;Acc:HGNC:5006]
**0,605030359**	0,000221143	0,000945474	*FAM117A*	family with sequence similarity 117 member A [Source:HGNC Symbol;Acc:HGNC:24179]
**0,603857729**	1,26E-16	1,70E-15	*HIP1R*	huntingtin interacting protein 1 related [Source:HGNC Symbol;Acc:HGNC:18415]
**0,603625801**	6,20E-06	3,40E-05	*TRIM66*	tripartite motif containing 66 [Source:HGNC Symbol;Acc:HGNC:29005]
**0,603265478**	2,62E-06	1,51E-05	*UBN2*	ubinuclein 2 [Source:HGNC Symbol;Acc:HGNC:21931]
**0,603245975**	1,08E-44	4,16E-43	*PLD3*	phospholipase D family member 3 [Source:HGNC Symbol;Acc:HGNC:17158]
**0,602992171**	0,003794334	0,012595425	*GTF2IRD2*	GTF2I repeat domain containing 2 [Source:HGNC Symbol;Acc:HGNC:30775]
**0,602851647**	9,76E-38	3,09E-36	*PGD*	phosphogluconate dehydrogenase [Source:HGNC Symbol;Acc:HGNC:8891]
**0,602751241**	1,51E-18	2,27E-17	*PDCD4*	programmed cell death 4 [Source:HGNC Symbol;Acc:HGNC:8763]
**0,602606528**	2,96E-09	2,34E-08	*BHLHE41*	basic helix-loop-helix family member e41 [Source:HGNC Symbol;Acc:HGNC:16617]
**0,601901533**	2,58E-05	0,000129819	*SEMA6B*	semaphorin 6B [Source:HGNC Symbol;Acc:HGNC:10739]
**0,601885011**	2,81E-10	2,42E-09	*CACFD1*	calcium channel flower domain containing 1 [Source:HGNC Symbol;Acc:HGNC:1365]
**0,601478164**	1,81E-10	1,59E-09	*C1orf21*	chromosome 1 open reading frame 21 [Source:HGNC Symbol;Acc:HGNC:15494]
**0,601406553**	2,71E-09	2,15E-08	*ABHD14B*	abhydrolase domain containing 14B [Source:HGNC Symbol;Acc:HGNC:28235]
**0,600664261**	8,53E-14	9,76E-13	*SUSD6*	sushi domain containing 6 [Source:HGNC Symbol;Acc:HGNC:19956]
**0,599898587**	0,012342939	0,035693794	*MFSD13A*	major facilitator superfamily domain containing 13A [Source:HGNC Symbol;Acc:HGNC:26196]
**0,599431127**	9,41E-06	5,02E-05	*JCAD*	junctional cadherin 5 associated [Source:HGNC Symbol;Acc:HGNC:29283]
**0,599255894**	0,000190408	0,000826263	*BDH1*	3-hydroxybutyrate dehydrogenase 1 [Source:HGNC Symbol;Acc:HGNC:1027]
**0,59873387**	2,00E-18	3,00E-17	*REV3L*	REV3 like, DNA directed polymerase zeta catalytic subunit [Source:HGNC Symbol;Acc:HGNC:9968]
**0,598428701**	8,98E-10	7,41E-09	*DEDD2*	death effector domain containing 2 [Source:HGNC Symbol;Acc:HGNC:24450]
**0,598276563**	3,32E-16	4,36E-15	*ZFYVE1*	zinc finger FYVE-type containing 1 [Source:HGNC Symbol;Acc:HGNC:13180]
**0,598038095**	1,02E-06	6,18E-06	*TDRP*	testis development related protein [Source:HGNC Symbol;Acc:HGNC:26951]
**0,598027779**	5,14E-13	5,62E-12	*ATOX1*	antioxidant 1 copper chaperone [Source:HGNC Symbol;Acc:HGNC:798]
**0,597409513**	2,68E-28	6,12E-27	*TXN*	thioredoxin [Source:HGNC Symbol;Acc:HGNC:12435]
**0,596480325**	0,001175029	0,004374183	*SETDB2*	SET domain bifurcated 2 [Source:HGNC Symbol;Acc:HGNC:20263]
**0,595373315**	1,39E-08	1,03E-07	*EFNB3*	ephrin B3 [Source:HGNC Symbol;Acc:HGNC:3228]
**0,594951933**	3,80E-07	2,42E-06	*PRTFDC1*	phosphoribosyl transferase domain containing 1 [Source:HGNC Symbol;Acc:HGNC:23333]

**0,594353163**	7,56E-10	6,28E-09	*TRPS1*	transcriptional repressor GATA binding 1 [Source:HGNC Symbol;Acc:HGNC:12340]
**0,593554311**	2,59E-27	5,69E-26	*ACSL3*	acyl-CoA synthetase long chain family member 3 [Source:HGNC Symbol;Acc:HGNC:3570]
**0,593496588**	2,92E-06	1,67E-05	*CNOT6L*	CCR4-NOT transcription complex subunit 6 like [Source:HGNC Symbol;Acc:HGNC:18042]
**0,593385097**	1,50E-08	1,12E-07	*TMEM260*	transmembrane protein 260 [Source:HGNC Symbol;Acc:HGNC:20185]
**0,592771038**	1,07E-07	7,28E-07	*BLVRB*	biliverdin reductase B [Source:HGNC Symbol;Acc:HGNC:1063]
**0,590921124**	9,57E-07	5,84E-06	*SOX8*	SRY-box 8 [Source:HGNC Symbol;Acc:HGNC:11203]
**0,590200266**	3,15E-05	0,000156755	*PCDHGB6*	protocadherin gamma subfamily B, 6 [Source:HGNC Symbol;Acc:HGNC:8713]
**0,590048748**	0,000445319	0,001805653	*LRRC32*	leucine rich repeat containing 32 [Source:HGNC Symbol;Acc:HGNC:4161]
**0,589668839**	1,74E-09	1,40E-08	*PCDHGB7*	protocadherin gamma subfamily B, 7 [Source:HGNC Symbol;Acc:HGNC:8714]
**0,589503002**	0,000990815	0,003749564	*CDHR1*	cadherin related family member 1 [Source:HGNC Symbol;Acc:HGNC:14550]
**0,589385634**	0,000236001	0,001003909	*GGT5*	gamma-glutamyltransferase 5 [Source:HGNC Symbol;Acc:HGNC:4260]
**0,589162759**	1,37E-06	8,19E-06	*POU3F4*	POU class 3 homeobox 4 [Source:HGNC Symbol;Acc:HGNC:9217]
**0,589150929**	0,00109505	0,004108766	*MTHFD2L*	methylenetetrahydrofolate dehydrogenase (NADP+ dependent) 2 like [Source:HGNC Symbol;Acc:HGNC:31865]
**0,589085759**	4,58E-25	9,20E-24	*ATP6V0E2*	ATPase H+ transporting V0 subunit e2 [Source:HGNC Symbol;Acc:HGNC:21723]
**0,588725417**	3,06E-10	2,64E-09	*CBFA2T2*	CBFA2/RUNX1 translocation partner 2 [Source:HGNC Symbol;Acc:HGNC:1536]
**0,588407168**	4,01E-06	2,25E-05	*FGF12*	fibroblast growth factor 12 [Source:HGNC Symbol;Acc:HGNC:3668]
**0,586683472**	1,05E-14	1,27E-13	*CRELD1*	cysteine rich with EGF like domains 1 [Source:HGNC Symbol;Acc:HGNC:14630]
**0,586499254**	2,64E-20	4,32E-19	*TP53I3*	tumor protein p53 inducible protein 3 [Source:HGNC Symbol;Acc:HGNC:19373]
**0,586265408**	7,70E-44	2,90E-42	*SOX2*	SRY-box 2 [Source:HGNC Symbol;Acc:HGNC:11195]
**0,585204284**	0,000165095	0,000724754	*GNG7*	G protein subunit gamma 7 [Source:HGNC Symbol;Acc:HGNC:4410]
**0,584888834**	0,015626885	0,043766105	*SH3BP1*	SH3 domain binding protein 1 [Source:HGNC Symbol;Acc:HGNC:10824]
**0,584496129**	1,12E-16	1,51E-15	*ITGA6*	integrin subunit alpha 6 [Source:HGNC Symbol;Acc:HGNC:6142]
**0,584106911**	0,009104841	0,027392688	*KLHL14*	kelch like family member 14 [Source:HGNC Symbol;Acc:HGNC:29266]
**0,583931786**	0,005695028	0,018117659	*MPP4*	membrane palmitoylated protein 4 [Source:HGNC Symbol;Acc:HGNC:13680]
**0,58336502**	2,37E-12	2,45E-11	*SPATS2*	spermatogenesis associated serine rich 2 [Source:HGNC Symbol;Acc:HGNC:18650]
**0,583264606**	2,84E-05	0,000141819	*SLC16A4*	solute carrier family 16 member 4 [Source:HGNC Symbol;Acc:HGNC:10925]
**0,582658408**	3,92E-41	1,36E-39	*VAT1*	vesicle amine transport 1 [Source:HGNC Symbol;Acc:HGNC:16919]
**0,581835147**	2,91E-16	3,84E-15	*TOB1*	transducer of ERBB2, 1 [Source:HGNC Symbol;Acc:HGNC:11979]
**0,581764456**	0,000193874	0,000840554	*PHACTR1*	phosphatase and actin regulator 1 [Source:HGNC Symbol;Acc:HGNC:20990]
**0,581492274**	1,16E-08	8,66E-08	*DAB1*	DAB1, reelin adaptor protein [Source:HGNC Symbol;Acc:HGNC:2661]
**0,58139668**	0,00562572	0,017932445	*RAC2*	Rac family small GTPase 2 [Source:HGNC Symbol;Acc:HGNC:9802]

**0,581305988**	2,90E-06	1,66E-05	*FGF13*	fibroblast growth factor 13 [Source:HGNC Symbol;Acc:HGNC:3670]
**0,580576968**	5,41E-05	0,000259537	*WNT9A*	Wnt family member 9A [Source:HGNC Symbol;Acc:HGNC:12778]
**0,580385732**	0,006738603	0,021057864	*IZUMO4*	IZUMO family member 4 [Source:HGNC Symbol;Acc:HGNC:26950]
**0,580010194**	6,60E-14	7,59E-13	*MAN1A1*	mannosidase alpha class 1A member 1 [Source:HGNC Symbol;Acc:HGNC:6821]
**0,579736929**	1,16E-07	7,86E-07	*MTMR11*	myotubularin related protein 11 [Source:HGNC Symbol;Acc:HGNC:24307]
**0,579556091**	5,22E-12	5,20E-11	*MARF1*	meiosis regulator and mRNA stability factor 1 [Source:HGNC Symbol;Acc:HGNC:29562]
**0,578302505**	2,82E-20	4,61E-19	*GPM6A*	glycoprotein M6A [Source:HGNC Symbol;Acc:HGNC:4460]
**0,577991863**	0,003638084	0,012145304	*ZBTB3*	zinc finger and BTB domain containing 3 [Source:HGNC Symbol;Acc:HGNC:22918]
**0,577780364**	0,012838642	0,036910619	*C11orf71*	chromosome 11 open reading frame 71 [Source:HGNC Symbol;Acc:HGNC:25937]
**0,5772181**	0,002482244	0,008623422	*ZNF44*	zinc finger protein 44 [Source:HGNC Symbol;Acc:HGNC:13110]
**0,576495296**	4,12E-12	4,15E-11	*ISLR2*	immunoglobulin superfamily containing leucine rich repeat 2 [Source:HGNC Symbol;Acc:HGNC:29286]
**0,576480552**	3,90E-16	5,09E-15	*MCC*	MCC, WNT signaling pathway regulator [Source:HGNC Symbol;Acc:HGNC:6935]
**0,576373474**	3,36E-14	3,94E-13	*CHD9*	chromodomain helicase DNA binding protein 9 [Source:HGNC Symbol;Acc:HGNC:25701]
**0,575647349**	2,82E-39	9,29E-38	*TXNRD1*	thioredoxin reductase 1 [Source:HGNC Symbol;Acc:HGNC:12437]
**0,575605296**	3,46E-07	2,22E-06	*MAP2K5*	mitogen-activated protein kinase kinase 5 [Source:HGNC Symbol;Acc:HGNC:6845]
**0,575349584**	0,009206265	0,027647287	*SLC10A7*	solute carrier family 10 member 7 [Source:HGNC Symbol;Acc:HGNC:23088]
**0,575141974**	3,33E-11	3,11E-10	*SEPT6*	septin 6 [Source:HGNC Symbol;Acc:HGNC:15848]
**0,574993627**	0,006325621	0,019940709	*CNTNAP3B*	contactin associated protein like 3B [Source:HGNC Symbol;Acc:HGNC:32035]
**0,574114265**	2,42E-17	3,43E-16	*SLC16A2*	solute carrier family 16 member 2 [Source:HGNC Symbol;Acc:HGNC:10923]
**0,57407494**	0,002061498	0,007289618	*CASTOR2*	cytosolic arginine sensor for mTORC1 subunit 2 [Source:HGNC Symbol;Acc:HGNC:37073]
**0,574009916**	0,005280614	0,016946519	*NEK8*	NIMA related kinase 8 [Source:HGNC Symbol;Acc:HGNC:13387]
**0,573354471**	4,61E-05	0,000223327	*PCCA*	propionyl-CoA carboxylase subunit alpha [Source:HGNC Symbol;Acc:HGNC:8653]
**0,571927202**	0,012600379	0,036344078	*DGKG*	diacylglycerol kinase gamma [Source:HGNC Symbol;Acc:HGNC:2853]
**0,571645601**	2,64E-09	2,10E-08	*EZH1*	enhancer of zeste 1 polycomb repressive complex 2 subunit [Source:HGNC Symbol;Acc:HGNC:3526]
**0,570749469**	6,84E-10	5,71E-09	*ACP6*	acid phosphatase 6, lysophosphatidic [Source:HGNC Symbol;Acc:HGNC:29609]
**0,570229561**	3,23E-10	2,77E-09	*JMY*	junction mediating and regulatory protein, p53 cofactor [Source:HGNC Symbol;Acc:HGNC:28916]
**0,570113357**	2,60E-24	5,09E-23	*RRM2B*	ribonucleotide reductase regulatory TP53 inducible subunit M2B [Source:HGNC Symbol;Acc:HGNC:17296]
**0,570024012**	0,000602072	0,002382504	*GPRASP1*	G protein-coupled receptor associated sorting protein 1 [Source:HGNC Symbol;Acc:HGNC:24834]
**0,569993623**	4,90E-17	6,79E-16	*NPC2*	NPC intracellular cholesterol transporter 2 [Source:HGNC Symbol;Acc:HGNC:14537]
**0,569495007**	1,35E-23	2,53E-22	*CYBRD1*	cytochrome b reductase 1 [Source:HGNC Symbol;Acc:HGNC:20797]
**0,569109337**	8,07E-07	4,97E-06	*DUSP23*	dual specificity phosphatase 23 [Source:HGNC Symbol;Acc:HGNC:21480]

**0,568851345**	7,32E-09	5,58E-08	*KCTD12*	potassium channel tetramerization domain containing 12 [Source:HGNC Symbol;Acc:HGNC:14678]
**0,568685517**	1,01E-19	1,61E-18	*TFDP2*	transcription factor Dp-2 [Source:HGNC Symbol;Acc:HGNC:11751]
**0,568473451**	7,10E-09	5,42E-08	*ASTN2*	astrotactin 2 [Source:HGNC Symbol;Acc:HGNC:17021]
**0,568431923**	1,31E-17	1,88E-16	*ADGRG1*	adhesion G protein-coupled receptor G1 [Source:HGNC Symbol;Acc:HGNC:4512]
**0,567210223**	1,01E-11	9,84E-11	*IGBP1*	immunoglobulin binding protein 1 [Source:HGNC Symbol;Acc:HGNC:5461]
**0,566984144**	2,77E-05	0,000138716	*RNF180*	ring finger protein 180 [Source:HGNC Symbol;Acc:HGNC:27752]
**0,566968413**	6,14E-05	0,00029189	*SPTLC3*	serine palmitoyltransferase long chain base subunit 3 [Source:HGNC Symbol;Acc:HGNC:16253]
**0,566638906**	1,82E-16	2,42E-15	*ALAS1*	5'-aminolevulinate synthase 1 [Source:HGNC Symbol;Acc:HGNC:396]
**0,566606595**	1,53E-18	2,30E-17	*ADGRB2*	adhesion G protein-coupled receptor B2 [Source:HGNC Symbol;Acc:HGNC:944]
**0,566437053**	4,00E-14	4,68E-13	*PHF21A*	PHD finger protein 21A [Source:HGNC Symbol;Acc:HGNC:24156]
**0,566157759**	4,31E-27	9,37E-26	*MDM2*	MDM2 proto-oncogene [Source:HGNC Symbol;Acc:HGNC:6973]
**0,564983243**	1,80E-13	2,02E-12	*ASIC1*	acid sensing ion channel subunit 1 [Source:HGNC Symbol;Acc:HGNC:100]
**0,564684725**	0,003430207	0,011518706	*RNASET2*	ribonuclease T2 [Source:HGNC Symbol;Acc:HGNC:21686]
**0,564605161**	5,37E-18	7,87E-17	*IFI16*	interferon gamma inducible protein 16 [Source:HGNC Symbol;Acc:HGNC:5395]
**0,564029412**	3,32E-05	0,000164312	*SBK1*	SH3 domain binding kinase 1 [Source:HGNC Symbol;Acc:HGNC:17699]
**0,563388673**	3,77E-29	8,96E-28	*MT2A*	metallothionein 2A [Source:HGNC Symbol;Acc:HGNC:7406]
**0,562794872**	0,004302643	0,014123325	*PDIK1L*	PDLIM1 interacting kinase 1 like [Source:HGNC Symbol;Acc:HGNC:18981]
**0,562352227**	0,001041998	0,003927936	*YPEL2*	yippee like 2 [Source:HGNC Symbol;Acc:HGNC:18326]
**0,562024253**	1,92E-18	2,87E-17	*AMOTL1*	angiomotin like 1 [Source:HGNC Symbol;Acc:HGNC:17811]
**0,561829772**	2,27E-05	0,000114899	*AJM1*	apical junction component 1 homolog [Source:HGNC Symbol;Acc:HGNC:37284]
**0,560819433**	4,28E-33	1,18E-31	*CCNG1*	cyclin G1 [Source:HGNC Symbol;Acc:HGNC:1592]
**0,560583634**	4,70E-06	2,61E-05	*WDR91*	WD repeat domain 91 [Source:HGNC Symbol;Acc:HGNC:24997]
**0,560406818**	3,62E-07	2,31E-06	*SLC50A1*	solute carrier family 50 member 1 [Source:HGNC Symbol;Acc:HGNC:30657]
**0,560063551**	0,000141694	0,000629946	*HSD17B7*	hydroxysteroid 17-beta dehydrogenase 7 [Source:HGNC Symbol;Acc:HGNC:5215]
**0,559860373**	4,39E-31	1,12E-29	*ZFP36L1*	ZFP36 ring finger protein like 1 [Source:HGNC Symbol;Acc:HGNC:1107]
**0,559540231**	0,003229624	0,010913126	*CRABP2*	cellular retinoic acid binding protein 2 [Source:HGNC Symbol;Acc:HGNC:2339]
**0,558925157**	0,000986925	0,003735569	*ZNRF2*	zinc and ring finger 2 [Source:HGNC Symbol;Acc:HGNC:22316]
**0,558613012**	0,000337752	0,001396635	*SP4*	Sp4 transcription factor [Source:HGNC Symbol;Acc:HGNC:11209]
**0,558199129**	0,000106078	0,00048334	*NMB*	neuromedin B [Source:HGNC Symbol;Acc:HGNC:7842]
**0,558029325**	7,68E-07	4,73E-06	*IRF2*	interferon regulatory factor 2 [Source:HGNC Symbol;Acc:HGNC:6117]
**0,557602688**	2,60E-12	2,68E-11	*XPC*	XPC complex subunit, DNA damage recognition and repair factor [Source:HGNC Symbol;Acc:HGNC:12816]
**0,556830752**	0,002756986	0,009462693	*WNK3*	WNK lysine deficient protein kinase 3 [Source:HGNC Symbol;Acc:HGNC:14543]
**0,556319022**	0,008370415	0,025440295	*APOL1*	apolipoprotein L1 [Source:HGNC Symbol;Acc:HGNC:618]
**0,55615746**	2,08E-08	1,52E-07	*MAP3K12*	mitogen-activated protein kinase kinase kinase 12 [Source:HGNC Symbol;Acc:HGNC:6851]

**0,555609697**	1,59E-12	1,67E-11	*HIST2H2BE*	histone cluster 2 H2B family member e [Source:HGNC Symbol;Acc:HGNC:4760]
**0,55550406**	2,28E-13	2,54E-12	*KSR1*	kinase suppressor of ras 1 [Source:HGNC Symbol;Acc:HGNC:6465]
**0,555157597**	2,10E-09	1,68E-08	*CERS4*	ceramide synthase 4 [Source:HGNC Symbol;Acc:HGNC:23747]
**0,554692746**	2,60E-11	2,45E-10	*CYB5D2*	cytochrome b5 domain containing 2 [Source:HGNC Symbol;Acc:HGNC:28471]
**0,554105942**	4,45E-10	3,77E-09	*NOVA2*	NOVA alternative splicing regulator 2 [Source:HGNC Symbol;Acc:HGNC:7887]
**0,553957056**	7,06E-16	9,08E-15	*PARD3*	par-3 family cell polarity regulator [Source:HGNC Symbol;Acc:HGNC:16051]
**0,553706277**	3,07E-12	3,15E-11	*DDB2*	damage specific DNA binding protein 2 [Source:HGNC Symbol;Acc:HGNC:2718]
**0,553331343**	1,34E-37	4,23E-36	*FTH1*	ferritin heavy chain 1 [Source:HGNC Symbol;Acc:HGNC:3976]
**0,55317871**	6,00E-20	9,66E-19	*DUSP4*	dual specificity phosphatase 4 [Source:HGNC Symbol;Acc:HGNC:3070]
**0,552797175**	2,51E-16	3,33E-15	*RASSF8*	Ras association domain family member 8 [Source:HGNC Symbol;Acc:HGNC:13232]
**0,552421556**	4,51E-11	4,15E-10	*PRRX1*	paired related homeobox 1 [Source:HGNC Symbol;Acc:HGNC:9142]
**0,551593437**	0,003172596	0,010746621	*PUS10*	pseudouridylate synthase 10 [Source:HGNC Symbol;Acc:HGNC:26505]
**0,551358196**	0,000301135	0,001256468	*GRAMD1B*	GRAM domain containing 1B [Source:HGNC Symbol;Acc:HGNC:29214]
**0,551201276**	0,000894319	0,003421082	*QPRT*	quinolinate phosphoribosyltransferase [Source:HGNC Symbol;Acc:HGNC:9755]
**0,548579188**	0,000942741	0,003587223	*APOBEC3F*	apolipoprotein B mRNA editing enzyme catalytic subunit 3F [Source:HGNC Symbol;Acc:HGNC:17356]
**0,548417453**	1,99E-13	2,23E-12	*CCNG2*	cyclin G2 [Source:HGNC Symbol;Acc:HGNC:1593]
**0,547116489**	3,37E-16	4,42E-15	*NLRP1*	NLR family pyrin domain containing 1 [Source:HGNC Symbol;Acc:HGNC:14374]
**0,547015181**	8,02E-05	0,000373874	*EGFL7*	EGF like domain multiple 7 [Source:HGNC Symbol;Acc:HGNC:20594]
**0,546497953**	1,43E-26	3,05E-25	*CPE*	carboxypeptidase E [Source:HGNC Symbol;Acc:HGNC:2303]
**0,545397098**	1,54E-05	7,97E-05	*DYNC2H1*	dynein cytoplasmic 2 heavy chain 1 [Source:HGNC Symbol;Acc:HGNC:2962]
**0,544605758**	4,29E-08	3,04E-07	*PCMTD1*	protein-L-isoaspartate (D-aspartate) O-methyltransferase domain containing 1 [Source:HGNC Symbol;Acc:HGNC:30483]
**0,5443925**	2,33E-17	3,30E-16	*PRXL2A*	peroxiredoxin like 2A [Source:HGNC Symbol;Acc:HGNC:28651]
**0,544071301**	1,39E-09	1,13E-08	*TOX*	thymocyte selection associated high mobility group box [Source:HGNC Symbol;Acc:HGNC:18988]
**0,543906381**	1,09E-21	1,89E-20	*HIPK2*	homeodomain interacting protein kinase 2 [Source:HGNC Symbol;Acc:HGNC:14402]
**0,543577275**	2,11E-06	1,23E-05	*CIT*	citron rho-interacting serine/threonine kinase [Source:HGNC Symbol;Acc:HGNC:1985]
**0,543544323**	1,33E-05	6,95E-05	*CXADR*	CXADR, Ig-like cell adhesion molecule [Source:HGNC Symbol;Acc:HGNC:2559]
**0,543305902**	2,33E-05	0,000117807	*LRP1*	LDL receptor related protein 1 [Source:HGNC Symbol;Acc:HGNC:6692]
**0,542802686**	4,87E-10	4,12E-09	*KAT2B*	lysine acetyltransferase 2B [Source:HGNC Symbol;Acc:HGNC:8638]
**0,542594169**	2,84E-11	2,67E-10	*ECH1*	enoyl-CoA hydratase 1 [Source:HGNC Symbol;Acc:HGNC:3149]
**0,541383487**	3,10E-06	1,77E-05	*ARL4A*	ADP ribosylation factor like GTPase 4A [Source:HGNC Symbol;Acc:HGNC:695]
**0,541342545**	8,96E-10	7,39E-09	*HES1*	hes family bHLH transcription factor 1 [Source:HGNC Symbol;Acc:HGNC:5192]

**0,54095389**	2,47E-12	2,55E-11	*RNF220*	ring finger protein 220 [Source:HGNC Symbol;Acc:HGNC:25552]
**0,540949806**	6,93E-07	4,29E-06	*SLC26A11*	solute carrier family 26 member 11 [Source:HGNC Symbol;Acc:HGNC:14471]
**0,540886788**	0,000698958	0,002737432	*PIM2*	Pim-2 proto-oncogene, serine/threonine kinase [Source:HGNC Symbol;Acc:HGNC:8987]
**0,540838524**	1,07E-07	7,24E-07	*CIR1*	corepressor interacting with RBPJ, 1 [Source:HGNC Symbol;Acc:HGNC:24217]
**0,540604914**	0,000278917	0,001170811	*ATXN7L2*	ataxin 7 like 2 [Source:HGNC Symbol;Acc:HGNC:28713]
**0,540311288**	3,06E-30	7,57E-29	*TNPO1*	transportin 1 [Source:HGNC Symbol;Acc:HGNC:6401]
**0,539456486**	7,87E-13	8,48E-12	*MRPS30*	mitochondrial ribosomal protein S30 [Source:HGNC Symbol;Acc:HGNC:8769]
**0,539391962**	0,003420641	0,01149056	*THAP8*	THAP domain containing 8 [Source:HGNC Symbol;Acc:HGNC:23191]
**0,539373729**	5,80E-05	0,000277181	*EPHB1*	EPH receptor B1 [Source:HGNC Symbol;Acc:HGNC:3392]
**0,539276049**	1,88E-06	1,11E-05	*USP54*	ubiquitin specific peptidase 54 [Source:HGNC Symbol;Acc:HGNC:23513]
**0,539217016**	6,06E-14	7,00E-13	*PCYT2*	phosphate cytidylyltransferase 2, ethanolamine [Source:HGNC Symbol;Acc:HGNC:8756]
**0,53888369**	5,72E-13	6,23E-12	*CD109*	CD109 molecule [Source:HGNC Symbol;Acc:HGNC:21685]
**0,538710909**	0,000241482	0,001025202	*GHDC*	GH3 domain containing [Source:HGNC Symbol;Acc:HGNC:24438]
**0,537633954**	2,93E-08	2,11E-07	*PLXNA4*	plexin A4 [Source:HGNC Symbol;Acc:HGNC:9102]
**0,537149304**	0,000858324	0,003298343	*LRRC37B*	leucine rich repeat containing 37B [Source:HGNC Symbol;Acc:HGNC:29070]
**0,537040638**	2,90E-16	3,82E-15	*DBI*	diazepam binding inhibitor, acyl-CoA binding protein [Source:HGNC Symbol;Acc:HGNC:2690]
**0,536994165**	5,87E-05	0,000279974	*RPP25L*	ribonuclease P/MRP subunit p25 like [Source:HGNC Symbol;Acc:HGNC:19909]
**0,536866468**	0,001804588	0,006450514	*MGST2*	microsomal glutathione S-transferase 2 [Source:HGNC Symbol;Acc:HGNC:7063]
**0,536736069**	3,64E-05	0,000179007	*KMT5C*	lysine methyltransferase 5C [Source:HGNC Symbol;Acc:HGNC:28405]
**0,536707158**	1,01E-05	5,36E-05	*BCL7A*	BCL tumor suppressor 7A [Source:HGNC Symbol;Acc:HGNC:1004]
**0,536522924**	5,43E-10	4,57E-09	*LRBA*	LPS responsive beige-like anchor protein [Source:HGNC Symbol;Acc:HGNC:1742]
**0,53579842**	0,0010113	0,003818149	*RLBP1*	retinaldehyde binding protein 1 [Source:HGNC Symbol;Acc:HGNC:10024]
**0,535762683**	1,09E-24	2,15E-23	*TBC1D16*	TBC1 domain family member 16 [Source:HGNC Symbol;Acc:HGNC:28356]
**0,535192371**	3,66E-16	4,77E-15	*SEC14L2*	SEC14 like lipid binding 2 [Source:HGNC Symbol;Acc:HGNC:10699]
**0,534975631**	5,72E-08	4,00E-07	*MTURN*	maturin, neural progenitor differentiation regulator homolog [Source:HGNC Symbol;Acc:HGNC:25457]
**0,53484363**	2,62E-14	3,09E-13	*CAT*	catalase [Source:HGNC Symbol;Acc:HGNC:1516]
**0,534745133**	0,007769156	0,023825576	*AVIL*	advillin [Source:HGNC Symbol;Acc:HGNC:14188]
**0,534595622**	7,34E-11	6,63E-10	*CPNE2*	copine 2 [Source:HGNC Symbol;Acc:HGNC:2315]
**0,53450438**	0,003042511	0,010354782	*PHYHIP*	phytanoyl-CoA 2-hydroxylase interacting protein [Source:HGNC Symbol;Acc:HGNC:16865]
**0,53429957**	1,03E-11	9,97E-11	*VPS11*	VPS11, CORVET/HOPS core subunit [Source:HGNC Symbol;Acc:HGNC:14583]
**0,534282457**	4,33E-06	2,42E-05	*ZBTB12*	zinc finger and BTB domain containing 12 [Source:HGNC Symbol;Acc:HGNC:19066]
**0,533793132**	2,42E-22	4,30E-21	*CLIP3*	CAP-Gly domain containing linker protein 3 [Source:HGNC Symbol;Acc:HGNC:24314]
**0,533441297**	0,003455371	0,011587166	*SNCA*	synuclein alpha [Source:HGNC Symbol;Acc:HGNC:11138]
**0,532863081**	2,05E-11	1,95E-10	*WDR81*	WD repeat domain 81 [Source:HGNC Symbol;Acc:HGNC:26600]
**0,532116944**	2,70E-05	0,000135576	*ROM1*	retinal outer segment membrane protein 1 [Source:HGNC Symbol;Acc:HGNC:10254]

**0,531698575**	4,87E-09	3,78E-08	*KIAA1549L*	KIAA1549 like [Source:HGNC Symbol;Acc:HGNC:24836]
**0,531690725**	7,24E-05	0,000339455	*NUDT14*	nudix hydrolase 14 [Source:HGNC Symbol;Acc:HGNC:20141]
**0,531612921**	4,52E-09	3,52E-08	*SLCO3A1*	solute carrier organic anion transporter family member 3A1 [Source:HGNC Symbol;Acc:HGNC:10952]
**0,531251375**	7,07E-09	5,41E-08	*MBP*	myelin basic protein [Source:HGNC Symbol;Acc:HGNC:6925]
**0,531070938**	0,0087352	0,026400138	*AC073111,5*	novel zinc finger protein
**0,530949189**	1,15E-15	1,46E-14	*CES2*	carboxylesterase 2 [Source:HGNC Symbol;Acc:HGNC:1864]
**0,530033844**	2,81E-09	2,23E-08	*DNAJC4*	DnaJ heat shock protein family (Hsp40) member C4 [Source:HGNC Symbol;Acc:HGNC:5271]
**0,529955003**	5,45E-11	4,97E-10	*SLC44A1*	solute carrier family 44 member 1 [Source:HGNC Symbol;Acc:HGNC:18798]
**0,529667846**	5,14E-20	8,29E-19	*NRP1*	neuropilin 1 [Source:HGNC Symbol;Acc:HGNC:8004]
**0,529322454**	0,000106844	0,000486489	*POC1B*	POC1 centriolar protein B [Source:HGNC Symbol;Acc:HGNC:30836]
**0,528667151**	1,47E-05	7,63E-05	*MARK1*	microtubule affinity regulating kinase 1 [Source:HGNC Symbol;Acc:HGNC:6896]
**0,527847924**	2,04E-34	5,84E-33	*FADS2*	fatty acid desaturase 2 [Source:HGNC Symbol;Acc:HGNC:3575]
**0,527577446**	0,000197527	0,000854102	*POLM*	DNA polymerase mu [Source:HGNC Symbol;Acc:HGNC:9185]
**0,527552473**	1,47E-09	1,19E-08	*HOMER2*	homer scaffold protein 2 [Source:HGNC Symbol;Acc:HGNC:17513]
**0,527476272**	2,06E-06	1,20E-05	*PFDN4*	prefoldin subunit 4 [Source:HGNC Symbol;Acc:HGNC:8868]
**0,527228296**	0,004557215	0,014870911	*RNF157*	ring finger protein 157 [Source:HGNC Symbol;Acc:HGNC:29402]
**0,527080056**	0,013884863	0,039531933	*CHRM3*	cholinergic receptor muscarinic 3 [Source:HGNC Symbol;Acc:HGNC:1952]
**0,526713681**	1,56E-11	1,50E-10	*NFATC4*	nuclear factor of activated T cells 4 [Source:HGNC Symbol;Acc:HGNC:7778]
**0,526701003**	0,005919184	0,018790722	*UBB*	ubiquitin B [Source:HGNC Symbol;Acc:HGNC:12463]
**0,526394546**	4,36E-25	8,77E-24	*S100A6*	S100 calcium binding protein A6 [Source:HGNC Symbol;Acc:HGNC:10496]
**0,5263645**	4,08E-08	2,90E-07	*ATG14*	autophagy related 14 [Source:HGNC Symbol;Acc:HGNC:19962]
**0,526124676**	2,49E-13	2,77E-12	*TMX4*	thioredoxin related transmembrane protein 4 [Source:HGNC Symbol;Acc:HGNC:25237]
**0,525954166**	0,0029199	0,009983012	*CDC42BPG*	CDC42 binding protein kinase gamma [Source:HGNC Symbol;Acc:HGNC:29829]
**0,525925948**	2,68E-08	1,93E-07	*SLC2A3*	solute carrier family 2 member 3 [Source:HGNC Symbol;Acc:HGNC:11007]
**0,525662038**	2,85E-13	3,16E-12	*THSD4*	thrombospondin type 1 domain containing 4 [Source:HGNC Symbol;Acc:HGNC:25835]
**0,524698508**	3,22E-08	2,31E-07	*MVK*	mevalonate kinase [Source:HGNC Symbol;Acc:HGNC:7530]
**0,524656464**	1,00E-05	5,33E-05	*FAM76B*	family with sequence similarity 76 member B [Source:HGNC Symbol;Acc:HGNC:28492]
**0,524159697**	3,01E-09	2,38E-08	*KMT2C*	lysine methyltransferase 2C [Source:HGNC Symbol;Acc:HGNC:13726]
**0,523869941**	0,003134389	0,010637628	*SCN2B*	sodium voltage-gated channel beta subunit 2 [Source:HGNC Symbol;Acc:HGNC:10589]
**0,523808955**	3,24E-09	2,55E-08	*SNX29*	sorting nexin 29 [Source:HGNC Symbol;Acc:HGNC:30542]
**0,523425821**	0,00025325	0,001071881	*MRNIP*	MRN complex interacting protein [Source:HGNC Symbol;Acc:HGNC:30817]
**0,523218045**	5,06E-23	9,26E-22	*CLIP2*	CAP-Gly domain containing linker protein 2 [Source:HGNC Symbol;Acc:HGNC:2586]
**0,523007885**	0,000161728	0,000711221	*LRRC75B*	leucine rich repeat containing 75B [Source:HGNC Symbol;Acc:HGNC:33155]

**0,522899823**	3,02E-10	2,60E-09	*OPHN1*	oligophrenin 1 [Source:HGNC Symbol;Acc:HGNC:8148]
**0,522792314**	7,76E-34	2,18E-32	*CDKN1A*	cyclin dependent kinase inhibitor 1A [Source:HGNC Symbol;Acc:HGNC:1784]
**0,522745388**	8,88E-06	4,77E-05	*BICRAL*	BRD4 interacting chromatin remodeling complex associated protein like [Source:HGNC Symbol;Acc:HGNC:21111]
**0,522672679**	7,64E-12	7,49E-11	*MBOAT2*	membrane bound O-acyltransferase domain containing 2 [Source:HGNC Symbol;Acc:HGNC:25193]
**0,521714413**	2,84E-10	2,45E-09	*MAGI1*	membrane associated guanylate kinase, WW and PDZ domain containing 1 [Source:HGNC Symbol;Acc:HGNC:946]
**0,521551688**	2,88E-13	3,19E-12	*C8orf82*	chromosome 8 open reading frame 82 [Source:HGNC Symbol;Acc:HGNC:33826]
**0,520562828**	1,60E-07	1,07E-06	*FIG4*	FIG4 phosphoinositide 5-phosphatase [Source:HGNC Symbol;Acc:HGNC:16873]
**0,519435527**	4,47E-10	3,79E-09	*IFT140*	intraflagellar transport 140 [Source:HGNC Symbol;Acc:HGNC:29077]
**0,51939763**	0,003968662	0,013115778	*ATXN7L1*	ataxin 7 like 1 [Source:HGNC Symbol;Acc:HGNC:22210]
**0,519096053**	2,56E-06	1,47E-05	*S100A13*	S100 calcium binding protein A13 [Source:HGNC Symbol;Acc:HGNC:10490]
**0,518853594**	7,20E-20	1,15E-18	*SUB1*	SUB1 homolog, transcriptional regulator [Source:HGNC Symbol;Acc:HGNC:19985]
**0,518759545**	4,57E-07	2,89E-06	*ZNF581*	zinc finger protein 581 [Source:HGNC Symbol;Acc:HGNC:25017]
**0,518718513**	0,013139951	0,037676402	*TMEM256*	transmembrane protein 256 [Source:HGNC Symbol;Acc:HGNC:28618]
**0,518571729**	0,001993071	0,007065682	*SLC25A45*	solute carrier family 25 member 45 [Source:HGNC Symbol;Acc:HGNC:27442]
**0,518370618**	6,96E-21	1,17E-19	*GLUD1*	glutamate dehydrogenase 1 [Source:HGNC Symbol;Acc:HGNC:4335]
**0,517981337**	0,000255837	0,001080945	*VPS37D*	VPS37D, ESCRT-I subunit [Source:HGNC Symbol;Acc:HGNC:18287]
**0,517540703**	0,000941167	0,00358334	*PTK2B*	protein tyrosine kinase 2 beta [Source:HGNC Symbol;Acc:HGNC:9612]
**0,517404658**	1,79E-31	4,63E-30	*DPYSL3*	dihydropyrimidinase like 3 [Source:HGNC Symbol;Acc:HGNC:3015]
**0,517282676**	1,12E-11	1,09E-10	*SALL2*	spalt like transcription factor 2 [Source:HGNC Symbol;Acc:HGNC:10526]
**0,516744119**	3,93E-05	0,000192523	*NMRK1*	nicotinamide riboside kinase 1 [Source:HGNC Symbol;Acc:HGNC:26057]
**0,516595746**	0,009857262	0,029329965	*KAZALD1*	Kazal type serine peptidase inhibitor domain 1 [Source:HGNC Symbol;Acc:HGNC:25460]
**0,516573844**	3,26E-05	0,000161658	*ANKS1B*	ankyrin repeat and sterile alpha motif domain containing 1B [Source:HGNC Symbol;Acc:HGNC:24600]
**0,516327122**	0,012688449	0,036554638	*TMEM79*	transmembrane protein 79 [Source:HGNC Symbol;Acc:HGNC:28196]
**0,515568399**	3,99E-07	2,54E-06	*TRAF3IP2*	TRAF3 interacting protein 2 [Source:HGNC Symbol;Acc:HGNC:1343]
**0,515388228**	0,002753416	0,009457132	*GYG2*	glycogenin 2 [Source:HGNC Symbol;Acc:HGNC:4700]
**0,515328243**	7,59E-10	6,30E-09	*PDGFD*	platelet derived growth factor D [Source:HGNC Symbol;Acc:HGNC:30620]
**0,514767714**	0,00094187	0,00358461	*SRXN1*	sulfiredoxin 1 [Source:HGNC Symbol;Acc:HGNC:16132]
**0,514705614**	2,42E-31	6,26E-30	*SREBF2*	sterol regulatory element binding transcription factor 2 [Source:HGNC Symbol;Acc:HGNC:11290]
**0,514486174**	0,014141348	0,040150255	*SULT1C4*	sulfotransferase family 1C member 4 [Source:HGNC Symbol;Acc:HGNC:11457]
**0,513587445**	0,002569044	0,008880454	*LRRC1*	leucine rich repeat containing 1 [Source:HGNC Symbol;Acc:HGNC:14307]
**0,5133308**	5,33E-11	4,88E-10	*MRPL10*	mitochondrial ribosomal protein L10 [Source:HGNC Symbol;Acc:HGNC:14055]
**0,51316999**	2,93E-12	3,01E-11	*UBTD1*	ubiquitin domain containing 1 [Source:HGNC Symbol;Acc:HGNC:25683]

**0,513160345**	4,32E-12	4,34E-11	*DCAF11*	DDB1 and CUL4 associated factor 11 [Source:HGNC Symbol;Acc:HGNC:20258]
**0,512611734**	1,72E-11	1,64E-10	*GATAD2B*	GATA zinc finger domain containing 2B [Source:HGNC Symbol;Acc:HGNC:30778]
**0,51211545**	0,000176543	0,000771098	*LCA5*	LCA5, lebercilin [Source:HGNC Symbol;Acc:HGNC:31923]
**0,511930375**	2,12E-20	3,48E-19	*REEP5*	receptor accessory protein 5 [Source:HGNC Symbol;Acc:HGNC:30077]
**0,511892812**	1,88E-17	2,68E-16	*FRMD5*	FERM domain containing 5 [Source:HGNC Symbol;Acc:HGNC:28214]
**0,511670609**	1,00E-11	9,74E-11	*CDIP1*	cell death inducing p53 target 1 [Source:HGNC Symbol;Acc:HGNC:13234]
**0,511391644**	0,003707637	0,012352002	*C6orf136*	chromosome 6 open reading frame 136 [Source:HGNC Symbol;Acc:HGNC:21301]
**0,511265034**	0,002945622	0,01006032	*MAST1*	microtubule associated serine/threonine kinase 1 [Source:HGNC Symbol;Acc:HGNC:19034]
**0,511107121**	0,00044667	0,001810082	*SPACA9*	sperm acrosome associated 9 [Source:HGNC Symbol;Acc:HGNC:1367]
**0,510257028**	0,00164912	0,005948481	*ZNF79*	zinc finger protein 79 [Source:HGNC Symbol;Acc:HGNC:13153]
**0,509691688**	0,007935727	0,024259722	*TBC1D3L*	TBC1 domain family member 3L [Source:HGNC Symbol;Acc:HGNC:51246]
**0,509547458**	4,21E-05	0,000205629	*ZNF385A*	zinc finger protein 385A [Source:HGNC Symbol;Acc:HGNC:17521]
**0,509450369**	8,07E-06	4,36E-05	*MBTD1*	mbt domain containing 1 [Source:HGNC Symbol;Acc:HGNC:19866]
**0,509235712**	4,85E-08	3,42E-07	*PDK2*	pyruvate dehydrogenase kinase 2 [Source:HGNC Symbol;Acc:HGNC:8810]
**0,509028734**	0,000276025	0,001159425	*ETNK2*	ethanolamine kinase 2 [Source:HGNC Symbol;Acc:HGNC:25575]
**0,508994354**	7,55E-06	4,09E-05	*CADM3*	cell adhesion molecule 3 [Source:HGNC Symbol;Acc:HGNC:17601]
**0,50858861**	0,000691009	0,002709036	*PLPP6*	phospholipid phosphatase 6 [Source:HGNC Symbol;Acc:HGNC:23682]
**0,508549144**	2,89E-07	1,87E-06	*OLFML3*	olfactomedin like 3 [Source:HGNC Symbol;Acc:HGNC:24956]
**0,508133402**	9,45E-14	1,08E-12	*CHD2*	chromodomain helicase DNA binding protein 2 [Source:HGNC Symbol;Acc:HGNC:1917]
**0,507254886**	0,002906569	0,009939185	*DDIT3*	DNA damage inducible transcript 3 [Source:HGNC Symbol;Acc:HGNC:2726]
**0,506805671**	1,17E-12	1,24E-11	*MAP1A*	microtubule associated protein 1A [Source:HGNC Symbol;Acc:HGNC:6835]
**0,506752277**	5,55E-05	0,000265854	*PBLD*	phenazine biosynthesis like protein domain containing [Source:HGNC Symbol;Acc:HGNC:23301]
**0,506458799**	3,47E-15	4,30E-14	*CHP1*	calcineurin like EF-hand protein 1 [Source:HGNC Symbol;Acc:HGNC:17433]
**0,506410166**	3,35E-05	0,000165432	*ADAMTS13*	ADAM metallopeptidase with thrombospondin type 1 motif 13 [Source:HGNC Symbol;Acc:HGNC:1366]
**0,505885988**	1,60E-13	1,80E-12	*COX5B*	cytochrome c oxidase subunit 5B [Source:HGNC Symbol;Acc:HGNC:2269]
**0,505414735**	3,57E-05	0,000175722	*R3HDM2*	R3H domain containing 2 [Source:HGNC Symbol;Acc:HGNC:29167]
**0,50539566**	0,006965799	0,021638913	*ZNF14*	zinc finger protein 14 [Source:HGNC Symbol;Acc:HGNC:12924]
**0,505300159**	2,30E-09	1,83E-08	*ZNF770*	zinc finger protein 770 [Source:HGNC Symbol;Acc:HGNC:26061]
**0,504648273**	7,12E-07	4,40E-06	*ATP7A*	ATPase copper transporting alpha [Source:HGNC Symbol;Acc:HGNC:869]
**0,503806766**	4,15E-31	1,06E-29	*LSS*	lanosterol synthase [Source:HGNC Symbol;Acc:HGNC:6708]
**0,502463881**	4,40E-15	5,42E-14	*BBX*	BBX, HMG-box containing [Source:HGNC Symbol;Acc:HGNC:14422]

**0,502115793**	9,13E-06	4,89E-05	*SPIN3*	spindlin family member 3 [Source:HGNC Symbol;Acc:HGNC:27272]
**0,501652501**	2,18E-06	1,27E-05	*RICTOR*	RPTOR independent companion of MTOR complex 2 [Source:HGNC Symbol;Acc:HGNC:28611]
**0,501523615**	0,000219207	0,000938023	*ZNF75D*	zinc finger protein 75D [Source:HGNC Symbol;Acc:HGNC:13145]
**0,500836591**	0,000584642	0,002318259	*ACBD4*	acyl-CoA binding domain containing 4 [Source:HGNC Symbol;Acc:HGNC:23337]
**0,500682171**	4,11E-30	1,01E-28	*APLP2*	amyloid beta precursor like protein 2 [Source:HGNC Symbol;Acc:HGNC:598]
**0,500603869**	9,65E-05	0,000443712	*SIX5*	SIX homeobox 5 [Source:HGNC Symbol;Acc:HGNC:10891]
**0,500217906**	0,001590179	0,005750842	*ZNF280D*	zinc finger protein 280D [Source:HGNC Symbol;Acc:HGNC:25953]
**0,499115651**	6,99E-20	1,12E-18	*SOX12*	SRY-box 12 [Source:HGNC Symbol;Acc:HGNC:11198]
**0,497946842**	0,001668359	0,006007825	*MSX1*	msh homeobox 1 [Source:HGNC Symbol;Acc:HGNC:7391]
**0,497161355**	2,09E-16	2,78E-15	*APBA2*	amyloid beta precursor protein binding family A member 2 [Source:HGNC Symbol;Acc:HGNC:579]
**0,496594054**	3,84E-19	5,98E-18	*WDR26*	WD repeat domain 26 [Source:HGNC Symbol;Acc:HGNC:21208]
**0,496464535**	0,000199387	0,000860994	*IKBKE*	inhibitor of nuclear factor kappa B kinase subunit epsilon [Source:HGNC Symbol;Acc:HGNC:14552]
**0,495920116**	0,000867607	0,003328742	*NTRK2*	neurotrophic receptor tyrosine kinase 2 [Source:HGNC Symbol;Acc:HGNC:8032]
**0,495710305**	5,99E-13	6,51E-12	*TMEM132D*	transmembrane protein 132D [Source:HGNC Symbol;Acc:HGNC:29411]
**0,495701529**	0,006248482	0,019716755	*PLEKHA6*	pleckstrin homology domain containing A6 [Source:HGNC Symbol;Acc:HGNC:17053]
**0,495683347**	8,11E-05	0,000376987	*TRIM62*	tripartite motif containing 62 [Source:HGNC Symbol;Acc:HGNC:25574]
**0,495601087**	5,05E-06	2,80E-05	*IL11*	interleukin 11 [Source:HGNC Symbol;Acc:HGNC:5966]
**0,495311686**	9,64E-05	0,00044345	*AR*	androgen receptor [Source:HGNC Symbol;Acc:HGNC:644]
**0,495061533**	8,72E-15	1,06E-13	*RTL8A*	retrotransposon Gag like 8A [Source:HGNC Symbol;Acc:HGNC:24514]
**0,494862548**	3,44E-17	4,81E-16	*TCF4*	transcription factor 4 [Source:HGNC Symbol;Acc:HGNC:11634]
**0,4947601**	0,000194236	0,000841937	*HAS3*	hyaluronan synthase 3 [Source:HGNC Symbol;Acc:HGNC:4820]
**0,49391448**	0,004991331	0,016126827	*HOXB6*	homeobox B6 [Source:HGNC Symbol;Acc:HGNC:5117]
**0,493883327**	7,75E-15	9,41E-14	*OXA1L*	OXA1L, mitochondrial inner membrane protein [Source:HGNC Symbol;Acc:HGNC:8526]
**0,492013837**	8,28E-08	5,70E-07	*ATE1*	arginyltransferase 1 [Source:HGNC Symbol;Acc:HGNC:782]
**0,491736243**	0,0149194	0,041984611	*AKAP6*	A-kinase anchoring protein 6 [Source:HGNC Symbol;Acc:HGNC:376]
**0,491559806**	2,04E-09	1,63E-08	*PHF1*	PHD finger protein 1 [Source:HGNC Symbol;Acc:HGNC:8919]
**0,491146122**	0,000397922	0,001629494	*PCDHGA6*	protocadherin gamma subfamily A, 6 [Source:HGNC Symbol;Acc:HGNC:8704]
**0,490866319**	0,00208716	0,007374986	*GRIP1*	glutamate receptor interacting protein 1 [Source:HGNC Symbol;Acc:HGNC:18708]
**0,490804997**	2,15E-07	1,41E-06	*IFT57*	intraflagellar transport 57 [Source:HGNC Symbol;Acc:HGNC:17367]
**0,49015663**	1,56E-06	9,31E-06	*GPRC5C*	G protein-coupled receptor class C group 5 member C [Source:HGNC Symbol;Acc:HGNC:13309]
**0,489806941**	1,80E-05	9,25E-05	*LYNX1*	Ly6/neurotoxin 1 [Source:HGNC Symbol;Acc:HGNC:29604]
**0,489802222**	3,40E-13	3,75E-12	*NDRG4*	NDRG family member 4 [Source:HGNC Symbol;Acc:HGNC:14466]

**0,48953642**	6,78E-07	4,20E-06	*TOX2*	TOX high mobility group box family member 2 [Source:HGNC Symbol;Acc:HGNC:16095]
**0,488785775**	1,02E-08	7,70E-08	*HMCES*	5-hydroxymethylcytosine binding, ES cell specific [Source:HGNC Symbol;Acc:HGNC:24446]
**0,488632435**	1,60E-08	1,19E-07	*ZNF740*	zinc finger protein 740 [Source:HGNC Symbol;Acc:HGNC:27465]
**0,488366001**	0,006169673	0,019496607	*SYT14*	synaptotagmin 14 [Source:HGNC Symbol;Acc:HGNC:23143]
**0,488304084**	0,000103664	0,000473453	*POLD4*	DNA polymerase delta 4, accessory subunit [Source:HGNC Symbol;Acc:HGNC:14106]
**0,488301184**	5,59E-07	3,50E-06	*FBXW7*	F-box and WD repeat domain containing 7 [Source:HGNC Symbol;Acc:HGNC:16712]
**0,488233344**	5,78E-07	3,61E-06	*LMBR1L*	limb development membrane protein 1 like [Source:HGNC Symbol;Acc:HGNC:18268]
**0,48794787**	1,50E-12	1,58E-11	*GSS*	glutathione synthetase [Source:HGNC Symbol;Acc:HGNC:4624]
**0,487831525**	3,46E-12	3,51E-11	*PDZD8*	PDZ domain containing 8 [Source:HGNC Symbol;Acc:HGNC:26974]
**0,487774759**	2,59E-07	1,69E-06	*NATD1*	N-acetyltransferase domain containing 1 [Source:HGNC Symbol;Acc:HGNC:30770]
**0,487749334**	2,14E-10	1,87E-09	*TSC22D3*	TSC22 domain family member 3 [Source:HGNC Symbol;Acc:HGNC:3051]
**0,487086734**	0,000844184	0,003251732	*CHIC1*	cysteine rich hydrophobic domain 1 [Source:HGNC Symbol;Acc:HGNC:1934]
**0,486690533**	1,06E-11	1,02E-10	*WDR45*	WD repeat domain 45 [Source:HGNC Symbol;Acc:HGNC:28912]
**0,486481286**	0,001102241	0,004131754	*FAM155A*	family with sequence similarity 155 member A [Source:HGNC Symbol;Acc:HGNC:33877]
**0,486307634**	7,28E-27	1,57E-25	*PTPRS*	protein tyrosine phosphatase, receptor type S [Source:HGNC Symbol;Acc:HGNC:9681]
**0,486187726**	1,68E-06	9,93E-06	*ALDOC*	aldolase, fructose-bisphosphate C [Source:HGNC Symbol;Acc:HGNC:418]
**0,48546538**	1,10E-12	1,17E-11	*RGL1*	ral guanine nucleotide dissociation stimulator like 1 [Source:HGNC Symbol;Acc:HGNC:30281]
**0,484990419**	0,000833031	0,003213237	*SMPDL3A*	sphingomyelin phosphodiesterase acid like 3A [Source:HGNC Symbol;Acc:HGNC:17389]
**0,484569409**	6,38E-14	7,36E-13	*PTPN12*	protein tyrosine phosphatase, non-receptor type 12 [Source:HGNC Symbol;Acc:HGNC:9645]
**0,484504124**	0,000357711	0,001473518	*SVIP*	small VCP interacting protein [Source:HGNC Symbol;Acc:HGNC:25238]
**0,48438622**	0,000142863	0,000634709	*STARD9*	StAR related lipid transfer domain containing 9 [Source:HGNC Symbol;Acc:HGNC:19162]
**0,483890993**	9,92E-12	9,65E-11	*MAT2B*	methionine adenosyltransferase 2B [Source:HGNC Symbol;Acc:HGNC:6905]
**0,483820865**	0,000185007	0,000804446	*ACCS*	1-aminocyclopropane-1-carboxylate synthase homolog (inactive) [Source:HGNC Symbol;Acc:HGNC:23989]
**0,483661024**	0,000127869	0,000574802	*OLFML2B*	olfactomedin like 2B [Source:HGNC Symbol;Acc:HGNC:24558]
**0,483508716**	4,54E-05	0,000220289	*TCEAL1*	transcription elongation factor A like 1 [Source:HGNC Symbol;Acc:HGNC:11616]
**0,483358565**	1,28E-15	1,62E-14	*PLEKHG1*	pleckstrin homology and RhoGEF domain containing G1 [Source:HGNC Symbol;Acc:HGNC:20884]
**0,481889898**	0,002814385	0,009646052	*NRG2*	neuregulin 2 [Source:HGNC Symbol;Acc:HGNC:7998]
**0,481838596**	0,00089317	0,003418291	*POLR3GL*	RNA polymerase III subunit G like [Source:HGNC Symbol;Acc:HGNC:28466]
**0,481786961**	4,73E-05	0,000228573	*MMAB*	methylmalonic aciduria (cobalamin deficiency) cblB type [Source:HGNC Symbol;Acc:HGNC:19331]
**0,481738412**	0,00127174	0,004704419	*SHROOM4*	shroom family member 4 [Source:HGNC Symbol;Acc:HGNC:29215]
**0,481218886**	5,43E-11	4,96E-10	*TLK1*	tousled like kinase 1 [Source:HGNC Symbol;Acc:HGNC:11841]

**0,480886724**	2,99E-09	2,36E-08	*ACAT1*	acetyl-CoA acetyltransferase 1 [Source:HGNC Symbol;Acc:HGNC:93]
**0,480633706**	2,90E-28	6,60E-27	*CRTAP*	cartilage associated protein [Source:HGNC Symbol;Acc:HGNC:2379]
**0,480574777**	1,18E-20	1,95E-19	*HLA-A*	major histocompatibility complex, class I, A [Source:HGNC Symbol;Acc:HGNC:4931]
**0,480484962**	0,000663444	0,002609931	*ERMP1*	endoplasmic reticulum metallopeptidase 1 [Source:HGNC Symbol;Acc:HGNC:23703]
**0,480401096**	0,008665715	0,026239174	*TSTD1*	thiosulfate sulfurtransferase like domain containing 1 [Source:HGNC Symbol;Acc:HGNC:35410]
**0,479947442**	2,39E-10	2,08E-09	*SLU7*	SLU7 homolog, splicing factor [Source:HGNC Symbol;Acc:HGNC:16939]
**0,479721074**	0,00092531	0,003529892	*THNSL2*	threonine synthase like 2 [Source:HGNC Symbol;Acc:HGNC:25602]
**0,478490715**	0,000233073	0,000992762	*SNX25*	sorting nexin 25 [Source:HGNC Symbol;Acc:HGNC:21883]
**0,47828023**	0,007908456	0,024191599	*FUT10*	fucosyltransferase 10 [Source:HGNC Symbol;Acc:HGNC:19234]
**0,477997322**	8,69E-05	0,000402598	*ATXN3*	ataxin 3 [Source:HGNC Symbol;Acc:HGNC:7106]
**0,47740768**	0,000705242	0,002758703	*ATG16L2*	autophagy related 16 like 2 [Source:HGNC Symbol;Acc:HGNC:25464]
**0,477207892**	6,35E-06	3,48E-05	*ITPR3*	inositol 1,4,5-trisphosphate receptor type 3 [Source:HGNC Symbol;Acc:HGNC:6182]
**0,476953468**	9,51E-10	7,83E-09	*PLIN2*	perilipin 2 [Source:HGNC Symbol;Acc:HGNC:248]
**0,476748163**	0,001049853	0,003953705	*TCP11L2*	t-complex 11 like 2 [Source:HGNC Symbol;Acc:HGNC:28627]
**0,476191835**	0,011787191	0,034304473	*TBX2*	T-box 2 [Source:HGNC Symbol;Acc:HGNC:11597]
**0,476050931**	1,11E-06	6,72E-06	*RTKN*	rhotekin [Source:HGNC Symbol;Acc:HGNC:10466]
**0,475999988**	1,92E-17	2,74E-16	*DCTN4*	dynactin subunit 4 [Source:HGNC Symbol;Acc:HGNC:15518]
**0,475799134**	9,21E-09	6,96E-08	*CUL9*	cullin 9 [Source:HGNC Symbol;Acc:HGNC:15982]
**0,475746496**	7,33E-08	5,07E-07	*PLA2R1*	phospholipase A2 receptor 1 [Source:HGNC Symbol;Acc:HGNC:9042]
**0,474521152**	7,75E-10	6,44E-09	*TPBG*	trophoblast glycoprotein [Source:HGNC Symbol;Acc:HGNC:12004]
**0,474454191**	0,009109241	0,027398317	*VILL*	villin like [Source:HGNC Symbol;Acc:HGNC:30906]
**0,474044461**	0,00085208	0,003278244	*GLB1L*	galactosidase beta 1 like [Source:HGNC Symbol;Acc:HGNC:28129]
**0,473803155**	1,34E-06	8,06E-06	*NECAB3*	N-terminal EF-hand calcium binding protein 3 [Source:HGNC Symbol;Acc:HGNC:15851]
**0,473102337**	0,000876994	0,003364092	*ABRAXAS1*	abraxas 1, BRCA1 A complex subunit [Source:HGNC Symbol;Acc:HGNC:25829]
**0,47304257**	1,11E-27	2,47E-26	*MVP*	major vault protein [Source:HGNC Symbol;Acc:HGNC:7531]
**0,472490515**	9,99E-10	8,20E-09	*ZNF516*	zinc finger protein 516 [Source:HGNC Symbol;Acc:HGNC:28990]
**0,472254094**	1,23E-05	6,46E-05	*COL14A1*	collagen type XIV alpha 1 chain [Source:HGNC Symbol;Acc:HGNC:2191]
**0,472121951**	2,41E-07	1,57E-06	*ENO2*	enolase 2 [Source:HGNC Symbol;Acc:HGNC:3353]
**0,471881013**	2,65E-05	0,000132888	*TNRC6C*	trinucleotide repeat containing 6C [Source:HGNC Symbol;Acc:HGNC:29318]
**0,471870308**	4,25E-11	3,95E-10	*SRGAP2*	SLIT-ROBO Rho GTPase activating protein 2 [Source:HGNC Symbol;Acc:HGNC:19751]
**0,471121585**	1,65E-20	2,72E-19	*LIMK1*	LIM domain kinase 1 [Source:HGNC Symbol;Acc:HGNC:6613]
**0,47065352**	0,00114749	0,004287409	*FAM69C*	family with sequence similarity 69 member C [Source:HGNC Symbol;Acc:HGNC:31729]
**0,470280476**	4,71E-09	3,66E-08	*MRPL45*	mitochondrial ribosomal protein L45 [Source:HGNC Symbol;Acc:HGNC:16651]
**0,470161471**	0,000592188	0,002345784	*N4BP2*	NEDD4 binding protein 2 [Source:HGNC Symbol;Acc:HGNC:29851]

**0,469039775**	0,002282259	0,007991612	*GABRB3*	gamma-aminobutyric acid type A receptor beta3 subunit [Source:HGNC Symbol;Acc:HGNC:4083]
**0,468993622**	0,000274787	0,001154976	*JAK2*	Janus kinase 2 [Source:HGNC Symbol;Acc:HGNC:6192]
**0,468992723**	9,89E-07	6,03E-06	*SUOX*	sulfite oxidase [Source:HGNC Symbol;Acc:HGNC:11460]
**0,468778671**	1,10E-22	1,98E-21	*S100A16*	S100 calcium binding protein A16 [Source:HGNC Symbol;Acc:HGNC:20441]
**0,468657702**	2,55E-12	2,63E-11	*FDPS*	farnesyl diphosphate synthase [Source:HGNC Symbol;Acc:HGNC:3631]
**0,468534247**	5,89E-23	1,07E-21	*SOX9*	SRY-box 9 [Source:HGNC Symbol;Acc:HGNC:11204]
**0,468341116**	2,85E-11	2,68E-10	*ZKSCAN8*	zinc finger with KRAB and SCAN domains 8 [Source:HGNC Symbol;Acc:HGNC:12983]
**0,468026816**	4,73E-07	2,98E-06	*ANK3*	ankyrin 3 [Source:HGNC Symbol;Acc:HGNC:494]
**0,467905906**	9,55E-31	2,42E-29	*PHLDA1*	pleckstrin homology like domain family A member 1 [Source:HGNC Symbol;Acc:HGNC:8933]
**0,467711155**	0,003507208	0,011752873	*RNASEL*	ribonuclease L [Source:HGNC Symbol;Acc:HGNC:10050]
**0,467554075**	0,012820021	0,036868009	*ZFP14*	ZFP14 zinc finger protein [Source:HGNC Symbol;Acc:HGNC:29312]
**0,467047594**	0,000160014	0,000704295	*CLCN4*	chloride voltage-gated channel 4 [Source:HGNC Symbol;Acc:HGNC:2022]
**0,46702282**	7,99E-24	1,52E-22	*GPRC5B*	G protein-coupled receptor class C group 5 member B [Source:HGNC Symbol;Acc:HGNC:13308]
**0,466921704**	2,70E-11	2,54E-10	*SLC25A36*	solute carrier family 25 member 36 [Source:HGNC Symbol;Acc:HGNC:25554]
**0,46673195**	4,84E-06	2,69E-05	*TRIM16L*	tripartite motif containing 16 like [Source:HGNC Symbol;Acc:HGNC:32670]
**0,466441159**	5,38E-09	4,15E-08	*ABCD1*	ATP binding cassette subfamily D member 1 [Source:HGNC Symbol;Acc:HGNC:61]
**0,465694189**	2,56E-10	2,22E-09	*RUNX1*	runt related transcription factor 1 [Source:HGNC Symbol;Acc:HGNC:10471]
**0,46503869**	0,000327624	0,001358231	*GSTM2*	glutathione S-transferase mu 2 [Source:HGNC Symbol;Acc:HGNC:4634]
**0,465003998**	3,07E-08	2,20E-07	*EPC2*	enhancer of polycomb homolog 2 [Source:HGNC Symbol;Acc:HGNC:24543]
**0,464875689**	2,73E-24	5,34E-23	*GPC1*	glypican 1 [Source:HGNC Symbol;Acc:HGNC:4449]
**0,46438043**	0,005423912	0,01736332	*ZKSCAN3*	zinc finger with KRAB and SCAN domains 3 [Source:HGNC Symbol;Acc:HGNC:13853]
**0,464313111**	1,07E-08	8,02E-08	*RPRD2*	regulation of nuclear pre-mRNA domain containing 2 [Source:HGNC Symbol;Acc:HGNC:29039]
**0,463839019**	3,51E-08	2,51E-07	*KLC4*	kinesin light chain 4 [Source:HGNC Symbol;Acc:HGNC:21624]
**0,463772873**	4,26E-05	0,000207657	*MYL5*	myosin light chain 5 [Source:HGNC Symbol;Acc:HGNC:7586]
**0,463754981**	0,007131413	0,022099428	*SH2B2*	SH2B adaptor protein 2 [Source:HGNC Symbol;Acc:HGNC:17381]
**0,463553368**	0,000475367	0,001917875	*SPON2*	spondin 2 [Source:HGNC Symbol;Acc:HGNC:11253]
**0,463030399**	2,60E-06	1,50E-05	*APH1B*	aph-1 homolog B, gamma-secretase subunit [Source:HGNC Symbol;Acc:HGNC:24080]
**0,463008575**	2,99E-25	6,07E-24	*GALNT10*	polypeptide N-acetylgalactosaminyltransferase 10 [Source:HGNC Symbol;Acc:HGNC:19873]
**0,462601825**	1,22E-07	8,23E-07	*ING4*	inhibitor of growth family member 4 [Source:HGNC Symbol;Acc:HGNC:19423]
**0,462547561**	4,13E-07	2,62E-06	*STIM2*	stromal interaction molecule 2 [Source:HGNC Symbol;Acc:HGNC:19205]
**0,462354047**	3,64E-10	3,11E-09	*TBC1D17*	TBC1 domain family member 17 [Source:HGNC Symbol;Acc:HGNC:25699]
**0,462298418**	4,50E-15	5,55E-14	*ARX*	aristaless related homeobox [Source:HGNC Symbol;Acc:HGNC:18060]
**0,462257754**	8,77E-16	1,12E-14	*GNPDA1*	glucosamine-6-phosphate deaminase 1 [Source:HGNC Symbol;Acc:HGNC:4417]
**0,46225104**	0,004863343	0,015750674	*CCDC125*	coiled-coil domain containing 125 [Source:HGNC Symbol;Acc:HGNC:28924]

**0,461774662**	9,22E-07	5,64E-06	*ZBTB44*	zinc finger and BTB domain containing 44 [Source:HGNC Symbol;Acc:HGNC:25001]
**0,461720978**	0,000755638	0,002938071	*TRAPPC6A*	trafficking protein particle complex 6A [Source:HGNC Symbol;Acc:HGNC:23069]
**0,461151314**	0,00457056	0,014909445	*ZNF446*	zinc finger protein 446 [Source:HGNC Symbol;Acc:HGNC:21036]
**0,460638388**	2,83E-09	2,24E-08	*MGAT5B*	alpha-1,6-mannosylglycoprotein 6-beta-N-acetylglucosaminyltransferase B [Source:HGNC Symbol;Acc:HGNC:24140]
**0,460277626**	0,011398843	0,033324186	*CDKL5*	cyclin dependent kinase like 5 [Source:HGNC Symbol;Acc:HGNC:11411]
**0,459728324**	1,11E-08	8,34E-08	*NEPRO*	nucleolus and neural progenitor protein [Source:HGNC Symbol;Acc:HGNC:24496]
**0,45935638**	3,73E-08	2,66E-07	*ALDH6A1*	aldehyde dehydrogenase 6 family member A1 [Source:HGNC Symbol;Acc:HGNC:7179]
**0,459336419**	0,000225228	0,000961242	*ZNF277*	zinc finger protein 277 [Source:HGNC Symbol;Acc:HGNC:13070]
**0,458882372**	0,00024535	0,001040484	*GTF2E1*	general transcription factor IIE subunit 1 [Source:HGNC Symbol;Acc:HGNC:4650]
**0,458804607**	3,14E-10	2,70E-09	*BLOC1S2*	biogenesis of lysosomal organelles complex 1 subunit 2 [Source:HGNC Symbol;Acc:HGNC:20984]
**0,458485761**	5,21E-13	5,70E-12	*PGM2L1*	phosphoglucomutase 2 like 1 [Source:HGNC Symbol;Acc:HGNC:20898]
**0,4582502**	9,21E-17	1,25E-15	*PBX1*	PBX homeobox 1 [Source:HGNC Symbol;Acc:HGNC:8632]
**0,457997126**	9,26E-06	4,95E-05	*GPANK1*	G-patch domain and ankyrin repeats 1 [Source:HGNC Symbol;Acc:HGNC:13920]
**0,457943872**	7,18E-12	7,04E-11	*SYNRG*	synergin gamma [Source:HGNC Symbol;Acc:HGNC:557]
**0,457892673**	3,63E-06	2,05E-05	*FBXL17*	F-box and leucine rich repeat protein 17 [Source:HGNC Symbol;Acc:HGNC:13615]
**0,456802116**	0,000385016	0,001579956	*PHKA1*	phosphorylase kinase regulatory subunit alpha 1 [Source:HGNC Symbol;Acc:HGNC:8925]
**0,456503946**	1,25E-05	6,56E-05	*SEPT4*	septin 4 [Source:HGNC Symbol;Acc:HGNC:9165]
**0,456301432**	0,009066758	0,027291686	*LACC1*	laccase domain containing 1 [Source:HGNC Symbol;Acc:HGNC:26789]
**0,455921208**	6,14E-24	1,18E-22	*CCT7*	chaperonin containing TCP1 subunit 7 [Source:HGNC Symbol;Acc:HGNC:1622]
**0,455673735**	1,53E-05	7,90E-05	*GALNT16*	polypeptide N-acetylgalactosaminyltransferase 16 [Source:HGNC Symbol;Acc:HGNC:23233]
**0,455539882**	5,12E-06	2,83E-05	*ZC3H12C*	zinc finger CCCH-type containing 12C [Source:HGNC Symbol;Acc:HGNC:29362]
**0,455388114**	0,002247732	0,007889218	*PRPF40B*	pre-mRNA processing factor 40 homolog B [Source:HGNC Symbol;Acc:HGNC:25031]
**0,454905683**	6,98E-19	1,07E-17	*TNFRSF10D*	TNF receptor superfamily member 10d [Source:HGNC Symbol;Acc:HGNC:11907]
**0,454734878**	4,79E-05	0,000231273	*PHYKPL*	5-phosphohydroxy-L-lysine phospho-lyase [Source:HGNC Symbol;Acc:HGNC:28249]
**0,454574228**	2,09E-09	1,67E-08	*PLEKHM1*	pleckstrin homology and RUN domain containing M1 [Source:HGNC Symbol;Acc:HGNC:29017]
**0,454430213**	1,28E-07	8,60E-07	*MADD*	MAP kinase activating death domain [Source:HGNC Symbol;Acc:HGNC:6766]
**0,454097984**	2,31E-07	1,51E-06	*CNNM3*	cyclin and CBS domain divalent metal cation transport mediator 3 [Source:HGNC Symbol;Acc:HGNC:104]
**0,454068118**	6,38E-15	7,75E-14	*LAMA5*	laminin subunit alpha 5 [Source:HGNC Symbol;Acc:HGNC:6485]
**0,453898205**	0,00869622	0,026311015	*KCNJ6*	potassium voltage-gated channel subfamily J member 6 [Source:HGNC Symbol;Acc:HGNC:6267]
**0,453329204**	0,002589835	0,008945564	*KANSL1L*	KAT8 regulatory NSL complex subunit 1 like [Source:HGNC Symbol;Acc:HGNC:26310]
**0,451663127**	0,002166152	0,007629102	*SOX1*	SRY-box 1 [Source:HGNC Symbol;Acc:HGNC:11189]
**0,451136243**	2,23E-11	2,12E-10	*GSE1*	Gse1 coiled-coil protein [Source:HGNC Symbol;Acc:HGNC:28979]

**0,450991629**	1,30E-21	2,24E-20	*LAMP2*	lysosomal associated membrane protein 2 [Source:HGNC Symbol;Acc:HGNC:6501]
**0,450948712**	3,74E-05	0,000183962	*THYN1*	thymocyte nuclear protein 1 [Source:HGNC Symbol;Acc:HGNC:29560]
**0,450905442**	1,76E-06	1,04E-05	*SNX21*	sorting nexin family member 21 [Source:HGNC Symbol;Acc:HGNC:16154]
**0,450846783**	8,67E-06	4,66E-05	*PPM1M*	protein phosphatase, Mg2+/Mn2+ dependent 1M [Source:HGNC Symbol;Acc:HGNC:26506]
**0,450814483**	4,32E-17	6,00E-16	*TMEM131*	transmembrane protein 131 [Source:HGNC Symbol;Acc:HGNC:30366]
**0,450547691**	1,44E-22	2,58E-21	*NOP53*	NOP53 ribosome biogenesis factor [Source:HGNC Symbol;Acc:HGNC:4333]
**0,450461252**	1,42E-05	7,36E-05	*MBD5*	methyl-CpG binding domain protein 5 [Source:HGNC Symbol;Acc:HGNC:20444]
**0,449719101**	2,06E-09	1,64E-08	*SH3PXD2A*	SH3 and PX domains 2A [Source:HGNC Symbol;Acc:HGNC:23664]
**0,449594637**	0,012299362	0,035597527	*CRADD*	CASP2 and RIPK1 domain containing adaptor with death domain [Source:HGNC Symbol;Acc:HGNC:2340]
**0,448783839**	0,008216875	0,025020661	*IPMK*	inositol polyphosphate multikinase [Source:HGNC Symbol;Acc:HGNC:20739]
**0,448544732**	0,007618557	0,023405883	*DNAH1*	dynein axonemal heavy chain 1 [Source:HGNC Symbol;Acc:HGNC:2940]
**0,448538996**	0,000425011	0,001731632	*BCAS3*	BCAS3, microtubule associated cell migration factor [Source:HGNC Symbol;Acc:HGNC:14347]
**0,44835951**	0,000276757	0,001162249	*PHLDB3*	pleckstrin homology like domain family B member 3 [Source:HGNC Symbol;Acc:HGNC:30499]
**0,448241319**	4,34E-06	2,42E-05	*NOA1*	nitric oxide associated 1 [Source:HGNC Symbol;Acc:HGNC:28473]
**0,448032441**	6,43E-21	1,08E-19	*SOX4*	SRY-box 4 [Source:HGNC Symbol;Acc:HGNC:11200]
**0,448025497**	5,34E-16	6,91E-15	*VGLL4*	vestigial like family member 4 [Source:HGNC Symbol;Acc:HGNC:28966]
**0,447985025**	2,71E-07	1,76E-06	*ZNF358*	zinc finger protein 358 [Source:HGNC Symbol;Acc:HGNC:16838]
**0,447663009**	6,96E-07	4,31E-06	*NNAT*	neuronatin [Source:HGNC Symbol;Acc:HGNC:7860]
**0,447632355**	5,01E-09	3,88E-08	*AKAP9*	A-kinase anchoring protein 9 [Source:HGNC Symbol;Acc:HGNC:379]
**0,447259451**	0,005644795	0,017982964	*ST8SIA1*	ST8 alpha-N-acetyl-neuraminide alpha-2,8-sialyltransferase 1 [Source:HGNC Symbol;Acc:HGNC:10869]
**0,446714383**	1,14E-20	1,89E-19	*CTDSP2*	CTD small phosphatase 2 [Source:HGNC Symbol;Acc:HGNC:17077]
**0,446601182**	8,98E-11	8,04E-10	*CPT1C*	carnitine palmitoyltransferase 1C [Source:HGNC Symbol;Acc:HGNC:18540]
**0,446570362**	1,73E-11	1,65E-10	*ZNF217*	zinc finger protein 217 [Source:HGNC Symbol;Acc:HGNC:13009]
**0,446383094**	1,27E-14	1,53E-13	*B4GALT5*	beta-1,4-galactosyltransferase 5 [Source:HGNC Symbol;Acc:HGNC:928]
**0,44582099**	3,55E-07	2,27E-06	*OSBPL6*	oxysterol binding protein like 6 [Source:HGNC Symbol;Acc:HGNC:16388]
**0,445403189**	6,01E-07	3,75E-06	*INSYN1*	inhibitory synaptic factor 1 [Source:HGNC Symbol;Acc:HGNC:33753]
**0,445273013**	0,00267078	0,009202619	*IKZF4*	IKAROS family zinc finger 4 [Source:HGNC Symbol;Acc:HGNC:13179]
**0,444729704**	0,000656057	0,002584541	*UVRAG*	UV radiation resistance associated [Source:HGNC Symbol;Acc:HGNC:12640]
**0,444523648**	1,29E-12	1,36E-11	*FCGRT*	Fc fragment of IgG receptor and transporter [Source:HGNC Symbol;Acc:HGNC:3621]
**0,444176043**	1,17E-09	9,53E-09	*ZMYND8*	zinc finger MYND-type containing 8 [Source:HGNC Symbol;Acc:HGNC:9397]
**0,443704362**	2,61E-06	1,50E-05	*GABPA*	GA binding protein transcription factor subunit alpha [Source:HGNC Symbol;Acc:HGNC:4071]

**0,443575535**	8,73E-08	5,99E-07	*SHC3*	SHC adaptor protein 3 [Source:HGNC Symbol;Acc:HGNC:18181]
**0,443158266**	0,002106703	0,007438624	*KCNJ10*	potassium voltage-gated channel subfamily J member 10 [Source:HGNC Symbol;Acc:HGNC:6256]
**0,442910912**	0,008292196	0,025226266	*TMEM255A*	transmembrane protein 255A [Source:HGNC Symbol;Acc:HGNC:26086]
**0,442223153**	0,00048857	0,001965422	*SPATA6*	spermatogenesis associated 6 [Source:HGNC Symbol;Acc:HGNC:18309]
**0,442207191**	1,45E-05	7,54E-05	*CHD7*	chromodomain helicase DNA binding protein 7 [Source:HGNC Symbol;Acc:HGNC:20626]
**0,441878807**	0,003198175	0,010820045	*ACYP2*	acylphosphatase 2 [Source:HGNC Symbol;Acc:HGNC:180]
**0,441865672**	2,33E-08	1,70E-07	*KHDRBS3*	KH RNA binding domain containing, signal transduction associated 3 [Source:HGNC Symbol;Acc:HGNC:18117]
**0,441827412**	2,43E-05	0,000122717	*TIAM2*	T cell lymphoma invasion and metastasis 2 [Source:HGNC Symbol;Acc:HGNC:11806]
**0,440906604**	2,12E-05	0,000108025	*ZNF528*	zinc finger protein 528 [Source:HGNC Symbol;Acc:HGNC:29384]
**0,440159732**	1,98E-14	2,35E-13	*HEXIM1*	hexamethylene bisacetamide inducible 1 [Source:HGNC Symbol;Acc:HGNC:24953]
**0,439815817**	0,000287338	0,001202	*LMTK3*	lemur tyrosine kinase 3 [Source:HGNC Symbol;Acc:HGNC:19295]
**0,439714686**	6,91E-11	6,25E-10	*RNF130*	ring finger protein 130 [Source:HGNC Symbol;Acc:HGNC:18280]
**0,438518631**	0,006363408	0,020040293	*TTC33*	tetratricopeptide repeat domain 33 [Source:HGNC Symbol;Acc:HGNC:29959]
**0,438480734**	1,16E-19	1,84E-18	*ZMAT3*	zinc finger matrin-type 3 [Source:HGNC Symbol;Acc:HGNC:29983]
**0,437602095**	4,86E-26	1,02E-24	*IGFBP2*	insulin like growth factor binding protein 2 [Source:HGNC Symbol;Acc:HGNC:5471]
**0,436745176**	0,013991386	0,039794348	*KIF16B*	kinesin family member 16B [Source:HGNC Symbol;Acc:HGNC:15869]
**0,436618398**	4,06E-06	2,27E-05	*TBKBP1*	TBK1 binding protein 1 [Source:HGNC Symbol;Acc:HGNC:30140]
**0,436588416**	7,54E-16	9,68E-15	*AEBP1*	AE binding protein 1 [Source:HGNC Symbol;Acc:HGNC:303]
**0,436439909**	4,25E-09	3,31E-08	*ALDH2*	aldehyde dehydrogenase 2 family member [Source:HGNC Symbol;Acc:HGNC:404]
**0,435914794**	2,17E-10	1,89E-09	*SGSM2*	small G protein signaling modulator 2 [Source:HGNC Symbol;Acc:HGNC:29026]
**0,435709131**	0,000256417	0,001083158	*CHST7*	carbohydrate sulfotransferase 7 [Source:HGNC Symbol;Acc:HGNC:13817]
**0,435622536**	6,81E-06	3,71E-05	*ZNF397*	zinc finger protein 397 [Source:HGNC Symbol;Acc:HGNC:18818]
**0,435263769**	5,60E-08	3,93E-07	*NME3*	NME/NM23 nucleoside diphosphate kinase 3 [Source:HGNC Symbol;Acc:HGNC:7851]
**0,434741436**	0,000802664	0,00310598	*DMXL2*	Dmx like 2 [Source:HGNC Symbol;Acc:HGNC:2938]
**0,434565685**	1,50E-09	1,22E-08	*KLF3*	Kruppel like factor 3 [Source:HGNC Symbol;Acc:HGNC:16516]
**0,434452069**	4,05E-10	3,45E-09	*NADSYN1*	NAD synthetase 1 [Source:HGNC Symbol;Acc:HGNC:29832]
**0,434037859**	3,63E-08	2,59E-07	*ATF7IP*	activating transcription factor 7 interacting protein [Source:HGNC Symbol;Acc:HGNC:20092]
**0,433143405**	0,000511054	0,002046115	*WDR19*	WD repeat domain 19 [Source:HGNC Symbol;Acc:HGNC:18340]
**0,432825432**	1,14E-05	6,02E-05	*AFTPH*	aftiphilin [Source:HGNC Symbol;Acc:HGNC:25951]
**0,432413582**	0,001296701	0,004786718	*CSPG5*	chondroitin sulfate proteoglycan 5 [Source:HGNC Symbol;Acc:HGNC:2467]
**0,431989445**	6,68E-05	0,000315573	*COA5*	cytochrome c oxidase assembly factor 5 [Source:HGNC Symbol;Acc:HGNC:33848]
**0,431358175**	0,009612043	0,028679474	*BTN3A2*	butyrophilin subfamily 3 member A2 [Source:HGNC Symbol;Acc:HGNC:1139]

**0,431309448**	3,16E-07	2,03E-06	*CRTC1*	CREB regulated transcription coactivator 1 [Source:HGNC Symbol;Acc:HGNC:16062]
**0,431150712**	0,000101352	0,000463876	*GPRIN1*	G protein regulated inducer of neurite outgrowth 1 [Source:HGNC Symbol;Acc:HGNC:24835]
**0,43102555**	5,33E-15	6,53E-14	*USP11*	ubiquitin specific peptidase 11 [Source:HGNC Symbol;Acc:HGNC:12609]
**0,431023148**	4,33E-06	2,42E-05	*PIM3*	Pim-3 proto-oncogene, serine/threonine kinase [Source:HGNC Symbol;Acc:HGNC:19310]
**0,430835475**	2,99E-10	2,58E-09	*NDUFB4*	NADH:ubiquinone oxidoreductase subunit B4 [Source:HGNC Symbol;Acc:HGNC:7699]
**0,43059956**	0,012727459	0,036645263	*CNTFR*	ciliary neurotrophic factor receptor [Source:HGNC Symbol;Acc:HGNC:2170]
**0,430447844**	8,82E-07	5,41E-06	*EIF1B*	eukaryotic translation initiation factor 1B [Source:HGNC Symbol;Acc:HGNC:30792]
**0,430443682**	1,24E-12	1,31E-11	*EHMT2*	euchromatic histone lysine methyltransferase 2 [Source:HGNC Symbol;Acc:HGNC:14129]
**0,430367479**	4,79E-06	2,66E-05	*DMAP1*	DNA methyltransferase 1 associated protein 1 [Source:HGNC Symbol;Acc:HGNC:18291]
**0,430336009**	0,003502529	0,01173922	*TRIM45*	tripartite motif containing 45 [Source:HGNC Symbol;Acc:HGNC:19018]
**0,430223996**	0,000960318	0,003644099	*MEIS1*	Meis homeobox 1 [Source:HGNC Symbol;Acc:HGNC:7000]
**0,429996308**	0,013689016	0,039054548	*ZNF610*	zinc finger protein 610 [Source:HGNC Symbol;Acc:HGNC:26687]
**0,429828352**	0,00023906	0,001016255	*CDH3*	cadherin 3 [Source:HGNC Symbol;Acc:HGNC:1762]
**0,4296072**	1,26E-13	1,43E-12	*ISCU*	iron-sulfur cluster assembly enzyme [Source:HGNC Symbol;Acc:HGNC:29882]
**0,429407096**	0,000494775	0,001987086	*TAF6L*	TATA-box binding protein associated factor 6 like [Source:HGNC Symbol;Acc:HGNC:17305]
**0,428841456**	0,001726815	0,006196482	*IFT74*	intraflagellar transport 74 [Source:HGNC Symbol;Acc:HGNC:21424]
**0,428645019**	0,000282575	0,001184632	*CAB39L*	calcium binding protein 39 like [Source:HGNC Symbol;Acc:HGNC:20290]
**0,428592208**	0,000261481	0,001102868	*FNIP2*	folliculin interacting protein 2 [Source:HGNC Symbol;Acc:HGNC:29280]
**0,428211323**	0,001053292	0,00396358	*PELI1*	pellino E3 ubiquitin protein ligase 1 [Source:HGNC Symbol;Acc:HGNC:8827]
**0,428108451**	0,009984843	0,02967318	*SRGAP2B*	SLIT-ROBO Rho GTPase activating protein 2B [Source:HGNC Symbol;Acc:HGNC:35237]
**0,428083061**	4,71E-10	3,99E-09	*THRA*	thyroid hormone receptor alpha [Source:HGNC Symbol;Acc:HGNC:11796]
**0,428004392**	1,07E-06	6,47E-06	*AGBL5*	ATP/GTP binding protein like 5 [Source:HGNC Symbol;Acc:HGNC:26147]
**0,427959899**	2,68E-06	1,54E-05	*TAF1*	TATA-box binding protein associated factor 1 [Source:HGNC Symbol;Acc:HGNC:11535]
**0,427939433**	5,26E-10	4,43E-09	*KMT2E*	lysine methyltransferase 2E [Source:HGNC Symbol;Acc:HGNC:18541]
**0,427547839**	1,27E-07	8,56E-07	*ABCD4*	ATP binding cassette subfamily D member 4 [Source:HGNC Symbol;Acc:HGNC:68]
**0,426775422**	4,21E-12	4,24E-11	*MYO10*	myosin X [Source:HGNC Symbol;Acc:HGNC:7593]
**0,426414367**	2,55E-05	0,000128535	*CYSTM1*	cysteine rich transmembrane module containing 1 [Source:HGNC Symbol;Acc:HGNC:30239]
**0,4262621**	1,06E-06	6,46E-06	*ZSCAN18*	zinc finger and SCAN domain containing 18 [Source:HGNC Symbol;Acc:HGNC:21037]
**0,425375815**	0,006623406	0,020741297	*MOB3C*	MOB kinase activator 3C [Source:HGNC Symbol;Acc:HGNC:29800]
**0,425013932**	5,82E-08	4,07E-07	*FOXJ3*	forkhead box J3 [Source:HGNC Symbol;Acc:HGNC:29178]
**0,424922419**	0,000473985	0,001913287	*BDH2*	3-hydroxybutyrate dehydrogenase 2 [Source:HGNC Symbol;Acc:HGNC:32389]
**0,424914884**	0,000589952	0,002337881	*GLI4*	GLI family zinc finger 4 [Source:HGNC Symbol;Acc:HGNC:4320]

**0,42485447**	3,02E-06	1,72E-05	*HEXA*	hexosaminidase subunit alpha [Source:HGNC Symbol;Acc:HGNC:4878]
**0,424727714**	3,18E-07	2,04E-06	*FAM131B*	family with sequence similarity 131 member B [Source:HGNC Symbol;Acc:HGNC:22202]
**0,424632564**	5,15E-06	2,85E-05	*PPP1R21*	protein phosphatase 1 regulatory subunit 21 [Source:HGNC Symbol;Acc:HGNC:30595]
**0,424499929**	1,13E-08	8,44E-08	*SEMA4B*	semaphorin 4B [Source:HGNC Symbol;Acc:HGNC:10730]
**0,423550764**	0,000786616	0,003050575	*PLPP4*	phospholipid phosphatase 4 [Source:HGNC Symbol;Acc:HGNC:23531]
**0,423454874**	0,004137953	0,013612667	*B9D1*	B9 domain containing 1 [Source:HGNC Symbol;Acc:HGNC:24123]
**0,422996524**	4,55E-18	6,71E-17	*SREBF1*	sterol regulatory element binding transcription factor 1 [Source:HGNC Symbol;Acc:HGNC:11289]
**0,422814075**	1,90E-21	3,26E-20	*AES*	amino-terminal enhancer of split [Source:HGNC Symbol;Acc:HGNC:307]
**0,422255836**	1,61E-08	1,19E-07	*CHMP1B*	charged multivesicular body protein 1B [Source:HGNC Symbol;Acc:HGNC:24287]
**0,422121643**	0,000383687	0,001574836	*TEF*	TEF, PAR bZIP transcription factor [Source:HGNC Symbol;Acc:HGNC:11722]
**0,422035343**	5,35E-09	4,13E-08	*USP6NL*	USP6 N-terminal like [Source:HGNC Symbol;Acc:HGNC:16858]
**0,421373999**	0,007435028	0,022905883	*MPPED2*	metallophosphoesterase domain containing 2 [Source:HGNC Symbol;Acc:HGNC:1180]
**0,421305428**	5,60E-11	5,10E-10	*PIK3R3*	phosphoinositide-3-kinase regulatory subunit 3 [Source:HGNC Symbol;Acc:HGNC:8981]
**0,421247117**	2,91E-05	0,00014514	*SRGAP2C*	SLIT-ROBO Rho GTPase activating protein 2C [Source:HGNC Symbol;Acc:HGNC:30584]
**0,421201843**	2,33E-11	2,21E-10	*CLSTN3*	calsyntenin 3 [Source:HGNC Symbol;Acc:HGNC:18371]
**0,42076853**	0,001330846	0,004900646	*ZNF425*	zinc finger protein 425 [Source:HGNC Symbol;Acc:HGNC:20690]
**0,420721215**	0,006948125	0,021610458	*ZNF211*	zinc finger protein 211 [Source:HGNC Symbol;Acc:HGNC:13003]
**0,420390024**	0,000513853	0,002056473	*AMMECR1*	Alport syndrome, mental retardation, midface hypoplasia and elliptocytosis chromosomal region gene 1 [Source:HGNC Symbol;Acc:HGNC:467]
**0,420098537**	1,15E-16	1,56E-15	*FYN*	FYN proto-oncogene, Src family tyrosine kinase [Source:HGNC Symbol;Acc:HGNC:4037]
**0,419983658**	4,41E-21	7,48E-20	*ELOVL5*	ELOVL fatty acid elongase 5 [Source:HGNC Symbol;Acc:HGNC:21308]
**0,419891459**	1,08E-07	7,30E-07	*TOM1*	target of myb1 membrane trafficking protein [Source:HGNC Symbol;Acc:HGNC:11982]
**0,419497295**	5,24E-11	4,80E-10	*CPNE3*	copine 3 [Source:HGNC Symbol;Acc:HGNC:2316]
**0,419436248**	7,26E-05	0,000340491	*SMIM20*	small integral membrane protein 20 [Source:HGNC Symbol;Acc:HGNC:37260]
**0,419418403**	1,93E-05	9,87E-05	*TRPT1*	tRNA phosphotransferase 1 [Source:HGNC Symbol;Acc:HGNC:20316]
**0,419111278**	6,16E-21	1,04E-19	*SDCBP*	syndecan binding protein [Source:HGNC Symbol;Acc:HGNC:10662]
**0,419058472**	1,01E-18	1,53E-17	*ATP1A1*	ATPase Na+/K+ transporting subunit alpha 1 [Source:HGNC Symbol;Acc:HGNC:799]
**0,418897175**	0,014183696	0,040258709	*DYRK3*	dual specificity tyrosine phosphorylation regulated kinase 3 [Source:HGNC Symbol;Acc:HGNC:3094]
**0,418330135**	3,95E-08	2,81E-07	*CCNDBP1*	cyclin D1 binding protein 1 [Source:HGNC Symbol;Acc:HGNC:1587]
**0,417988971**	5,58E-07	3,49E-06	*EDA2R*	ectodysplasin A2 receptor [Source:HGNC Symbol;Acc:HGNC:17756]
**0,417988237**	1,05E-07	7,12E-07	*GSTO1*	glutathione S-transferase omega 1 [Source:HGNC Symbol;Acc:HGNC:13312]
**0,417854271**	0,00038666	0,001586367	*ZMYM5*	zinc finger MYM-type containing 5 [Source:HGNC Symbol;Acc:HGNC:13029]
**0,417035469**	0,000137089	0,000611574	*CNNM2*	cyclin and CBS domain divalent metal cation transport mediator 2 [Source:HGNC Symbol;Acc:HGNC:103]

**0,416571087**	0,010135183	0,03006012	*MRM3*	mitochondrial rRNA methyltransferase 3 [Source:HGNC Symbol;Acc:HGNC:18485]
**0,416549844**	1,05E-05	5,56E-05	*DCP2*	decapping mRNA 2 [Source:HGNC Symbol;Acc:HGNC:24452]
**0,416199385**	0,001480075	0,005399949	*MSRA*	methionine sulfoxide reductase A [Source:HGNC Symbol;Acc:HGNC:7377]
**0,416137239**	0,00066521	0,00261582	*LRP1B*	LDL receptor related protein 1B [Source:HGNC Symbol;Acc:HGNC:6693]
**0,416050422**	5,11E-07	3,21E-06	*HMGCL*	3-hydroxy-3-methylglutaryl-CoA lyase [Source:HGNC Symbol;Acc:HGNC:5005]
**0,415666383**	0,006510474	0,020453799	*AUH*	AU RNA binding methylglutaconyl-CoA hydratase [Source:HGNC Symbol;Acc:HGNC:890]
**0,415592064**	7,11E-08	4,94E-07	*WASHC2C*	WASH complex subunit 2C [Source:HGNC Symbol;Acc:HGNC:23414]
**0,415286922**	9,67E-06	5,15E-05	*MAPRE3*	microtubule associated protein RP/EB family member 3 [Source:HGNC Symbol;Acc:HGNC:6892]
**0,415060502**	1,54E-15	1,94E-14	*SERINC3*	serine incorporator 3 [Source:HGNC Symbol;Acc:HGNC:11699]
**0,414942136**	9,83E-15	1,19E-13	*BAG3*	BCL2 associated athanogene 3 [Source:HGNC Symbol;Acc:HGNC:939]
**0,414343728**	3,73E-13	4,11E-12	*PARP4*	poly(ADP-ribose) polymerase family member 4 [Source:HGNC Symbol;Acc:HGNC:271]
**0,414049667**	6,32E-12	6,26E-11	*HEXB*	hexosaminidase subunit beta [Source:HGNC Symbol;Acc:HGNC:4879]
**0,413820584**	3,91E-06	2,20E-05	*ATG7*	autophagy related 7 [Source:HGNC Symbol;Acc:HGNC:16935]
**0,413544701**	1,58E-10	1,39E-09	*MCCC2*	methylcrotonoyl-CoA carboxylase 2 [Source:HGNC Symbol;Acc:HGNC:6937]
**0,413339877**	6,23E-05	0,000295829	*NR1D2*	nuclear receptor subfamily 1 group D member 2 [Source:HGNC Symbol;Acc:HGNC:7963]
**0,412896595**	3,05E-14	3,59E-13	*WEE1*	WEE1 G2 checkpoint kinase [Source:HGNC Symbol;Acc:HGNC:12761]
**0,412604025**	4,43E-10	3,76E-09	*ATP9A*	ATPase phospholipid transporting 9A (putative) [Source:HGNC Symbol;Acc:HGNC:13540]
**0,412553662**	1,74E-06	1,03E-05	*RUFY3*	RUN and FYVE domain containing 3 [Source:HGNC Symbol;Acc:HGNC:30285]
**0,412170302**	6,95E-05	0,000327215	*ZFHX3*	zinc finger homeobox 3 [Source:HGNC Symbol;Acc:HGNC:777]
**0,41190205**	9,30E-12	9,07E-11	*SLC6A8*	solute carrier family 6 member 8 [Source:HGNC Symbol;Acc:HGNC:11055]
**0,411681867**	2,59E-07	1,69E-06	*TSPAN6*	tetraspanin 6 [Source:HGNC Symbol;Acc:HGNC:11858]
**0,411535493**	0,001932991	0,006870262	*SLC25A42*	solute carrier family 25 member 42 [Source:HGNC Symbol;Acc:HGNC:28380]
**0,411320846**	0,01225056	0,035493335	*ENO3*	enolase 3 [Source:HGNC Symbol;Acc:HGNC:3354]
**0,411289017**	0,000683007	0,002681454	*FUZ*	fuzzy planar cell polarity protein [Source:HGNC Symbol;Acc:HGNC:26219]
**0,410742804**	7,99E-14	9,17E-13	*GALNT7*	polypeptide N-acetylgalactosaminyltransferase 7 [Source:HGNC Symbol;Acc:HGNC:4129]
**0,410699354**	7,57E-08	5,23E-07	*NRIP1*	nuclear receptor interacting protein 1 [Source:HGNC Symbol;Acc:HGNC:8001]
**0,41059805**	0,002524438	0,008748079	*TWIST2*	twist family bHLH transcription factor 2 [Source:HGNC Symbol;Acc:HGNC:20670]
**0,410375974**	3,10E-11	2,90E-10	*BCL9*	B cell CLL/lymphoma 9 [Source:HGNC Symbol;Acc:HGNC:1008]
**0,409864373**	6,67E-08	4,64E-07	*RFX7*	regulatory factor X7 [Source:HGNC Symbol;Acc:HGNC:25777]
**0,408798367**	0,009243021	0,027739829	*SDK2*	sidekick cell adhesion molecule 2 [Source:HGNC Symbol;Acc:HGNC:19308]
**0,408617765**	1,43E-13	1,61E-12	*UBE3A*	ubiquitin protein ligase E3A [Source:HGNC Symbol;Acc:HGNC:12496]
**0,408518294**	0,005512147	0,017610918	*PER3*	period circadian regulator 3 [Source:HGNC Symbol;Acc:HGNC:8847]

**0,408149934**	0,000969685	0,003675332	*KCNN3*	potassium calcium-activated channel subfamily N member 3 [Source:HGNC Symbol;Acc:HGNC:6292]
**0,407982918**	7,75E-11	6,97E-10	*ACAT2*	acetyl-CoA acetyltransferase 2 [Source:HGNC Symbol;Acc:HGNC:94]
**0,406174075**	7,68E-09	5,85E-08	*EPN2*	epsin 2 [Source:HGNC Symbol;Acc:HGNC:18639]
**0,406037003**	7,47E-08	5,16E-07	*ERG28*	ergosterol biosynthesis 28 homolog [Source:HGNC Symbol;Acc:HGNC:1187]
**0,405444072**	2,21E-13	2,46E-12	*SLC22A23*	solute carrier family 22 member 23 [Source:HGNC Symbol;Acc:HGNC:21106]
**0,405313193**	1,23E-08	9,20E-08	*VANGL2*	VANGL planar cell polarity protein 2 [Source:HGNC Symbol;Acc:HGNC:15511]
**0,404930212**	4,87E-23	8,93E-22	*LRP10*	LDL receptor related protein 10 [Source:HGNC Symbol;Acc:HGNC:14553]
**0,404526075**	1,72E-07	1,14E-06	*SLK*	STE20 like kinase [Source:HGNC Symbol;Acc:HGNC:11088]
**0,404396617**	3,82E-06	2,15E-05	*DLG4*	discs large MAGUK scaffold protein 4 [Source:HGNC Symbol;Acc:HGNC:2903]
**0,404102503**	4,47E-11	4,12E-10	*DVL2*	dishevelled segment polarity protein 2 [Source:HGNC Symbol;Acc:HGNC:3086]
**0,40315641**	4,40E-11	4,07E-10	*CYFIP2*	cytoplasmic FMR1 interacting protein 2 [Source:HGNC Symbol;Acc:HGNC:13760]
**0,402942854**	0,007838703	0,024016082	*PHYHD1*	phytanoyl-CoA dioxygenase domain containing 1 [Source:HGNC Symbol;Acc:HGNC:23396]
**0,402288611**	0,001308286	0,004825934	*DPM3*	dolichyl-phosphate mannosyltransferase subunit 3 [Source:HGNC Symbol;Acc:HGNC:3007]
**0,402275062**	2,40E-11	2,27E-10	*SMARCA2*	SWI/SNF related, matrix associated, actin dependent regulator of chromatin, subfamily a, member 2 [Source:HGNC Symbol;Acc:HGNC:11098]
**0,402143938**	0,002044465	0,0072373	*MCCC1*	methylcrotonoyl-CoA carboxylase 1 [Source:HGNC Symbol;Acc:HGNC:6936]
**0,402079481**	1,28E-12	1,35E-11	*DDIT4*	DNA damage inducible transcript 4 [Source:HGNC Symbol;Acc:HGNC:24944]
**0,401999745**	1,37E-09	1,11E-08	*PAIP2*	poly(A) binding protein interacting protein 2 [Source:HGNC Symbol;Acc:HGNC:17970]
**0,401967127**	0,000684817	0,002685845	*MIA2*	MIA SH3 domain ER export factor 2 [Source:HGNC Symbol;Acc:HGNC:18432]
**0,401892768**	6,37E-08	4,44E-07	*SLC35C2*	solute carrier family 35 member C2 [Source:HGNC Symbol;Acc:HGNC:17117]
**0,401841388**	1,53E-07	1,02E-06	*ADA2*	adenosine deaminase 2 [Source:HGNC Symbol;Acc:HGNC:1839]
**0,401822664**	4,55E-10	3,85E-09	*PHF23*	PHD finger protein 23 [Source:HGNC Symbol;Acc:HGNC:28428]
**0,401396742**	1,37E-11	1,32E-10	*ARAP1*	ArfGAP with RhoGAP domain, ankyrin repeat and PH domain 1 [Source:HGNC Symbol;Acc:HGNC:16925]
**0,401355476**	0,00864572	0,026190891	*PCSK4*	proprotein convertase subtilisin/kexin type 4 [Source:HGNC Symbol;Acc:HGNC:8746]
**0,400824035**	0,016978099	0,047007702	*ST6GAL1*	ST6 beta-galactoside alpha-2,6-sialyltransferase 1 [Source:HGNC Symbol;Acc:HGNC:10860]
**0,400738693**	6,57E-05	0,00031082	*NAPRT*	nicotinate phosphoribosyltransferase [Source:HGNC Symbol;Acc:HGNC:30450]
**0,400171072**	0,001281541	0,00473616	*LGALS8*	galectin 8 [Source:HGNC Symbol;Acc:HGNC:6569]
**0,400149774**	5,69E-08	3,99E-07	*ZNF687*	zinc finger protein 687 [Source:HGNC Symbol;Acc:HGNC:29277]
**0,400132713**	4,38E-09	3,41E-08	*WASHC2A*	WASH complex subunit 2A [Source:HGNC Symbol;Acc:HGNC:23416]
**-6,426481538**	3,83E-17	5,34E-16	*KRT17*	keratin 17 [Source:HGNC Symbol;Acc:HGNC:6427]
**-6,167605712**	2,06E-06	1,20E-05	*CCDC190*	coiled-coil domain containing 190 [Source:HGNC Symbol;Acc:HGNC:28736]
**-6,080014365**	3,02E-06	1,72E-05	*HLA-DQA2*	major histocompatibility complex, class II, DQ alpha 2 [Source:HGNC Symbol;Acc:HGNC:4943]
**-6,045241702**	4,62E-08	3,27E-07	*KCNJ13*	potassium voltage-gated channel subfamily J member 13 [Source:HGNC Symbol;Acc:HGNC:6259]

**-5,90396903**	4,33E-05	0,000210588	*TRPC6*	transient receptor potential cation channel subfamily C member 6 [Source:HGNC Symbol;Acc:HGNC:12338]
**-5,811821332**	5,89E-05	0,000280792	*NPPB*	natriuretic peptide B [Source:HGNC Symbol;Acc:HGNC:7940]
**-5,412154295**	0,000387943	0,001591295	*CHRM2*	cholinergic receptor muscarinic 2 [Source:HGNC Symbol;Acc:HGNC:1951]
**-5,382535102**	7,82E-05	0,000364677	*SOST*	sclerostin [Source:HGNC Symbol;Acc:HGNC:13771]
**-5,377963503**	9,73E-05	0,000447026	*KCNMB1*	potassium calcium-activated channel subfamily M regulatory beta subunit 1 [Source:HGNC Symbol;Acc:HGNC:6285]
**-5,155104609**	9,46E-06	5,05E-05	*C5orf46*	chromosome 5 open reading frame 46 [Source:HGNC Symbol;Acc:HGNC:33768]
**-4,643477889**	0,001756494	0,006292509	*C11orf88*	chromosome 11 open reading frame 88 [Source:HGNC Symbol;Acc:HGNC:25061]
**-4,63920801**	0,001578489	0,00571357	*SSTR1*	somatostatin receptor 1 [Source:HGNC Symbol;Acc:HGNC:11330]
**-4,588642185**	0,002555299	0,008837665	*RAET1L*	retinoic acid early transcript 1L [Source:HGNC Symbol;Acc:HGNC:16798]
**-4,575251396**	0,001977015	0,007015167	*PRND*	prion like protein doppel [Source:HGNC Symbol;Acc:HGNC:15748]
**-4,54538278**	0	0	*CNN1*	calponin 1 [Source:HGNC Symbol;Acc:HGNC:2155]
**-4,372468224**	2,05E-05	0,000104443	*NPAS4*	neuronal PAS domain protein 4 [Source:HGNC Symbol;Acc:HGNC:18983]
**-4,371845368**	1,11E-77	8,80E-76	*ACTA2*	actin, alpha 2, smooth muscle, aorta [Source:HGNC Symbol;Acc:HGNC:130]
**-4,246282114**	0,000857266	0,003294931	*MYOG*	myogenin [Source:HGNC Symbol;Acc:HGNC:7612]
**-4,242306054**	0,005976161	0,018949898	*ACAN*	aggrecan [Source:HGNC Symbol;Acc:HGNC:319]
**-4,063078198**	1,44E-06	8,58E-06	*GPR17*	G protein-coupled receptor 17 [Source:HGNC Symbol;Acc:HGNC:4471]
**-4,003257884**	3,45E-40	1,17E-38	*LMOD1*	leiomodin 1 [Source:HGNC Symbol;Acc:HGNC:6647]
**-3,911610326**	2,99E-292	3,42E-289	*ANKRD1*	ankyrin repeat domain 1 [Source:HGNC Symbol;Acc:HGNC:15819]
**-3,812571183**	1,37E-70	9,56E-69	*CEMIP*	cell migration inducing hyaluronidase 1 [Source:HGNC Symbol;Acc:HGNC:29213]
**-3,688334932**	0,001162398	0,004332982	*KRTAP2-3*	keratin associated protein 2-3 [Source:HGNC Symbol;Acc:HGNC:18906]
**-3,611393431**	2,28E-08	1,66E-07	*HLA-DQA1*	major histocompatibility complex, class II, DQ alpha 1 [Source:HGNC Symbol;Acc:HGNC:4942]
**-3,581521172**	0,000217044	0,000929791	*MYO7B*	myosin VIIB [Source:HGNC Symbol;Acc:HGNC:7607]
**-3,549093501**	8,45E-30	2,04E-28	*GFAP*	glial fibrillary acidic protein [Source:HGNC Symbol;Acc:HGNC:4235]
**-3,50303486**	1,51E-53	7,24E-52	*TSPAN2*	tetraspanin 2 [Source:HGNC Symbol;Acc:HGNC:20659]
**-3,427273026**	2,09E-05	0,000106536	*ADTRP*	androgen dependent TFPI regulating protein [Source:HGNC Symbol;Acc:HGNC:21214]
**-3,412569638**	0,013719989	0,039125659	*IGF2*	insulin like growth factor 2 [Source:HGNC Symbol;Acc:HGNC:5466]
**-3,386061841**	4,37E-226	2,42E-223	*CPA4*	carboxypeptidase A4 [Source:HGNC Symbol;Acc:HGNC:15740]
**-3,375406179**	2,18E-71	1,57E-69	*ACTC1*	actin, alpha, cardiac muscle 1 [Source:HGNC Symbol;Acc:HGNC:143]
**-3,366012762**	2,97E-06	1,70E-05	*HLA-DRB5*	major histocompatibility complex, class II, DR beta 5 [Source:HGNC Symbol;Acc:HGNC:4953]
**-3,359191539**	0,004626113	0,015067866	*SCUBE1*	signal peptide, CUB domain and EGF like domain containing 1 [Source:HGNC Symbol;Acc:HGNC:13441]
**-3,355380039**	9,12E-37	2,82E-35	*LIX1*	limb and CNS expressed 1 [Source:HGNC Symbol;Acc:HGNC:18581]
**-3,330346775**	2,78E-246	2,00E-243	*ACTG2*	actin, gamma 2, smooth muscle, enteric [Source:HGNC Symbol;Acc:HGNC:145]
**-3,321747963**	0	0	*TAGLN*	transgelin [Source:HGNC Symbol;Acc:HGNC:11553]
**-3,304460344**	3,08E-30	7,59E-29	*RPRM*	reprimo, TP53 dependent G2 arrest mediator homolog [Source:HGNC Symbol;Acc:HGNC:24201]

**-3,302049979**	2,08E-14	2,47E-13	*ACTA1*	actin, alpha 1, skeletal muscle [Source:HGNC Symbol;Acc:HGNC:129]
**-3,287162968**	0,000558778	0,002221605	*LTF*	lactotransferrin [Source:HGNC Symbol;Acc:HGNC:6720]
**-3,268597205**	5,58E-143	1,24E-140	*LIMS2*	LIM zinc finger domain containing 2 [Source:HGNC Symbol;Acc:HGNC:16084]
**-3,267887614**	7,24E-17	9,95E-16	*CYP24A1*	cytochrome P450 family 24 subfamily A member 1 [Source:HGNC Symbol;Acc:HGNC:2602]
**-3,243625923**	0,007596216	0,023343179	*FGF7*	fibroblast growth factor 7 [Source:HGNC Symbol;Acc:HGNC:3685]
**-3,22703896**	0,00737767	0,022750845	*CBLC*	Cbl proto-oncogene C [Source:HGNC Symbol;Acc:HGNC:15961]
**-3,208228978**	2,34E-30	5,85E-29	*SAMD11*	sterile alpha motif domain containing 11 [Source:HGNC Symbol;Acc:HGNC:28706]
**-3,189411872**	7,31E-24	1,39E-22	*ACTBL2*	actin, beta like 2 [Source:HGNC Symbol;Acc:HGNC:17780]
**-3,171260906**	2,32E-09	1,85E-08	*COL6A6*	collagen type VI alpha 6 chain [Source:HGNC Symbol;Acc:HGNC:27023]
**-3,135242164**	1,34E-243	9,32E-241	*C1QL1*	complement C1q like 1 [Source:HGNC Symbol;Acc:HGNC:24182]
**-3,121846691**	4,77E-28	1,08E-26	*PLPPR1*	phospholipid phosphatase related 1 [Source:HGNC Symbol;Acc:HGNC:25993]
**-3,088900322**	0,002637442	0,009097439	*DAW1*	dynein assembly factor with WD repeats 1 [Source:HGNC Symbol;Acc:HGNC:26383]
**-3,00409957**	2,26E-76	1,73E-74	*OXTR*	oxytocin receptor [Source:HGNC Symbol;Acc:HGNC:8529]
**-2,997843263**	6,34E-88	6,22E-86	*IL32*	interleukin 32 [Source:HGNC Symbol;Acc:HGNC:16830]
**-2,994478613**	5,21E-119	8,02E-117	*MYL9*	myosin light chain 9 [Source:HGNC Symbol;Acc:HGNC:15754]
**-2,981199857**	1,18E-06	7,10E-06	*ABCB5*	ATP binding cassette subfamily B member 5 [Source:HGNC Symbol;Acc:HGNC:46]
**-2,901904148**	0,016992882	0,047028503	*ELF3*	E74 like ETS transcription factor 3 [Source:HGNC Symbol;Acc:HGNC:3318]
**-2,879064601**	0,000985646	0,003732912	*ADRB1*	adrenoceptor beta 1 [Source:HGNC Symbol;Acc:HGNC:285]
**-2,867829818**	1,80E-111	2,54E-109	*EDN1*	endothelin 1 [Source:HGNC Symbol;Acc:HGNC:3176]
**-2,840761554**	2,15E-06	1,26E-05	*SCN1A*	sodium voltage-gated channel alpha subunit 1 [Source:HGNC Symbol;Acc:HGNC:10585]
**-2,803643668**	2,73E-06	1,57E-05	*ADCYAP1*	adenylate cyclase activating polypeptide 1 [Source:HGNC Symbol;Acc:HGNC:241]
**-2,781202444**	0	0	*CTGF*	connective tissue growth factor [Source:HGNC Symbol;Acc:HGNC:2500]
**-2,772130549**	8,26E-68	5,49E-66	*CRYAB*	crystallin alpha B [Source:HGNC Symbol;Acc:HGNC:2389]
**-2,770087081**	0	0	*THBS1*	thrombospondin 1 [Source:HGNC Symbol;Acc:HGNC:11785]
**-2,742045041**	0	0	*LBH*	limb bud and heart development [Source:HGNC Symbol;Acc:HGNC:29532]
**-2,741290756**	5,27E-52	2,43E-50	*EGLN3*	egl-9 family hypoxia inducible factor 3 [Source:HGNC Symbol;Acc:HGNC:14661]
**-2,723022637**	0	0	*CYR61*	cysteine rich angiogenic inducer 61 [Source:HGNC Symbol;Acc:HGNC:2654]
**-2,686028977**	3,64E-35	1,07E-33	*PDGFB*	platelet derived growth factor subunit B [Source:HGNC Symbol;Acc:HGNC:8800]
**-2,682957387**	2,48E-41	8,69E-40	*HLA-DPA1*	major histocompatibility complex, class II, DP alpha 1 [Source:HGNC Symbol;Acc:HGNC:4938]
**-2,65103924**	4,93E-11	4,52E-10	*KRT81*	keratin 81 [Source:HGNC Symbol;Acc:HGNC:6458]
**-2,639898466**	0,010439613	0,030821716	*SLC28A3*	solute carrier family 28 member 3 [Source:HGNC Symbol;Acc:HGNC:16484]
**-2,634275327**	1,90E-15	2,39E-14	*TNFRSF9*	TNF receptor superfamily member 9 [Source:HGNC Symbol;Acc:HGNC:11924]
**-2,621318896**	2,75E-12	2,83E-11	*RIMS1*	regulating synaptic membrane exocytosis 1 [Source:HGNC Symbol;Acc:HGNC:17282]

**-2,621307529**	8,86E-49	3,84E-47	*COL8A2*	collagen type VIII alpha 2 chain [Source:HGNC Symbol;Acc:HGNC:2216]
**-2,620265083**	0,006226116	0,019649374	*TRIM67*	tripartite motif containing 67 [Source:HGNC Symbol;Acc:HGNC:31859]
**-2,617852796**	0,001057996	0,003978965	*KLRG2*	killer cell lectin like receptor G2 [Source:HGNC Symbol;Acc:HGNC:24778]
**-2,610733921**	0,000500235	0,00200694	*NXPH2*	neurexophilin 2 [Source:HGNC Symbol;Acc:HGNC:8076]
**-2,589869466**	3,53E-200	1,34E-197	*EFEMP1*	EGF containing fibulin extracellular matrix protein 1 [Source:HGNC Symbol;Acc:HGNC:3218]
**-2,573773028**	3,74E-65	2,43E-63	*COL26A1*	collagen type XXVI alpha 1 chain [Source:HGNC Symbol;Acc:HGNC:18038]
**-2,570799347**	4,08E-74	3,01E-72	*BDNF*	brain derived neurotrophic factor [Source:HGNC Symbol;Acc:HGNC:1033]
**-2,570745111**	9,48E-05	0,00043642	*LCP1*	lymphocyte cytosolic protein 1 [Source:HGNC Symbol;Acc:HGNC:6528]
**-2,569410117**	4,73E-44	1,79E-42	*MYBL1*	MYB proto-oncogene like 1 [Source:HGNC Symbol;Acc:HGNC:7547]
**-2,545218886**	1,79E-25	3,67E-24	*NPR3*	natriuretic peptide receptor 3 [Source:HGNC Symbol;Acc:HGNC:7945]
**-2,545169145**	0,006931661	0,021567391	*OLFML1*	olfactomedin like 1 [Source:HGNC Symbol;Acc:HGNC:24473]
**-2,541447464**	1,33E-17	1,91E-16	*HLA-DMB*	major histocompatibility complex, class II, DM beta [Source:HGNC Symbol;Acc:HGNC:4935]
**-2,536207712**	0,001890758	0,006728782	*GIPC2*	GIPC PDZ domain containing family member 2 [Source:HGNC Symbol;Acc:HGNC:18177]
**-2,53220855**	1,33E-38	4,31E-37	*RPE65*	RPE65, retinoid isomerohydrolase [Source:HGNC Symbol;Acc:HGNC:10294]
**-2,516972754**	2,67E-68	1,78E-66	*PCDH7*	protocadherin 7 [Source:HGNC Symbol;Acc:HGNC:8659]
**-2,511415573**	1,24E-23	2,32E-22	*CLDN6*	claudin 6 [Source:HGNC Symbol;Acc:HGNC:2048]
**-2,495857696**	3,52E-26	7,42E-25	*HLA-DQB1*	major histocompatibility complex, class II, DQ beta 1 [Source:HGNC Symbol;Acc:HGNC:4944]
**-2,49268247**	2,88E-187	9,98E-185	*LMO7*	LIM domain 7 [Source:HGNC Symbol;Acc:HGNC:6646]
**-2,492280681**	1,99E-15	2,49E-14	*NGF*	nerve growth factor [Source:HGNC Symbol;Acc:HGNC:7808]
**-2,488087451**	6,19E-123	9,85E-121	*JPH2*	junctophilin 2 [Source:HGNC Symbol;Acc:HGNC:14202]
**-2,486884112**	3,83E-07	2,44E-06	*CES1*	carboxylesterase 1 [Source:HGNC Symbol;Acc:HGNC:1863]
**-2,486143101**	3,76E-94	4,08E-92	*DIAPH3*	diaphanous related formin 3 [Source:HGNC Symbol;Acc:HGNC:15480]
**-2,484119707**	3,49E-12	3,54E-11	*ABCC9*	ATP binding cassette subfamily C member 9 [Source:HGNC Symbol;Acc:HGNC:60]
**-2,482615487**	0	0	*TPM1*	tropomyosin 1 [Source:HGNC Symbol;Acc:HGNC:12010]
**-2,467072741**	3,10E-90	3,16E-88	*MEGF6*	multiple EGF like domains 6 [Source:HGNC Symbol;Acc:HGNC:3232]
**-2,456881954**	3,65E-82	3,21E-80	*ANXA3*	annexin A3 [Source:HGNC Symbol;Acc:HGNC:541]
**-2,444042278**	0,013990439	0,039794348	*MYBPH*	myosin binding protein H [Source:HGNC Symbol;Acc:HGNC:7552]
**-2,428803588**	5,82E-21	9,83E-20	*FGFR3*	fibroblast growth factor receptor 3 [Source:HGNC Symbol;Acc:HGNC:3690]
**-2,426723857**	2,09E-06	1,22E-05	*PAQR5*	progestin and adipoQ receptor family member 5 [Source:HGNC Symbol;Acc:HGNC:29645]
**-2,423429599**	1,52E-137	3,11E-135	*FZD5*	frizzled class receptor 5 [Source:HGNC Symbol;Acc:HGNC:4043]
**-2,406944584**	2,81E-77	2,22E-75	*SH3RF2*	SH3 domain containing ring finger 2 [Source:HGNC Symbol;Acc:HGNC:26299]
**-2,40422223**	2,41E-28	5,54E-27	*L1CAM*	L1 cell adhesion molecule [Source:HGNC Symbol;Acc:HGNC:6470]
**-2,397495298**	3,78E-88	3,72E-86	*TENT5B*	terminal nucleotidyltransferase 5B [Source:HGNC Symbol;Acc:HGNC:28273]
**-2,38145862**	1,42E-28	3,29E-27	*HLA-DRB1*	major histocompatibility complex, class II, DR beta 1 [Source:HGNC Symbol;Acc:HGNC:4948]

**-2,36967907**	9,17E-49	3,96E-47	*GPRC5A*	G protein-coupled receptor class C group 5 member A [Source:HGNC Symbol;Acc:HGNC:9836]
**-2,361872901**	0,002207631	0,007759696	*C1QTNF2*	C1q and TNF related 2 [Source:HGNC Symbol;Acc:HGNC:14325]
**-2,359791954**	1,62E-247	1,21E-244	*HLA-DRA*	major histocompatibility complex, class II, DR alpha [Source:HGNC Symbol;Acc:HGNC:4947]
**-2,342292664**	4,54E-18	6,69E-17	*TINAGL1*	tubulointerstitial nephritis antigen like 1 [Source:HGNC Symbol;Acc:HGNC:19168]
**-2,338513419**	3,85E-42	1,38E-40	*BRIP1*	BRCA1 interacting protein C-terminal helicase 1 [Source:HGNC Symbol;Acc:HGNC:20473]
**-2,336416041**	6,85E-09	5,24E-08	*ERBB4*	erb-b2 receptor tyrosine kinase 4 [Source:HGNC Symbol;Acc:HGNC:3432]
**-2,333798713**	3,51E-69	2,40E-67	*HSPG2*	heparan sulfate proteoglycan 2 [Source:HGNC Symbol;Acc:HGNC:5273]
**-2,32330981**	0,015506556	0,043454185	*ITIH3*	inter-alpha-trypsin inhibitor heavy chain 3 [Source:HGNC Symbol;Acc:HGNC:6168]
**-2,32135173**	0,00210883	0,00744478	*DCAF12L1*	DDB1 and CUL4 associated factor 12 like 1 [Source:HGNC Symbol;Acc:HGNC:29395]
**-2,304039608**	7,03E-111	9,68E-109	*RFLNA*	refilin A [Source:HGNC Symbol;Acc:HGNC:27051]
**-2,276006124**	1,49E-133	2,91E-131	*CRISPLD2*	cysteine rich secretory protein LCCL domain containing 2 [Source:HGNC Symbol;Acc:HGNC:25248]
**-2,272312728**	0,000572196	0,002271692	*B3GALT2*	beta-1,3-galactosyltransferase 2 [Source:HGNC Symbol;Acc:HGNC:917]
**-2,270200462**	4,26E-38	1,36E-36	*KCNIP1*	potassium voltage-gated channel interacting protein 1 [Source:HGNC Symbol;Acc:HGNC:15521]
**-2,252748838**	0,001743966	0,006252246	*PTPRQ*	protein tyrosine phosphatase, receptor type Q [Source:HGNC Symbol;Acc:HGNC:9679]
**-2,251948023**	4,04E-163	1,14E-160	*PHLDB2*	pleckstrin homology like domain family B member 2 [Source:HGNC Symbol;Acc:HGNC:29573]
**-2,24996232**	3,12E-08	2,24E-07	*EPPK1*	epiplakin 1 [Source:HGNC Symbol;Acc:HGNC:15577]
**-2,235003241**	4,22E-161	1,15E-158	*NEXN*	nexilin F-actin binding protein [Source:HGNC Symbol;Acc:HGNC:29557]
**-2,230986504**	4,30E-05	0,00020937	*TC2N*	tandem C2 domains, nuclear [Source:HGNC Symbol;Acc:HGNC:19859]
**-2,230938821**	1,92E-14	2,28E-13	*RELN*	reelin [Source:HGNC Symbol;Acc:HGNC:9957]
**-2,202691487**	1,45E-167	4,62E-165	*RASSF3*	Ras association domain family member 3 [Source:HGNC Symbol;Acc:HGNC:14271]
**-2,188009071**	0	0	*SLC7A5*	solute carrier family 7 member 5 [Source:HGNC Symbol;Acc:HGNC:11063]
**-2,183334412**	0,001236736	0,004582794	*SLFN13*	schlafen family member 13 [Source:HGNC Symbol;Acc:HGNC:26481]
**-2,182018528**	4,38E-16	5,69E-15	*HLA-DPB1*	major histocompatibility complex, class II, DP beta 1 [Source:HGNC Symbol;Acc:HGNC:4940]
**-2,181754022**	0,004755804	0,015438462	*STON1-GTF2A1L*	STON1-GTF2A1L readthrough [Source:HGNC Symbol;Acc:HGNC:30651]
**-2,180137014**	1,33E-294	1,62E-291	*STC2*	stanniocalcin 2 [Source:HGNC Symbol;Acc:HGNC:11374]
**-2,176302804**	9,89E-50	4,41E-48	*IQCJ-SCHIP1*	IQCJ-SCHIP1 readthrough [Source:HGNC Symbol;Acc:HGNC:38842]
**-2,169246191**	2,21E-70	1,54E-68	*VGLL3*	vestigial like family member 3 [Source:HGNC Symbol;Acc:HGNC:24327]
**-2,163398207**	3,55E-09	2,79E-08	*PLP1*	proteolipid protein 1 [Source:HGNC Symbol;Acc:HGNC:9086]
**-2,157314266**	3,94E-21	6,70E-20	*AKAP5*	A-kinase anchoring protein 5 [Source:HGNC Symbol;Acc:HGNC:375]
**-2,156663124**	5,20E-06	2,88E-05	*IL34*	interleukin 34 [Source:HGNC Symbol;Acc:HGNC:28529]
**-2,147823543**	4,50E-32	1,19E-30	*SULF1*	sulfatase 1 [Source:HGNC Symbol;Acc:HGNC:20391]
**-2,14369795**	4,28E-28	9,69E-27	*CLEC19A*	C-type lectin domain containing 19A [Source:HGNC Symbol;Acc:HGNC:34522]
**-2,142546937**	1,30E-34	3,76E-33	*FAT3*	FAT atypical cadherin 3 [Source:HGNC Symbol;Acc:HGNC:23112]
**-2,129066452**	1,31E-23	2,46E-22	*RGS5*	regulator of G protein signaling 5 [Source:HGNC Symbol;Acc:HGNC:10001]

**-2,126284249**	1,25E-06	7,54E-06	*LRRC2*	leucine rich repeat containing 2 [Source:HGNC Symbol;Acc:HGNC:14676]
**-2,125410795**	0,014023792	0,039870165	*ATAD3C*	ATPase family, AAA domain containing 3C [Source:HGNC Symbol;Acc:HGNC:32151]
**-2,124188605**	7,41E-08	5,12E-07	*DOCK8*	dedicator of cytokinesis 8 [Source:HGNC Symbol;Acc:HGNC:19191]
**-2,123236691**	4,76E-171	1,54E-168	*GALNT17*	polypeptide N-acetylgalactosaminyltransferase 17 [Source:HGNC Symbol;Acc:HGNC:16347]
**-2,117321655**	0,010342411	0,030576605	*ALKAL2*	ALK and LTK ligand 2 [Source:HGNC Symbol;Acc:HGNC:27683]
**-2,116688186**	0,000394549	0,001617027	*KISS1*	KiSS-1 metastasis suppressor [Source:HGNC Symbol;Acc:HGNC:6341]
**-2,092510187**	0,000126039	0,000567104	*STX11*	syntaxin 11 [Source:HGNC Symbol;Acc:HGNC:11429]
**-2,091668237**	0,001564097	0,005670253	*HAND2*	heart and neural crest derivatives expressed 2 [Source:HGNC Symbol;Acc:HGNC:4808]
**-2,07474047**	2,78E-41	9,72E-40	*KIF18B*	kinesin family member 18B [Source:HGNC Symbol;Acc:HGNC:27102]
**-2,071612363**	3,04E-10	2,62E-09	*ADRB2*	adrenoceptor beta 2 [Source:HGNC Symbol;Acc:HGNC:286]
**-2,062896431**	0,01190099	0,034604528	*TMEM139*	transmembrane protein 139 [Source:HGNC Symbol;Acc:HGNC:22058]
**-2,062732796**	7,82E-69	5,27E-67	*FILIP1L*	filamin A interacting protein 1 like [Source:HGNC Symbol;Acc:HGNC:24589]
**-2,060874993**	1,99E-07	1,31E-06	*GSG1L*	GSG1 like [Source:HGNC Symbol;Acc:HGNC:28283]
**-2,060186408**	1,41E-09	1,14E-08	*ALS2CL*	ALS2 C-terminal like [Source:HGNC Symbol;Acc:HGNC:20605]
**-2,05720146**	3,19E-192	1,15E-189	*FSTL3*	follistatin like 3 [Source:HGNC Symbol;Acc:HGNC:3973]
**-2,052605917**	1,10E-111	1,57E-109	*GBP1*	guanylate binding protein 1 [Source:HGNC Symbol;Acc:HGNC:4182]
**-2,051571496**	6,10E-255	5,15E-252	*COL11A1*	collagen type XI alpha 1 chain [Source:HGNC Symbol;Acc:HGNC:2186]
**-2,050743546**	0,000493662	0,00198385	*PTGES3L*	prostaglandin E synthase 3 like [Source:HGNC Symbol;Acc:HGNC:43943]
**-2,048276154**	1,13E-291	1,22E-288	*MEST*	mesoderm specific transcript [Source:HGNC Symbol;Acc:HGNC:7028]
**-2,042059953**	0,010013671	0,029740635	*GPC3*	glypican 3 [Source:HGNC Symbol;Acc:HGNC:4451]
**-2,040608499**	0,017959767	0,049324545	*PROKR1*	prokineticin receptor 1 [Source:HGNC Symbol;Acc:HGNC:4524]
**-2,039786801**	9,54E-64	6,03E-62	*CRB2*	crumbs 2, cell polarity complex component [Source:HGNC Symbol;Acc:HGNC:18688]
**-2,038787303**	8,65E-115	1,28E-112	*P3H2*	prolyl 3-hydroxylase 2 [Source:HGNC Symbol;Acc:HGNC:19317]
**-2,034815235**	4,95E-06	2,74E-05	*KCNK3*	potassium two pore domain channel subfamily K member 3 [Source:HGNC Symbol;Acc:HGNC:6278]
**-2,030647411**	2,92E-55	1,48E-53	*ADAMTSL3*	ADAMTS like 3 [Source:HGNC Symbol;Acc:HGNC:14633]
**-2,027285678**	5,11E-09	3,95E-08	*SLC16A12*	solute carrier family 16 member 12 [Source:HGNC Symbol;Acc:HGNC:23094]
**-2,027098882**	4,61E-22	8,10E-21	*MCM10*	minichromosome maintenance 10 replication initiation factor [Source:HGNC Symbol;Acc:HGNC:18043]
**-2,026315793**	0,010219134	0,030253626	*C1QL4*	complement C1q like 4 [Source:HGNC Symbol;Acc:HGNC:31416]
**-2,024019444**	8,90E-35	2,58E-33	*HLA-DMA*	major histocompatibility complex, class II, DM alpha [Source:HGNC Symbol;Acc:HGNC:4934]
**-2,019212096**	0	0	*GAS6*	growth arrest specific 6 [Source:HGNC Symbol;Acc:HGNC:4168]
**-2,018957792**	5,23E-05	0,000251606	*CCDC81*	coiled-coil domain containing 81 [Source:HGNC Symbol;Acc:HGNC:26281]
**-2,013073156**	5,94E-69	4,03E-67	*PROM1*	prominin 1 [Source:HGNC Symbol;Acc:HGNC:9454]
**-2,011971426**	1,73E-08	1,28E-07	*CA3*	carbonic anhydrase 3 [Source:HGNC Symbol;Acc:HGNC:1374]

**-2,009893348**	1,07E-77	8,58E-76	*KIT*	KIT proto-oncogene receptor tyrosine kinase [Source:HGNC Symbol;Acc:HGNC:6342]
**-1,998587436**	0,014982021	0,042130285	*KCTD8*	potassium channel tetramerization domain containing 8 [Source:HGNC Symbol;Acc:HGNC:22394]
**-1,994359696**	5,94E-54	2,89E-52	*MYBL2*	MYB proto-oncogene like 2 [Source:HGNC Symbol;Acc:HGNC:7548]
**-1,989899321**	1,30E-06	7,79E-06	*HAPLN1*	hyaluronan and proteoglycan link protein 1 [Source:HGNC Symbol;Acc:HGNC:2380]
**-1,978341194**	1,62E-44	6,22E-43	*TSPAN12*	tetraspanin 12 [Source:HGNC Symbol;Acc:HGNC:21641]
**-1,976706625**	3,23E-56	1,68E-54	*KIF23*	kinesin family member 23 [Source:HGNC Symbol;Acc:HGNC:6392]
**-1,968394364**	3,95E-09	3,09E-08	*C6orf132*	chromosome 6 open reading frame 132 [Source:HGNC Symbol;Acc:HGNC:21288]
**-1,967327118**	5,54E-251	4,48E-248	*AMOTL2*	angiomotin like 2 [Source:HGNC Symbol;Acc:HGNC:17812]
**-1,961427712**	2,19E-17	3,11E-16	*FAM111B*	family with sequence similarity 111 member B [Source:HGNC Symbol;Acc:HGNC:24200]
**-1,961023948**	1,02E-08	7,67E-08	*TRIM58*	tripartite motif containing 58 [Source:HGNC Symbol;Acc:HGNC:24150]
**-1,957876745**	1,07E-12	1,14E-11	*ADAMTS5*	ADAM metallopeptidase with thrombospondin type 1 motif 5 [Source:HGNC Symbol;Acc:HGNC:221]
**-1,955299569**	4,37E-30	1,07E-28	*AURKB*	aurora kinase B [Source:HGNC Symbol;Acc:HGNC:11390]
**-1,953967539**	1,13E-123	1,86E-121	*LAYN*	layilin [Source:HGNC Symbol;Acc:HGNC:29471]
**-1,948693469**	5,09E-34	1,44E-32	*PDCD1LG2*	programmed cell death 1 ligand 2 [Source:HGNC Symbol;Acc:HGNC:18731]
**-1,943220954**	0,003923666	0,012986981	*OR2I1P*	olfactory receptor family 2 subfamily I member 1 pseudogene [Source:HGNC Symbol;Acc:HGNC:8258]
**-1,942540706**	6,05E-35	1,76E-33	*BIRC3*	baculoviral IAP repeat containing 3 [Source:HGNC Symbol;Acc:HGNC:591]
**-1,942357675**	9,98E-101	1,22E-98	*SLIT3*	slit guidance ligand 3 [Source:HGNC Symbol;Acc:HGNC:11087]
**-1,936403877**	8,57E-09	6,49E-08	*C3*	complement C3 [Source:HGNC Symbol;Acc:HGNC:1318]
**-1,933031279**	1,63E-08	1,20E-07	*LRRC7*	leucine rich repeat containing 7 [Source:HGNC Symbol;Acc:HGNC:18531]
**-1,927110816**	5,61E-13	6,12E-12	*GALNT3*	polypeptide N-acetylgalactosaminyltransferase 3 [Source:HGNC Symbol;Acc:HGNC:4125]
**-1,926127132**	7,22E-13	7,80E-12	*P2RY6*	pyrimidinergic receptor P2Y6 [Source:HGNC Symbol;Acc:HGNC:8543]
**-1,916281694**	9,74E-06	5,19E-05	*FANCB*	FA complementation group B [Source:HGNC Symbol;Acc:HGNC:3583]
**-1,916154861**	6,76E-21	1,13E-19	*CDH20*	cadherin 20 [Source:HGNC Symbol;Acc:HGNC:1760]
**-1,91481774**	0,016709753	0,046417045	*MYOCD*	myocardin [Source:HGNC Symbol;Acc:HGNC:16067]
**-1,906681874**	6,22E-28	1,39E-26	*MMP24*	matrix metallopeptidase 24 [Source:HGNC Symbol;Acc:HGNC:7172]
**-1,906167513**	0,00121056	0,004496099	*FMO1*	flavin containing monooxygenase 1 [Source:HGNC Symbol;Acc:HGNC:3769]
**-1,903684081**	1,35E-33	3,75E-32	*FGFR2*	fibroblast growth factor receptor 2 [Source:HGNC Symbol;Acc:HGNC:3689]
**-1,902651674**	1,62E-175	5,32E-173	*MSRB3*	methionine sulfoxide reductase B3 [Source:HGNC Symbol;Acc:HGNC:27375]
**-1,900312308**	1,13E-43	4,25E-42	*SMAD6*	SMAD family member 6 [Source:HGNC Symbol;Acc:HGNC:6772]
**-1,894641206**	8,59E-13	9,20E-12	*HAS2*	hyaluronan synthase 2 [Source:HGNC Symbol;Acc:HGNC:4819]
**-1,892186148**	1,34E-166	4,07E-164	*F3*	coagulation factor III, tissue factor [Source:HGNC Symbol;Acc:HGNC:3541]
**-1,88815614**	4,60E-22	8,09E-21	*PLCB4*	phospholipase C beta 4 [Source:HGNC Symbol;Acc:HGNC:9059]
**-1,884988723**	1,90E-24	3,73E-23	*DTL*	denticleless E3 ubiquitin protein ligase homolog [Source:HGNC Symbol;Acc:HGNC:30288]
**-1,884328982**	4,54E-164	1,29E-161	*ALPK2*	alpha kinase 2 [Source:HGNC Symbol;Acc:HGNC:20565]

**-1,880836421**	2,66E-152	6,80E-150	*MICAL2*	microtubule associated monooxygenase, calponin and LIM domain containing 2 [Source:HGNC Symbol;Acc:HGNC:24693]
**-1,877929487**	0,000336267	0,00139109	*LYPD6B*	LY6/PLAUR domain containing 6B [Source:HGNC Symbol;Acc:HGNC:27018]
**-1,877567369**	6,71E-270	5,92E-267	*CD74*	CD74 molecule [Source:HGNC Symbol;Acc:HGNC:1697]
**-1,877481804**	4,74E-08	3,35E-07	*E2F2*	E2F transcription factor 2 [Source:HGNC Symbol;Acc:HGNC:3114]
**-1,875204324**	4,10E-34	1,16E-32	*KRT80*	keratin 80 [Source:HGNC Symbol;Acc:HGNC:27056]
**-1,870226985**	1,71E-103	2,17E-101	*ANLN*	anillin actin binding protein [Source:HGNC Symbol;Acc:HGNC:14082]
**-1,864468313**	2,82E-11	2,65E-10	*CHL1*	cell adhesion molecule L1 like [Source:HGNC Symbol;Acc:HGNC:1939]
**-1,858978609**	0	0	*GPC4*	glypican 4 [Source:HGNC Symbol;Acc:HGNC:4452]
**-1,848896428**	1,70E-08	1,26E-07	*FSTL5*	follistatin like 5 [Source:HGNC Symbol;Acc:HGNC:21386]
**-1,848659161**	1,95E-32	5,25E-31	*VSIR*	V-set immunoregulatory receptor [Source:HGNC Symbol;Acc:HGNC:30085]
**-1,847236543**	2,11E-60	1,23E-58	*GADD45B*	growth arrest and DNA damage inducible beta [Source:HGNC Symbol;Acc:HGNC:4096]
**-1,831608186**	3,12E-36	9,51E-35	*IQGAP3*	IQ motif containing GTPase activating protein 3 [Source:HGNC Symbol;Acc:HGNC:20669]
**-1,830549882**	7,88E-37	2,44E-35	*CDC6*	cell division cycle 6 [Source:HGNC Symbol;Acc:HGNC:1744]
**-1,82694484**	0,003146322	0,010674393	*NOTUM*	notum, palmitoleoyl-protein carboxylesterase [Source:HGNC Symbol;Acc:HGNC:27106]
**-1,823523857**	1,12E-141	2,43E-139	*PDE1C*	phosphodiesterase 1C [Source:HGNC Symbol;Acc:HGNC:8776]
**-1,823293478**	2,15E-32	5,76E-31	*RRM2*	ribonucleotide reductase regulatory subunit M2 [Source:HGNC Symbol;Acc:HGNC:10452]
**-1,820873674**	2,68E-179	8,96E-177	*DDAH1*	dimethylarginine dimethylaminohydrolase 1 [Source:HGNC Symbol;Acc:HGNC:2715]
**-1,811154247**	1,27E-06	7,66E-06	*LPL*	lipoprotein lipase [Source:HGNC Symbol;Acc:HGNC:6677]
**-1,808126702**	5,59E-12	5,56E-11	*SEZ6*	seizure related 6 homolog [Source:HGNC Symbol;Acc:HGNC:15955]
**-1,803180894**	0,000184531	0,000802917	*MTFR2*	mitochondrial fission regulator 2 [Source:HGNC Symbol;Acc:HGNC:21115]
**-1,801178086**	0,00149418	0,005447313	*ARHGEF10*	Rho guanine nucleotide exchange factor 10 [Source:HGNC Symbol;Acc:HGNC:14103]
**-1,791896053**	5,45E-15	6,66E-14	*CD200*	CD200 molecule [Source:HGNC Symbol;Acc:HGNC:7203]
**-1,790118471**	1,28E-40	4,38E-39	*DAAM2*	dishevelled associated activator of morphogenesis 2 [Source:HGNC Symbol;Acc:HGNC:18143]
**-1,783990703**	6,94E-159	1,85E-156	*PADI2*	peptidyl arginine deiminase 2 [Source:HGNC Symbol;Acc:HGNC:18341]
**-1,781763611**	1,24E-197	4,54E-195	*GLIPR1*	GLI pathogenesis related 1 [Source:HGNC Symbol;Acc:HGNC:17001]
**-1,78157244**	1,60E-124	2,67E-122	*GREM1*	gremlin 1, DAN family BMP antagonist [Source:HGNC Symbol;Acc:HGNC:2001]
**-1,772337834**	7,99E-13	8,60E-12	*CENPI*	centromere protein I [Source:HGNC Symbol;Acc:HGNC:3968]
**-1,770224261**	0,002713648	0,009338723	*SCNN1B*	sodium channel epithelial 1 beta subunit [Source:HGNC Symbol;Acc:HGNC:10600]
**-1,762408447**	1,50E-86	1,44E-84	*TGFB3*	transforming growth factor beta 3 [Source:HGNC Symbol;Acc:HGNC:11769]
**-1,761970729**	7,28E-24	1,39E-22	*CDCA8*	cell division cycle associated 8 [Source:HGNC Symbol;Acc:HGNC:14629]
**-1,760626086**	5,50E-24	1,06E-22	*SFRP4*	secreted frizzled related protein 4 [Source:HGNC Symbol;Acc:HGNC:10778]
**-1,760485883**	3,46E-08	2,48E-07	*ZNF488*	zinc finger protein 488 [Source:HGNC Symbol;Acc:HGNC:23535]
**-1,760277945**	1,56E-22	2,79E-21	*GINS2*	GINS complex subunit 2 [Source:HGNC Symbol;Acc:HGNC:24575]

**-1,752359904**	3,09E-05	0,000153615	*POLE2*	DNA polymerase epsilon 2, accessory subunit [Source:HGNC Symbol;Acc:HGNC:9178]
**-1,750255867**	0,017206321	0,04752432	*KCNQ4*	potassium voltage-gated channel subfamily Q member 4 [Source:HGNC Symbol;Acc:HGNC:6298]
**-1,749132021**	8,49E-13	9,10E-12	*CENPK*	centromere protein K [Source:HGNC Symbol;Acc:HGNC:29479]
**-1,744797081**	0,006337593	0,019965475	*FLT1*	fms related tyrosine kinase 1 [Source:HGNC Symbol;Acc:HGNC:3763]
**-1,736190368**	1,61E-06	9,55E-06	*KCTD4*	potassium channel tetramerization domain containing 4 [Source:HGNC Symbol;Acc:HGNC:23227]
**-1,731771207**	1,99E-135	3,95E-133	*IGFBP4*	insulin like growth factor binding protein 4 [Source:HGNC Symbol;Acc:HGNC:5473]
**-1,729673716**	4,88E-29	1,16E-27	*ASF1B*	anti-silencing function 1B histone chaperone [Source:HGNC Symbol;Acc:HGNC:20996]
**-1,724056219**	4,57E-10	3,88E-09	*CHD5*	chromodomain helicase DNA binding protein 5 [Source:HGNC Symbol;Acc:HGNC:16816]
**-1,718938642**	1,04E-15	1,33E-14	*NTF3*	neurotrophin 3 [Source:HGNC Symbol;Acc:HGNC:8023]
**-1,708343865**	2,66E-139	5,55E-137	*UGCG*	UDP-glucose ceramide glucosyltransferase [Source:HGNC Symbol;Acc:HGNC:12524]
**-1,703359319**	3,66E-25	7,38E-24	*SYNPO2*	synaptopodin 2 [Source:HGNC Symbol;Acc:HGNC:17732]
**-1,701196469**	7,58E-46	3,03E-44	*PTX3*	pentraxin 3 [Source:HGNC Symbol;Acc:HGNC:9692]
**-1,699235931**	6,69E-06	3,65E-05	*EDN2*	endothelin 2 [Source:HGNC Symbol;Acc:HGNC:3177]
**-1,698121832**	3,83E-38	1,23E-36	*CCNA2*	cyclin A2 [Source:HGNC Symbol;Acc:HGNC:1578]
**-1,693348988**	1,77E-06	1,04E-05	*IL7R*	interleukin 7 receptor [Source:HGNC Symbol;Acc:HGNC:6024]
**-1,688699657**	8,74E-10	7,22E-09	*PLCL1*	phospholipase C like 1 (inactive) [Source:HGNC Symbol;Acc:HGNC:9063]
**-1,686273834**	0,001683647	0,006053892	*HLA-F*	major histocompatibility complex, class I, F [Source:HGNC Symbol;Acc:HGNC:4963]
**-1,680745826**	1,05E-24	2,07E-23	*MATN2*	matrilin 2 [Source:HGNC Symbol;Acc:HGNC:6908]
**-1,680017606**	3,89E-78	3,15E-76	*WWC1*	WW and C2 domain containing 1 [Source:HGNC Symbol;Acc:HGNC:29435]
**-1,679520267**	1,92E-23	3,56E-22	*ADM2*	adrenomedullin 2 [Source:HGNC Symbol;Acc:HGNC:28898]
**-1,677976894**	5,53E-133	1,06E-130	*KIFC3*	kinesin family member C3 [Source:HGNC Symbol;Acc:HGNC:6326]
**-1,677881728**	1,79E-29	4,29E-28	*LRRCC1*	leucine rich repeat and coiled-coil centrosomal protein 1 [Source:HGNC Symbol;Acc:HGNC:29373]
**-1,677547277**	5,61E-06	3,09E-05	*E2F8*	E2F transcription factor 8 [Source:HGNC Symbol;Acc:HGNC:24727]
**-1,673513051**	6,53E-34	1,83E-32	*CEP55*	centrosomal protein 55 [Source:HGNC Symbol;Acc:HGNC:1161]
**-1,67323216**	3,30E-21	5,64E-20	*NCAPH*	non-SMC condensin I complex subunit H [Source:HGNC Symbol;Acc:HGNC:1112]
**-1,672120712**	5,95E-46	2,41E-44	*CLDN1*	claudin 1 [Source:HGNC Symbol;Acc:HGNC:2032]
**-1,671754315**	9,82E-08	6,71E-07	*KBTBD8*	kelch repeat and BTB domain containing 8 [Source:HGNC Symbol;Acc:HGNC:30691]
**-1,664582782**	3,69E-73	2,71E-71	*TNFAIP3*	TNF alpha induced protein 3 [Source:HGNC Symbol;Acc:HGNC:11896]
**-1,663183039**	7,95E-08	5,48E-07	*KRT75*	keratin 75 [Source:HGNC Symbol;Acc:HGNC:24431]
**-1,660242638**	6,76E-35	1,97E-33	*GTSE1*	G2 and S-phase expressed 1 [Source:HGNC Symbol;Acc:HGNC:13698]
**-1,660099676**	1,43E-190	5,04E-188	*SDC4*	syndecan 4 [Source:HGNC Symbol;Acc:HGNC:10661]
**-1,659741302**	8,90E-63	5,45E-61	*TUFT1*	tuftelin 1 [Source:HGNC Symbol;Acc:HGNC:12422]
**-1,65884461**	3,76E-33	1,04E-31	*SHCBP1*	SHC binding and spindle associated 1 [Source:HGNC Symbol;Acc:HGNC:29547]
**-1,658105744**	0,009776908	0,029108725	*HCN4*	hyperpolarization activated cyclic nucleotide gated potassium channel 4 [Source:HGNC Symbol;Acc:HGNC:16882]
**-1,65609667**	8,70E-10	7,19E-09	*ITGBL1*	integrin subunit beta like 1 [Source:HGNC Symbol;Acc:HGNC:6164]

**-1,654008646**	0,001409381	0,005167286	*GCNT4*	glucosaminyl (N-acetyl) transferase 4, core 2 [Source:HGNC Symbol;Acc:HGNC:17973]
**-1,651982404**	1,27E-44	4,90E-43	*KIFC1*	kinesin family member C1 [Source:HGNC Symbol;Acc:HGNC:6389]
**-1,651396347**	1,68E-05	8,65E-05	*PAPPA2*	pappalysin 2 [Source:HGNC Symbol;Acc:HGNC:14615]
**-1,650802015**	2,99E-31	7,69E-30	*HLA-DOA*	major histocompatibility complex, class II, DO alpha [Source:HGNC Symbol;Acc:HGNC:4936]
**-1,650212889**	2,42E-231	1,43E-228	*CALD1*	caldesmon 1 [Source:HGNC Symbol;Acc:HGNC:1441]
**-1,647388645**	7,60E-26	1,59E-24	*TCF19*	transcription factor 19 [Source:HGNC Symbol;Acc:HGNC:11629]
**-1,645377215**	7,54E-23	1,37E-21	*PKMYT1*	protein kinase, membrane associated tyrosine/threonine 1 [Source:HGNC Symbol;Acc:HGNC:29650]
**-1,63807686**	1,14E-149	2,70E-147	*IFFO2*	intermediate filament family orphan 2 [Source:HGNC Symbol;Acc:HGNC:27006]
**-1,634864559**	1,37E-07	9,21E-07	*GPR1*	G protein-coupled receptor 1 [Source:HGNC Symbol;Acc:HGNC:4463]
**-1,633810317**	5,21E-152	1,30E-149	*FERMT2*	fermitin family member 2 [Source:HGNC Symbol;Acc:HGNC:15767]
**-1,633254752**	9,71E-35	2,81E-33	*MFSD2A*	major facilitator superfamily domain containing 2A [Source:HGNC Symbol;Acc:HGNC:25897]
**-1,630070359**	1,73E-10	1,52E-09	*XRCC2*	X-ray repair cross complementing 2 [Source:HGNC Symbol;Acc:HGNC:12829]
**-1,627350033**	1,25E-14	1,50E-13	*SCRG1*	stimulator of chondrogenesis 1 [Source:HGNC Symbol;Acc:HGNC:17036]
**-1,626410608**	3,98E-34	1,13E-32	*TRIP13*	thyroid hormone receptor interactor 13 [Source:HGNC Symbol;Acc:HGNC:12307]
**-1,62088973**	1,25E-23	2,35E-22	*ESPL1*	extra spindle pole bodies like 1, separase [Source:HGNC Symbol;Acc:HGNC:16856]
**-1,616733997**	0,009419614	0,028205066	*AJAP1*	adherens junctions associated protein 1 [Source:HGNC Symbol;Acc:HGNC:30801]
**-1,616582437**	0	0	*CCL2*	C-C motif chemokine ligand 2 [Source:HGNC Symbol;Acc:HGNC:10618]
**-1,610412017**	1,12E-06	6,78E-06	*ERCC6L*	ERCC excision repair 6 like, spindle assembly checkpoint helicase [Source:HGNC Symbol;Acc:HGNC:20794]
**-1,60994375**	2,51E-66	1,65E-64	*MKI67*	marker of proliferation Ki-67 [Source:HGNC Symbol;Acc:HGNC:7107]
**-1,609566919**	0,002205533	0,007755132	*MSTN*	myostatin [Source:HGNC Symbol;Acc:HGNC:4223]
**-1,598513752**	0,012999341	0,037328383	*TTPA*	alpha tocopherol transfer protein [Source:HGNC Symbol;Acc:HGNC:12404]
**-1,595327741**	2,36E-16	3,13E-15	*CDC45*	cell division cycle 45 [Source:HGNC Symbol;Acc:HGNC:1739]
**-1,594693848**	0,011483992	0,033552897	*MKX*	mohawk homeobox [Source:HGNC Symbol;Acc:HGNC:23729]
**-1,594259091**	1,05E-61	6,33E-60	*SEMA3F*	semaphorin 3F [Source:HGNC Symbol;Acc:HGNC:10728]
**-1,584772503**	7,55E-44	2,85E-42	*TSPAN18*	tetraspanin 18 [Source:HGNC Symbol;Acc:HGNC:20660]
**-1,582914779**	1,21E-06	7,32E-06	*FILIP1*	filamin A interacting protein 1 [Source:HGNC Symbol;Acc:HGNC:21015]
**-1,575220676**	3,00E-07	1,94E-06	*STOX1*	storkhead box 1 [Source:HGNC Symbol;Acc:HGNC:23508]
**-1,574165814**	1,19E-220	5,94E-218	*SERPINE1*	serpin family E member 1 [Source:HGNC Symbol;Acc:HGNC:8583]
**-1,57313203**	6,37E-19	9,79E-18	*PKP2*	plakophilin 2 [Source:HGNC Symbol;Acc:HGNC:9024]
**-1,570672199**	0,00237285	0,00828044	*TNNC1*	troponin C1, slow skeletal and cardiac type [Source:HGNC Symbol;Acc:HGNC:11943]
**-1,570139027**	1,06E-14	1,28E-13	*CDC25A*	cell division cycle 25A [Source:HGNC Symbol;Acc:HGNC:1725]
**-1,563260448**	3,50E-05	0,000172564	*MRPS24*	mitochondrial ribosomal protein S24 [Source:HGNC Symbol;Acc:HGNC:14510]
**-1,561996596**	6,00E-09	4,61E-08	*PRSS35*	serine protease 35 [Source:HGNC Symbol;Acc:HGNC:21387]
**-1,561741606**	7,20E-50	3,22E-48	*DMD*	dystrophin [Source:HGNC Symbol;Acc:HGNC:2928]

**-1,560278004**	1,44E-15	1,82E-14	*MAOB*	monoamine oxidase B [Source:HGNC Symbol;Acc:HGNC:6834]
**-1,559852721**	0,002694895	0,009277477	*C15orf48*	chromosome 15 open reading frame 48 [Source:HGNC Symbol;Acc:HGNC:29898]
**-1,554518394**	2,09E-31	5,41E-30	*ICAM1*	intercellular adhesion molecule 1 [Source:HGNC Symbol;Acc:HGNC:5344]
**-1,552201696**	1,32E-08	9,86E-08	*C21orf62*	chromosome 21 open reading frame 62 [Source:HGNC Symbol;Acc:HGNC:1305]
**-1,551245037**	7,65E-40	2,56E-38	*CIITA*	class II major histocompatibility complex transactivator [Source:HGNC Symbol;Acc:HGNC:7067]
**-1,551093118**	4,25E-39	1,40E-37	*ENTPD7*	ectonucleoside triphosphate diphosphohydrolase 7 [Source:HGNC Symbol;Acc:HGNC:19745]
**-1,550913411**	3,92E-184	1,34E-181	*FLNA*	filamin A [Source:HGNC Symbol;Acc:HGNC:3754]
**-1,55034752**	1,87E-130	3,50E-128	*NXN*	nucleoredoxin [Source:HGNC Symbol;Acc:HGNC:18008]
**-1,548284844**	5,80E-05	0,000277127	*RARRES2*	retinoic acid receptor responder 2 [Source:HGNC Symbol;Acc:HGNC:9868]
**-1,547201872**	5,64E-123	9,05E-121	*IER3*	immediate early response 3 [Source:HGNC Symbol;Acc:HGNC:5392]
**-1,546227628**	7,97E-86	7,51E-84	*TNFAIP2*	TNF alpha induced protein 2 [Source:HGNC Symbol;Acc:HGNC:11895]
**-1,546063564**	4,52E-13	4,96E-12	*SKA3*	spindle and kinetochore associated complex subunit 3 [Source:HGNC Symbol;Acc:HGNC:20262]
**-1,543143701**	3,23E-90	3,27E-88	*BOK*	BOK, BCL2 family apoptosis regulator [Source:HGNC Symbol;Acc:HGNC:1087]
**-1,540753089**	9,04E-07	5,54E-06	*SIAH3*	siah E3 ubiquitin protein ligase family member 3 [Source:HGNC Symbol;Acc:HGNC:30553]
**-1,540617219**	8,24E-47	3,42E-45	*ID1*	inhibitor of DNA binding 1, HLH protein [Source:HGNC Symbol;Acc:HGNC:5360]
**-1,539461844**	4,69E-96	5,29E-94	*SAMD4A*	sterile alpha motif domain containing 4A [Source:HGNC Symbol;Acc:HGNC:23023]
**-1,536468854**	0,000789047	0,003058777	*AQP4*	aquaporin 4 [Source:HGNC Symbol;Acc:HGNC:637]
**-1,536038526**	6,16E-52	2,84E-50	*LRRN3*	leucine rich repeat neuronal 3 [Source:HGNC Symbol;Acc:HGNC:17200]
**-1,535254801**	1,84E-30	4,60E-29	*RAPH1*	Ras association (RalGDS/AF-6) and pleckstrin homology domains 1 [Source:HGNC Symbol;Acc:HGNC:14436]
**-1,533608432**	0,000309671	0,001289587	*ELOVL7*	ELOVL fatty acid elongase 7 [Source:HGNC Symbol;Acc:HGNC:26292]
**-1,532429693**	7,62E-62	4,59E-60	*DYSF*	dysferlin [Source:HGNC Symbol;Acc:HGNC:3097]
**-1,532340836**	6,47E-10	5,42E-09	*MAP3K7CL*	MAP3K7 C-terminal like [Source:HGNC Symbol;Acc:HGNC:16457]
**-1,531439072**	1,33E-150	3,27E-148	*ARSJ*	arylsulfatase family member J [Source:HGNC Symbol;Acc:HGNC:26286]
**-1,531224851**	1,77E-17	2,52E-16	*PIMREG*	PICALM interacting mitotic regulator [Source:HGNC Symbol;Acc:HGNC:25483]
**-1,530456672**	1,40E-55	7,13E-54	*INHBA*	inhibin subunit beta A [Source:HGNC Symbol;Acc:HGNC:6066]
**-1,527376819**	0,001399063	0,00513528	*CD180*	CD180 molecule [Source:HGNC Symbol;Acc:HGNC:6726]
**-1,526677509**	0,013995548	0,039800351	*FLRT1*	fibronectin leucine rich transmembrane protein 1 [Source:HGNC Symbol;Acc:HGNC:3760]
**-1,524090419**	6,91E-114	1,02E-111	*UAP1*	UDP-N-acetylglucosamine pyrophosphorylase 1 [Source:HGNC Symbol;Acc:HGNC:12457]
**-1,523449881**	0,001395887	0,005124591	*TACSTD2*	tumor associated calcium signal transducer 2 [Source:HGNC Symbol;Acc:HGNC:11530]
**-1,521169842**	4,50E-05	0,000218534	*IL4I1*	interleukin 4 induced 1 [Source:HGNC Symbol;Acc:HGNC:19094]
**-1,518449724**	3,40E-277	3,14E-274	*TPM2*	tropomyosin 2 [Source:HGNC Symbol;Acc:HGNC:12011]
**-1,518159419**	3,73E-82	3,26E-80	*VASN*	vasorin [Source:HGNC Symbol;Acc:HGNC:18517]
**-1,517781475**	1,09E-08	8,18E-08	*ZNF367*	zinc finger protein 367 [Source:HGNC Symbol;Acc:HGNC:18320]
**-1,516740152**	3,87E-12	3,91E-11	*ESCO2*	establishment of sister chromatid cohesion N-acetyltransferase 2 [Source:HGNC Symbol;Acc:HGNC:27230]

**-1,51436288**	0,009021106	0,02717113	*CALB2*	calbindin 2 [Source:HGNC Symbol;Acc:HGNC:1435]
**-1,51415209**	1,62E-33	4,50E-32	*ANOS1*	anosmin 1 [Source:HGNC Symbol;Acc:HGNC:6211]
**-1,51248538**	4,09E-89	4,09E-87	*CDO1*	cysteine dioxygenase type 1 [Source:HGNC Symbol;Acc:HGNC:1795]
**-1,510269074**	0,009519438	0,028451289	*CP*	ceruloplasmin [Source:HGNC Symbol;Acc:HGNC:2295]
**-1,510055742**	5,03E-17	6,96E-16	*SLC16A7*	solute carrier family 16 member 7 [Source:HGNC Symbol;Acc:HGNC:10928]
**-1,509170409**	6,71E-142	1,46E-139	*HEG1*	heart development protein with EGF like domains 1 [Source:HGNC Symbol;Acc:HGNC:29227]
**-1,508167982**	1,26E-08	9,43E-08	*CENPA*	centromere protein A [Source:HGNC Symbol;Acc:HGNC:1851]
**-1,506150811**	2,55E-09	2,02E-08	*POLQ*	DNA polymerase theta [Source:HGNC Symbol;Acc:HGNC:9186]
**-1,506016127**	2,11E-16	2,80E-15	*MAP3K21*	mitogen-activated protein kinase kinase kinase 21 [Source:HGNC Symbol;Acc:HGNC:29798]
**-1,505055195**	4,01E-23	7,37E-22	*CHAC1*	ChaC glutathione specific gamma-glutamylcyclotransferase 1 [Source:HGNC Symbol;Acc:HGNC:28680]
**-1,504011563**	6,12E-199	2,28E-196	*TNFRSF12A*	TNF receptor superfamily member 12A [Source:HGNC Symbol;Acc:HGNC:18152]
**-1,50242541**	3,88E-11	3,60E-10	*CENPM*	centromere protein M [Source:HGNC Symbol;Acc:HGNC:18352]
**-1,500467344**	8,85E-11	7,93E-10	*NOX4*	NADPH oxidase 4 [Source:HGNC Symbol;Acc:HGNC:7891]
**-1,499886722**	0,000197222	0,000852972	*SEMA3D*	semaphorin 3D [Source:HGNC Symbol;Acc:HGNC:10726]
**-1,499561437**	5,45E-77	4,22E-75	*NNMT*	nicotinamide N-methyltransferase [Source:HGNC Symbol;Acc:HGNC:7861]
**-1,499551968**	2,30E-48	9,86E-47	*RGS4*	regulator of G protein signaling 4 [Source:HGNC Symbol;Acc:HGNC:10000]
**-1,496161956**	1,51E-09	1,22E-08	*BEND4*	BEN domain containing 4 [Source:HGNC Symbol;Acc:HGNC:23815]
**-1,492798673**	4,46E-218	2,11E-215	*MCAM*	melanoma cell adhesion molecule [Source:HGNC Symbol;Acc:HGNC:6934]
**-1,491375957**	4,52E-14	5,28E-13	*CERKL*	ceramide kinase like [Source:HGNC Symbol;Acc:HGNC:21699]
**-1,487572998**	2,76E-25	5,60E-24	*UBE2C*	ubiquitin conjugating enzyme E2 C [Source:HGNC Symbol;Acc:HGNC:15937]
**-1,486455296**	9,15E-98	1,06E-95	*RSU1*	Ras suppressor protein 1 [Source:HGNC Symbol;Acc:HGNC:10464]
**-1,486258901**	0,01467092	0,041375363	*TNNT2*	troponin T2, cardiac type [Source:HGNC Symbol;Acc:HGNC:11949]
**-1,485029945**	1,83E-23	3,41E-22	*WNT2B*	Wnt family member 2B [Source:HGNC Symbol;Acc:HGNC:12781]
**-1,484677942**	2,64E-05	0,000132551	*SGO1*	shugoshin 1 [Source:HGNC Symbol;Acc:HGNC:25088]
**-1,484581098**	0,002301819	0,008055745	*MICB*	MHC class I polypeptide-related sequence B [Source:HGNC Symbol;Acc:HGNC:7091]
**-1,48446816**	5,87E-09	4,51E-08	*EXO1*	exonuclease 1 [Source:HGNC Symbol;Acc:HGNC:3511]
**-1,482939208**	0,000528073	0,00210903	*NEIL3*	nei like DNA glycosylase 3 [Source:HGNC Symbol;Acc:HGNC:24573]
**-1,480600139**	6,56E-05	0,000310148	*CCDC160*	coiled-coil domain containing 160 [Source:HGNC Symbol;Acc:HGNC:37286]
**-1,477604999**	9,70E-21	1,62E-19	*GINS1*	GINS complex subunit 1 [Source:HGNC Symbol;Acc:HGNC:28980]
**-1,476925215**	2,34E-22	4,17E-21	*KIF2C*	kinesin family member 2C [Source:HGNC Symbol;Acc:HGNC:6393]
**-1,471426624**	1,14E-23	2,14E-22	*TEAD4*	TEA domain transcription factor 4 [Source:HGNC Symbol;Acc:HGNC:11717]
**-1,470611434**	2,95E-11	2,77E-10	*CCDC3*	coiled-coil domain containing 3 [Source:HGNC Symbol;Acc:HGNC:23813]
**-1,469620274**	3,96E-16	5,16E-15	*SPTBN5*	spectrin beta, non-erythrocytic 5 [Source:HGNC Symbol;Acc:HGNC:15680]
**-1,468378167**	2,66E-283	2,58E-280	*CNN2*	calponin 2 [Source:HGNC Symbol;Acc:HGNC:2156]

**-1,467652426**	3,63E-48	1,56E-46	*DLC1*	DLC1 Rho GTPase activating protein [Source:HGNC Symbol;Acc:HGNC:2897]
**-1,463480021**	1,86E-51	8,49E-50	*ATP8B1*	ATPase phospholipid transporting 8B1 [Source:HGNC Symbol;Acc:HGNC:3706]
**-1,460900704**	1,96E-108	2,59E-106	*TGFB2*	transforming growth factor beta 2 [Source:HGNC Symbol;Acc:HGNC:11768]
**-1,459530992**	0,002162195	0,007616548	*CYP26B1*	cytochrome P450 family 26 subfamily B member 1 [Source:HGNC Symbol;Acc:HGNC:20581]
**-1,455854751**	8,06E-110	1,09E-107	*MMP15*	matrix metallopeptidase 15 [Source:HGNC Symbol;Acc:HGNC:7161]
**-1,454152776**	7,03E-26	1,47E-24	*SGMS2*	sphingomyelin synthase 2 [Source:HGNC Symbol;Acc:HGNC:28395]
**-1,453798349**	1,87E-06	1,10E-05	*HASPIN*	histone H3 associated protein kinase [Source:HGNC Symbol;Acc:HGNC:19682]
**-1,45267289**	1,82E-15	2,29E-14	*PI15*	peptidase inhibitor 15 [Source:HGNC Symbol;Acc:HGNC:8946]
**-1,44907822**	8,09E-16	1,04E-14	*FANCD2*	FA complementation group D2 [Source:HGNC Symbol;Acc:HGNC:3585]
**-1,44789246**	4,20E-31	1,07E-29	*ZWINT*	ZW10 interacting kinetochore protein [Source:HGNC Symbol;Acc:HGNC:13195]
**-1,447605446**	2,79E-09	2,21E-08	*TUNAR*	TCL1 upstream neural differentiation-associated RNA [Source:HGNC Symbol;Acc:HGNC:44088]
**-1,446752615**	2,44E-07	1,59E-06	*RAD54L*	RAD54 like [Source:HGNC Symbol;Acc:HGNC:9826]
**-1,445817704**	9,02E-36	2,69E-34	*GATA6*	GATA binding protein 6 [Source:HGNC Symbol;Acc:HGNC:4174]
**-1,44580098**	0,006125569	0,019369855	*PALM2-AKAP2*	PALM2-AKAP2 readthrough [Source:HGNC Symbol;Acc:HGNC:33529]
**-1,444997387**	2,50E-28	5,74E-27	*CD70*	CD70 molecule [Source:HGNC Symbol;Acc:HGNC:11937]
**-1,444065784**	0,000211903	0,000909577	*ECM2*	extracellular matrix protein 2 [Source:HGNC Symbol;Acc:HGNC:3154]
**-1,443262397**	4,91E-14	5,70E-13	*NUF2*	NUF2, NDC80 kinetochore complex component [Source:HGNC Symbol;Acc:HGNC:14621]
**-1,440418984**	1,79E-07	1,18E-06	*DES*	desmin [Source:HGNC Symbol;Acc:HGNC:2770]
**-1,439238766**	2,10E-224	1,10E-221	*ACTN1*	actinin alpha 1 [Source:HGNC Symbol;Acc:HGNC:163]
**-1,438379755**	0,006749874	0,021072723	*KCNJ3*	potassium voltage-gated channel subfamily J member 3 [Source:HGNC Symbol;Acc:HGNC:6264]
**-1,435339909**	7,07E-40	2,37E-38	*PLEKHA7*	pleckstrin homology domain containing A7 [Source:HGNC Symbol;Acc:HGNC:27049]
**-1,435165029**	0,000164401	0,000722294	*DIO2*	iodothyronine deiodinase 2 [Source:HGNC Symbol;Acc:HGNC:2884]
**-1,430323829**	8,81E-24	1,67E-22	*E2F1*	E2F transcription factor 1 [Source:HGNC Symbol;Acc:HGNC:3113]
**-1,428571418**	1,35E-05	7,02E-05	*ARHGDIB*	Rho GDP dissociation inhibitor beta [Source:HGNC Symbol;Acc:HGNC:679]
**-1,428144126**	8,51E-12	8,32E-11	*SRGN*	serglycin [Source:HGNC Symbol;Acc:HGNC:9361]
**-1,42730888**	1,16E-128	2,11E-126	*TUBB6*	tubulin beta 6 class V [Source:HGNC Symbol;Acc:HGNC:20776]
**-1,426397056**	1,01E-12	1,08E-11	*UBE2T*	ubiquitin conjugating enzyme E2 T [Source:HGNC Symbol;Acc:HGNC:25009]
**-1,423497843**	2,03E-87	1,98E-85	*SFRP2*	secreted frizzled related protein 2 [Source:HGNC Symbol;Acc:HGNC:10777]
**-1,421199082**	5,67E-33	1,55E-31	*PLK1*	polo like kinase 1 [Source:HGNC Symbol;Acc:HGNC:9077]
**-1,420452811**	1,26E-17	1,81E-16	*STRIP2*	striatin interacting protein 2 [Source:HGNC Symbol;Acc:HGNC:22209]
**-1,419311091**	1,37E-13	1,54E-12	*NDC80*	NDC80, kinetochore complex component [Source:HGNC Symbol;Acc:HGNC:16909]
**-1,41545509**	1,31E-13	1,48E-12	*TROAP*	trophinin associated protein [Source:HGNC Symbol;Acc:HGNC:12327]
**-1,413065942**	1,37E-28	3,18E-27	*CDCA5*	cell division cycle associated 5 [Source:HGNC Symbol;Acc:HGNC:14626]

**-1,412239682**	2,51E-150	6,01E-148	*RANGAP1*	Ran GTPase activating protein 1 [Source:HGNC Symbol;Acc:HGNC:9854]
**-1,41183406**	5,39E-29	1,27E-27	*STAC*	SH3 and cysteine rich domain [Source:HGNC Symbol;Acc:HGNC:11353]
**-1,410983746**	0,001675372	0,006029722	*AUNIP*	aurora kinase A and ninein interacting protein [Source:HGNC Symbol;Acc:HGNC:28363]
**-1,406770258**	2,10E-32	5,64E-31	*TK1*	thymidine kinase 1 [Source:HGNC Symbol;Acc:HGNC:11830]
**-1,405845474**	2,69E-39	8,90E-38	*TENM2*	teneurin transmembrane protein 2 [Source:HGNC Symbol;Acc:HGNC:29943]
**-1,40485372**	2,11E-27	4,65E-26	*VCAM1*	vascular cell adhesion molecule 1 [Source:HGNC Symbol;Acc:HGNC:12663]
**-1,402209206**	1,96E-14	2,33E-13	*WDR62*	WD repeat domain 62 [Source:HGNC Symbol;Acc:HGNC:24502]
**-1,397740372**	6,11E-24	1,17E-22	*MELK*	maternal embryonic leucine zipper kinase [Source:HGNC Symbol;Acc:HGNC:16870]
**-1,397121842**	3,38E-24	6,59E-23	*CDK1*	cyclin dependent kinase 1 [Source:HGNC Symbol;Acc:HGNC:1722]
**-1,396905116**	4,56E-40	1,54E-38	*NUAK2*	NUAK family kinase 2 [Source:HGNC Symbol;Acc:HGNC:29558]
**-1,396404054**	2,69E-85	2,50E-83	*PVR*	poliovirus receptor [Source:HGNC Symbol;Acc:HGNC:9705]
**-1,396024419**	3,68E-23	6,79E-22	*ELN*	elastin [Source:HGNC Symbol;Acc:HGNC:3327]
**-1,394559857**	2,48E-26	5,25E-25	*MUC1*	mucin 1, cell surface associated [Source:HGNC Symbol;Acc:HGNC:7508]
**-1,385134804**	2,19E-25	4,46E-24	*TBC1D2*	TBC1 domain family member 2 [Source:HGNC Symbol;Acc:HGNC:18026]
**-1,384499153**	9,72E-61	5,70E-59	*NTRK3*	neurotrophic receptor tyrosine kinase 3 [Source:HGNC Symbol;Acc:HGNC:8033]
**-1,381568957**	7,08E-12	6,95E-11	*HOXA3*	homeobox A3 [Source:HGNC Symbol;Acc:HGNC:5104]
**-1,380373303**	2,91E-30	7,22E-29	*KIF20A*	kinesin family member 20A [Source:HGNC Symbol;Acc:HGNC:9787]
**-1,379647312**	1,08E-238	6,76E-236	*CSRP1*	cysteine and glycine rich protein 1 [Source:HGNC Symbol;Acc:HGNC:2469]
**-1,376996809**	1,35E-25	2,80E-24	*BUB1B*	BUB1 mitotic checkpoint serine/threonine kinase B [Source:HGNC Symbol;Acc:HGNC:1149]
**-1,375680287**	1,55E-28	3,59E-27	*ARHGAP11A*	Rho GTPase activating protein 11A [Source:HGNC Symbol;Acc:HGNC:15783]
**-1,375112535**	1,80E-16	2,40E-15	*PBK*	PDZ binding kinase [Source:HGNC Symbol;Acc:HGNC:18282]
**-1,372483493**	1,94E-19	3,06E-18	*HJURP*	Holliday junction recognition protein [Source:HGNC Symbol;Acc:HGNC:25444]
**-1,371590642**	0,002663092	0,009179389	*ARMC4*	armadillo repeat containing 4 [Source:HGNC Symbol;Acc:HGNC:25583]
**-1,370326203**	5,44E-12	5,43E-11	*SLF1*	SMC5-SMC6 complex localization factor 1 [Source:HGNC Symbol;Acc:HGNC:25408]
**-1,369490344**	5,75E-07	3,59E-06	*POLA2*	DNA polymerase alpha 2, accessory subunit [Source:HGNC Symbol;Acc:HGNC:30073]
**-1,366434092**	1,31E-59	7,44E-58	*TPX2*	TPX2, microtubule nucleation factor [Source:HGNC Symbol;Acc:HGNC:1249]
**-1,364281278**	0,01200124	0,034860275	*IL6*	interleukin 6 [Source:HGNC Symbol;Acc:HGNC:6018]
**-1,364193987**	0,015470458	0,043359287	*TRIM55*	tripartite motif containing 55 [Source:HGNC Symbol;Acc:HGNC:14215]
**-1,363464708**	2,23E-05	0,000113083	*MGAT4C*	MGAT4 family member C [Source:HGNC Symbol;Acc:HGNC:30871]
**-1,362114822**	1,79E-54	8,89E-53	*ID3*	inhibitor of DNA binding 3, HLH protein [Source:HGNC Symbol;Acc:HGNC:5362]
**-1,359828687**	4,87E-79	3,99E-77	*TOP2A*	DNA topoisomerase II alpha [Source:HGNC Symbol;Acc:HGNC:11989]
**-1,359313929**	8,33E-07	5,12E-06	*SYDE2*	synapse defective Rho GTPase homolog 2 [Source:HGNC Symbol;Acc:HGNC:25841]

**-1,357001095**	3,49E-06	1,98E-05	*SKA1*	spindle and kinetochore associated complex subunit 1 [Source:HGNC Symbol;Acc:HGNC:28109]
**-1,355619171**	1,57E-09	1,26E-08	*A2M*	alpha-2-macroglobulin [Source:HGNC Symbol;Acc:HGNC:7]
**-1,354716325**	4,77E-11	4,38E-10	*HPD*	4-hydroxyphenylpyruvate dioxygenase [Source:HGNC Symbol;Acc:HGNC:5147]
**-1,351763471**	2,21E-104	2,82E-102	*RHOB*	ras homolog family member B [Source:HGNC Symbol;Acc:HGNC:668]
**-1,349742998**	1,74E-36	5,37E-35	*HACD1*	3-hydroxyacyl-CoA dehydratase 1 [Source:HGNC Symbol;Acc:HGNC:9639]
**-1,348904871**	0,000220013	0,000941262	*HOXA5*	homeobox A5 [Source:HGNC Symbol;Acc:HGNC:5106]
**-1,348611062**	4,58E-130	8,46E-128	*PDLIM5*	PDZ and LIM domain 5 [Source:HGNC Symbol;Acc:HGNC:17468]
**-1,348396397**	4,53E-97	5,20E-95	*LIF*	LIF, interleukin 6 family cytokine [Source:HGNC Symbol;Acc:HGNC:6596]
**-1,348244747**	6,32E-38	2,01E-36	*PAMR1*	peptidase domain containing associated with muscle regeneration 1 [Source:HGNC Symbol;Acc:HGNC:24554]
**-1,348180935**	1,13E-08	8,49E-08	*ABCB1*	ATP binding cassette subfamily B member 1 [Source:HGNC Symbol;Acc:HGNC:40]
**-1,345928719**	9,42E-08	6,44E-07	*DEPDC1B*	DEP domain containing 1B [Source:HGNC Symbol;Acc:HGNC:24902]
**-1,345505636**	0,005586567	0,017819347	*PABPC4L*	poly(A) binding protein cytoplasmic 4 like [Source:HGNC Symbol;Acc:HGNC:31955]
**-1,345012324**	1,96E-146	4,42E-144	*FSTL1*	follistatin like 1 [Source:HGNC Symbol;Acc:HGNC:3972]
**-1,344811134**	8,24E-97	9,41E-95	*PPME1*	protein phosphatase methylesterase 1 [Source:HGNC Symbol;Acc:HGNC:30178]
**-1,343154504**	7,05E-12	6,93E-11	*KIF15*	kinesin family member 15 [Source:HGNC Symbol;Acc:HGNC:17273]
**-1,342759508**	2,58E-07	1,68E-06	*PRELID2*	PRELI domain containing 2 [Source:HGNC Symbol;Acc:HGNC:28306]
**-1,341409773**	2,39E-64	1,53E-62	*FGF2*	fibroblast growth factor 2 [Source:HGNC Symbol;Acc:HGNC:3676]
**-1,340018072**	1,38E-74	1,02E-72	*IGFBP3*	insulin like growth factor binding protein 3 [Source:HGNC Symbol;Acc:HGNC:5472]
**-1,338984121**	1,38E-112	2,00E-110	*MYADM*	myeloid associated differentiation marker [Source:HGNC Symbol;Acc:HGNC:7544]
**-1,338521372**	3,85E-15	4,75E-14	*PPP1R13L*	protein phosphatase 1 regulatory subunit 13 like [Source:HGNC Symbol;Acc:HGNC:18838]
**-1,337044803**	1,70E-13	1,92E-12	*DEPDC1*	DEP domain containing 1 [Source:HGNC Symbol;Acc:HGNC:22949]
**-1,336263831**	1,75E-62	1,07E-60	*HACD2*	3-hydroxyacyl-CoA dehydratase 2 [Source:HGNC Symbol;Acc:HGNC:9640]
**-1,335802208**	6,48E-56	3,33E-54	*MTHFD2*	methylenetetrahydrofolate dehydrogenase (NADP+ dependent) 2, methenyltetrahydrofolate cyclohydrolase [Source:HGNC Symbol;Acc:HGNC:7434]
**-1,335724005**	5,97E-16	7,71E-15	*CD274*	CD274 molecule [Source:HGNC Symbol;Acc:HGNC:17635]
**-1,334614447**	9,16E-18	1,33E-16	*ELMO1*	engulfment and cell motility 1 [Source:HGNC Symbol;Acc:HGNC:16286]
**-1,330121183**	5,75E-06	3,17E-05	*SLC26A7*	solute carrier family 26 member 7 [Source:HGNC Symbol;Acc:HGNC:14467]
**-1,325145427**	8,15E-10	6,76E-09	*KIF18A*	kinesin family member 18A [Source:HGNC Symbol;Acc:HGNC:29441]
**-1,323033875**	0,000320213	0,001330063	*RARRES1*	retinoic acid receptor responder 1 [Source:HGNC Symbol;Acc:HGNC:9867]
**-1,322841355**	1,14E-20	1,90E-19	*NOCT*	nocturnin [Source:HGNC Symbol;Acc:HGNC:14254]
**-1,322695014**	1,42E-07	9,50E-07	*DEPP1*	DEPP1, autophagy regulator [Source:HGNC Symbol;Acc:HGNC:23355]
**-1,322626025**	7,31E-100	8,71E-98	*TMTC1*	transmembrane and tetratricopeptide repeat containing 1 [Source:HGNC Symbol;Acc:HGNC:24099]
**-1,320382441**	1,84E-103	2,32E-101	*CDC42EP1*	CDC42 effector protein 1 [Source:HGNC Symbol;Acc:HGNC:17014]

**-1,319761801**	9,33E-08	6,38E-07	*CYP46A1*	cytochrome P450 family 46 subfamily A member 1 [Source:HGNC Symbol;Acc:HGNC:2641]
**-1,319177252**	1,17E-239	7,58E-237	*PDLIM7*	PDZ and LIM domain 7 [Source:HGNC Symbol;Acc:HGNC:22958]
**-1,318801451**	1,67E-72	1,21E-70	*UHRF1*	ubiquitin like with PHD and ring finger domains 1 [Source:HGNC Symbol;Acc:HGNC:12556]
**-1,317957609**	3,75E-64	2,38E-62	*GLIPR2*	GLI pathogenesis related 2 [Source:HGNC Symbol;Acc:HGNC:18007]
**-1,317342316**	3,84E-85	3,54E-83	*CCDC80*	coiled-coil domain containing 80 [Source:HGNC Symbol;Acc:HGNC:30649]
**-1,310742268**	2,13E-18	3,18E-17	*ADAMTSL4*	ADAMTS like 4 [Source:HGNC Symbol;Acc:HGNC:19706]
**-1,308626838**	3,75E-06	2,11E-05	*CORO2A*	coronin 2A [Source:HGNC Symbol;Acc:HGNC:2255]
**-1,306672464**	0,005994193	0,018995137	*ZFP92*	ZFP92 zinc finger protein [Source:HGNC Symbol;Acc:HGNC:12865]
**-1,306318409**	0,000235691	0,00100303	*S100A3*	S100 calcium binding protein A3 [Source:HGNC Symbol;Acc:HGNC:10493]
**-1,305866972**	0,002457392	0,008550861	*CACNG5*	calcium voltage-gated channel auxiliary subunit gamma 5 [Source:HGNC Symbol;Acc:HGNC:1409]
**-1,304649766**	0,000541473	0,002158108	*RASL11B*	RAS like family 11 member B [Source:HGNC Symbol;Acc:HGNC:23804]
**-1,30418161**	3,63E-52	1,69E-50	*ALDH1B1*	aldehyde dehydrogenase 1 family member B1 [Source:HGNC Symbol;Acc:HGNC:407]
**-1,303816582**	3,10E-82	2,74E-80	*ARID5B*	AT-rich interaction domain 5B [Source:HGNC Symbol;Acc:HGNC:17362]
**-1,303563498**	2,09E-16	2,78E-15	*FBN1*	fibrillin 1 [Source:HGNC Symbol;Acc:HGNC:3603]
**-1,300533938**	3,38E-159	9,12E-157	*CAVIN1*	caveolae associated protein 1 [Source:HGNC Symbol;Acc:HGNC:9688]
**-1,300004282**	0,000328882	0,001362864	*STAC2*	SH3 and cysteine rich domain 2 [Source:HGNC Symbol;Acc:HGNC:23990]
**-1,299706197**	3,34E-05	0,00016506	*MTBP*	MDM2 binding protein [Source:HGNC Symbol;Acc:HGNC:7417]
**-1,297659052**	7,92E-98	9,25E-96	*NOL6*	nucleolar protein 6 [Source:HGNC Symbol;Acc:HGNC:19910]
**-1,29722846**	1,07E-48	4,61E-47	*ARRDC4*	arrestin domain containing 4 [Source:HGNC Symbol;Acc:HGNC:28087]
**-1,296852859**	1,93E-32	5,21E-31	*DOP1B*	DOP1 leucine zipper like protein B [Source:HGNC Symbol;Acc:HGNC:1291]
**-1,29585044**	1,06E-09	8,72E-09	*CPM*	carboxypeptidase M [Source:HGNC Symbol;Acc:HGNC:2311]
**-1,295763648**	2,87E-16	3,79E-15	*MOK*	MOK protein kinase [Source:HGNC Symbol;Acc:HGNC:9833]
**-1,295667149**	8,81E-12	8,60E-11	*CHAF1B*	chromatin assembly factor 1 subunit B [Source:HGNC Symbol;Acc:HGNC:1911]
**-1,294136621**	2,48E-127	4,30E-125	*LOXL2*	lysyl oxidase like 2 [Source:HGNC Symbol;Acc:HGNC:6666]
**-1,293965692**	9,43E-08	6,45E-07	*PSMC3IP*	PSMC3 interacting protein [Source:HGNC Symbol;Acc:HGNC:17928]
**-1,293217618**	5,82E-09	4,48E-08	*ZNF536*	zinc finger protein 536 [Source:HGNC Symbol;Acc:HGNC:29025]
**-1,29193549**	5,28E-59	2,94E-57	*PRPS1*	phosphoribosyl pyrophosphate synthetase 1 [Source:HGNC Symbol;Acc:HGNC:9462]
**-1,291422754**	2,05E-39	6,79E-38	*CDC20*	cell division cycle 20 [Source:HGNC Symbol;Acc:HGNC:1723]
**-1,29053992**	1,43E-31	3,73E-30	*GDPD5*	glycerophosphodiester phosphodiesterase domain containing 5 [Source:HGNC Symbol;Acc:HGNC:28804]
**-1,284753098**	0,014662148	0,041356635	*AC091057,6*	Rho GTPase-activating protein 11B [Source:UniProtKB/Swiss-Prot;Acc:Q3KRB8]
**-1,281400113**	3,56E-42	1,28E-40	*TRNP1*	TMF1-regulated nuclear protein 1 [Source:HGNC Symbol;Acc:HGNC:34348]
**-1,280357712**	2,02E-05	0,000103226	*SLCO1C1*	solute carrier organic anion transporter family member 1C1 [Source:HGNC Symbol;Acc:HGNC:13819]

**-1,279144202**	6,53E-31	1,66E-29	*KRT18*	keratin 18 [Source:HGNC Symbol;Acc:HGNC:6430]
**-1,278390466**	1,69E-40	5,78E-39	*ITGA4*	integrin subunit alpha 4 [Source:HGNC Symbol;Acc:HGNC:6140]
**-1,278003942**	4,60E-32	1,22E-30	*ATAD2*	ATPase family, AAA domain containing 2 [Source:HGNC Symbol;Acc:HGNC:30123]
**-1,277905227**	2,28E-08	1,66E-07	*NEK2*	NIMA related kinase 2 [Source:HGNC Symbol;Acc:HGNC:7745]
**-1,276827329**	3,01E-21	5,15E-20	*DLGAP5*	DLG associated protein 5 [Source:HGNC Symbol;Acc:HGNC:16864]
**-1,275595984**	2,81E-39	9,29E-38	*RFLNB*	refilin B [Source:HGNC Symbol;Acc:HGNC:28705]
**-1,270763648**	6,14E-10	5,15E-09	*PLK4*	polo like kinase 4 [Source:HGNC Symbol;Acc:HGNC:11397]
**-1,270405593**	0,018091819	0,04963102	*NKX2-2*	NK2 homeobox 2 [Source:HGNC Symbol;Acc:HGNC:7835]
**-1,268711381**	1,88E-51	8,60E-50	*CTPS1*	CTP synthase 1 [Source:HGNC Symbol;Acc:HGNC:2519]
**-1,267151097**	2,15E-12	2,23E-11	*WNK4*	WNK lysine deficient protein kinase 4 [Source:HGNC Symbol;Acc:HGNC:14544]
**-1,265419031**	1,26E-10	1,12E-09	*SMCO4*	single-pass membrane protein with coiled-coil domains 4 [Source:HGNC Symbol;Acc:HGNC:24810]
**-1,265183995**	0,00075037	0,002919341	*BORA*	bora, aurora kinase A activator [Source:HGNC Symbol;Acc:HGNC:24724]
**-1,26219184**	1,86E-05	9,50E-05	*CEP128*	centrosomal protein 128 [Source:HGNC Symbol;Acc:HGNC:20359]
**-1,261876481**	8,30E-75	6,20E-73	*PDLIM1*	PDZ and LIM domain 1 [Source:HGNC Symbol;Acc:HGNC:2067]
**-1,260455265**	3,44E-15	4,26E-14	*FST*	follistatin [Source:HGNC Symbol;Acc:HGNC:3971]
**-1,259259792**	1,06E-58	5,84E-57	*LIMCH1*	LIM and calponin homology domains 1 [Source:HGNC Symbol;Acc:HGNC:29191]
**-1,254637979**	3,97E-05	0,00019452	*CASZ1*	castor zinc finger 1 [Source:HGNC Symbol;Acc:HGNC:26002]
**-1,254615778**	2,46E-17	3,48E-16	*HELLS*	helicase, lymphoid specific [Source:HGNC Symbol;Acc:HGNC:4861]
**-1,254535196**	3,22E-09	2,54E-08	*WDR76*	WD repeat domain 76 [Source:HGNC Symbol;Acc:HGNC:25773]
**-1,254379991**	5,31E-28	1,20E-26	*TMEM178A*	transmembrane protein 178A [Source:HGNC Symbol;Acc:HGNC:28517]
**-1,250876765**	3,28E-12	3,34E-11	*ADAMTS16*	ADAM metallopeptidase with thrombospondin type 1 motif 16 [Source:HGNC Symbol;Acc:HGNC:17108]
**-1,250159956**	9,71E-31	2,45E-29	*BIRC5*	baculoviral IAP repeat containing 5 [Source:HGNC Symbol;Acc:HGNC:593]
**-1,250102272**	4,37E-121	6,84E-119	*CRIM1*	cysteine rich transmembrane BMP regulator 1 [Source:HGNC Symbol;Acc:HGNC:2359]
**-1,249783847**	1,21E-35	3,58E-34	*FLG*	filaggrin [Source:HGNC Symbol;Acc:HGNC:3748]
**-1,24930892**	0,000182638	0,000795751	*CCNE2*	cyclin E2 [Source:HGNC Symbol;Acc:HGNC:1590]
**-1,248539026**	1,30E-07	8,74E-07	*RAD51AP1*	RAD51 associated protein 1 [Source:HGNC Symbol;Acc:HGNC:16956]
**-1,248095966**	0,000677038	0,002659099	*CDK15*	cyclin dependent kinase 15 [Source:HGNC Symbol;Acc:HGNC:14434]
**-1,247127119**	7,15E-46	2,87E-44	*PSRC1*	proline and serine rich coiled-coil 1 [Source:HGNC Symbol;Acc:HGNC:24472]
**-1,247045845**	4,34E-21	7,37E-20	*PEAR1*	platelet endothelial aggregation receptor 1 [Source:HGNC Symbol;Acc:HGNC:33631]
**-1,246391265**	2,35E-41	8,26E-40	*PXDC1*	PX domain containing 1 [Source:HGNC Symbol;Acc:HGNC:21361]
**-1,246311208**	0,002326672	0,008132456	*NCKAP5*	NCK associated protein 5 [Source:HGNC Symbol;Acc:HGNC:29847]
**-1,244479457**	1,74E-228	9,95E-226	*ZYX*	zyxin [Source:HGNC Symbol;Acc:HGNC:13200]
**-1,242361555**	1,78E-05	9,15E-05	*TGM1*	transglutaminase 1 [Source:HGNC Symbol;Acc:HGNC:11777]
**-1,242285777**	1,96E-61	1,17E-59	*DUSP1*	dual specificity phosphatase 1 [Source:HGNC Symbol;Acc:HGNC:3064]

**-1,2408562**	1,11E-12	1,18E-11	*FAM83D*	family with sequence similarity 83 member D [Source:HGNC Symbol;Acc:HGNC:16122]
**-1,23792129**	4,65E-61	2,74E-59	*SYNPO*	synaptopodin [Source:HGNC Symbol;Acc:HGNC:30672]
**-1,233743262**	4,58E-05	0,000221945	*KCNE1B*	potassium voltage-gated channel subfamily E regulatory subunit 1B [Source:HGNC Symbol;Acc:HGNC:52280]
**-1,233153193**	0,000135627	0,000606026	*TRIM17*	tripartite motif containing 17 [Source:HGNC Symbol;Acc:HGNC:13430]
**-1,231304791**	3,07E-08	2,20E-07	*CALHM5*	calcium homeostasis modulator family member 5 [Source:HGNC Symbol;Acc:HGNC:21568]
**-1,226609614**	2,05E-14	2,43E-13	*SH2D4A*	SH2 domain containing 4A [Source:HGNC Symbol;Acc:HGNC:26102]
**-1,222756661**	3,19E-12	3,26E-11	*CKAP2L*	cytoskeleton associated protein 2 like [Source:HGNC Symbol;Acc:HGNC:26877]
**-1,220796211**	3,31E-14	3,90E-13	*ORC6*	origin recognition complex subunit 6 [Source:HGNC Symbol;Acc:HGNC:17151]
**-1,220666246**	4,97E-33	1,36E-31	*SPDL1*	spindle apparatus coiled-coil protein 1 [Source:HGNC Symbol;Acc:HGNC:26010]
**-1,219415137**	5,25E-54	2,57E-52	*LATS2*	large tumor suppressor kinase 2 [Source:HGNC Symbol;Acc:HGNC:6515]
**-1,218075237**	4,44E-82	3,86E-80	*EHD4*	EH domain containing 4 [Source:HGNC Symbol;Acc:HGNC:3245]
**-1,215516507**	1,51E-46	6,22E-45	*MFAP5*	microfibril associated protein 5 [Source:HGNC Symbol;Acc:HGNC:29673]
**-1,215367023**	6,01E-15	7,32E-14	*OCA2*	OCA2 melanosomal transmembrane protein [Source:HGNC Symbol;Acc:HGNC:8101]
**-1,214944828**	3,43E-12	3,48E-11	*CLSPN*	claspin [Source:HGNC Symbol;Acc:HGNC:19715]
**-1,214614273**	3,58E-07	2,29E-06	*ST6GALNAC3*	ST6 N-acetylgalactosaminide alpha-2,6-sialyltransferase 3 [Source:HGNC Symbol;Acc:HGNC:19343]
**-1,213093238**	1,08E-70	7,55E-69	*SCARF2*	scavenger receptor class F member 2 [Source:HGNC Symbol;Acc:HGNC:19869]
**-1,212847236**	1,76E-10	1,55E-09	*TTK*	TTK protein kinase [Source:HGNC Symbol;Acc:HGNC:12401]
**-1,210801563**	5,12E-60	2,94E-58	*RRAS2*	RAS related 2 [Source:HGNC Symbol;Acc:HGNC:17271]
**-1,210677191**	8,58E-10	7,10E-09	*TMEM255B*	transmembrane protein 255B [Source:HGNC Symbol;Acc:HGNC:28297]
**-1,209026982**	1,54E-19	2,44E-18	*COL5A1*	collagen type V alpha 1 chain [Source:HGNC Symbol;Acc:HGNC:2209]
**-1,206261021**	3,42E-07	2,19E-06	*CENPW*	centromere protein W [Source:HGNC Symbol;Acc:HGNC:21488]
**-1,201187373**	5,69E-05	0,000272127	*NEXMIF*	neurite extension and migration factor [Source:HGNC Symbol;Acc:HGNC:29433]
**-1,199404099**	1,49E-32	4,04E-31	*ALDH1L2*	aldehyde dehydrogenase 1 family member L2 [Source:HGNC Symbol;Acc:HGNC:26777]
**-1,19365711**	8,66E-12	8,46E-11	*DRD4*	dopamine receptor D4 [Source:HGNC Symbol;Acc:HGNC:3025]
**-1,192787866**	3,59E-56	1,85E-54	*NCEH1*	neutral cholesterol ester hydrolase 1 [Source:HGNC Symbol;Acc:HGNC:29260]
**-1,191493091**	7,90E-09	6,01E-08	*MRVI1*	murine retrovirus integration site 1 homolog [Source:HGNC Symbol;Acc:HGNC:7237]
**-1,190413543**	6,28E-17	8,64E-16	*C5orf30*	chromosome 5 open reading frame 30 [Source:HGNC Symbol;Acc:HGNC:25052]
**-1,188832101**	2,93E-17	4,13E-16	*AURKA*	aurora kinase A [Source:HGNC Symbol;Acc:HGNC:11393]
**-1,188470035**	2,97E-19	4,64E-18	*DSP*	desmoplakin [Source:HGNC Symbol;Acc:HGNC:3052]
**-1,187703155**	1,87E-19	2,95E-18	*SLC8A1*	solute carrier family 8 member A1 [Source:HGNC Symbol;Acc:HGNC:11068]
**-1,186784343**	0,016829454	0,04668273	*EEF1AKMT4*	EEF1A lysine methyltransferase 4 [Source:HGNC Symbol;Acc:HGNC:53611]
**-1,185395552**	0,000614741	0,002431153	*HECW1*	HECT, C2 and WW domain containing E3 ubiquitin protein ligase 1 [Source:HGNC Symbol;Acc:HGNC:22195]
**-1,184358608**	5,00E-09	3,87E-08	*MATN3*	matrilin 3 [Source:HGNC Symbol;Acc:HGNC:6909]

**-1,181767998**	0,006962057	0,021630752	*SLC17A9*	solute carrier family 17 member 9 [Source:HGNC Symbol;Acc:HGNC:16192]
**-1,181151356**	0,00129331	0,004775108	*GPR63*	G protein-coupled receptor 63 [Source:HGNC Symbol;Acc:HGNC:13302]
**-1,180369863**	4,38E-22	7,71E-21	*SEMA3B*	semaphorin 3B [Source:HGNC Symbol;Acc:HGNC:10724]
**-1,17946834**	1,08E-45	4,30E-44	*PAPLN*	papilin, proteoglycan like sulfated glycoprotein [Source:HGNC Symbol;Acc:HGNC:19262]
**-1,178409597**	4,23E-55	2,13E-53	*NREP*	neuronal regeneration related protein [Source:HGNC Symbol;Acc:HGNC:16834]
**-1,177550022**	5,66E-42	2,02E-40	*HOPX*	HOP homeobox [Source:HGNC Symbol;Acc:HGNC:24961]
**-1,174252675**	5,12E-107	6,63E-105	*MYH9*	myosin heavy chain 9 [Source:HGNC Symbol;Acc:HGNC:7579]
**-1,171533448**	1,45E-41	5,14E-40	*LMNB1*	lamin B1 [Source:HGNC Symbol;Acc:HGNC:6637]
**-1,169783978**	1,42E-95	1,58E-93	*SRF*	serum response factor [Source:HGNC Symbol;Acc:HGNC:11291]
**-1,169002196**	7,86E-55	3,92E-53	*STX12*	syntaxin 12 [Source:HGNC Symbol;Acc:HGNC:11430]
**-1,167450511**	2,99E-133	5,79E-131	*FOSL2*	FOS like 2, AP-1 transcription factor subunit [Source:HGNC Symbol;Acc:HGNC:3798]
**-1,16634237**	4,98E-10	4,21E-09	*ARSI*	arylsulfatase family member I [Source:HGNC Symbol;Acc:HGNC:32521]
**-1,166000911**	4,73E-11	4,35E-10	*KNL1*	kinetochore scaffold 1 [Source:HGNC Symbol;Acc:HGNC:24054]
**-1,164174358**	4,32E-48	1,84E-46	*C11orf24*	chromosome 11 open reading frame 24 [Source:HGNC Symbol;Acc:HGNC:1174]
**-1,163545514**	2,71E-05	0,000135903	*BRCA2*	BRCA2, DNA repair associated [Source:HGNC Symbol;Acc:HGNC:1101]
**-1,163370551**	4,13E-14	4,83E-13	*CRISPLD1*	cysteine rich secretory protein LCCL domain containing 1 [Source:HGNC Symbol;Acc:HGNC:18206]
**-1,159714468**	2,78E-41	9,72E-40	*FBLN5*	fibulin 5 [Source:HGNC Symbol;Acc:HGNC:3602]
**-1,158179893**	1,10E-105	1,42E-103	*TLN1*	talin 1 [Source:HGNC Symbol;Acc:HGNC:11845]
**-1,15810865**	4,87E-06	2,70E-05	*TPH1*	tryptophan hydroxylase 1 [Source:HGNC Symbol;Acc:HGNC:12008]
**-1,156996581**	2,88E-24	5,62E-23	*RECQL4*	RecQ like helicase 4 [Source:HGNC Symbol;Acc:HGNC:9949]
**-1,155289578**	2,87E-08	2,07E-07	*BMPER*	BMP binding endothelial regulator [Source:HGNC Symbol;Acc:HGNC:24154]
**-1,154572037**	5,91E-11	5,36E-10	*FANCA*	FA complementation group A [Source:HGNC Symbol;Acc:HGNC:3582]
**-1,15336218**	1,39E-07	9,29E-07	*C3orf52*	chromosome 3 open reading frame 52 [Source:HGNC Symbol;Acc:HGNC:26255]
**-1,153252691**	3,64E-52	1,70E-50	*ETS1*	ETS proto-oncogene 1, transcription factor [Source:HGNC Symbol;Acc:HGNC:3488]
**-1,152206315**	0,006221564	0,019638202	*C4B*	complement C4B (Chido blood group) [Source:HGNC Symbol;Acc:HGNC:1324]
**-1,150615361**	1,50E-05	7,77E-05	*GDNF*	glial cell derived neurotrophic factor [Source:HGNC Symbol;Acc:HGNC:4232]
**-1,14994443**	4,46E-77	3,46E-75	*SPOCD1*	SPOC domain containing 1 [Source:HGNC Symbol;Acc:HGNC:26338]
**-1,149750436**	0,007781574	0,023856364	*AC068831,7*	novel transcript
**-1,149373933**	2,97E-06	1,70E-05	*C1orf112*	chromosome 1 open reading frame 112 [Source:HGNC Symbol;Acc:HGNC:25565]
**-1,147926021**	3,58E-123	5,80E-121	*TOM1L2*	target of myb1 like 2 membrane trafficking protein [Source:HGNC Symbol;Acc:HGNC:11984]
**-1,147817731**	1,15E-12	1,22E-11	*ZNF93*	zinc finger protein 93 [Source:HGNC Symbol;Acc:HGNC:13169]
**-1,146882971**	8,78E-111	1,20E-108	*TIMP3*	TIMP metallopeptidase inhibitor 3 [Source:HGNC Symbol;Acc:HGNC:11822]
**-1,146779101**	0,016926069	0,046877023	*HGF*	hepatocyte growth factor [Source:HGNC Symbol;Acc:HGNC:4893]
**-1,146655703**	1,84E-08	1,35E-07	*LSM11*	LSM11, U7 small nuclear RNA associated [Source:HGNC Symbol;Acc:HGNC:30860]

**-1,145323416**	3,97E-73	2,90E-71	*PAWR*	pro-apoptotic WT1 regulator [Source:HGNC Symbol;Acc:HGNC:8614]
**-1,144492726**	4,94E-17	6,84E-16	*MAD2L1*	mitotic arrest deficient 2 like 1 [Source:HGNC Symbol;Acc:HGNC:6763]
**-1,144290447**	1,49E-80	1,25E-78	*RAI14*	retinoic acid induced 14 [Source:HGNC Symbol;Acc:HGNC:14873]
**-1,142617964**	6,19E-07	3,86E-06	*ORC1*	origin recognition complex subunit 1 [Source:HGNC Symbol;Acc:HGNC:8487]
**-1,140027556**	7,21E-05	0,000338113	*ZNF850*	zinc finger protein 850 [Source:HGNC Symbol;Acc:HGNC:27994]
**-1,139272063**	0,000809884	0,003130799	*DKK2*	dickkopf WNT signaling pathway inhibitor 2 [Source:HGNC Symbol;Acc:HGNC:2892]
**-1,138252533**	0,001083853	0,004068327	*CHRNA5*	cholinergic receptor nicotinic alpha 5 subunit [Source:HGNC Symbol;Acc:HGNC:1959]
**-1,13821358**	4,35E-36	1,31E-34	*WNT5B*	Wnt family member 5B [Source:HGNC Symbol;Acc:HGNC:16265]
**-1,137739327**	1,54E-16	2,06E-15	*WDR86*	WD repeat domain 86 [Source:HGNC Symbol;Acc:HGNC:28020]
**-1,134958915**	5,46E-54	2,67E-52	*PLEKHG4B*	pleckstrin homology and RhoGEF domain containing G4B [Source:HGNC Symbol;Acc:HGNC:29399]
**-1,132453128**	5,14E-54	2,52E-52	*GFRA1*	GDNF family receptor alpha 1 [Source:HGNC Symbol;Acc:HGNC:4243]
**-1,129978114**	3,73E-165	1,10E-162	*PLK2*	polo like kinase 2 [Source:HGNC Symbol;Acc:HGNC:19699]
**-1,12953532**	0,000949954	0,003607596	*SLC44A3*	solute carrier family 44 member 3 [Source:HGNC Symbol;Acc:HGNC:28689]
**-1,125772625**	0,00036105	0,001486323	*ULBP2*	UL16 binding protein 2 [Source:HGNC Symbol;Acc:HGNC:14894]
**-1,123949376**	8,70E-36	2,60E-34	*HBEGF*	heparin binding EGF like growth factor [Source:HGNC Symbol;Acc:HGNC:3059]
**-1,123845134**	7,01E-101	8,61E-99	*TGFB1I1*	transforming growth factor beta 1 induced transcript 1 [Source:HGNC Symbol;Acc:HGNC:11767]
**-1,122851317**	0,001281939	0,004736732	*HTR1B*	5-hydroxytryptamine receptor 1B [Source:HGNC Symbol;Acc:HGNC:5287]
**-1,121616175**	1,01E-12	1,08E-11	*EFHD1*	EF-hand domain family member D1 [Source:HGNC Symbol;Acc:HGNC:29556]
**-1,121491831**	1,72E-20	2,83E-19	*ASPM*	abnormal spindle microtubule assembly [Source:HGNC Symbol;Acc:HGNC:19048]
**-1,117927812**	1,33E-16	1,79E-15	*RRAD*	RRAD, Ras related glycolysis inhibitor and calcium channel regulator [Source:HGNC Symbol;Acc:HGNC:10446]
**-1,117578783**	2,83E-64	1,80E-62	*LBR*	lamin B receptor [Source:HGNC Symbol;Acc:HGNC:6518]
**-1,116015399**	3,64E-24	7,05E-23	*PRICKLE1*	prickle planar cell polarity protein 1 [Source:HGNC Symbol;Acc:HGNC:17019]
**-1,115644198**	4,15E-30	1,02E-28	*MCM6*	minichromosome maintenance complex component 6 [Source:HGNC Symbol;Acc:HGNC:6949]
**-1,11486429**	0,000909858	0,00347436	*NUSAP1*	nucleolar and spindle associated protein 1 [Source:HGNC Symbol;Acc:HGNC:18538]
**-1,113631544**	2,57E-11	2,43E-10	*KIF20B*	kinesin family member 20B [Source:HGNC Symbol;Acc:HGNC:7212]
**-1,112881679**	9,32E-12	9,08E-11	*COL1A1*	collagen type I alpha 1 chain [Source:HGNC Symbol;Acc:HGNC:2197]
**-1,111926935**	2,13E-09	1,70E-08	*ERFE*	erythroferrone [Source:HGNC Symbol;Acc:HGNC:26727]
**-1,111788662**	9,44E-71	6,64E-69	*FLNB*	filamin B [Source:HGNC Symbol;Acc:HGNC:3755]
**-1,111191222**	9,45E-10	7,78E-09	*IL1RAP*	interleukin 1 receptor accessory protein [Source:HGNC Symbol;Acc:HGNC:5995]
**-1,11008318**	1,72E-17	2,46E-16	*CXCL8*	C-X-C motif chemokine ligand 8 [Source:HGNC Symbol;Acc:HGNC:6025]
**-1,109867497**	6,19E-22	1,08E-20	*PRSS12*	serine protease 12 [Source:HGNC Symbol;Acc:HGNC:9477]
**-1,108511202**	2,70E-22	4,79E-21	*ATOH8*	atonal bHLH transcription factor 8 [Source:HGNC Symbol;Acc:HGNC:24126]

**-1,108409132**	5,93E-11	5,38E-10	*FRAS1*	Fraser extracellular matrix complex subunit 1 [Source:HGNC Symbol;Acc:HGNC:19185]
**-1,106415336**	5,87E-38	1,87E-36	*NABP1*	nucleic acid binding protein 1 [Source:HGNC Symbol;Acc:HGNC:26232]
**-1,102655601**	3,14E-34	8,94E-33	*PLCE1*	phospholipase C epsilon 1 [Source:HGNC Symbol;Acc:HGNC:17175]
**-1,102205382**	6,42E-34	1,81E-32	*INAFM2*	InaF motif containing 2 [Source:HGNC Symbol;Acc:HGNC:35165]
**-1,100574567**	1,31E-05	6,86E-05	*SPC25*	SPC25, NDC80 kinetochore complex component [Source:HGNC Symbol;Acc:HGNC:24031]
**-1,100154473**	1,88E-46	7,71E-45	*PPRC1*	peroxisome proliferator-activated receptor gamma, coactivator-related 1 [Source:HGNC Symbol;Acc:HGNC:30025]
**-1,099551851**	4,04E-124	6,71E-122	*PALLD*	palladin, cytoskeletal associated protein [Source:HGNC Symbol;Acc:HGNC:17068]
**-1,098990361**	1,94E-13	2,17E-12	*FNDC1*	fibronectin type III domain containing 1 [Source:HGNC Symbol;Acc:HGNC:21184]
**-1,096993098**	7,56E-19	1,16E-17	*CEP78*	centrosomal protein 78 [Source:HGNC Symbol;Acc:HGNC:25740]
**-1,095537131**	3,19E-71	2,28E-69	*AFAP1*	actin filament associated protein 1 [Source:HGNC Symbol;Acc:HGNC:24017]
**-1,095165213**	7,70E-11	6,93E-10	*DCLRE1B*	DNA cross-link repair 1B [Source:HGNC Symbol;Acc:HGNC:17641]
**-1,093985939**	2,95E-30	7,32E-29	*CDR2*	cerebellar degeneration related protein 2 [Source:HGNC Symbol;Acc:HGNC:1799]
**-1,090611958**	1,76E-08	1,30E-07	*RTKN2*	rhotekin 2 [Source:HGNC Symbol;Acc:HGNC:19364]
**-1,089032589**	2,33E-81	2,00E-79	*SORT1*	sortilin 1 [Source:HGNC Symbol;Acc:HGNC:11186]
**-1,088756267**	2,33E-10	2,03E-09	*STIL*	STIL, centriolar assembly protein [Source:HGNC Symbol;Acc:HGNC:10879]
**-1,088751997**	2,09E-06	1,22E-05	*DDIAS*	DNA damage induced apoptosis suppressor [Source:HGNC Symbol;Acc:HGNC:26351]
**-1,087134461**	0,000323897	0,001344216	*SLC6A11*	solute carrier family 6 member 11 [Source:HGNC Symbol;Acc:HGNC:11044]
**-1,086511194**	0,000214898	0,000921411	*TRPC4*	transient receptor potential cation channel subfamily C member 4 [Source:HGNC Symbol;Acc:HGNC:12336]
**-1,085802111**	4,26E-54	2,10E-52	*VWA1*	von Willebrand factor A domain containing 1 [Source:HGNC Symbol;Acc:HGNC:30910]
**-1,084697324**	1,54E-10	1,36E-09	*GABRE*	gamma-aminobutyric acid type A receptor epsilon subunit [Source:HGNC Symbol;Acc:HGNC:4085]
**-1,083395776**	1,44E-10	1,28E-09	*CENPU*	centromere protein U [Source:HGNC Symbol;Acc:HGNC:21348]
**-1,083184448**	0,017481385	0,048188034	*LDB3*	LIM domain binding 3 [Source:HGNC Symbol;Acc:HGNC:15710]
**-1,08144855**	2,17E-52	1,02E-50	*SLC16A3*	solute carrier family 16 member 3 [Source:HGNC Symbol;Acc:HGNC:10924]
**-1,08109903**	7,18E-08	4,97E-07	*BCL2*	BCL2, apoptosis regulator [Source:HGNC Symbol;Acc:HGNC:990]
**-1,080028234**	8,15E-136	1,63E-133	*TPM4*	tropomyosin 4 [Source:HGNC Symbol;Acc:HGNC:12013]
**-1,078643391**	6,70E-07	4,16E-06	*KIAA0895*	KIAA0895 [Source:HGNC Symbol;Acc:HGNC:22206]
**-1,078548383**	2,18E-42	7,89E-41	*NTN4*	netrin 4 [Source:HGNC Symbol;Acc:HGNC:13658]
**-1,077677124**	1,16E-24	2,29E-23	*PIM1*	Pim-1 proto-oncogene, serine/threonine kinase [Source:HGNC Symbol;Acc:HGNC:8986]
**-1,077148508**	4,13E-34	1,17E-32	*FHOD3*	formin homology 2 domain containing 3 [Source:HGNC Symbol;Acc:HGNC:26178]
**-1,077135298**	1,81E-15	2,28E-14	*STXBP6*	syntaxin binding protein 6 [Source:HGNC Symbol;Acc:HGNC:19666]
**-1,075521353**	1,48E-25	3,05E-24	*POLE*	DNA polymerase epsilon, catalytic subunit [Source:HGNC Symbol;Acc:HGNC:9177]
**-1,074226789**	4,73E-18	6,95E-17	*BRINP1*	BMP/retinoic acid inducible neural specific 1 [Source:HGNC Symbol;Acc:HGNC:2687]
**-1,073622582**	1,24E-17	1,79E-16	*CXCL6*	C-X-C motif chemokine ligand 6 [Source:HGNC Symbol;Acc:HGNC:10643]

**-1,072798384**	3,29E-05	0,000163203	*CEP152*	centrosomal protein 152 [Source:HGNC Symbol;Acc:HGNC:29298]
**-1,072045207**	2,85E-83	2,56E-81	*TNS3*	tensin 3 [Source:HGNC Symbol;Acc:HGNC:21616]
**-1,071849874**	7,16E-11	6,47E-10	*KIF14*	kinesin family member 14 [Source:HGNC Symbol;Acc:HGNC:19181]
**-1,070193779**	3,22E-17	4,52E-16	*SPAG5*	sperm associated antigen 5 [Source:HGNC Symbol;Acc:HGNC:13452]
**-1,069223791**	1,76E-47	7,39E-46	*SOCS3*	suppressor of cytokine signaling 3 [Source:HGNC Symbol;Acc:HGNC:19391]
**-1,068985287**	1,80E-44	6,91E-43	*NTN1*	netrin 1 [Source:HGNC Symbol;Acc:HGNC:8029]
**-1,068754847**	5,37E-34	1,51E-32	*SYNC*	syncoilin, intermediate filament protein [Source:HGNC Symbol;Acc:HGNC:28897]
**-1,067734561**	2,15E-08	1,57E-07	*IMMP2L*	inner mitochondrial membrane peptidase subunit 2 [Source:HGNC Symbol;Acc:HGNC:14598]
**-1,065114511**	7,40E-30	1,80E-28	*CENPF*	centromere protein F [Source:HGNC Symbol;Acc:HGNC:1857]
**-1,064551268**	2,39E-21	4,09E-20	*AMPH*	amphiphysin [Source:HGNC Symbol;Acc:HGNC:471]
**-1,063667597**	0,003753844	0,012475955	*PCLAF*	PCNA clamp associated factor [Source:HGNC Symbol;Acc:HGNC:28961]
**-1,062854316**	5,27E-15	6,48E-14	*BNC1*	basonuclin 1 [Source:HGNC Symbol;Acc:HGNC:1081]
**-1,057977904**	6,18E-06	3,39E-05	*LRR1*	leucine rich repeat protein 1 [Source:HGNC Symbol;Acc:HGNC:19742]
**-1,057008784**	9,12E-15	1,10E-13	*DACT1*	dishevelled binding antagonist of beta catenin 1 [Source:HGNC Symbol;Acc:HGNC:17748]
**-1,056743066**	6,94E-38	2,20E-36	*TMEM109*	transmembrane protein 109 [Source:HGNC Symbol;Acc:HGNC:28771]
**-1,056440145**	0,001578695	0,00571357	*ARHGEF39*	Rho guanine nucleotide exchange factor 39 [Source:HGNC Symbol;Acc:HGNC:25909]
**-1,055952646**	6,95E-10	5,80E-09	*CDCA2*	cell division cycle associated 2 [Source:HGNC Symbol;Acc:HGNC:14623]
**-1,054772681**	2,23E-52	1,05E-50	*TMTC4*	transmembrane and tetratricopeptide repeat containing 4 [Source:HGNC Symbol;Acc:HGNC:25904]
**-1,052072828**	5,22E-05	0,000251057	*CEACAM19*	carcinoembryonic antigen related cell adhesion molecule 19 [Source:HGNC Symbol;Acc:HGNC:31951]
**-1,051736049**	3,44E-30	8,48E-29	*PRKAG2*	protein kinase AMP-activated non-catalytic subunit gamma 2 [Source:HGNC Symbol;Acc:HGNC:9386]
**-1,051479627**	8,87E-34	2,47E-32	*C9orf3*	chromosome 9 open reading frame 3 [Source:HGNC Symbol;Acc:HGNC:1361]
**-1,050075175**	1,14E-11	1,10E-10	*SCUBE3*	signal peptide, CUB domain and EGF like domain containing 3 [Source:HGNC Symbol;Acc:HGNC:13655]
**-1,049744611**	5,91E-10	4,96E-09	*PLD5*	phospholipase D family member 5 [Source:HGNC Symbol;Acc:HGNC:26879]
**-1,048327414**	1,10E-72	8,05E-71	*KPNA2*	karyopherin subunit alpha 2 [Source:HGNC Symbol;Acc:HGNC:6395]
**-1,048141345**	0,000180256	0,000786078	*TEK*	TEK receptor tyrosine kinase [Source:HGNC Symbol;Acc:HGNC:11724]
**-1,047280952**	5,82E-08	4,07E-07	*ADAMTS6*	ADAM metallopeptidase with thrombospondin type 1 motif 6 [Source:HGNC Symbol;Acc:HGNC:222]
**-1,045971314**	3,44E-09	2,71E-08	*HMMR*	hyaluronan mediated motility receptor [Source:HGNC Symbol;Acc:HGNC:5012]
**-1,045372296**	8,14E-21	1,36E-19	*KNSTRN*	kinetochore localized astrin (SPAG5) binding protein [Source:HGNC Symbol;Acc:HGNC:30767]
**-1,043742081**	3,40E-19	5,31E-18	*PCK2*	phosphoenolpyruvate carboxykinase 2, mitochondrial [Source:HGNC Symbol;Acc:HGNC:8725]
**-1,043307551**	0,00098618	0,003733479	*GINS3*	GINS complex subunit 3 [Source:HGNC Symbol;Acc:HGNC:25851]
**-1,042873269**	0,011043238	0,032411536	*PRIM1*	DNA primase subunit 1 [Source:HGNC Symbol;Acc:HGNC:9369]
**-1,042450978**	5,42E-60	3,10E-58	*SYDE1*	synapse defective Rho GTPase homolog 1 [Source:HGNC Symbol;Acc:HGNC:25824]
**-1,042245629**	3,77E-23	6,94E-22	*SERAC1*	serine active site containing 1 [Source:HGNC Symbol;Acc:HGNC:21061]

**-1,040166259**	6,41E-24	1,23E-22	*TRAF5*	TNF receptor associated factor 5 [Source:HGNC Symbol;Acc:HGNC:12035]
**-1,039476106**	5,47E-167	1,69E-164	*ACTB*	actin beta [Source:HGNC Symbol;Acc:HGNC:132]
**-1,038497037**	1,60E-37	5,03E-36	*MCM5*	minichromosome maintenance complex component 5 [Source:HGNC Symbol;Acc:HGNC:6948]
**-1,038329206**	2,20E-24	4,32E-23	*EVA1A*	eva-1 homolog A, regulator of programmed cell death [Source:HGNC Symbol;Acc:HGNC:25816]
**-1,038032489**	8,45E-24	1,61E-22	*NCAPG2*	non-SMC condensin II complex subunit G2 [Source:HGNC Symbol;Acc:HGNC:21904]
**-1,0376445**	2,25E-32	6,01E-31	*DAB2*	DAB2, clathrin adaptor protein [Source:HGNC Symbol;Acc:HGNC:2662]
**-1,037532375**	5,70E-50	2,56E-48	*KCNK2*	potassium two pore domain channel subfamily K member 2 [Source:HGNC Symbol;Acc:HGNC:6277]
**-1,036622103**	2,88E-107	3,75E-105	*LOX*	lysyl oxidase [Source:HGNC Symbol;Acc:HGNC:6664]
**-1,035797819**	5,73E-23	1,05E-21	*ECT2*	epithelial cell transforming 2 [Source:HGNC Symbol;Acc:HGNC:3155]
**-1,035181149**	4,52E-19	6,99E-18	*HIVEP2*	human immunodeficiency virus type I enhancer binding protein 2 [Source:HGNC Symbol;Acc:HGNC:4921]
**-1,034830193**	1,32E-28	3,08E-27	*PKP4*	plakophilin 4 [Source:HGNC Symbol;Acc:HGNC:9026]
**-1,033117005**	2,59E-09	2,06E-08	*RAB3B*	RAB3B, member RAS oncogene family [Source:HGNC Symbol;Acc:HGNC:9778]
**-1,032561213**	2,18E-113	3,18E-111	*LDHA*	lactate dehydrogenase A [Source:HGNC Symbol;Acc:HGNC:6535]
**-1,032401607**	3,89E-37	1,22E-35	*C12orf75*	chromosome 12 open reading frame 75 [Source:HGNC Symbol;Acc:HGNC:35164]
**-1,031693176**	0,002488563	0,008636096	*AQP3*	aquaporin 3 (Gill blood group) [Source:HGNC Symbol;Acc:HGNC:636]
**-1,02832701**	1,04E-76	7,98E-75	*SLC38A1*	solute carrier family 38 member 1 [Source:HGNC Symbol;Acc:HGNC:13447]
**-1,028241868**	7,37E-13	7,95E-12	*KIF4A*	kinesin family member 4A [Source:HGNC Symbol;Acc:HGNC:13339]
**-1,027377705**	8,40E-18	1,22E-16	*KIF11*	kinesin family member 11 [Source:HGNC Symbol;Acc:HGNC:6388]
**-1,026857138**	9,71E-79	7,88E-77	*MICALL1*	MICAL like 1 [Source:HGNC Symbol;Acc:HGNC:29804]
**-1,026062022**	3,74E-27	8,13E-26	*WTIP*	WT1 interacting protein [Source:HGNC Symbol;Acc:HGNC:20964]
**-1,025714784**	1,24E-56	6,54E-55	*GLS*	glutaminase [Source:HGNC Symbol;Acc:HGNC:4331]
**-1,024709959**	2,73E-60	1,58E-58	*CYTH3*	cytohesin 3 [Source:HGNC Symbol;Acc:HGNC:9504]
**-1,024391899**	2,86E-13	3,17E-12	*CCDC9B*	coiled-coil domain containing 9B [Source:HGNC Symbol;Acc:HGNC:33488]
**-1,024391276**	1,32E-15	1,68E-14	*WNK2*	WNK lysine deficient protein kinase 2 [Source:HGNC Symbol;Acc:HGNC:14542]
**-1,023608025**	3,83E-69	2,61E-67	*CSF1*	colony stimulating factor 1 [Source:HGNC Symbol;Acc:HGNC:2432]
**-1,022756467**	0,00183443	0,006548741	*EME1*	essential meiotic structure-specific endonuclease 1 [Source:HGNC Symbol;Acc:HGNC:24965]
**-1,021774873**	5,33E-11	4,87E-10	*RAP1GAP2*	RAP1 GTPase activating protein 2 [Source:HGNC Symbol;Acc:HGNC:29176]
**-1,017564569**	9,24E-22	1,61E-20	*ZNF469*	zinc finger protein 469 [Source:HGNC Symbol;Acc:HGNC:23216]
**-1,016698351**	3,37E-88	3,33E-86	*MYL12A*	myosin light chain 12A [Source:HGNC Symbol;Acc:HGNC:16701]
**-1,016531282**	1,03E-13	1,17E-12	*PUS7*	pseudouridylate synthase 7 [Source:HGNC Symbol;Acc:HGNC:26033]
**-1,01630817**	1,69E-52	7,97E-51	*EXT1*	exostosin glycosyltransferase 1 [Source:HGNC Symbol;Acc:HGNC:3512]
**-1,014901912**	5,09E-16	6,59E-15	*RAB23*	RAB23, member RAS oncogene family [Source:HGNC Symbol;Acc:HGNC:14263]
**-1,013713906**	3,34E-10	2,87E-09	*CDCA3*	cell division cycle associated 3 [Source:HGNC Symbol;Acc:HGNC:14624]
**-1,012770798**	3,45E-85	3,19E-83	*CDK6*	cyclin dependent kinase 6 [Source:HGNC Symbol;Acc:HGNC:1777]

**-1,012667503**	1,15E-10	1,02E-09	*EGF*	epidermal growth factor [Source:HGNC Symbol;Acc:HGNC:3229]
**-1,012550213**	2,45E-20	4,01E-19	*RASSF1*	Ras association domain family member 1 [Source:HGNC Symbol;Acc:HGNC:9882]
**-1,011702491**	2,79E-07	1,81E-06	*COBLL1*	cordon-bleu WH2 repeat protein like 1 [Source:HGNC Symbol;Acc:HGNC:23571]
**-1,010954832**	2,13E-08	1,56E-07	*CD83*	CD83 molecule [Source:HGNC Symbol;Acc:HGNC:1703]
**-1,010473862**	6,27E-13	6,80E-12	*KLHL23*	kelch like family member 23 [Source:HGNC Symbol;Acc:HGNC:27506]
**-1,009518952**	1,55E-31	4,04E-30	*TACC3*	transforming acidic coiled-coil containing protein 3 [Source:HGNC Symbol;Acc:HGNC:11524]
**-1,008988383**	5,59E-28	1,26E-26	*ST3GAL1*	ST3 beta-galactoside alpha-2,3-sialyltransferase 1 [Source:HGNC Symbol;Acc:HGNC:10862]
**-1,008712101**	6,52E-05	0,000308341	*LDLRAD4*	low density lipoprotein receptor class A domain containing 4 [Source:HGNC Symbol;Acc:HGNC:1224]
**-1,007736611**	5,76E-05	0,000275234	*GDF6*	growth differentiation factor 6 [Source:HGNC Symbol;Acc:HGNC:4221]
**-1,007692902**	3,44E-27	7,51E-26	*SEMA7A*	semaphorin 7A (John Milton Hagen blood group) [Source:HGNC Symbol;Acc:HGNC:10741]
**-1,007473023**	2,15E-61	1,28E-59	*PSAT1*	phosphoserine aminotransferase 1 [Source:HGNC Symbol;Acc:HGNC:19129]
**-1,006843806**	2,73E-15	3,40E-14	*NCAPG*	non-SMC condensin I complex subunit G [Source:HGNC Symbol;Acc:HGNC:24304]
**-1,006576267**	4,30E-07	2,72E-06	*FAXC*	failed axon connections homolog [Source:HGNC Symbol;Acc:HGNC:20742]
**-1,00599093**	1,98E-34	5,68E-33	*MCM3*	minichromosome maintenance complex component 3 [Source:HGNC Symbol;Acc:HGNC:6945]
**-1,005332192**	7,83E-06	4,24E-05	*CCDC138*	coiled-coil domain containing 138 [Source:HGNC Symbol;Acc:HGNC:26531]
**-1,00492291**	0,003412765	0,011466087	*DNAH5*	dynein axonemal heavy chain 5 [Source:HGNC Symbol;Acc:HGNC:2950]
**-1,004578178**	2,66E-47	1,11E-45	*BCAT1*	branched chain amino acid transaminase 1 [Source:HGNC Symbol;Acc:HGNC:976]
**-1,003966719**	4,19E-07	2,65E-06	*DSCC1*	DNA replication and sister chromatid cohesion 1 [Source:HGNC Symbol;Acc:HGNC:24453]
**-1,002595876**	5,10E-49	2,23E-47	*CHST15*	carbohydrate sulfotransferase 15 [Source:HGNC Symbol;Acc:HGNC:18137]
**-0,999235344**	2,98E-15	3,71E-14	*EOGT*	EGF domain specific O-linked N-acetylglucosamine transferase [Source:HGNC Symbol;Acc:HGNC:28526]
**-0,998661126**	3,47E-35	1,02E-33	*DNAJB4*	DnaJ heat shock protein family (Hsp40) member B4 [Source:HGNC Symbol;Acc:HGNC:14886]
**-0,997880752**	7,10E-42	2,52E-40	*SGK1*	serum/glucocorticoid regulated kinase 1 [Source:HGNC Symbol;Acc:HGNC:10810]
**-0,996233305**	3,01E-09	2,38E-08	*VEGFC*	vascular endothelial growth factor C [Source:HGNC Symbol;Acc:HGNC:12682]
**-0,995578289**	1,02E-111	1,46E-109	*PLAU*	plasminogen activator, urokinase [Source:HGNC Symbol;Acc:HGNC:9052]
**-0,994522709**	8,39E-09	6,36E-08	*MARCH4*	membrane associated ring-CH-type finger 4 [Source:HGNC Symbol;Acc:HGNC:29269]
**-0,992578223**	2,25E-26	4,78E-25	*CCNB1*	cyclin B1 [Source:HGNC Symbol;Acc:HGNC:1579]
**-0,992313359**	0,000129687	0,0005823	*HRCT1*	histidine rich carboxyl terminus 1 [Source:HGNC Symbol;Acc:HGNC:33872]
**-0,99112847**	2,46E-33	6,79E-32	*AKIP1*	A-kinase interacting protein 1 [Source:HGNC Symbol;Acc:HGNC:1170]
**-0,990996259**	1,12E-09	9,15E-09	*GRB14*	growth factor receptor bound protein 14 [Source:HGNC Symbol;Acc:HGNC:4565]
**-0,990762695**	4,71E-08	3,33E-07	*SPC24*	SPC24, NDC80 kinetochore complex component [Source:HGNC Symbol;Acc:HGNC:26913]
**-0,99070319**	3,79E-94	4,09E-92	*FBLIM1*	filamin binding LIM protein 1 [Source:HGNC Symbol;Acc:HGNC:24686]
**-0,988389301**	3,95E-101	4,88E-99	*VCL*	vinculin [Source:HGNC Symbol;Acc:HGNC:12665]

**-0,988345356**	1,82E-30	4,57E-29	*NT5C2*	5'-nucleotidase, cytosolic II [Source:HGNC Symbol;Acc:HGNC:8022]
**-0,9866179**	2,46E-10	2,13E-09	*CENPE*	centromere protein E [Source:HGNC Symbol;Acc:HGNC:1856]
**-0,985622443**	1,51E-33	4,19E-32	*GNAO1*	G protein subunit alpha o1 [Source:HGNC Symbol;Acc:HGNC:4389]
**-0,985620409**	2,27E-17	3,22E-16	*CDCA4*	cell division cycle associated 4 [Source:HGNC Symbol;Acc:HGNC:14625]
**-0,984767888**	1,48E-07	9,86E-07	*BARD1*	BRCA1 associated RING domain 1 [Source:HGNC Symbol;Acc:HGNC:952]
**-0,984315314**	6,41E-42	2,28E-40	*NFE2L3*	nuclear factor, erythroid 2 like 3 [Source:HGNC Symbol;Acc:HGNC:7783]
**-0,984122937**	2,57E-15	3,22E-14	*SLC7A6*	solute carrier family 7 member 6 [Source:HGNC Symbol;Acc:HGNC:11064]
**-0,984041644**	2,06E-06	1,20E-05	*C8orf88*	chromosome 8 open reading frame 88 [Source:HGNC Symbol;Acc:HGNC:44672]
**-0,983589454**	6,48E-11	5,87E-10	*CDT1*	chromatin licensing and DNA replication factor 1 [Source:HGNC Symbol;Acc:HGNC:24576]
**-0,983141055**	0,00022329	0,00095402	*BLM*	Bloom syndrome RecQ like helicase [Source:HGNC Symbol;Acc:HGNC:1058]
**-0,983111296**	5,42E-63	3,35E-61	*SLC39A14*	solute carrier family 39 member 14 [Source:HGNC Symbol;Acc:HGNC:20858]
**-0,981595216**	2,06E-64	1,32E-62	*PLOD2*	procollagen-lysine,2-oxoglutarate 5-dioxygenase 2 [Source:HGNC Symbol;Acc:HGNC:9082]
**-0,981303328**	2,95E-56	1,54E-54	*DDX21*	DExD-box helicase 21 [Source:HGNC Symbol;Acc:HGNC:2744]
**-0,980462157**	2,68E-23	4,95E-22	*POLD1*	DNA polymerase delta 1, catalytic subunit [Source:HGNC Symbol;Acc:HGNC:9175]
**-0,978613094**	7,08E-45	2,75E-43	*GALNT5*	polypeptide N-acetylgalactosaminyltransferase 5 [Source:HGNC Symbol;Acc:HGNC:4127]
**-0,978548414**	0,00040954	0,00167246	*C4A*	complement C4A (Rodgers blood group) [Source:HGNC Symbol;Acc:HGNC:1323]
**-0,97828917**	1,99E-11	1,89E-10	*CIP2A*	cell proliferation regulating inhibitor of protein phosphatase 2A [Source:HGNC Symbol;Acc:HGNC:29302]
**-0,975848177**	2,12E-07	1,39E-06	*CDC7*	cell division cycle 7 [Source:HGNC Symbol;Acc:HGNC:1745]
**-0,97584281**	3,38E-27	7,39E-26	*RAB32*	RAB32, member RAS oncogene family [Source:HGNC Symbol;Acc:HGNC:9772]
**-0,975065688**	2,39E-06	1,38E-05	*CHODL*	chondrolectin [Source:HGNC Symbol;Acc:HGNC:17807]
**-0,974826821**	5,30E-06	2,93E-05	*PROSER2*	proline and serine rich 2 [Source:HGNC Symbol;Acc:HGNC:23728]
**-0,973359902**	1,14E-127	2,01E-125	*COTL1*	coactosin like F-actin binding protein 1 [Source:HGNC Symbol;Acc:HGNC:18304]
**-0,973267951**	2,70E-63	1,68E-61	*COL4A1*	collagen type IV alpha 1 chain [Source:HGNC Symbol;Acc:HGNC:2202]
**-0,97311518**	2,32E-32	6,20E-31	*LUZP1*	leucine zipper protein 1 [Source:HGNC Symbol;Acc:HGNC:14985]
**-0,973112298**	2,02E-39	6,72E-38	*EPDR1*	ependymin related 1 [Source:HGNC Symbol;Acc:HGNC:17572]
**-0,970348886**	5,00E-08	3,53E-07	*MMS22L*	MMS22 like, DNA repair protein [Source:HGNC Symbol;Acc:HGNC:21475]
**-0,970097577**	4,92E-09	3,82E-08	*SGO2*	shugoshin 2 [Source:HGNC Symbol;Acc:HGNC:30812]
**-0,968740329**	1,23E-13	1,39E-12	*E2F5*	E2F transcription factor 5 [Source:HGNC Symbol;Acc:HGNC:3119]
**-0,968700842**	5,26E-44	1,99E-42	*CACNG4*	calcium voltage-gated channel auxiliary subunit gamma 4 [Source:HGNC Symbol;Acc:HGNC:1408]
**-0,965887912**	5,02E-21	8,50E-20	*CD34*	CD34 molecule [Source:HGNC Symbol;Acc:HGNC:1662]
**-0,965596819**	0,008855421	0,026734335	*CARD11*	caspase recruitment domain family member 11 [Source:HGNC Symbol;Acc:HGNC:16393]
**-0,965481023**	2,33E-52	1,09E-50	*NF2*	neurofibromin 2 [Source:HGNC Symbol;Acc:HGNC:7773]

**-0,964115025**	2,96E-07	1,91E-06	*C9orf40*	chromosome 9 open reading frame 40 [Source:HGNC Symbol;Acc:HGNC:23433]
**-0,963961518**	2,32E-17	3,29E-16	*RRS1*	ribosome biogenesis regulator homolog [Source:HGNC Symbol;Acc:HGNC:17083]
**-0,963655275**	2,62E-46	1,07E-44	*SMURF2*	SMAD specific E3 ubiquitin protein ligase 2 [Source:HGNC Symbol;Acc:HGNC:16809]
**-0,963359504**	0,000783975	0,003042766	*PLEKHH2*	pleckstrin homology, MyTH4 and FERM domain containing H2 [Source:HGNC Symbol;Acc:HGNC:30506]
**-0,961253388**	0,003044851	0,010359111	*OIP5*	Opa interacting protein 5 [Source:HGNC Symbol;Acc:HGNC:20300]
**-0,960191873**	2,00E-69	1,37E-67	*SUN2*	Sad1 and UNC84 domain containing 2 [Source:HGNC Symbol;Acc:HGNC:14210]
**-0,958599335**	4,10E-16	5,34E-15	*ARNTL2*	aryl hydrocarbon receptor nuclear translocator like 2 [Source:HGNC Symbol;Acc:HGNC:18984]
**-0,958413448**	1,12E-08	8,43E-08	*C21orf58*	chromosome 21 open reading frame 58 [Source:HGNC Symbol;Acc:HGNC:1300]
**-0,956911111**	7,82E-17	1,07E-15	*CDK2*	cyclin dependent kinase 2 [Source:HGNC Symbol;Acc:HGNC:1771]
**-0,954599964**	2,30E-10	2,01E-09	*VCAN*	versican [Source:HGNC Symbol;Acc:HGNC:2464]
**-0,954510814**	7,90E-38	2,50E-36	*MCM4*	minichromosome maintenance complex component 4 [Source:HGNC Symbol;Acc:HGNC:6947]
**-0,952986945**	1,76E-86	1,68E-84	*VASP*	vasodilator stimulated phosphoprotein [Source:HGNC Symbol;Acc:HGNC:12652]
**-0,95288636**	3,23E-37	1,01E-35	*LMCD1*	LIM and cysteine rich domains 1 [Source:HGNC Symbol;Acc:HGNC:6633]
**-0,952849787**	4,54E-16	5,90E-15	*NDC1*	NDC1 transmembrane nucleoporin [Source:HGNC Symbol;Acc:HGNC:25525]
**-0,952772878**	6,47E-13	7,01E-12	*KNTC1*	kinetochore associated 1 [Source:HGNC Symbol;Acc:HGNC:17255]
**-0,95215529**	6,80E-32	1,79E-30	*GPX3*	glutathione peroxidase 3 [Source:HGNC Symbol;Acc:HGNC:4555]
**-0,951226043**	6,06E-71	4,29E-69	*NECTIN2*	nectin cell adhesion molecule 2 [Source:HGNC Symbol;Acc:HGNC:9707]
**-0,950698739**	3,49E-31	8,94E-30	*AMOT*	angiomotin [Source:HGNC Symbol;Acc:HGNC:17810]
**-0,950111072**	5,42E-09	4,18E-08	*CCDC107*	coiled-coil domain containing 107 [Source:HGNC Symbol;Acc:HGNC:28465]
**-0,948857171**	6,86E-37	2,14E-35	*KLF7*	Kruppel like factor 7 [Source:HGNC Symbol;Acc:HGNC:6350]
**-0,948617845**	1,42E-18	2,14E-17	*NANS*	N-acetylneuraminate synthase [Source:HGNC Symbol;Acc:HGNC:19237]
**-0,948296378**	7,19E-37	2,23E-35	*COL12A1*	collagen type XII alpha 1 chain [Source:HGNC Symbol;Acc:HGNC:2188]
**-0,947775375**	5,22E-21	8,83E-20	*TES*	testin LIM domain protein [Source:HGNC Symbol;Acc:HGNC:14620]
**-0,947668332**	1,48E-31	3,87E-30	*HSPA2*	heat shock protein family A (Hsp70) member 2 [Source:HGNC Symbol;Acc:HGNC:5235]
**-0,946279273**	2,48E-42	8,93E-41	*PYCR1*	pyrroline-5-carboxylate reductase 1 [Source:HGNC Symbol;Acc:HGNC:9721]
**-0,944902387**	6,46E-81	5,50E-79	*DAPK3*	death associated protein kinase 3 [Source:HGNC Symbol;Acc:HGNC:2676]
**-0,943546665**	2,39E-05	0,000120889	*DCDC2*	doublecortin domain containing 2 [Source:HGNC Symbol;Acc:HGNC:18141]
**-0,943498082**	0,000243787	0,001034534	*TRAIP*	TRAF interacting protein [Source:HGNC Symbol;Acc:HGNC:30764]
**-0,942767283**	0,009538802	0,028495996	*CCDC150*	coiled-coil domain containing 150 [Source:HGNC Symbol;Acc:HGNC:26834]
**-0,940085402**	0,003883479	0,012875927	*PLAC9*	placenta specific 9 [Source:HGNC Symbol;Acc:HGNC:19255]
**-0,939907297**	2,25E-07	1,48E-06	*CENPJ*	centromere protein J [Source:HGNC Symbol;Acc:HGNC:17272]
**-0,939471229**	1,16E-27	2,59E-26	*PUM3*	pumilio RNA binding family member 3 [Source:HGNC Symbol;Acc:HGNC:29676]

**-0,936946533**	2,39E-11	2,27E-10	*RASSF7*	Ras association domain family member 7 [Source:HGNC Symbol;Acc:HGNC:1166]
**-0,936294927**	5,37E-24	1,04E-22	*DNAJC1*	DnaJ heat shock protein family (Hsp40) member C1 [Source:HGNC Symbol;Acc:HGNC:20090]
**-0,935487687**	5,32E-10	4,48E-09	*NFKBIZ*	NFKB inhibitor zeta [Source:HGNC Symbol;Acc:HGNC:29805]
**-0,933582532**	8,83E-17	1,21E-15	*HIVEP1*	human immunodeficiency virus type I enhancer binding protein 1 [Source:HGNC Symbol;Acc:HGNC:4920]
**-0,931890456**	1,93E-09	1,54E-08	*BRI3BP*	BRI3 binding protein [Source:HGNC Symbol;Acc:HGNC:14251]
**-0,931562058**	2,26E-54	1,12E-52	*LRRC59*	leucine rich repeat containing 59 [Source:HGNC Symbol;Acc:HGNC:28817]
**-0,931454334**	0,000948315	0,003603486	*CTSV*	cathepsin V [Source:HGNC Symbol;Acc:HGNC:2538]
**-0,929536924**	3,47E-17	4,85E-16	*JMJD6*	jumonji domain containing 6, arginine demethylase and lysine hydroxylase [Source:HGNC Symbol;Acc:HGNC:19355]
**-0,927660477**	3,09E-09	2,44E-08	*MAOA*	monoamine oxidase A [Source:HGNC Symbol;Acc:HGNC:6833]
**-0,927087118**	4,47E-14	5,22E-13	*URB2*	URB2 ribosome biogenesis 2 homolog (S, cerevisiae) [Source:HGNC Symbol;Acc:HGNC:28967]
**-0,926500247**	3,56E-05	0,000175568	*PHETA2*	PH domain containing endocytic trafficking adaptor 2 [Source:HGNC Symbol;Acc:HGNC:27161]
**-0,924631749**	0,005900656	0,018738035	*KCNH1*	potassium voltage-gated channel subfamily H member 1 [Source:HGNC Symbol;Acc:HGNC:6250]
**-0,924497994**	1,34E-08	9,96E-08	*GGCT*	gamma-glutamylcyclotransferase [Source:HGNC Symbol;Acc:HGNC:21705]
**-0,923180963**	1,28E-58	7,00E-57	*FHL1*	four and a half LIM domains 1 [Source:HGNC Symbol;Acc:HGNC:3702]
**-0,923142358**	0,004647955	0,015128853	*SLC26A10*	solute carrier family 26 member 10 [Source:HGNC Symbol;Acc:HGNC:14470]
**-0,921887555**	9,09E-09	6,87E-08	*RAD18*	RAD18, E3 ubiquitin protein ligase [Source:HGNC Symbol;Acc:HGNC:18278]
**-0,920962052**	1,10E-32	2,99E-31	*ITGA11*	integrin subunit alpha 11 [Source:HGNC Symbol;Acc:HGNC:6136]
**-0,920758181**	3,17E-77	2,48E-75	*NACC2*	NACC family member 2 [Source:HGNC Symbol;Acc:HGNC:23846]
**-0,916425625**	7,90E-06	4,27E-05	*GLRB*	glycine receptor beta [Source:HGNC Symbol;Acc:HGNC:4329]
**-0,916410536**	1,08E-90	1,12E-88	*DSTN*	destrin, actin depolymerizing factor [Source:HGNC Symbol;Acc:HGNC:15750]
**-0,915255416**	4,70E-19	7,27E-18	*FEN1*	flap structure-specific endonuclease 1 [Source:HGNC Symbol;Acc:HGNC:3650]
**-0,914733554**	0,002873213	0,009832436	*AFAP1L1*	actin filament associated protein 1 like 1 [Source:HGNC Symbol;Acc:HGNC:26714]
**-0,913930583**	0,000711002	0,002778435	*CEP72*	centrosomal protein 72 [Source:HGNC Symbol;Acc:HGNC:25547]
**-0,91309678**	8,98E-14	1,03E-12	*ACOX3*	acyl-CoA oxidase 3, pristanoyl [Source:HGNC Symbol;Acc:HGNC:121]
**-0,912199842**	8,42E-22	1,47E-20	*NECTIN3*	nectin cell adhesion molecule 3 [Source:HGNC Symbol;Acc:HGNC:17664]
**-0,911364061**	0,000101136	0,000462999	*RPL39L*	ribosomal protein L39 like [Source:HGNC Symbol;Acc:HGNC:17094]
**-0,910830294**	7,79E-47	3,24E-45	*H2AFX*	H2A histone family member X [Source:HGNC Symbol;Acc:HGNC:4739]
**-0,910382879**	3,24E-09	2,56E-08	*RFC4*	replication factor C subunit 4 [Source:HGNC Symbol;Acc:HGNC:9972]
**-0,908978407**	2,10E-17	2,99E-16	*CHTF18*	chromosome transmission fidelity factor 18 [Source:HGNC Symbol;Acc:HGNC:18435]
**-0,908812674**	2,44E-38	7,89E-37	*BMP4*	bone morphogenetic protein 4 [Source:HGNC Symbol;Acc:HGNC:1071]
**-0,908714163**	0,004691856	0,015258949	*ADAMTSL5*	ADAMTS like 5 [Source:HGNC Symbol;Acc:HGNC:27912]

**-0,907983565**	2,74E-30	6,83E-29	*FIBCD1*	fibrinogen C domain containing 1 [Source:HGNC Symbol;Acc:HGNC:25922]
**-0,907074648**	4,71E-30	1,15E-28	*MBNL2*	muscleblind like splicing regulator 2 [Source:HGNC Symbol;Acc:HGNC:16746]
**-0,906404395**	1,20E-23	2,27E-22	*POLR1E*	RNA polymerase I subunit E [Source:HGNC Symbol;Acc:HGNC:17631]
**-0,905704697**	6,60E-10	5,52E-09	*CCNB2*	cyclin B2 [Source:HGNC Symbol;Acc:HGNC:1580]
**-0,904706812**	1,16E-16	1,57E-15	*HADH*	hydroxyacyl-CoA dehydrogenase [Source:HGNC Symbol;Acc:HGNC:4799]
**-0,904556862**	4,94E-14	5,73E-13	*BUB1*	BUB1 mitotic checkpoint serine/threonine kinase [Source:HGNC Symbol;Acc:HGNC:1148]
**-0,903660521**	3,60E-09	2,82E-08	*COL1A2*	collagen type I alpha 2 chain [Source:HGNC Symbol;Acc:HGNC:2198]
**-0,903240489**	4,06E-54	2,00E-52	*TNFAIP1*	TNF alpha induced protein 1 [Source:HGNC Symbol;Acc:HGNC:11894]
**-0,902275653**	3,66E-39	1,21E-37	*MYO1E*	myosin IE [Source:HGNC Symbol;Acc:HGNC:7599]
**-0,90225176**	9,67E-20	1,54E-18	*SHISA9*	shisa family member 9 [Source:HGNC Symbol;Acc:HGNC:37231]
**-0,901756664**	2,46E-25	4,99E-24	*CXCL14*	C-X-C motif chemokine ligand 14 [Source:HGNC Symbol;Acc:HGNC:10640]
**-0,901236427**	2,38E-43	8,87E-42	*DEGS1*	delta 4-desaturase, sphingolipid 1 [Source:HGNC Symbol;Acc:HGNC:13709]
**-0,901045828**	0,00010713	0,000487564	*USP43*	ubiquitin specific peptidase 43 [Source:HGNC Symbol;Acc:HGNC:20072]
**-0,900825965**	1,29E-44	4,97E-43	*SNX18*	sorting nexin 18 [Source:HGNC Symbol;Acc:HGNC:19245]
**-0,900568896**	4,93E-05	0,000237701	*ARFGEF3*	ARFGEF family member 3 [Source:HGNC Symbol;Acc:HGNC:21213]
**-0,900531857**	1,45E-53	6,98E-52	*B4GALT1*	beta-1,4-galactosyltransferase 1 [Source:HGNC Symbol;Acc:HGNC:924]
**-0,900493178**	4,90E-42	1,75E-40	*ADAMTS10*	ADAM metallopeptidase with thrombospondin type 1 motif 10 [Source:HGNC Symbol;Acc:HGNC:13201]
**-0,900224513**	2,31E-10	2,01E-09	*GPR39*	G protein-coupled receptor 39 [Source:HGNC Symbol;Acc:HGNC:4496]
**-0,89885575**	0,010682111	0,031446762	*PHKG1*	phosphorylase kinase catalytic subunit gamma 1 [Source:HGNC Symbol;Acc:HGNC:8930]
**-0,89857884**	3,91E-08	2,78E-07	*GFOD1*	glucose-fructose oxidoreductase domain containing 1 [Source:HGNC Symbol;Acc:HGNC:21096]
**-0,898561044**	6,56E-40	2,20E-38	*COL5A2*	collagen type V alpha 2 chain [Source:HGNC Symbol;Acc:HGNC:2210]
**-0,897675217**	3,16E-77	2,48E-75	*NDST1*	N-deacetylase and N-sulfotransferase 1 [Source:HGNC Symbol;Acc:HGNC:7680]
**-0,897404472**	6,80E-63	4,18E-61	*FHL2*	four and a half LIM domains 2 [Source:HGNC Symbol;Acc:HGNC:3703]
**-0,896892924**	9,58E-11	8,55E-10	*TCF7L1*	transcription factor 7 like 1 [Source:HGNC Symbol;Acc:HGNC:11640]
**-0,896731145**	0,001546173	0,005615765	*CTNNA2*	catenin alpha 2 [Source:HGNC Symbol;Acc:HGNC:2510]
**-0,896646025**	1,05E-37	3,33E-36	*MYC*	MYC proto-oncogene, bHLH transcription factor [Source:HGNC Symbol;Acc:HGNC:7553]
**-0,896244249**	2,17E-125	3,66E-123	*ACTG1*	actin gamma 1 [Source:HGNC Symbol;Acc:HGNC:144]
**-0,895398781**	1,16E-16	1,57E-15	*MAP3K14*	mitogen-activated protein kinase kinase kinase 14 [Source:HGNC Symbol;Acc:HGNC:6853]
**-0,894728728**	6,21E-06	3,40E-05	*SPATA5*	spermatogenesis associated 5 [Source:HGNC Symbol;Acc:HGNC:18119]
**-0,894204392**	4,47E-06	2,49E-05	*CCNE1*	cyclin E1 [Source:HGNC Symbol;Acc:HGNC:1589]
**-0,893599816**	6,44E-07	4,00E-06	*OLFM1*	olfactomedin 1 [Source:HGNC Symbol;Acc:HGNC:17187]
**-0,893103276**	3,09E-15	3,85E-14	*ADAMTS2*	ADAM metallopeptidase with thrombospondin type 1 motif 2 [Source:HGNC Symbol;Acc:HGNC:218]
**-0,892619936**	6,73E-66	4,41E-64	*COL4A2*	collagen type IV alpha 2 chain [Source:HGNC Symbol;Acc:HGNC:2203]
**-0,891918647**	6,11E-13	6,64E-12	*CFI*	complement factor I [Source:HGNC Symbol;Acc:HGNC:5394]

**-0,891486277**	2,29E-76	1,75E-74	*CAP1*	cyclase associated actin cytoskeleton regulatory protein 1 [Source:HGNC Symbol;Acc:HGNC:20040]
**-0,890158762**	1,46E-63	9,16E-62	*JPT1*	Jupiter microtubule associated homolog 1 [Source:HGNC Symbol;Acc:HGNC:14569]
**-0,890009234**	6,25E-06	3,42E-05	*HAUS8*	HAUS augmin like complex subunit 8 [Source:HGNC Symbol;Acc:HGNC:30532]
**-0,889642729**	1,81E-92	1,92E-90	*GNG12*	G protein subunit gamma 12 [Source:HGNC Symbol;Acc:HGNC:19663]
**-0,889064885**	5,78E-07	3,61E-06	*RBM24*	RNA binding motif protein 24 [Source:HGNC Symbol;Acc:HGNC:21539]
**-0,888108522**	8,60E-20	1,37E-18	*AMIGO2*	adhesion molecule with Ig like domain 2 [Source:HGNC Symbol;Acc:HGNC:24073]
**-0,887957206**	3,84E-05	0,000188594	*GAS2L3*	growth arrest specific 2 like 3 [Source:HGNC Symbol;Acc:HGNC:27475]
**-0,886180112**	3,57E-17	4,98E-16	*FAM107B*	family with sequence similarity 107 member B [Source:HGNC Symbol;Acc:HGNC:23726]
**-0,886179948**	1,45E-05	7,53E-05	*PDSS1*	decaprenyl diphosphate synthase subunit 1 [Source:HGNC Symbol;Acc:HGNC:17759]
**-0,885851578**	1,11E-23	2,09E-22	*ALDH4A1*	aldehyde dehydrogenase 4 family member A1 [Source:HGNC Symbol;Acc:HGNC:406]
**-0,885849905**	1,04E-16	1,41E-15	*BICC1*	BicC family RNA binding protein 1 [Source:HGNC Symbol;Acc:HGNC:19351]
**-0,883046497**	2,85E-15	3,55E-14	*FOXM1*	forkhead box M1 [Source:HGNC Symbol;Acc:HGNC:3818]
**-0,882701658**	1,39E-80	1,18E-78	*CDV3*	CDV3 homolog [Source:HGNC Symbol;Acc:HGNC:26928]
**-0,88223512**	4,93E-07	3,10E-06	*BRCA1*	BRCA1, DNA repair associated [Source:HGNC Symbol;Acc:HGNC:1100]
**-0,880450971**	2,47E-19	3,87E-18	*RACGAP1*	Rac GTPase activating protein 1 [Source:HGNC Symbol;Acc:HGNC:9804]
**-0,880064614**	9,25E-14	1,05E-12	*PARD3B*	par-3 family cell polarity regulator beta [Source:HGNC Symbol;Acc:HGNC:14446]
**-0,879885614**	5,18E-46	2,10E-44	*LMNB2*	lamin B2 [Source:HGNC Symbol;Acc:HGNC:6638]
**-0,879845988**	0,004311431	0,014147383	*FSCN2*	fascin actin-bundling protein 2, retinal [Source:HGNC Symbol;Acc:HGNC:3960]
**-0,879086046**	0,000803987	0,003110482	*SCLT1*	sodium channel and clathrin linker 1 [Source:HGNC Symbol;Acc:HGNC:26406]
**-0,878814673**	1,78E-25	3,64E-24	*ARHGAP31*	Rho GTPase activating protein 31 [Source:HGNC Symbol;Acc:HGNC:29216]
**-0,878555215**	5,15E-11	4,72E-10	*DPYSL5*	dihydropyrimidinase like 5 [Source:HGNC Symbol;Acc:HGNC:20637]
**-0,877260875**	9,89E-07	6,03E-06	*FRZB*	frizzled related protein [Source:HGNC Symbol;Acc:HGNC:3959]
**-0,876560888**	4,35E-20	7,02E-19	*IFRD2*	interferon related developmental regulator 2 [Source:HGNC Symbol;Acc:HGNC:5457]
**-0,876120895**	3,31E-66	2,18E-64	*EDIL3*	EGF like repeats and discoidin domains 3 [Source:HGNC Symbol;Acc:HGNC:3173]
**-0,875783882**	3,52E-28	7,98E-27	*COL9A2*	collagen type IX alpha 2 chain [Source:HGNC Symbol;Acc:HGNC:2218]
**-0,874454864**	1,18E-13	1,34E-12	*ZWILCH*	zwilch kinetochore protein [Source:HGNC Symbol;Acc:HGNC:25468]
**-0,87323661**	4,13E-09	3,23E-08	*NEGR1*	neuronal growth regulator 1 [Source:HGNC Symbol;Acc:HGNC:17302]
**-0,872909772**	2,21E-46	9,03E-45	*MYH10*	myosin heavy chain 10 [Source:HGNC Symbol;Acc:HGNC:7568]
**-0,872128726**	1,23E-28	2,86E-27	*CASKIN2*	CASK interacting protein 2 [Source:HGNC Symbol;Acc:HGNC:18200]
**-0,871944366**	3,06E-30	7,58E-29	*PLXND1*	plexin D1 [Source:HGNC Symbol;Acc:HGNC:9107]
**-0,870326093**	0,004195982	0,01378954	*COL25A1*	collagen type XXV alpha 1 chain [Source:HGNC Symbol;Acc:HGNC:18603]
**-0,869296791**	3,26E-25	6,60E-24	*RAB11FIP1*	RAB11 family interacting protein 1 [Source:HGNC Symbol;Acc:HGNC:30265]

**-0,868878493**	3,58E-36	1,08E-34	*MTHFD1L*	methylenetetrahydrofolate dehydrogenase (NADP+ dependent) 1 like [Source:HGNC Symbol;Acc:HGNC:21055]
**-0,867391676**	1,27E-24	2,50E-23	*KCTD15*	potassium channel tetramerization domain containing 15 [Source:HGNC Symbol;Acc:HGNC:23297]
**-0,866609238**	5,58E-76	4,25E-74	*ZC3H7B*	zinc finger CCCH-type containing 7B [Source:HGNC Symbol;Acc:HGNC:30869]
**-0,866376055**	1,87E-11	1,79E-10	*ARHGEF26*	Rho guanine nucleotide exchange factor 26 [Source:HGNC Symbol;Acc:HGNC:24490]
**-0,86630638**	2,17E-16	2,88E-15	*CASC10*	cancer susceptibility 10 [Source:HGNC Symbol;Acc:HGNC:31448]
**-0,865891388**	1,64E-18	2,46E-17	*MAFK*	MAF bZIP transcription factor K [Source:HGNC Symbol;Acc:HGNC:6782]
**-0,865776853**	6,00E-08	4,19E-07	*LRRC3*	leucine rich repeat containing 3 [Source:HGNC Symbol;Acc:HGNC:14965]
**-0,86525522**	6,13E-08	4,28E-07	*ZC3H12A*	zinc finger CCCH-type containing 12A [Source:HGNC Symbol;Acc:HGNC:26259]
**-0,865148898**	6,11E-08	4,27E-07	*NPY2R*	neuropeptide Y receptor Y2 [Source:HGNC Symbol;Acc:HGNC:7957]
**-0,861929343**	6,66E-40	2,24E-38	*CHPT1*	choline phosphotransferase 1 [Source:HGNC Symbol;Acc:HGNC:17852]
**-0,860821149**	5,81E-23	1,06E-21	*DIXDC1*	DIX domain containing 1 [Source:HGNC Symbol;Acc:HGNC:23695]
**-0,860429933**	3,01E-55	1,52E-53	*ARHGAP23*	Rho GTPase activating protein 23 [Source:HGNC Symbol;Acc:HGNC:29293]
**-0,860252789**	9,24E-13	9,88E-12	*RECK*	reversion inducing cysteine rich protein with kazal motifs [Source:HGNC Symbol;Acc:HGNC:11345]
**-0,858728146**	6,04E-17	8,33E-16	*SAP30*	Sin3A associated protein 30 [Source:HGNC Symbol;Acc:HGNC:10532]
**-0,85855696**	1,16E-17	1,67E-16	*GPATCH4*	G-patch domain containing 4 [Source:HGNC Symbol;Acc:HGNC:25982]
**-0,857827058**	1,33E-12	1,40E-11	*PLEKHG3*	pleckstrin homology and RhoGEF domain containing G3 [Source:HGNC Symbol;Acc:HGNC:20364]
**-0,857786408**	0,00983832	0,029278092	*GPR27*	G protein-coupled receptor 27 [Source:HGNC Symbol;Acc:HGNC:4482]
**-0,857692708**	2,96E-34	8,45E-33	*TEAD3*	TEA domain transcription factor 3 [Source:HGNC Symbol;Acc:HGNC:11716]
**-0,85717848**	1,27E-46	5,22E-45	*ZFP36L2*	ZFP36 ring finger protein like 2 [Source:HGNC Symbol;Acc:HGNC:1108]
**-0,85695964**	0,000665123	0,00261582	*ATAD5*	ATPase family, AAA domain containing 5 [Source:HGNC Symbol;Acc:HGNC:25752]
**-0,855409555**	7,33E-14	8,42E-13	*NMT2*	N-myristoyltransferase 2 [Source:HGNC Symbol;Acc:HGNC:7858]
**-0,855290496**	1,75E-26	3,73E-25	*STK38L*	serine/threonine kinase 38 like [Source:HGNC Symbol;Acc:HGNC:17848]
**-0,85305609**	3,35E-25	6,77E-24	*SRSF7*	serine and arginine rich splicing factor 7 [Source:HGNC Symbol;Acc:HGNC:10789]
**-0,852775239**	0,017869672	0,049111862	*C8orf48*	chromosome 8 open reading frame 48 [Source:HGNC Symbol;Acc:HGNC:26345]
**-0,852714305**	4,03E-18	5,94E-17	*LARGE1*	LARGE xylosyl- and glucuronyltransferase 1 [Source:HGNC Symbol;Acc:HGNC:6511]
**-0,851726232**	1,21E-64	7,75E-63	*SMAD3*	SMAD family member 3 [Source:HGNC Symbol;Acc:HGNC:6769]
**-0,851603708**	7,88E-18	1,15E-16	*L3HYPDH*	trans-L-3-hydroxyproline dehydratase [Source:HGNC Symbol;Acc:HGNC:20488]
**-0,851031801**	6,51E-39	2,12E-37	*LPP*	LIM domain containing preferred translocation partner in lipoma [Source:HGNC Symbol;Acc:HGNC:6679]
**-0,849056606**	0,000382202	0,001569073	*PARPBP*	PARP1 binding protein [Source:HGNC Symbol;Acc:HGNC:26074]
**-0,848494466**	2,82E-08	2,03E-07	*AADAT*	aminoadipate aminotransferase [Source:HGNC Symbol;Acc:HGNC:17929]

**-0,848478918**	7,44E-06	4,04E-05	*CA13*	carbonic anhydrase 13 [Source:HGNC Symbol;Acc:HGNC:14914]
**-0,848306649**	7,13E-07	4,41E-06	*NAV3*	neuron navigator 3 [Source:HGNC Symbol;Acc:HGNC:15998]
**-0,848171998**	8,63E-09	6,53E-08	*PKN3*	protein kinase N3 [Source:HGNC Symbol;Acc:HGNC:17999]
**-0,847900524**	4,22E-11	3,91E-10	*PNO1*	partner of NOB1 homolog [Source:HGNC Symbol;Acc:HGNC:32790]
**-0,846693893**	3,44E-11	3,21E-10	*CKS2*	CDC28 protein kinase regulatory subunit 2 [Source:HGNC Symbol;Acc:HGNC:2000]
**-0,844936085**	2,37E-52	1,11E-50	*SDC2*	syndecan 2 [Source:HGNC Symbol;Acc:HGNC:10659]
**-0,843560044**	1,13E-18	1,71E-17	*SHB*	SH2 domain containing adaptor protein B [Source:HGNC Symbol;Acc:HGNC:10838]
**-0,842950976**	1,16E-63	7,30E-62	*CNN3*	calponin 3 [Source:HGNC Symbol;Acc:HGNC:2157]
**-0,842547537**	2,93E-30	7,28E-29	*HMGB2*	high mobility group box 2 [Source:HGNC Symbol;Acc:HGNC:5000]
**-0,842248579**	1,54E-23	2,87E-22	*TLN2*	talin 2 [Source:HGNC Symbol;Acc:HGNC:15447]
**-0,841492758**	0,014536499	0,041067886	*FAM160A1*	family with sequence similarity 160 member A1 [Source:HGNC Symbol;Acc:HGNC:34237]
**-0,837160693**	7,46E-05	0,000348975	*SLFN12*	schlafen family member 12 [Source:HGNC Symbol;Acc:HGNC:25500]
**-0,836994756**	0,00654863	0,020550245	*RPS6KL1*	ribosomal protein S6 kinase like 1 [Source:HGNC Symbol;Acc:HGNC:20222]
**-0,836811819**	9,78E-10	8,04E-09	*PRR16*	proline rich 16 [Source:HGNC Symbol;Acc:HGNC:29654]
**-0,836606271**	5,81E-06	3,20E-05	*DBF4B*	DBF4 zinc finger B [Source:HGNC Symbol;Acc:HGNC:17883]
**-0,835713711**	2,67E-05	0,000134237	*C22orf23*	chromosome 22 open reading frame 23 [Source:HGNC Symbol;Acc:HGNC:18589]
**-0,83563303**	0,001370007	0,005035293	*FBXO36*	F-box protein 36 [Source:HGNC Symbol;Acc:HGNC:27020]
**-0,835549228**	0,00025341	0,001072321	*NALCN*	sodium leak channel, non-selective [Source:HGNC Symbol;Acc:HGNC:19082]
**-0,835206563**	5,36E-28	1,21E-26	*CTNNAL1*	catenin alpha like 1 [Source:HGNC Symbol;Acc:HGNC:2512]
**-0,834916199**	5,16E-05	0,000248342	*ELFN2*	extracellular leucine rich repeat and fibronectin type III domain containing 2 [Source:HGNC Symbol;Acc:HGNC:29396]
**-0,83366886**	1,56E-57	8,34E-56	*CCND1*	cyclin D1 [Source:HGNC Symbol;Acc:HGNC:1582]
**-0,832899213**	7,60E-08	5,25E-07	*MAPK4*	mitogen-activated protein kinase 4 [Source:HGNC Symbol;Acc:HGNC:6878]
**-0,832026832**	3,50E-83	3,13E-81	*WDR1*	WD repeat domain 1 [Source:HGNC Symbol;Acc:HGNC:12754]
**-0,831939877**	6,52E-46	2,63E-44	*PLCB3*	phospholipase C beta 3 [Source:HGNC Symbol;Acc:HGNC:9056]
**-0,83106459**	5,96E-06	3,28E-05	*CLTCL1*	clathrin heavy chain like 1 [Source:HGNC Symbol;Acc:HGNC:2093]
**-0,830574146**	9,79E-05	0,000449279	*DOK3*	docking protein 3 [Source:HGNC Symbol;Acc:HGNC:24583]
**-0,830043805**	1,38E-47	5,80E-46	*FUS*	FUS RNA binding protein [Source:HGNC Symbol;Acc:HGNC:4010]
**-0,829827395**	5,43E-07	3,41E-06	*FBN2*	fibrillin 2 [Source:HGNC Symbol;Acc:HGNC:3604]
**-0,829797519**	2,25E-05	0,000114101	*RBL1*	RB transcriptional corepressor like 1 [Source:HGNC Symbol;Acc:HGNC:9893]
**-0,829287721**	5,96E-43	2,21E-41	*CARD10*	caspase recruitment domain family member 10 [Source:HGNC Symbol;Acc:HGNC:16422]
**-0,829107006**	7,41E-35	2,15E-33	*SRM*	spermidine synthase [Source:HGNC Symbol;Acc:HGNC:11296]
**-0,827256082**	6,65E-08	4,63E-07	*ABHD17C*	abhydrolase domain containing 17C [Source:HGNC Symbol;Acc:HGNC:26925]
**-0,827244005**	9,09E-10	7,50E-09	*NOP16*	NOP16 nucleolar protein [Source:HGNC Symbol;Acc:HGNC:26934]

**-0,826856627**	3,54E-17	4,94E-16	*UTP20*	UTP20, small subunit processome component [Source:HGNC Symbol;Acc:HGNC:17897]
**-0,826526986**	5,02E-48	2,14E-46	*COL8A1*	collagen type VIII alpha 1 chain [Source:HGNC Symbol;Acc:HGNC:2215]
**-0,826384596**	9,81E-17	1,33E-15	*TIMELESS*	timeless circadian regulator [Source:HGNC Symbol;Acc:HGNC:11813]
**-0,825944443**	0,000520306	0,002079725	*PASK*	PAS domain containing serine/threonine kinase [Source:HGNC Symbol;Acc:HGNC:17270]
**-0,825575514**	0,003542893	0,011862212	*FAM53A*	family with sequence similarity 53 member A [Source:HGNC Symbol;Acc:HGNC:31860]
**-0,824901071**	8,36E-32	2,19E-30	*RNF41*	ring finger protein 41 [Source:HGNC Symbol;Acc:HGNC:18401]
**-0,824553869**	8,30E-83	7,39E-81	*ACTN4*	actinin alpha 4 [Source:HGNC Symbol;Acc:HGNC:166]
**-0,824382558**	7,95E-08	5,48E-07	*TSPAN15*	tetraspanin 15 [Source:HGNC Symbol;Acc:HGNC:23298]
**-0,823546062**	5,81E-12	5,76E-11	*CEP85*	centrosomal protein 85 [Source:HGNC Symbol;Acc:HGNC:25309]
**-0,823043795**	6,01E-24	1,16E-22	*CHN1*	chimerin 1 [Source:HGNC Symbol;Acc:HGNC:1943]
**-0,822692519**	0,000430059	0,001748608	*CHI3L2*	chitinase 3 like 2 [Source:HGNC Symbol;Acc:HGNC:1933]
**-0,822371804**	1,29E-12	1,37E-11	*BMP6*	bone morphogenetic protein 6 [Source:HGNC Symbol;Acc:HGNC:1073]
**-0,821800184**	6,13E-49	2,67E-47	*OSMR*	oncostatin M receptor [Source:HGNC Symbol;Acc:HGNC:8507]
**-0,821403244**	2,31E-08	1,68E-07	*ALPL*	alkaline phosphatase, liver/bone/kidney [Source:HGNC Symbol;Acc:HGNC:438]
**-0,821359709**	1,03E-47	4,36E-46	*SLC7A1*	solute carrier family 7 member 1 [Source:HGNC Symbol;Acc:HGNC:11057]
**-0,820193559**	0,011606971	0,033876506	*N4BP3*	NEDD4 binding protein 3 [Source:HGNC Symbol;Acc:HGNC:29852]
**-0,820035047**	1,32E-06	7,94E-06	*SLC43A3*	solute carrier family 43 member 3 [Source:HGNC Symbol;Acc:HGNC:17466]
**-0,818429278**	3,67E-09	2,88E-08	*JDP2*	Jun dimerization protein 2 [Source:HGNC Symbol;Acc:HGNC:17546]
**-0,817883972**	0,005686534	0,018093603	*PODNL1*	podocan like 1 [Source:HGNC Symbol;Acc:HGNC:26275]
**-0,817416925**	8,64E-06	4,65E-05	*NUP35*	nucleoporin 35 [Source:HGNC Symbol;Acc:HGNC:29797]
**-0,817266006**	1,46E-17	2,09E-16	*DCTPP1*	dCTP pyrophosphatase 1 [Source:HGNC Symbol;Acc:HGNC:28777]
**-0,816365841**	1,84E-25	3,76E-24	*SHROOM3*	shroom family member 3 [Source:HGNC Symbol;Acc:HGNC:30422]
**-0,816035112**	1,03E-77	8,26E-76	*CALU*	calumenin [Source:HGNC Symbol;Acc:HGNC:1458]
**-0,81478555**	2,15E-79	1,78E-77	*TUBB*	tubulin beta class I [Source:HGNC Symbol;Acc:HGNC:20778]
**-0,814576763**	9,95E-10	8,17E-09	*PID1*	phosphotyrosine interaction domain containing 1 [Source:HGNC Symbol;Acc:HGNC:26084]
**-0,813286294**	7,41E-20	1,18E-18	*BYSL*	bystin like [Source:HGNC Symbol;Acc:HGNC:1157]
**-0,812664023**	0,015449356	0,043318913	*ASNS*	asparagine synthetase (glutamine-hydrolyzing) [Source:HGNC Symbol;Acc:HGNC:753]
**-0,811485642**	3,49E-27	7,61E-26	*NFKBIA*	NFKB inhibitor alpha [Source:HGNC Symbol;Acc:HGNC:7797]
**-0,811457132**	1,15E-53	5,55E-52	*MYO1C*	myosin IC [Source:HGNC Symbol;Acc:HGNC:7597]
**-0,810775216**	2,38E-43	8,87E-42	*SEMA3C*	semaphorin 3C [Source:HGNC Symbol;Acc:HGNC:10725]
**-0,809793262**	4,74E-19	7,32E-18	*DYRK2*	dual specificity tyrosine phosphorylation regulated kinase 2 [Source:HGNC Symbol;Acc:HGNC:3093]
**-0,808878735**	2,18E-25	4,46E-24	*XBP1*	X-box binding protein 1 [Source:HGNC Symbol;Acc:HGNC:12801]
**-0,807767476**	6,44E-45	2,51E-43	*C1orf198*	chromosome 1 open reading frame 198 [Source:HGNC Symbol;Acc:HGNC:25900]
**-0,807494688**	3,41E-13	3,76E-12	*PYCR3*	pyrroline-5-carboxylate reductase 3 [Source:HGNC Symbol;Acc:HGNC:25846]
**-0,807234961**	4,39E-24	8,50E-23	*RCN3*	reticulocalbin 3 [Source:HGNC Symbol;Acc:HGNC:21145]
**-0,805928106**	1,40E-05	7,28E-05	*EIF5A2*	eukaryotic translation initiation factor 5A2 [Source:HGNC Symbol;Acc:HGNC:3301]

**-0,804695108**	1,79E-68	1,20E-66	*TUBB4B*	tubulin beta 4B class IVb [Source:HGNC Symbol;Acc:HGNC:20771]
**-0,803939162**	2,27E-10	1,98E-09	*SNX24*	sorting nexin 24 [Source:HGNC Symbol;Acc:HGNC:21533]
**-0,803593952**	1,73E-20	2,85E-19	*SMTN*	smoothelin [Source:HGNC Symbol;Acc:HGNC:11126]
**-0,803065287**	1,49E-10	1,32E-09	*GRAMD2B*	GRAM domain containing 2B [Source:HGNC Symbol;Acc:HGNC:24911]
**-0,802961536**	2,55E-15	3,19E-14	*S1PR3*	sphingosine-1-phosphate receptor 3 [Source:HGNC Symbol;Acc:HGNC:3167]
**-0,801475674**	6,08E-20	9,78E-19	*DUSP14*	dual specificity phosphatase 14 [Source:HGNC Symbol;Acc:HGNC:17007]
**-0,80090676**	1,85E-09	1,49E-08	*AFF3*	AF4/FMR2 family member 3 [Source:HGNC Symbol;Acc:HGNC:6473]
**-0,800659559**	4,51E-05	0,000218939	*SPATA33*	spermatogenesis associated 33 [Source:HGNC Symbol;Acc:HGNC:26463]
**-0,800460185**	0,0132382	0,037924492	*ZNF684*	zinc finger protein 684 [Source:HGNC Symbol;Acc:HGNC:28418]
**-0,800390056**	0,008165809	0,02488078	*EPSTI1*	epithelial stromal interaction 1 [Source:HGNC Symbol;Acc:HGNC:16465]
**-0,800120976**	4,97E-09	3,85E-08	*PRMT9*	protein arginine methyltransferase 9 [Source:HGNC Symbol;Acc:HGNC:25099]
**-0,799908212**	5,45E-17	7,53E-16	*INTS13*	integrator complex subunit 13 [Source:HGNC Symbol;Acc:HGNC:20174]
**-0,799218483**	1,09E-41	3,85E-40	*CYCS*	cytochrome c, somatic [Source:HGNC Symbol;Acc:HGNC:19986]
**-0,798647824**	6,53E-06	3,57E-05	*CXCL5*	C-X-C motif chemokine ligand 5 [Source:HGNC Symbol;Acc:HGNC:10642]
**-0,798640884**	6,38E-18	9,32E-17	*CEBPG*	CCAAT enhancer binding protein gamma [Source:HGNC Symbol;Acc:HGNC:1837]
**-0,798018833**	0,01060171	0,03125274	*GRPR*	gastrin releasing peptide receptor [Source:HGNC Symbol;Acc:HGNC:4609]
**-0,797575918**	3,07E-10	2,64E-09	*DUSP8*	dual specificity phosphatase 8 [Source:HGNC Symbol;Acc:HGNC:3074]
**-0,797543742**	0,000257328	0,001086768	*SGCD*	sarcoglycan delta [Source:HGNC Symbol;Acc:HGNC:10807]
**-0,796876019**	1,30E-05	6,78E-05	*FBXO5*	F-box protein 5 [Source:HGNC Symbol;Acc:HGNC:13584]
**-0,796714969**	4,13E-09	3,22E-08	*TMEM131L*	transmembrane 131 like [Source:HGNC Symbol;Acc:HGNC:29146]
**-0,79592218**	2,44E-06	1,41E-05	*CORO1A*	coronin 1A [Source:HGNC Symbol;Acc:HGNC:2252]
**-0,795227049**	9,50E-41	3,27E-39	*PDPN*	podoplanin [Source:HGNC Symbol;Acc:HGNC:29602]
**-0,795212401**	4,12E-15	5,07E-14	*TET3*	tet methylcytosine dioxygenase 3 [Source:HGNC Symbol;Acc:HGNC:28313]
**-0,795181055**	1,68E-06	9,91E-06	*PLSCR4*	phospholipid scramblase 4 [Source:HGNC Symbol;Acc:HGNC:16497]
**-0,793962315**	1,38E-09	1,12E-08	*DGKH*	diacylglycerol kinase eta [Source:HGNC Symbol;Acc:HGNC:2854]
**-0,793320044**	4,29E-09	3,34E-08	*TANGO6*	transport and golgi organization 6 homolog [Source:HGNC Symbol;Acc:HGNC:25749]
**-0,793115814**	2,17E-05	0,00011023	*SMAD9*	SMAD family member 9 [Source:HGNC Symbol;Acc:HGNC:6774]
**-0,792810473**	0,000436513	0,001771972	*LRRK1*	leucine rich repeat kinase 1 [Source:HGNC Symbol;Acc:HGNC:18608]
**-0,792372408**	2,94E-59	1,65E-57	*TUBA1B*	tubulin alpha 1b [Source:HGNC Symbol;Acc:HGNC:18809]
**-0,791457503**	2,62E-10	2,27E-09	*RNF217*	ring finger protein 217 [Source:HGNC Symbol;Acc:HGNC:21487]
**-0,790770072**	1,07E-21	1,86E-20	*CYP1B1*	cytochrome P450 family 1 subfamily B member 1 [Source:HGNC Symbol;Acc:HGNC:2597]
**-0,790115645**	0,008098322	0,024694538	*PGM5*	phosphoglucomutase 5 [Source:HGNC Symbol;Acc:HGNC:8908]
**-0,788658394**	2,14E-24	4,21E-23	*ELL2*	elongation factor for RNA polymerase II 2 [Source:HGNC Symbol;Acc:HGNC:17064]

**-0,787176528**	4,73E-26	9,95E-25	*WWC3*	WWC family member 3 [Source:HGNC Symbol;Acc:HGNC:29237]
**-0,786407509**	0,001468963	0,005365461	*ZNF804A*	zinc finger protein 804A [Source:HGNC Symbol;Acc:HGNC:21711]
**-0,785409474**	1,49E-16	1,99E-15	*SLC1A4*	solute carrier family 1 member 4 [Source:HGNC Symbol;Acc:HGNC:10942]
**-0,785062018**	8,90E-17	1,21E-15	*BAMBI*	BMP and activin membrane bound inhibitor [Source:HGNC Symbol;Acc:HGNC:30251]
**-0,783918641**	5,97E-15	7,27E-14	*INCENP*	inner centromere protein [Source:HGNC Symbol;Acc:HGNC:6058]
**-0,783414682**	4,74E-49	2,09E-47	*HNRNPAB*	heterogeneous nuclear ribonucleoprotein A/B [Source:HGNC Symbol;Acc:HGNC:5034]
**-0,783155328**	3,36E-56	1,74E-54	*ENAH*	ENAH, actin regulator [Source:HGNC Symbol;Acc:HGNC:18271]
**-0,78279168**	5,18E-18	7,60E-17	*FZD8*	frizzled class receptor 8 [Source:HGNC Symbol;Acc:HGNC:4046]
**-0,782072603**	5,44E-07	3,41E-06	*PEX3*	peroxisomal biogenesis factor 3 [Source:HGNC Symbol;Acc:HGNC:8858]
**-0,779963325**	1,26E-29	3,04E-28	*GPR176*	G protein-coupled receptor 176 [Source:HGNC Symbol;Acc:HGNC:32370]
**-0,778229974**	5,52E-13	6,03E-12	*TMEM160*	transmembrane protein 160 [Source:HGNC Symbol;Acc:HGNC:26042]
**-0,778162048**	0,000218426	0,000935093	*MPP3*	membrane palmitoylated protein 3 [Source:HGNC Symbol;Acc:HGNC:7221]
**-0,7780613**	4,17E-09	3,25E-08	*ARHGAP10*	Rho GTPase activating protein 10 [Source:HGNC Symbol;Acc:HGNC:26099]
**-0,777015658**	0,01030584	0,03049636	*LPAR4*	lysophosphatidic acid receptor 4 [Source:HGNC Symbol;Acc:HGNC:4478]
**-0,77664651**	5,04E-08	3,55E-07	*RNF152*	ring finger protein 152 [Source:HGNC Symbol;Acc:HGNC:26811]
**-0,775250148**	1,13E-10	1,01E-09	*FANCI*	FA complementation group I [Source:HGNC Symbol;Acc:HGNC:25568]
**-0,774783848**	1,33E-05	6,96E-05	*MARS2*	methionyl-tRNA synthetase 2, mitochondrial [Source:HGNC Symbol;Acc:HGNC:25133]
**-0,77475968**	1,72E-06	1,02E-05	*SERPING1*	serpin family G member 1 [Source:HGNC Symbol;Acc:HGNC:1228]
**-0,773436508**	1,22E-45	4,86E-44	*SERPINB6*	serpin family B member 6 [Source:HGNC Symbol;Acc:HGNC:8950]
**-0,773370997**	1,50E-20	2,48E-19	*TMPO*	thymopoietin [Source:HGNC Symbol;Acc:HGNC:11875]
**-0,771427878**	6,80E-46	2,73E-44	*CITED2*	Cbp/p300 interacting transactivator with Glu/Asp rich carboxy-terminal domain 2 [Source:HGNC Symbol;Acc:HGNC:1987]
**-0,771284643**	2,87E-27	6,28E-26	*TTL*	tubulin tyrosine ligase [Source:HGNC Symbol;Acc:HGNC:21586]
**-0,768292341**	3,63E-13	4,00E-12	*SNRPA1*	small nuclear ribonucleoprotein polypeptide A' [Source:HGNC Symbol;Acc:HGNC:11152]
**-0,768094687**	0,000552298	0,002198092	*KCNK1*	potassium two pore domain channel subfamily K member 1 [Source:HGNC Symbol;Acc:HGNC:6272]
**-0,767488029**	1,00E-17	1,45E-16	*FOXC2*	forkhead box C2 [Source:HGNC Symbol;Acc:HGNC:3801]
**-0,767290203**	1,80E-13	2,02E-12	*GAR1*	GAR1 ribonucleoprotein [Source:HGNC Symbol;Acc:HGNC:14264]
**-0,766664565**	3,39E-05	0,000167521	*RNF207*	ring finger protein 207 [Source:HGNC Symbol;Acc:HGNC:32947]
**-0,766660003**	0,001883182	0,00670428	*STBD1*	starch binding domain 1 [Source:HGNC Symbol;Acc:HGNC:24854]
**-0,765881063**	0,000130402	0,000585374	*DPY19L2*	dpy-19 like 2 [Source:HGNC Symbol;Acc:HGNC:19414]
**-0,765343685**	0,000152255	0,000673045	*DEXI*	Dexi homolog [Source:HGNC Symbol;Acc:HGNC:13267]
**-0,765026981**	1,50E-05	7,75E-05	*CENPN*	centromere protein N [Source:HGNC Symbol;Acc:HGNC:30873]
**-0,764902826**	3,20E-59	1,80E-57	*CKAP4*	cytoskeleton associated protein 4 [Source:HGNC Symbol;Acc:HGNC:16991]

**-0,764898754**	5,53E-15	6,76E-14	*SLC19A1*	solute carrier family 19 member 1 [Source:HGNC Symbol;Acc:HGNC:10937]
**-0,764412001**	7,21E-28	1,61E-26	*ATAD3A*	ATPase family, AAA domain containing 3A [Source:HGNC Symbol;Acc:HGNC:25567]
**-0,763603171**	7,77E-08	5,36E-07	*OPCML*	opioid binding protein/cell adhesion molecule like [Source:HGNC Symbol;Acc:HGNC:8143]
**-0,762118227**	5,58E-15	6,81E-14	*ATAD3B*	ATPase family, AAA domain containing 3B [Source:HGNC Symbol;Acc:HGNC:24007]
**-0,76199387**	0,000904754	0,003457592	*CENPQ*	centromere protein Q [Source:HGNC Symbol;Acc:HGNC:21347]
**-0,761722706**	6,59E-10	5,51E-09	*RFC2*	replication factor C subunit 2 [Source:HGNC Symbol;Acc:HGNC:9970]
**-0,760147085**	0,008706489	0,026333873	*KCNT2*	potassium sodium-activated channel subfamily T member 2 [Source:HGNC Symbol;Acc:HGNC:18866]
**-0,759707025**	3,93E-06	2,21E-05	*PLSCR1*	phospholipid scramblase 1 [Source:HGNC Symbol;Acc:HGNC:9092]
**-0,75932251**	2,60E-44	9,90E-43	*FBXO32*	F-box protein 32 [Source:HGNC Symbol;Acc:HGNC:16731]
**-0,756426728**	1,23E-42	4,51E-41	*MPZL1*	myelin protein zero like 1 [Source:HGNC Symbol;Acc:HGNC:7226]
**-0,756267636**	1,96E-07	1,29E-06	*LTV1*	LTV1 ribosome biogenesis factor [Source:HGNC Symbol;Acc:HGNC:21173]
**-0,756200734**	0,000170773	0,000747581	*PIF1*	PIF1 5'-to-3' DNA helicase [Source:HGNC Symbol;Acc:HGNC:26220]
**-0,756163886**	1,34E-14	1,60E-13	*CEBPD*	CCAAT enhancer binding protein delta [Source:HGNC Symbol;Acc:HGNC:1835]
**-0,75589444**	0,002681654	0,009235169	*BBOF1*	basal body orientation factor 1 [Source:HGNC Symbol;Acc:HGNC:19855]
**-0,755852845**	6,28E-50	2,82E-48	*GARS*	glycyl-tRNA synthetase [Source:HGNC Symbol;Acc:HGNC:4162]
**-0,754490849**	4,66E-20	7,53E-19	*KDELC2*	KDEL motif containing 2 [Source:HGNC Symbol;Acc:HGNC:28496]
**-0,754425841**	1,34E-06	8,05E-06	*RFC3*	replication factor C subunit 3 [Source:HGNC Symbol;Acc:HGNC:9971]
**-0,754287615**	2,87E-22	5,08E-21	*ANKRD13A*	ankyrin repeat domain 13A [Source:HGNC Symbol;Acc:HGNC:21268]
**-0,753747724**	0,000157818	0,000695417	*POC1A*	POC1 centriolar protein A [Source:HGNC Symbol;Acc:HGNC:24488]
**-0,75297081**	3,00E-06	1,71E-05	*SUSD2*	sushi domain containing 2 [Source:HGNC Symbol;Acc:HGNC:30667]
**-0,752740755**	8,16E-06	4,40E-05	*KCNK6*	potassium two pore domain channel subfamily K member 6 [Source:HGNC Symbol;Acc:HGNC:6281]
**-0,752515738**	1,29E-23	2,42E-22	*SRSF2*	serine and arginine rich splicing factor 2 [Source:HGNC Symbol;Acc:HGNC:10783]
**-0,752291699**	1,21E-05	6,35E-05	*FAM234B*	family with sequence similarity 234 member B [Source:HGNC Symbol;Acc:HGNC:29288]
**-0,752224021**	0,013010575	0,037347111	*PITX3*	paired like homeodomain 3 [Source:HGNC Symbol;Acc:HGNC:9006]
**-0,75132435**	1,99E-11	1,89E-10	*CHAF1A*	chromatin assembly factor 1 subunit A [Source:HGNC Symbol;Acc:HGNC:1910]
**-0,751271628**	9,10E-06	4,87E-05	*ARL13B*	ADP ribosylation factor like GTPase 13B [Source:HGNC Symbol;Acc:HGNC:25419]
**-0,750697187**	1,70E-21	2,92E-20	*PFAS*	phosphoribosylformylglycinamidine synthase [Source:HGNC Symbol;Acc:HGNC:8863]
**-0,750673072**	5,12E-09	3,96E-08	*PHYHIPL*	phytanoyl-CoA 2-hydroxylase interacting protein like [Source:HGNC Symbol;Acc:HGNC:29378]
**-0,750603075**	8,65E-30	2,09E-28	*RRAS*	RAS related [Source:HGNC Symbol;Acc:HGNC:10447]
**-0,749506022**	0,009834388	0,029270879	*SH3TC2*	SH3 domain and tetratricopeptide repeats 2 [Source:HGNC Symbol;Acc:HGNC:29427]
**-0,749468341**	5,97E-18	8,72E-17	*MCM2*	minichromosome maintenance complex component 2 [Source:HGNC Symbol;Acc:HGNC:6944]
**-0,748677851**	4,77E-70	3,30E-68	*ANXA6*	annexin A6 [Source:HGNC Symbol;Acc:HGNC:544]

**-0,748026453**	0,009521191	0,028452147	*CFAP157*	cilia and flagella associated protein 157 [Source:HGNC Symbol;Acc:HGNC:27843]
**-0,747995213**	7,70E-09	5,86E-08	*SUSD5*	sushi domain containing 5 [Source:HGNC Symbol;Acc:HGNC:29061]
**-0,747943314**	1,53E-21	2,64E-20	*LOXL3*	lysyl oxidase like 3 [Source:HGNC Symbol;Acc:HGNC:13869]
**-0,747468391**	8,24E-24	1,57E-22	*LOXL4*	lysyl oxidase like 4 [Source:HGNC Symbol;Acc:HGNC:17171]
**-0,747451393**	5,93E-38	1,89E-36	*ATP13A3*	ATPase 13A3 [Source:HGNC Symbol;Acc:HGNC:24113]
**-0,747351001**	2,32E-06	1,35E-05	*NAV1*	neuron navigator 1 [Source:HGNC Symbol;Acc:HGNC:15989]
**-0,746822973**	2,80E-10	2,42E-09	*ATP10A*	ATPase phospholipid transporting 10A (putative) [Source:HGNC Symbol;Acc:HGNC:13542]
**-0,746521839**	2,71E-10	2,34E-09	*RABEPK*	Rab9 effector protein with kelch motifs [Source:HGNC Symbol;Acc:HGNC:16896]
**-0,745955667**	1,62E-09	1,30E-08	*USP31*	ubiquitin specific peptidase 31 [Source:HGNC Symbol;Acc:HGNC:20060]
**-0,745841862**	3,15E-12	3,22E-11	*TOPBP1*	DNA topoisomerase II binding protein 1 [Source:HGNC Symbol;Acc:HGNC:17008]
**-0,745689819**	7,79E-09	5,93E-08	*INTU*	inturned planar cell polarity protein [Source:HGNC Symbol;Acc:HGNC:29239]
**-0,745541225**	2,04E-23	3,79E-22	*PMP22*	peripheral myelin protein 22 [Source:HGNC Symbol;Acc:HGNC:9118]
**-0,743938715**	3,35E-14	3,93E-13	*NEDD1*	neural precursor cell expressed, developmentally down-regulated 1 [Source:HGNC Symbol;Acc:HGNC:7723]
**-0,743407704**	0,003733806	0,012426384	*NCAN*	neurocan [Source:HGNC Symbol;Acc:HGNC:2465]
**-0,742609394**	0,000402311	0,001645712	*ITIH5*	inter-alpha-trypsin inhibitor heavy chain family member 5 [Source:HGNC Symbol;Acc:HGNC:21449]
**-0,742170314**	3,14E-45	1,23E-43	*PIEZO1*	piezo type mechanosensitive ion channel component 1 [Source:HGNC Symbol;Acc:HGNC:28993]
**-0,741571264**	4,66E-28	1,05E-26	*FRMD6*	FERM domain containing 6 [Source:HGNC Symbol;Acc:HGNC:19839]
**-0,739980826**	3,10E-49	1,37E-47	*SEPT11*	septin 11 [Source:HGNC Symbol;Acc:HGNC:25589]
**-0,739460944**	1,57E-29	3,77E-28	*TUBB2A*	tubulin beta 2A class IIa [Source:HGNC Symbol;Acc:HGNC:12412]
**-0,739420583**	0,003297742	0,011116203	*SAMD12*	sterile alpha motif domain containing 12 [Source:HGNC Symbol;Acc:HGNC:31750]
**-0,738687511**	4,59E-08	3,24E-07	*CMSS1*	cms1 ribosomal small subunit homolog (yeast) [Source:HGNC Symbol;Acc:HGNC:28666]
**-0,738336458**	3,45E-10	2,96E-09	*HHIPL1*	HHIP like 1 [Source:HGNC Symbol;Acc:HGNC:19710]
**-0,738162821**	9,85E-25	1,96E-23	*RCAN1*	regulator of calcineurin 1 [Source:HGNC Symbol;Acc:HGNC:3040]
**-0,73804822**	6,58E-13	7,12E-12	*SMC2*	structural maintenance of chromosomes 2 [Source:HGNC Symbol;Acc:HGNC:14011]
**-0,737721578**	0,000194513	0,000842947	*MECOM*	MDS1 and EVI1 complex locus [Source:HGNC Symbol;Acc:HGNC:3498]
**-0,737388721**	4,76E-30	1,17E-28	*MYBBP1A*	MYB binding protein 1a [Source:HGNC Symbol;Acc:HGNC:7546]
**-0,737250351**	8,78E-05	0,000406215	*PTPRB*	protein tyrosine phosphatase, receptor type B [Source:HGNC Symbol;Acc:HGNC:9665]
**-0,737097913**	3,82E-06	2,15E-05	*TLL2*	tolloid like 2 [Source:HGNC Symbol;Acc:HGNC:11844]
**-0,736299438**	7,50E-27	1,62E-25	*CTIF*	cap binding complex dependent translation initiation factor [Source:HGNC Symbol;Acc:HGNC:23925]
**-0,735330034**	2,35E-29	5,62E-28	*RFTN1*	raftlin, lipid raft linker 1 [Source:HGNC Symbol;Acc:HGNC:30278]
**-0,734370478**	1,40E-20	2,32E-19	*SBDS*	SBDS, ribosome maturation factor [Source:HGNC Symbol;Acc:HGNC:19440]
**-0,733871226**	3,43E-28	7,80E-27	*NASP*	nuclear autoantigenic sperm protein [Source:HGNC Symbol;Acc:HGNC:7644]
**-0,732582627**	1,86E-06	1,09E-05	*SIK1B*	salt inducible kinase 1B (putative) [Source:HGNC Symbol;Acc:HGNC:52389]

**-0,732283373**	1,01E-22	1,82E-21	*AMD1*	adenosylmethionine decarboxylase 1 [Source:HGNC Symbol;Acc:HGNC:457]
**-0,731347685**	8,72E-06	4,69E-05	*VRK1*	vaccinia related kinase 1 [Source:HGNC Symbol;Acc:HGNC:12718]
**-0,731275106**	0,000769273	0,002987495	*B3GNTL1*	UDP-GlcNAc:betaGal beta-1,3-N-acetylglucosaminyltransferase like 1 [Source:HGNC Symbol;Acc:HGNC:21727]
**-0,730482515**	4,20E-07	2,66E-06	*CENPO*	centromere protein O [Source:HGNC Symbol;Acc:HGNC:28152]
**-0,730356238**	0,00065804	0,002590775	*ZNF239*	zinc finger protein 239 [Source:HGNC Symbol;Acc:HGNC:13031]
**-0,729356806**	2,60E-24	5,09E-23	*STAT5B*	signal transducer and activator of transcription 5B [Source:HGNC Symbol;Acc:HGNC:11367]
**-0,728391634**	1,21E-27	2,69E-26	*CAD*	carbamoyl-phosphate synthetase 2, aspartate transcarbamylase, and dihydroorotase [Source:HGNC Symbol;Acc:HGNC:1424]
**-0,727877587**	1,59E-54	7,94E-53	*EZR*	ezrin [Source:HGNC Symbol;Acc:HGNC:12691]
**-0,727326258**	1,05E-23	1,97E-22	*GNL3*	G protein nucleolar 3 [Source:HGNC Symbol;Acc:HGNC:29931]
**-0,726580018**	1,86E-34	5,34E-33	*SRRT*	serrate, RNA effector molecule [Source:HGNC Symbol;Acc:HGNC:24101]
**-0,726502826**	3,07E-13	3,40E-12	*IER5L*	immediate early response 5 like [Source:HGNC Symbol;Acc:HGNC:23679]
**-0,725076284**	1,93E-36	5,92E-35	*UBE2S*	ubiquitin conjugating enzyme E2 S [Source:HGNC Symbol;Acc:HGNC:17895]
**-0,725076246**	8,85E-34	2,47E-32	*CAV2*	caveolin 2 [Source:HGNC Symbol;Acc:HGNC:1528]
**-0,724675467**	2,20E-06	1,28E-05	*RBM20*	RNA binding motif protein 20 [Source:HGNC Symbol;Acc:HGNC:27424]
**-0,7238687**	3,52E-10	3,02E-09	*EXOSC2*	exosome component 2 [Source:HGNC Symbol;Acc:HGNC:17097]
**-0,723525833**	9,65E-18	1,40E-16	*FKBP14*	FK506 binding protein 14 [Source:HGNC Symbol;Acc:HGNC:18625]
**-0,723491376**	4,08E-19	6,33E-18	*FAAP100*	FA core complex associated protein 100 [Source:HGNC Symbol;Acc:HGNC:26171]
**-0,722531832**	0,00065337	0,002574476	*CCDC18*	coiled-coil domain containing 18 [Source:HGNC Symbol;Acc:HGNC:30370]
**-0,721486003**	1,76E-05	9,03E-05	*INSYN2B*	inhibitory synaptic factor family member 2B [Source:HGNC Symbol;Acc:HGNC:37271]
**-0,721180726**	8,38E-41	2,89E-39	*MARS*	methionyl-tRNA synthetase [Source:HGNC Symbol;Acc:HGNC:6898]
**-0,719157063**	5,21E-28	1,18E-26	*SLC1A5*	solute carrier family 1 member 5 [Source:HGNC Symbol;Acc:HGNC:10943]
**-0,718829698**	1,29E-07	8,69E-07	*WDR4*	WD repeat domain 4 [Source:HGNC Symbol;Acc:HGNC:12756]
**-0,71841268**	5,43E-47	2,26E-45	*ALCAM*	activated leukocyte cell adhesion molecule [Source:HGNC Symbol;Acc:HGNC:400]
**-0,717181951**	1,04E-08	7,80E-08	*CENPX*	centromere protein X [Source:HGNC Symbol;Acc:HGNC:11422]
**-0,716999139**	8,66E-07	5,31E-06	*LCLAT1*	lysocardiolipin acyltransferase 1 [Source:HGNC Symbol;Acc:HGNC:26756]
**-0,71678871**	4,46E-39	1,46E-37	*NPTN*	neuroplastin [Source:HGNC Symbol;Acc:HGNC:17867]
**-0,716533069**	7,67E-20	1,23E-18	*RIC1*	RIC1 homolog, RAB6A GEF complex partner 1 [Source:HGNC Symbol;Acc:HGNC:17686]
**-0,716232296**	1,21E-42	4,45E-41	*TRAM2*	translocation associated membrane protein 2 [Source:HGNC Symbol;Acc:HGNC:16855]
**-0,716079226**	7,99E-13	8,60E-12	*RELB*	RELB proto-oncogene, NF-kB subunit [Source:HGNC Symbol;Acc:HGNC:9956]
**-0,714591169**	4,89E-32	1,29E-30	*KCTD20*	potassium channel tetramerization domain containing 20 [Source:HGNC Symbol;Acc:HGNC:21052]
**-0,713292025**	0,018069064	0,049575606	*DSC2*	desmocollin 2 [Source:HGNC Symbol;Acc:HGNC:3036]

**-0,712941314**	7,70E-19	1,18E-17	*MICAL3*	microtubule associated monooxygenase, calponin and LIM domain containing 3 [Source:HGNC Symbol;Acc:HGNC:24694]
**-0,7128309**	2,05E-06	1,20E-05	*NXT1*	nuclear transport factor 2 like export factor 1 [Source:HGNC Symbol;Acc:HGNC:15913]
**-0,712433017**	5,36E-11	4,90E-10	*MYO19*	myosin XIX [Source:HGNC Symbol;Acc:HGNC:26234]
**-0,711255675**	1,96E-06	1,15E-05	*TRIM14*	tripartite motif containing 14 [Source:HGNC Symbol;Acc:HGNC:16283]
**-0,710940633**	4,92E-65	3,18E-63	*MYL6*	myosin light chain 6 [Source:HGNC Symbol;Acc:HGNC:7587]
**-0,710922119**	1,03E-29	2,49E-28	*NOLC1*	nucleolar and coiled-body phosphoprotein 1 [Source:HGNC Symbol;Acc:HGNC:15608]
**-0,71013498**	0,000119134	0,000538156	*TSEN2*	tRNA splicing endonuclease subunit 2 [Source:HGNC Symbol;Acc:HGNC:28422]
**-0,708591744**	5,57E-45	2,17E-43	*SERPINH1*	serpin family H member 1 [Source:HGNC Symbol;Acc:HGNC:1546]
**-0,707462477**	6,92E-06	3,77E-05	*APBA1*	amyloid beta precursor protein binding family A member 1 [Source:HGNC Symbol;Acc:HGNC:578]
**-0,707252867**	4,17E-06	2,34E-05	*CDKN2C*	cyclin dependent kinase inhibitor 2C [Source:HGNC Symbol;Acc:HGNC:1789]
**-0,707122233**	1,05E-06	6,36E-06	*SUV39H2*	suppressor of variegation 3-9 homolog 2 [Source:HGNC Symbol;Acc:HGNC:17287]
**-0,706623897**	6,51E-17	8,95E-16	*RRP7A*	ribosomal RNA processing 7 homolog A [Source:HGNC Symbol;Acc:HGNC:24286]
**-0,706520727**	2,69E-11	2,54E-10	*NOC3L*	NOC3 like DNA replication regulator [Source:HGNC Symbol;Acc:HGNC:24034]
**-0,706449332**	7,18E-09	5,48E-08	*TMEM206*	transmembrane protein 206 [Source:HGNC Symbol;Acc:HGNC:25593]
**-0,706314069**	2,68E-06	1,54E-05	*LUM*	lumican [Source:HGNC Symbol;Acc:HGNC:6724]
**-0,706054916**	3,57E-19	5,57E-18	*TRAF3*	TNF receptor associated factor 3 [Source:HGNC Symbol;Acc:HGNC:12033]
**-0,705919491**	3,86E-16	5,03E-15	*ZNHIT6*	zinc finger HIT-type containing 6 [Source:HGNC Symbol;Acc:HGNC:26089]
**-0,70572582**	4,89E-14	5,69E-13	*PTPN21*	protein tyrosine phosphatase, non-receptor type 21 [Source:HGNC Symbol;Acc:HGNC:9651]
**-0,705194485**	6,51E-05	0,000308341	*GUCY1A2*	guanylate cyclase 1 soluble subunit alpha 2 [Source:HGNC Symbol;Acc:HGNC:4684]
**-0,70466109**	5,93E-13	6,45E-12	*TLNRD1*	talin rod domain containing 1 [Source:HGNC Symbol;Acc:HGNC:13519]
**-0,704109629**	1,57E-31	4,09E-30	*GALNT1*	polypeptide N-acetylgalactosaminyltransferase 1 [Source:HGNC Symbol;Acc:HGNC:4123]
**-0,703199124**	5,63E-12	5,60E-11	*COL16A1*	collagen type XVI alpha 1 chain [Source:HGNC Symbol;Acc:HGNC:2193]
**-0,702604651**	2,39E-06	1,38E-05	*LPAR1*	lysophosphatidic acid receptor 1 [Source:HGNC Symbol;Acc:HGNC:3166]
**-0,702340965**	2,83E-05	0,000141396	*ADAM33*	ADAM metallopeptidase domain 33 [Source:HGNC Symbol;Acc:HGNC:15478]
**-0,701909973**	9,39E-14	1,07E-12	*DHFR*	dihydrofolate reductase [Source:HGNC Symbol;Acc:HGNC:2861]
**-0,701414858**	0,000117264	0,0005302	*GALNT18*	polypeptide N-acetylgalactosaminyltransferase 18 [Source:HGNC Symbol;Acc:HGNC:30488]
**-0,701414831**	2,38E-12	2,46E-11	*NIP7*	NIP7, nucleolar pre-rRNA processing protein [Source:HGNC Symbol;Acc:HGNC:24328]
**-0,701347565**	2,05E-13	2,29E-12	*PRR5L*	proline rich 5 like [Source:HGNC Symbol;Acc:HGNC:25878]
**-0,701308284**	2,00E-32	5,36E-31	*CEMIP2*	cell migration inducing hyaluronidase 2 [Source:HGNC Symbol;Acc:HGNC:11869]
**-0,701150061**	1,27E-14	1,52E-13	*KDELR3*	KDEL endoplasmic reticulum protein retention receptor 3 [Source:HGNC Symbol;Acc:HGNC:6306]
**-0,700786956**	0,0125587	0,0362508	*CHEK2*	checkpoint kinase 2 [Source:HGNC Symbol;Acc:HGNC:16627]

**-0,700641206**	0,00453445	0,014806584	*PINX1*	PIN2 (TERF1) interacting telomerase inhibitor 1 [Source:HGNC Symbol;Acc:HGNC:30046]
**-0,700464464**	0,011697541	0,034104941	*FGF18*	fibroblast growth factor 18 [Source:HGNC Symbol;Acc:HGNC:3674]
**-0,70040573**	1,96E-23	3,64E-22	*STK38*	serine/threonine kinase 38 [Source:HGNC Symbol;Acc:HGNC:17847]
**-0,700379509**	4,35E-47	1,81E-45	*PICALM*	phosphatidylinositol binding clathrin assembly protein [Source:HGNC Symbol;Acc:HGNC:15514]
**-0,700339342**	6,17E-19	9,50E-18	*DKC1*	dyskerin pseudouridine synthase 1 [Source:HGNC Symbol;Acc:HGNC:2890]
**-0,699727933**	2,62E-31	6,76E-30	*IARS*	isoleucyl-tRNA synthetase [Source:HGNC Symbol;Acc:HGNC:5330]
**-0,699061822**	1,70E-06	1,00E-05	*FN1*	fibronectin 1 [Source:HGNC Symbol;Acc:HGNC:3778]
**-0,698781454**	4,26E-07	2,70E-06	*HSPB8*	heat shock protein family B (small) member 8 [Source:HGNC Symbol;Acc:HGNC:30171]
**-0,698402339**	3,33E-26	7,04E-25	*RAB11FIP5*	RAB11 family interacting protein 5 [Source:HGNC Symbol;Acc:HGNC:24845]
**-0,698263839**	7,73E-28	1,72E-26	*PODXL*	podocalyxin like [Source:HGNC Symbol;Acc:HGNC:9171]
**-0,698115268**	0,000207585	0,000892424	*THSD1*	thrombospondin type 1 domain containing 1 [Source:HGNC Symbol;Acc:HGNC:17754]
**-0,697989691**	0,000493783	0,001983925	*LRRC3B*	leucine rich repeat containing 3B [Source:HGNC Symbol;Acc:HGNC:28105]
**-0,697866223**	3,04E-05	0,000151533	*FLRT2*	fibronectin leucine rich transmembrane protein 2 [Source:HGNC Symbol;Acc:HGNC:3761]
**-0,697771939**	5,88E-09	4,52E-08	*PRR11*	proline rich 11 [Source:HGNC Symbol;Acc:HGNC:25619]
**-0,697722663**	8,44E-29	1,98E-27	*TOMM40*	translocase of outer mitochondrial membrane 40 [Source:HGNC Symbol;Acc:HGNC:18001]
**-0,697530493**	4,83E-13	5,29E-12	*LACTB*	lactamase beta [Source:HGNC Symbol;Acc:HGNC:16468]
**-0,697389495**	5,71E-10	4,80E-09	*FER*	FER tyrosine kinase [Source:HGNC Symbol;Acc:HGNC:3655]
**-0,697351525**	0,008156986	0,024861704	*TNFAIP8*	TNF alpha induced protein 8 [Source:HGNC Symbol;Acc:HGNC:17260]
**-0,696686201**	0,001096238	0,004112428	*FANCM*	FA complementation group M [Source:HGNC Symbol;Acc:HGNC:23168]
**-0,695406854**	8,73E-05	0,000404118	*SNAPC1*	small nuclear RNA activating complex polypeptide 1 [Source:HGNC Symbol;Acc:HGNC:11134]
**-0,693787797**	1,48E-10	1,31E-09	*TMEM170B*	transmembrane protein 170B [Source:HGNC Symbol;Acc:HGNC:34244]
**-0,693734765**	3,39E-29	8,06E-28	*FAM98A*	family with sequence similarity 98 member A [Source:HGNC Symbol;Acc:HGNC:24520]
**-0,693730295**	6,22E-09	4,77E-08	*KCNIP3*	potassium voltage-gated channel interacting protein 3 [Source:HGNC Symbol;Acc:HGNC:15523]
**-0,693642684**	0,002047218	0,007244404	*ADCY7*	adenylate cyclase 7 [Source:HGNC Symbol;Acc:HGNC:238]
**-0,693638951**	4,66E-11	4,28E-10	*NBEAL2*	neurobeachin like 2 [Source:HGNC Symbol;Acc:HGNC:31928]
**-0,692845308**	3,55E-15	4,40E-14	*TDG*	thymine DNA glycosylase [Source:HGNC Symbol;Acc:HGNC:11700]
**-0,692818999**	4,92E-18	7,23E-17	*MRTO4*	MRT4 homolog, ribosome maturation factor [Source:HGNC Symbol;Acc:HGNC:18477]
**-0,692045222**	7,51E-28	1,68E-26	*CHSY1*	chondroitin sulfate synthase 1 [Source:HGNC Symbol;Acc:HGNC:17198]
**-0,691881801**	0,001315803	0,004849851	*NKX6-1*	NK6 homeobox 1 [Source:HGNC Symbol;Acc:HGNC:7839]
**-0,691056285**	1,56E-24	3,08E-23	*GTPBP4*	GTP binding protein 4 [Source:HGNC Symbol;Acc:HGNC:21535]
**-0,69088732**	3,39E-09	2,66E-08	*AIMP2*	aminoacyl tRNA synthetase complex interacting multifunctional protein 2 [Source:HGNC Symbol;Acc:HGNC:20609]
**-0,690787918**	1,34E-18	2,03E-17	*TUBG1*	tubulin gamma 1 [Source:HGNC Symbol;Acc:HGNC:12417]

**-0,690363648**	6,07E-06	3,34E-05	*MASTL*	microtubule associated serine/threonine kinase like [Source:HGNC Symbol;Acc:HGNC:19042]
**-0,690325627**	2,86E-28	6,53E-27	*ADAM12*	ADAM metallopeptidase domain 12 [Source:HGNC Symbol;Acc:HGNC:190]
**-0,690318293**	3,27E-31	8,39E-30	*TMEM165*	transmembrane protein 165 [Source:HGNC Symbol;Acc:HGNC:30760]
**-0,689449103**	7,13E-19	1,09E-17	*VLDLR*	very low density lipoprotein receptor [Source:HGNC Symbol;Acc:HGNC:12698]
**-0,688711921**	7,51E-43	2,78E-41	*ARPC5*	actin related protein 2/3 complex subunit 5 [Source:HGNC Symbol;Acc:HGNC:708]
**-0,688707062**	2,29E-09	1,82E-08	*ITPR2*	inositol 1,4,5-trisphosphate receptor type 2 [Source:HGNC Symbol;Acc:HGNC:6181]
**-0,687731304**	3,02E-13	3,34E-12	*NUP107*	nucleoporin 107 [Source:HGNC Symbol;Acc:HGNC:29914]
**-0,686427964**	6,61E-46	2,66E-44	*EPHA2*	EPH receptor A2 [Source:HGNC Symbol;Acc:HGNC:3386]
**-0,686085909**	9,90E-27	2,13E-25	*FLNC*	filamin C [Source:HGNC Symbol;Acc:HGNC:3756]
**-0,685885881**	1,86E-50	8,41E-49	*MALT1*	MALT1 paracaspase [Source:HGNC Symbol;Acc:HGNC:6819]
**-0,685354541**	2,47E-42	8,91E-41	*NDFIP1*	Nedd4 family interacting protein 1 [Source:HGNC Symbol;Acc:HGNC:17592]
**-0,685306641**	0,000751859	0,002924551	*ZFPM2*	zinc finger protein, FOG family member 2 [Source:HGNC Symbol;Acc:HGNC:16700]
**-0,684475559**	8,94E-18	1,30E-16	*GBP3*	guanylate binding protein 3 [Source:HGNC Symbol;Acc:HGNC:4184]
**-0,68440576**	6,23E-10	5,22E-09	*SLC35F3*	solute carrier family 35 member F3 [Source:HGNC Symbol;Acc:HGNC:23616]
**-0,683791367**	2,69E-06	1,54E-05	*C4orf46*	chromosome 4 open reading frame 46 [Source:HGNC Symbol;Acc:HGNC:27320]
**-0,683349708**	8,78E-19	1,34E-17	*WWC2*	WW and C2 domain containing 2 [Source:HGNC Symbol;Acc:HGNC:24148]
**-0,682356033**	1,51E-42	5,52E-41	*CCT5*	chaperonin containing TCP1 subunit 5 [Source:HGNC Symbol;Acc:HGNC:1618]
**-0,682296811**	3,31E-25	6,69E-24	*TIMM17A*	translocase of inner mitochondrial membrane 17A [Source:HGNC Symbol;Acc:HGNC:17315]
**-0,681026457**	1,49E-41	5,26E-40	*WNT5A*	Wnt family member 5A [Source:HGNC Symbol;Acc:HGNC:12784]
**-0,680136264**	0,012589	0,036327456	*MITF*	melanogenesis associated transcription factor [Source:HGNC Symbol;Acc:HGNC:7105]
**-0,680082784**	0,004610356	0,015024108	*GBX2*	gastrulation brain homeobox 2 [Source:HGNC Symbol;Acc:HGNC:4186]
**-0,678930542**	0,006454024	0,020296028	*CENPP*	centromere protein P [Source:HGNC Symbol;Acc:HGNC:32933]
**-0,678687107**	0,000115301	0,000522054	*JAZF1*	JAZF zinc finger 1 [Source:HGNC Symbol;Acc:HGNC:28917]
**-0,678279691**	3,88E-48	1,66E-46	*KIRREL1*	kirre like nephrin family adhesion molecule 1 [Source:HGNC Symbol;Acc:HGNC:15734]
**-0,677919277**	2,06E-34	5,89E-33	*MTCL1*	microtubule crosslinking factor 1 [Source:HGNC Symbol;Acc:HGNC:29121]
**-0,677430058**	0,000103023	0,000470968	*TIFA*	TRAF interacting protein with forkhead associated domain [Source:HGNC Symbol;Acc:HGNC:19075]
**-0,677334356**	0,000718066	0,002803779	*ZNF486*	zinc finger protein 486 [Source:HGNC Symbol;Acc:HGNC:20807]
**-0,677217679**	0,000199347	0,000860994	*PAQR4*	progestin and adipoQ receptor family member 4 [Source:HGNC Symbol;Acc:HGNC:26386]
**-0,677169205**	1,17E-05	6,16E-05	*XRCC3*	X-ray repair cross complementing 3 [Source:HGNC Symbol;Acc:HGNC:12830]
**-0,677156412**	7,72E-06	4,18E-05	*BOLA3*	bolA family member 3 [Source:HGNC Symbol;Acc:HGNC:24415]
**-0,676273632**	1,48E-46	6,11E-45	*SRSF3*	serine and arginine rich splicing factor 3 [Source:HGNC Symbol;Acc:HGNC:10785]
**-0,67511431**	3,55E-24	6,89E-23	*ITSN1*	intersectin 1 [Source:HGNC Symbol;Acc:HGNC:6183]
**-0,674652028**	0,002329109	0,008139508	*HMCN1*	hemicentin 1 [Source:HGNC Symbol;Acc:HGNC:19194]

**-0,674398943**	0,000750263	0,002919341	*TEX30*	testis expressed 30 [Source:HGNC Symbol;Acc:HGNC:25188]
**-0,674171411**	4,06E-35	1,19E-33	*SOD2*	superoxide dismutase 2 [Source:HGNC Symbol;Acc:HGNC:11180]
**-0,673545144**	1,10E-23	2,08E-22	*TGM2*	transglutaminase 2 [Source:HGNC Symbol;Acc:HGNC:11778]
**-0,673101345**	2,13E-50	9,60E-49	*SFPQ*	splicing factor proline and glutamine rich [Source:HGNC Symbol;Acc:HGNC:10774]
**-0,672575673**	0,000175446	0,000766653	*TRAF1*	TNF receptor associated factor 1 [Source:HGNC Symbol;Acc:HGNC:12031]
**-0,672484802**	1,84E-34	5,31E-33	*SLC12A4*	solute carrier family 12 member 4 [Source:HGNC Symbol;Acc:HGNC:10913]
**-0,672406551**	4,24E-05	0,000207031	*TVP23A*	trans-golgi network vesicle protein 23 homolog A [Source:HGNC Symbol;Acc:HGNC:20398]
**-0,671800284**	0,009674627	0,028844033	*GUCY1B1*	guanylate cyclase 1 soluble subunit beta 1 [Source:HGNC Symbol;Acc:HGNC:4687]
**-0,670877571**	3,98E-15	4,91E-14	*CARS2*	cysteinyl-tRNA synthetase 2, mitochondrial [Source:HGNC Symbol;Acc:HGNC:25695]
**-0,670622605**	1,22E-37	3,86E-36	*EHD1*	EH domain containing 1 [Source:HGNC Symbol;Acc:HGNC:3242]
**-0,670402615**	5,61E-28	1,26E-26	*RBMS1*	RNA binding motif single stranded interacting protein 1 [Source:HGNC Symbol;Acc:HGNC:9907]
**-0,670280812**	1,70E-41	5,98E-40	*SSR3*	signal sequence receptor subunit 3 [Source:HGNC Symbol;Acc:HGNC:11325]
**-0,670113127**	0,000941847	0,00358461	*TIPIN*	TIMELESS interacting protein [Source:HGNC Symbol;Acc:HGNC:30750]
**-0,669756284**	0,000365126	0,001502149	*TEDC2*	tubulin epsilon and delta complex 2 [Source:HGNC Symbol;Acc:HGNC:25849]
**-0,669051572**	4,74E-13	5,20E-12	*FAM107A*	family with sequence similarity 107 member A [Source:HGNC Symbol;Acc:HGNC:30827]
**-0,667973596**	1,02E-38	3,32E-37	*THY1*	Thy-1 cell surface antigen [Source:HGNC Symbol;Acc:HGNC:11801]
**-0,6675272**	0,003027199	0,010306285	*APOBEC3B*	apolipoprotein B mRNA editing enzyme catalytic subunit 3B [Source:HGNC Symbol;Acc:HGNC:17352]
**-0,667309089**	9,49E-24	1,80E-22	*ICMT*	isoprenylcysteine carboxyl methyltransferase [Source:HGNC Symbol;Acc:HGNC:5350]
**-0,667221858**	1,16E-08	8,66E-08	*RARB*	retinoic acid receptor beta [Source:HGNC Symbol;Acc:HGNC:9865]
**-0,666501367**	7,92E-05	0,000369052	*ZNF649*	zinc finger protein 649 [Source:HGNC Symbol;Acc:HGNC:25741]
**-0,666281192**	2,93E-14	3,46E-13	*METRNL*	meteorin like, glial cell differentiation regulator [Source:HGNC Symbol;Acc:HGNC:27584]
**-0,665635496**	0,000475017	0,001916859	*ARAP2*	ArfGAP with RhoGAP domain, ankyrin repeat and PH domain 2 [Source:HGNC Symbol;Acc:HGNC:16924]
**-0,665482313**	3,32E-06	1,88E-05	*CLN6*	CLN6, transmembrane ER protein [Source:HGNC Symbol;Acc:HGNC:2077]
**-0,664423882**	1,93E-07	1,27E-06	*SMAD7*	SMAD family member 7 [Source:HGNC Symbol;Acc:HGNC:6773]
**-0,663972885**	0,003017476	0,01027679	*DNA2*	DNA replication helicase/nuclease 2 [Source:HGNC Symbol;Acc:HGNC:2939]
**-0,663897167**	9,01E-14	1,03E-12	*PPIF*	peptidylprolyl isomerase F [Source:HGNC Symbol;Acc:HGNC:9259]
**-0,663056633**	2,05E-09	1,64E-08	*CCDC14*	coiled-coil domain containing 14 [Source:HGNC Symbol;Acc:HGNC:25766]
**-0,662634512**	1,99E-08	1,46E-07	*CLCF1*	cardiotrophin like cytokine factor 1 [Source:HGNC Symbol;Acc:HGNC:17412]
**-0,66192371**	1,26E-11	1,22E-10	*CENPV*	centromere protein V [Source:HGNC Symbol;Acc:HGNC:29920]
**-0,661799422**	1,25E-25	2,60E-24	*H2AFZ*	H2A histone family member Z [Source:HGNC Symbol;Acc:HGNC:4741]
**-0,66050402**	9,47E-18	1,38E-16	*FAM84B*	family with sequence similarity 84 member B [Source:HGNC Symbol;Acc:HGNC:24166]

**-0,659984566**	5,23E-17	7,22E-16	*PEAK1*	pseudopodium enriched atypical kinase 1 [Source:HGNC Symbol;Acc:HGNC:29431]
**-0,659877871**	4,22E-29	1,00E-27	*MAPRE1*	microtubule associated protein RP/EB family member 1 [Source:HGNC Symbol;Acc:HGNC:6890]
**-0,659641251**	3,30E-28	7,52E-27	*SBNO2*	strawberry notch homolog 2 [Source:HGNC Symbol;Acc:HGNC:29158]
**-0,659242115**	1,06E-14	1,27E-13	*ACOT9*	acyl-CoA thioesterase 9 [Source:HGNC Symbol;Acc:HGNC:17152]
**-0,658972771**	3,16E-06	1,80E-05	*CCNJ*	cyclin J [Source:HGNC Symbol;Acc:HGNC:23434]
**-0,656880972**	8,70E-11	7,81E-10	*SPARC*	secreted protein acidic and cysteine rich [Source:HGNC Symbol;Acc:HGNC:11219]
**-0,656736706**	6,69E-08	4,66E-07	*GBP2*	guanylate binding protein 2 [Source:HGNC Symbol;Acc:HGNC:4183]
**-0,656721255**	0,006812681	0,021238054	*ZNF749*	zinc finger protein 749 [Source:HGNC Symbol;Acc:HGNC:32783]
**-0,656666608**	3,94E-22	6,95E-21	*MTHFD1*	methylenetetrahydrofolate dehydrogenase, cyclohydrolase and formyltetrahydrofolate synthetase 1 [Source:HGNC Symbol;Acc:HGNC:7432]
**-0,656080904**	1,11E-09	9,04E-09	*PHACTR2*	phosphatase and actin regulator 2 [Source:HGNC Symbol;Acc:HGNC:20956]
**-0,655641736**	2,31E-18	3,43E-17	*TCERG1*	transcription elongation regulator 1 [Source:HGNC Symbol;Acc:HGNC:15630]
**-0,655463278**	5,25E-10	4,43E-09	*KCTD11*	potassium channel tetramerization domain containing 11 [Source:HGNC Symbol;Acc:HGNC:21302]
**-0,655120853**	0,000404081	0,001651559	*NRIP3*	nuclear receptor interacting protein 3 [Source:HGNC Symbol;Acc:HGNC:1167]
**-0,654104132**	1,31E-13	1,48E-12	*SRSF1*	serine and arginine rich splicing factor 1 [Source:HGNC Symbol;Acc:HGNC:10780]
**-0,653341138**	4,96E-12	4,96E-11	*ADARB1*	adenosine deaminase, RNA specific B1 [Source:HGNC Symbol;Acc:HGNC:226]
**-0,653194499**	2,82E-14	3,32E-13	*FOSL1*	FOS like 1, AP-1 transcription factor subunit [Source:HGNC Symbol;Acc:HGNC:13718]
**-0,653145018**	7,49E-40	2,50E-38	*PLOD1*	procollagen-lysine,2-oxoglutarate 5-dioxygenase 1 [Source:HGNC Symbol;Acc:HGNC:9081]
**-0,653133955**	4,74E-13	5,20E-12	*PPIL1*	peptidylprolyl isomerase like 1 [Source:HGNC Symbol;Acc:HGNC:9260]
**-0,653092624**	6,83E-05	0,000322318	*SP140L*	SP140 nuclear body protein like [Source:HGNC Symbol;Acc:HGNC:25105]
**-0,653014018**	3,64E-10	3,11E-09	*REEP4*	receptor accessory protein 4 [Source:HGNC Symbol;Acc:HGNC:26176]
**-0,652959986**	7,39E-13	7,96E-12	*RCC1*	regulator of chromosome condensation 1 [Source:HGNC Symbol;Acc:HGNC:1913]
**-0,651759291**	6,38E-12	6,31E-11	*NIFK*	nucleolar protein interacting with the FHA domain of MKI67 [Source:HGNC Symbol;Acc:HGNC:17838]
**-0,651584524**	0,006566363	0,020599231	*NECAB1*	N-terminal EF-hand calcium binding protein 1 [Source:HGNC Symbol;Acc:HGNC:20983]
**-0,650428475**	2,78E-15	3,46E-14	*GGH*	gamma-glutamyl hydrolase [Source:HGNC Symbol;Acc:HGNC:4248]
**-0,65018509**	2,93E-06	1,68E-05	*TMEM45A*	transmembrane protein 45A [Source:HGNC Symbol;Acc:HGNC:25480]
**-0,649371015**	1,29E-30	3,24E-29	*COLGALT1*	collagen beta(1-O)galactosyltransferase 1 [Source:HGNC Symbol;Acc:HGNC:26182]
**-0,647949385**	0,002325053	0,008129727	*TTLL11*	tubulin tyrosine ligase like 11 [Source:HGNC Symbol;Acc:HGNC:18113]
**-0,647706063**	3,74E-16	4,88E-15	*FARSB*	phenylalanyl-tRNA synthetase subunit beta [Source:HGNC Symbol;Acc:HGNC:17800]
**-0,647595312**	2,30E-18	3,42E-17	*TSPAN4*	tetraspanin 4 [Source:HGNC Symbol;Acc:HGNC:11859]
**-0,647282727**	1,07E-09	8,73E-09	*C1R*	complement C1r [Source:HGNC Symbol;Acc:HGNC:1246]
**-0,647214632**	1,22E-56	6,48E-55	*CLIC4*	chloride intracellular channel 4 [Source:HGNC Symbol;Acc:HGNC:13518]
**-0,646518728**	9,11E-16	1,16E-14	*PAX8*	paired box 8 [Source:HGNC Symbol;Acc:HGNC:8622]

**-0,646025208**	0,000983429	0,003725245	*GRID1*	glutamate ionotropic receptor delta type subunit 1 [Source:HGNC Symbol;Acc:HGNC:4575]
**-0,645488592**	3,41E-05	0,000168383	*KLHL29*	kelch like family member 29 [Source:HGNC Symbol;Acc:HGNC:29404]
**-0,64541374**	1,59E-12	1,67E-11	*HAUS6*	HAUS augmin like complex subunit 6 [Source:HGNC Symbol;Acc:HGNC:25948]
**-0,645101492**	2,42E-06	1,40E-05	*MAK16*	MAK16 homolog [Source:HGNC Symbol;Acc:HGNC:13703]
**-0,644992555**	1,29E-09	1,05E-08	*C1S*	complement C1s [Source:HGNC Symbol;Acc:HGNC:1247]
**-0,644954599**	1,03E-42	3,80E-41	*ITGA5*	integrin subunit alpha 5 [Source:HGNC Symbol;Acc:HGNC:6141]
**-0,644359758**	0,007976338	0,024372352	*RFXAP*	regulatory factor X associated protein [Source:HGNC Symbol;Acc:HGNC:9988]
**-0,643784701**	6,45E-08	4,49E-07	*VDR*	vitamin D receptor [Source:HGNC Symbol;Acc:HGNC:12679]
**-0,643748679**	6,84E-08	4,76E-07	*GATM*	glycine amidinotransferase [Source:HGNC Symbol;Acc:HGNC:4175]
**-0,642077906**	1,52E-26	3,25E-25	*DCLK1*	doublecortin like kinase 1 [Source:HGNC Symbol;Acc:HGNC:2700]
**-0,641821385**	4,42E-12	4,44E-11	*LAMA1*	laminin subunit alpha 1 [Source:HGNC Symbol;Acc:HGNC:6481]
**-0,641432442**	8,85E-05	0,000409444	*FBXO17*	F-box protein 17 [Source:HGNC Symbol;Acc:HGNC:18754]
**-0,641035337**	2,67E-15	3,33E-14	*ILDR2*	immunoglobulin like domain containing receptor 2 [Source:HGNC Symbol;Acc:HGNC:18131]
**-0,640798045**	0,002548142	0,008819197	*P2RY11*	purinergic receptor P2Y11 [Source:HGNC Symbol;Acc:HGNC:8540]
**-0,640333003**	2,44E-28	5,61E-27	*FJX1*	four jointed box 1 [Source:HGNC Symbol;Acc:HGNC:17166]
**-0,64011043**	1,08E-22	1,95E-21	*RANBP1*	RAN binding protein 1 [Source:HGNC Symbol;Acc:HGNC:9847]
**-0,639570521**	7,62E-29	1,79E-27	*TWSG1*	twisted gastrulation BMP signaling modulator 1 [Source:HGNC Symbol;Acc:HGNC:12429]
**-0,639465523**	2,16E-43	8,08E-42	*DKK3*	dickkopf WNT signaling pathway inhibitor 3 [Source:HGNC Symbol;Acc:HGNC:2893]
**-0,639169123**	2,08E-41	7,30E-40	*ERRFI1*	ERBB receptor feedback inhibitor 1 [Source:HGNC Symbol;Acc:HGNC:18185]
**-0,639019649**	2,58E-08	1,87E-07	*KLF5*	Kruppel like factor 5 [Source:HGNC Symbol;Acc:HGNC:6349]
**-0,638793355**	1,64E-09	1,32E-08	*SKP2*	S-phase kinase associated protein 2 [Source:HGNC Symbol;Acc:HGNC:10901]
**-0,638724308**	0,001695866	0,006095569	*CCDC34*	coiled-coil domain containing 34 [Source:HGNC Symbol;Acc:HGNC:25079]
**-0,638653457**	2,84E-05	0,000141819	*TRIM47*	tripartite motif containing 47 [Source:HGNC Symbol;Acc:HGNC:19020]
**-0,638214352**	0,000418861	0,001708368	*ZNF107*	zinc finger protein 107 [Source:HGNC Symbol;Acc:HGNC:12887]
**-0,637015157**	2,63E-06	1,51E-05	*RPGRIP1L*	RPGRIP1 like [Source:HGNC Symbol;Acc:HGNC:29168]
**-0,636366994**	0,007902754	0,024185592	*PRKG1*	protein kinase cGMP-dependent 1 [Source:HGNC Symbol;Acc:HGNC:9414]
**-0,636351669**	8,73E-05	0,000404118	*PRIM2*	DNA primase subunit 2 [Source:HGNC Symbol;Acc:HGNC:9370]
**-0,63593933**	4,58E-05	0,000221945	*SETMAR*	SET domain and mariner transposase fusion gene [Source:HGNC Symbol;Acc:HGNC:10762]
**-0,635655476**	2,54E-05	0,000127879	*CCNF*	cyclin F [Source:HGNC Symbol;Acc:HGNC:1591]
**-0,63559177**	1,47E-18	2,21E-17	*USB1*	U6 snRNA biogenesis phosphodiesterase 1 [Source:HGNC Symbol;Acc:HGNC:25792]
**-0,635551659**	3,81E-17	5,30E-16	*VKORC1L1*	vitamin K epoxide reductase complex subunit 1 like 1 [Source:HGNC Symbol;Acc:HGNC:21492]
**-0,634702415**	5,48E-22	9,59E-21	*TGFBR1*	transforming growth factor beta receptor 1 [Source:HGNC Symbol;Acc:HGNC:11772]
**-0,634480845**	0,005788663	0,018397445	*SFXN2*	sideroflexin 2 [Source:HGNC Symbol;Acc:HGNC:16086]

**-0,634138348**	7,06E-11	6,39E-10	*RFWD3*	ring finger and WD repeat domain 3 [Source:HGNC Symbol;Acc:HGNC:25539]
**-0,634087486**	1,74E-21	2,98E-20	*PKD1*	polycystin 1, transient receptor potential channel interacting [Source:HGNC Symbol;Acc:HGNC:9008]
**-0,6337775**	1,14E-52	5,38E-51	*HSPA8*	heat shock protein family A (Hsp70) member 8 [Source:HGNC Symbol;Acc:HGNC:5241]
**-0,633719292**	1,93E-06	1,13E-05	*CDCA7*	cell division cycle associated 7 [Source:HGNC Symbol;Acc:HGNC:14628]
**-0,633097284**	2,96E-16	3,90E-15	*GRWD1*	glutamate rich WD repeat containing 1 [Source:HGNC Symbol;Acc:HGNC:21270]
**-0,632965721**	6,03E-29	1,42E-27	*EML1*	echinoderm microtubule associated protein like 1 [Source:HGNC Symbol;Acc:HGNC:3330]
**-0,632620212**	1,36E-27	3,02E-26	*HLA-B*	major histocompatibility complex, class I, B [Source:HGNC Symbol;Acc:HGNC:4932]
**-0,632359327**	0,002953938	0,010083399	*CD3EAP*	CD3e molecule associated protein [Source:HGNC Symbol;Acc:HGNC:24219]
**-0,631990495**	9,69E-45	3,75E-43	*ADAM9*	ADAM metallopeptidase domain 9 [Source:HGNC Symbol;Acc:HGNC:216]
**-0,631915534**	2,65E-21	4,53E-20	*ABCE1*	ATP binding cassette subfamily E member 1 [Source:HGNC Symbol;Acc:HGNC:69]
**-0,63184592**	1,62E-27	3,58E-26	*WARS*	tryptophanyl-tRNA synthetase [Source:HGNC Symbol;Acc:HGNC:12729]
**-0,631803481**	5,05E-15	6,21E-14	*RBPMS*	RNA binding protein, mRNA processing factor [Source:HGNC Symbol;Acc:HGNC:19097]
**-0,631352841**	4,51E-30	1,11E-28	*IPO5*	importin 5 [Source:HGNC Symbol;Acc:HGNC:6402]
**-0,630727633**	1,71E-26	3,65E-25	*FARP1*	FERM, ARH/RhoGEF and pleckstrin domain protein 1 [Source:HGNC Symbol;Acc:HGNC:3591]
**-0,630601938**	7,11E-07	4,40E-06	*RRP15*	ribosomal RNA processing 15 homolog [Source:HGNC Symbol;Acc:HGNC:24255]
**-0,629590705**	3,38E-16	4,43E-15	*LITAF*	lipopolysaccharide induced TNF factor [Source:HGNC Symbol;Acc:HGNC:16841]
**-0,629389486**	0,001654023	0,005963949	*CEP135*	centrosomal protein 135 [Source:HGNC Symbol;Acc:HGNC:29086]
**-0,629290761**	1,20E-07	8,09E-07	*CTDSPL*	CTD small phosphatase like [Source:HGNC Symbol;Acc:HGNC:16890]
**-0,628859432**	0,003062	0,010408332	*GABRA3*	gamma-aminobutyric acid type A receptor alpha3 subunit [Source:HGNC Symbol;Acc:HGNC:4077]
**-0,628200465**	3,50E-17	4,89E-16	*XPO5*	exportin 5 [Source:HGNC Symbol;Acc:HGNC:17675]
**-0,628145029**	9,30E-23	1,69E-21	*NOP56*	NOP56 ribonucleoprotein [Source:HGNC Symbol;Acc:HGNC:15911]
**-0,628079131**	1,20E-11	1,16E-10	*DSG2*	desmoglein 2 [Source:HGNC Symbol;Acc:HGNC:3049]
**-0,627659674**	2,29E-15	2,87E-14	*DDX39A*	DExD-box helicase 39A [Source:HGNC Symbol;Acc:HGNC:17821]
**-0,626845002**	3,40E-20	5,51E-19	*IDH2*	isocitrate dehydrogenase (NADP(+)) 2, mitochondrial [Source:HGNC Symbol;Acc:HGNC:5383]
**-0,626272614**	0,005720928	0,01819707	*TPD52*	tumor protein D52 [Source:HGNC Symbol;Acc:HGNC:12005]
**-0,625794021**	8,20E-13	8,81E-12	*LPCAT2*	lysophosphatidylcholine acyltransferase 2 [Source:HGNC Symbol;Acc:HGNC:26032]
**-0,625348806**	5,66E-16	7,32E-15	*ABL2*	ABL proto-oncogene 2, non-receptor tyrosine kinase [Source:HGNC Symbol;Acc:HGNC:77]
**-0,624724943**	1,75E-05	8,98E-05	*PLGRKT*	plasminogen receptor with a C-terminal lysine [Source:HGNC Symbol;Acc:HGNC:23633]
**-0,624498843**	2,57E-10	2,22E-09	*CCDC86*	coiled-coil domain containing 86 [Source:HGNC Symbol;Acc:HGNC:28359]
**-0,62433263**	0,000206288	0,000887041	*SLC19A2*	solute carrier family 19 member 2 [Source:HGNC Symbol;Acc:HGNC:10938]
**-0,623344378**	9,34E-11	8,35E-10	*ZMYND19*	zinc finger MYND-type containing 19 [Source:HGNC Symbol;Acc:HGNC:21146]
**-0,623146645**	4,58E-24	8,85E-23	*RHEB*	Ras homolog, mTORC1 binding [Source:HGNC Symbol;Acc:HGNC:10011]

**-0,623011252**	2,41E-14	2,85E-13	*SERTAD2*	SERTA domain containing 2 [Source:HGNC Symbol;Acc:HGNC:30784]
**-0,62300974**	5,98E-20	9,64E-19	*FAM114A1*	family with sequence similarity 114 member A1 [Source:HGNC Symbol;Acc:HGNC:25087]
**-0,62284916**	5,00E-40	1,68E-38	*KIF1C*	kinesin family member 1C [Source:HGNC Symbol;Acc:HGNC:6317]
**-0,622515414**	1,47E-14	1,76E-13	*SLC25A4*	solute carrier family 25 member 4 [Source:HGNC Symbol;Acc:HGNC:10990]
**-0,622485362**	4,74E-33	1,30E-31	*XPO6*	exportin 6 [Source:HGNC Symbol;Acc:HGNC:19733]
**-0,622411004**	3,97E-05	0,00019452	*PTGER4*	prostaglandin E receptor 4 [Source:HGNC Symbol;Acc:HGNC:9596]
**-0,622387584**	1,05E-26	2,25E-25	*MAT2A*	methionine adenosyltransferase 2A [Source:HGNC Symbol;Acc:HGNC:6904]
**-0,622141971**	0,000448925	0,001817996	*RELT*	RELT, TNF receptor [Source:HGNC Symbol;Acc:HGNC:13764]
**-0,622122599**	0,002756786	0,009462693	*CYP2R1*	cytochrome P450 family 2 subfamily R member 1 [Source:HGNC Symbol;Acc:HGNC:20580]
**-0,622062468**	2,92E-11	2,74E-10	*SIPA1L2*	signal induced proliferation associated 1 like 2 [Source:HGNC Symbol;Acc:HGNC:23800]
**-0,621220616**	0,005458488	0,017458546	*GABRG3*	gamma-aminobutyric acid type A receptor gamma3 subunit [Source:HGNC Symbol;Acc:HGNC:4088]
**-0,62120994**	2,53E-11	2,39E-10	*FARP2*	FERM, ARH/RhoGEF and pleckstrin domain protein 2 [Source:HGNC Symbol;Acc:HGNC:16460]
**-0,620857604**	1,50E-05	7,78E-05	*TTC13*	tetratricopeptide repeat domain 13 [Source:HGNC Symbol;Acc:HGNC:26204]
**-0,620578303**	0,000115109	0,000521429	*DSN1*	DSN1 homolog, MIS12 kinetochore complex component [Source:HGNC Symbol;Acc:HGNC:16165]
**-0,620304323**	1,07E-24	2,12E-23	*CTXN1*	cortexin 1 [Source:HGNC Symbol;Acc:HGNC:31108]
**-0,619768571**	3,28E-15	4,07E-14	*WDR5*	WD repeat domain 5 [Source:HGNC Symbol;Acc:HGNC:12757]
**-0,619221174**	3,49E-34	9,90E-33	*GJA1*	gap junction protein alpha 1 [Source:HGNC Symbol;Acc:HGNC:4274]
**-0,618756682**	1,78E-08	1,31E-07	*DNAJC9*	DnaJ heat shock protein family (Hsp40) member C9 [Source:HGNC Symbol;Acc:HGNC:19123]
**-0,618701243**	8,47E-11	7,61E-10	*JADE1*	jade family PHD finger 1 [Source:HGNC Symbol;Acc:HGNC:30027]
**-0,618296958**	2,27E-12	2,35E-11	*KLHL42*	kelch like family member 42 [Source:HGNC Symbol;Acc:HGNC:29252]
**-0,617629472**	1,60E-11	1,53E-10	*SYTL2*	synaptotagmin like 2 [Source:HGNC Symbol;Acc:HGNC:15585]
**-0,617430964**	3,12E-11	2,92E-10	*GRK6*	G protein-coupled receptor kinase 6 [Source:HGNC Symbol;Acc:HGNC:4545]
**-0,617371227**	8,31E-05	0,000385764	*RXYLT1*	ribitol xylosyltransferase 1 [Source:HGNC Symbol;Acc:HGNC:13530]
**-0,617326983**	8,99E-06	4,82E-05	*PREX2*	phosphatidylinositol-3,4,5-trisphosphate dependent Rac exchange factor 2 [Source:HGNC Symbol;Acc:HGNC:22950]
**-0,616774168**	2,23E-22	3,97E-21	*PGRMC2*	progesterone receptor membrane component 2 [Source:HGNC Symbol;Acc:HGNC:16089]
**-0,616261144**	2,12E-20	3,48E-19	*BCAR3*	BCAR3, NSP family adaptor protein [Source:HGNC Symbol;Acc:HGNC:973]
**-0,61562873**	1,21E-22	2,18E-21	*KCTD10*	potassium channel tetramerization domain containing 10 [Source:HGNC Symbol;Acc:HGNC:23236]
**-0,615276052**	0,000131273	0,00058874	*MSLN*	mesothelin [Source:HGNC Symbol;Acc:HGNC:7371]
**-0,615201604**	2,76E-25	5,60E-24	*BCAM*	basal cell adhesion molecule (Lutheran blood group) [Source:HGNC Symbol;Acc:HGNC:6722]
**-0,614378643**	0,00055635	0,002212405	*SIMC1*	SUMO interacting motifs containing 1 [Source:HGNC Symbol;Acc:HGNC:24779]
**-0,614366392**	2,74E-07	1,78E-06	*DDX11*	DEAD/H-box helicase 11 [Source:HGNC Symbol;Acc:HGNC:2736]
**-0,614204618**	0,001308319	0,004825934	*FEM1A*	fem-1 homolog A [Source:HGNC Symbol;Acc:HGNC:16934]

**-0,614099779**	6,24E-09	4,79E-08	*SGTB*	small glutamine rich tetratricopeptide repeat containing beta [Source:HGNC Symbol;Acc:HGNC:23567]
**-0,61308279**	0,000122839	0,000553731	*SERTAD4*	SERTA domain containing 4 [Source:HGNC Symbol;Acc:HGNC:25236]
**-0,612980093**	3,81E-12	3,85E-11	*DCBLD1*	discoidin, CUB and LCCL domain containing 1 [Source:HGNC Symbol;Acc:HGNC:21479]
**-0,612814755**	2,46E-05	0,000124304	*SLC25A28*	solute carrier family 25 member 28 [Source:HGNC Symbol;Acc:HGNC:23472]
**-0,612522056**	2,62E-15	3,28E-14	*ERCC2*	ERCC excision repair 2, TFIIH core complex helicase subunit [Source:HGNC Symbol;Acc:HGNC:3434]
**-0,612098973**	0,00152367	0,005543369	*POLR3K*	RNA polymerase III subunit K [Source:HGNC Symbol;Acc:HGNC:14121]
**-0,61208703**	2,17E-29	5,20E-28	*LARS*	leucyl-tRNA synthetase [Source:HGNC Symbol;Acc:HGNC:6512]
**-0,611932007**	1,07E-08	8,02E-08	*KIF22*	kinesin family member 22 [Source:HGNC Symbol;Acc:HGNC:6391]
**-0,611381008**	1,19E-05	6,24E-05	*CXCL1*	C-X-C motif chemokine ligand 1 [Source:HGNC Symbol;Acc:HGNC:4602]
**-0,611338905**	3,74E-06	2,11E-05	*AHNAK*	AHNAK nucleoprotein [Source:HGNC Symbol;Acc:HGNC:347]
**-0,610747991**	2,07E-08	1,51E-07	*C2CD3*	C2 calcium dependent domain containing 3 [Source:HGNC Symbol;Acc:HGNC:24564]
**-0,610253841**	3,80E-08	2,71E-07	*NUDCD1*	NudC domain containing 1 [Source:HGNC Symbol;Acc:HGNC:24306]
**-0,609832255**	5,99E-24	1,15E-22	*WSB2*	WD repeat and SOCS box containing 2 [Source:HGNC Symbol;Acc:HGNC:19222]
**-0,609686216**	1,11E-07	7,52E-07	*PAK1IP1*	PAK1 interacting protein 1 [Source:HGNC Symbol;Acc:HGNC:20882]
**-0,609659981**	1,22E-30	3,06E-29	*ARHGEF17*	Rho guanine nucleotide exchange factor 17 [Source:HGNC Symbol;Acc:HGNC:21726]
**-0,609651921**	1,20E-07	8,12E-07	*RABGGTB*	Rab geranylgeranyltransferase subunit beta [Source:HGNC Symbol;Acc:HGNC:9796]
**-0,609360094**	2,40E-27	5,26E-26	*ITGAV*	integrin subunit alpha V [Source:HGNC Symbol;Acc:HGNC:6150]
**-0,609333412**	2,33E-06	1,35E-05	*PPP1R12B*	protein phosphatase 1 regulatory subunit 12B [Source:HGNC Symbol;Acc:HGNC:7619]
**-0,609253319**	1,46E-23	2,72E-22	*CCDC85C*	coiled-coil domain containing 85C [Source:HGNC Symbol;Acc:HGNC:35459]
**-0,609000397**	1,62E-11	1,55E-10	*CHORDC1*	cysteine and histidine rich domain containing 1 [Source:HGNC Symbol;Acc:HGNC:14525]
**-0,608952529**	7,10E-09	5,42E-08	*KCNG1*	potassium voltage-gated channel modifier subfamily G member 1 [Source:HGNC Symbol;Acc:HGNC:6248]
**-0,608745327**	4,01E-06	2,25E-05	*PUS1*	pseudouridylate synthase 1 [Source:HGNC Symbol;Acc:HGNC:15508]
**-0,608651632**	4,67E-12	4,68E-11	*CBLB*	Cbl proto-oncogene B [Source:HGNC Symbol;Acc:HGNC:1542]
**-0,607788456**	3,12E-15	3,88E-14	*FADS3*	fatty acid desaturase 3 [Source:HGNC Symbol;Acc:HGNC:3576]
**-0,60713861**	1,54E-42	5,61E-41	*ACTR3*	ARP3 actin related protein 3 homolog [Source:HGNC Symbol;Acc:HGNC:170]
**-0,607111739**	2,29E-05	0,000115764	*LYAR*	Ly1 antibody reactive [Source:HGNC Symbol;Acc:HGNC:26021]
**-0,606864977**	3,61E-24	7,01E-23	*SLC16A1*	solute carrier family 16 member 1 [Source:HGNC Symbol;Acc:HGNC:10922]
**-0,606811983**	3,02E-06	1,72E-05	*AFF2*	AF4/FMR2 family member 2 [Source:HGNC Symbol;Acc:HGNC:3776]
**-0,606796125**	5,11E-13	5,59E-12	*CAP2*	cyclase associated actin cytoskeleton regulatory protein 2 [Source:HGNC Symbol;Acc:HGNC:20039]
**-0,606568205**	3,84E-22	6,77E-21	*ANKLE2*	ankyrin repeat and LEM domain containing 2 [Source:HGNC Symbol;Acc:HGNC:29101]
**-0,606274311**	1,78E-13	2,00E-12	*EBNA1BP2*	EBNA1 binding protein 2 [Source:HGNC Symbol;Acc:HGNC:15531]

**-0,604993989**	4,32E-05	0,000210318	*G2E3*	G2/M-phase specific E3 ubiquitin protein ligase [Source:HGNC Symbol;Acc:HGNC:20338]
**-0,604798532**	1,38E-09	1,12E-08	*AK4*	adenylate kinase 4 [Source:HGNC Symbol;Acc:HGNC:363]
**-0,604249796**	3,10E-16	4,07E-15	*LRRFIP2*	LRR binding FLII interacting protein 2 [Source:HGNC Symbol;Acc:HGNC:6703]
**-0,60403196**	1,38E-12	1,45E-11	*CNTNAP1*	contactin associated protein 1 [Source:HGNC Symbol;Acc:HGNC:8011]
**-0,603836049**	2,47E-20	4,05E-19	*RHOJ*	ras homolog family member J [Source:HGNC Symbol;Acc:HGNC:688]
**-0,603802226**	3,06E-07	1,98E-06	*CKS1B*	CDC28 protein kinase regulatory subunit 1B [Source:HGNC Symbol;Acc:HGNC:19083]
**-0,603636114**	8,65E-17	1,18E-15	*RUVBL1*	RuvB like AAA ATPase 1 [Source:HGNC Symbol;Acc:HGNC:10474]
**-0,602909309**	1,44E-09	1,17E-08	*PDLIM2*	PDZ and LIM domain 2 [Source:HGNC Symbol;Acc:HGNC:13992]
**-0,602790964**	5,72E-21	9,67E-20	*FNDC3B*	fibronectin type III domain containing 3B [Source:HGNC Symbol;Acc:HGNC:24670]
**-0,601996909**	9,50E-47	3,93E-45	*CALM2*	calmodulin 2 [Source:HGNC Symbol;Acc:HGNC:1445]
**-0,60177638**	2,96E-23	5,47E-22	*CLIP1*	CAP-Gly domain containing linker protein 1 [Source:HGNC Symbol;Acc:HGNC:10461]
**-0,601424447**	9,53E-09	7,20E-08	*PNPT1*	polyribonucleotide nucleotidyltransferase 1 [Source:HGNC Symbol;Acc:HGNC:23166]
**-0,601308519**	5,03E-16	6,52E-15	*MOXD1*	monooxygenase DBH like 1 [Source:HGNC Symbol;Acc:HGNC:21063]
**-0,600560818**	0,00011768	0,000531958	*AOX1*	aldehyde oxidase 1 [Source:HGNC Symbol;Acc:HGNC:553]
**-0,600285678**	8,72E-16	1,12E-14	*FZD1*	frizzled class receptor 1 [Source:HGNC Symbol;Acc:HGNC:4038]
**-0,599918444**	8,17E-08	5,63E-07	*DERA*	deoxyribose-phosphate aldolase [Source:HGNC Symbol;Acc:HGNC:24269]
**-0,599502999**	0,000531664	0,002122498	*PDE5A*	phosphodiesterase 5A [Source:HGNC Symbol;Acc:HGNC:8784]
**-0,599459159**	6,32E-13	6,85E-12	*NLN*	neurolysin [Source:HGNC Symbol;Acc:HGNC:16058]
**-0,599382182**	1,04E-13	1,18E-12	*SNTB2*	syntrophin beta 2 [Source:HGNC Symbol;Acc:HGNC:11169]
**-0,5990893**	3,41E-06	1,93E-05	*FIGN*	fidgetin, microtubule severing factor [Source:HGNC Symbol;Acc:HGNC:13285]
**-0,598608614**	0,000112545	0,000510361	*C12orf29*	chromosome 12 open reading frame 29 [Source:HGNC Symbol;Acc:HGNC:25322]
**-0,597416629**	1,73E-07	1,15E-06	*ITGA1*	integrin subunit alpha 1 [Source:HGNC Symbol;Acc:HGNC:6134]
**-0,597298983**	5,58E-35	1,63E-33	*CDC42EP3*	CDC42 effector protein 3 [Source:HGNC Symbol;Acc:HGNC:16943]
**-0,59719828**	0,000116775	0,000528113	*GMNN*	geminin, DNA replication inhibitor [Source:HGNC Symbol;Acc:HGNC:17493]
**-0,597117578**	0,000121577	0,000548551	*GAL3ST3*	galactose-3-O-sulfotransferase 3 [Source:HGNC Symbol;Acc:HGNC:24144]
**-0,597055481**	9,30E-32	2,44E-30	*PARVA*	parvin alpha [Source:HGNC Symbol;Acc:HGNC:14652]
**-0,596898555**	2,37E-06	1,37E-05	*SIAH2*	siah E3 ubiquitin protein ligase 2 [Source:HGNC Symbol;Acc:HGNC:10858]
**-0,596851546**	1,93E-11	1,83E-10	*CLSTN2*	calsyntenin 2 [Source:HGNC Symbol;Acc:HGNC:17448]
**-0,596794988**	7,63E-11	6,87E-10	*DIMT1*	DIM1 dimethyladenosine transferase 1 homolog [Source:HGNC Symbol;Acc:HGNC:30217]
**-0,596449349**	3,78E-14	4,44E-13	*CD2AP*	CD2 associated protein [Source:HGNC Symbol;Acc:HGNC:14258]
**-0,596371011**	1,84E-09	1,48E-08	*IMPDH1*	inosine monophosphate dehydrogenase 1 [Source:HGNC Symbol;Acc:HGNC:6052]
**-0,596217715**	5,68E-11	5,17E-10	*NFKB2*	nuclear factor kappa B subunit 2 [Source:HGNC Symbol;Acc:HGNC:7795]
**-0,59584319**	1,63E-06	9,67E-06	*EXOSC9*	exosome component 9 [Source:HGNC Symbol;Acc:HGNC:9137]

**-0,594507765**	2,35E-07	1,54E-06	*GPR180*	G protein-coupled receptor 180 [Source:HGNC Symbol;Acc:HGNC:28899]
**-0,594330291**	2,13E-09	1,70E-08	*AHR*	aryl hydrocarbon receptor [Source:HGNC Symbol;Acc:HGNC:348]
**-0,594096861**	1,74E-45	6,87E-44	*RAN*	RAN, member RAS oncogene family [Source:HGNC Symbol;Acc:HGNC:9846]
**-0,593821048**	9,45E-05	0,000434986	*NLRC5*	NLR family CARD domain containing 5 [Source:HGNC Symbol;Acc:HGNC:29933]
**-0,593202292**	5,56E-11	5,07E-10	*PAPPA*	pappalysin 1 [Source:HGNC Symbol;Acc:HGNC:8602]
**-0,593115652**	1,18E-09	9,64E-09	*DSE*	dermatan sulfate epimerase [Source:HGNC Symbol;Acc:HGNC:21144]
**-0,592773664**	0,000572154	0,002271692	*IQCB1*	IQ motif containing B1 [Source:HGNC Symbol;Acc:HGNC:28949]
**-0,592760531**	0,007131347	0,022099428	*EIF4A1*	eukaryotic translation initiation factor 4A1 [Source:HGNC Symbol;Acc:HGNC:3282]
**-0,592411116**	0,000827784	0,003195538	*SRFBP1*	serum response factor binding protein 1 [Source:HGNC Symbol;Acc:HGNC:26333]
**-0,591969519**	3,86E-18	5,70E-17	*MEX3D*	mex-3 RNA binding family member D [Source:HGNC Symbol;Acc:HGNC:16734]
**-0,591910734**	3,29E-16	4,32E-15	*IGFBP7*	insulin like growth factor binding protein 7 [Source:HGNC Symbol;Acc:HGNC:5476]
**-0,590464628**	2,66E-06	1,53E-05	*HEATR3*	HEAT repeat containing 3 [Source:HGNC Symbol;Acc:HGNC:26087]
**-0,59002912**	0,000199484	0,000861221	*STS*	steroid sulfatase [Source:HGNC Symbol;Acc:HGNC:11425]
**-0,589927055**	8,10E-05	0,000376889	*MCM8*	minichromosome maintenance 8 homologous recombination repair factor [Source:HGNC Symbol;Acc:HGNC:16147]
**-0,58959448**	0,001859915	0,00662996	*MTERF3*	mitochondrial transcription termination factor 3 [Source:HGNC Symbol;Acc:HGNC:24258]
**-0,589112536**	0,000937781	0,003571851	*SLC25A15*	solute carrier family 25 member 15 [Source:HGNC Symbol;Acc:HGNC:10985]
**-0,588596632**	7,62E-24	1,45E-22	*HDAC7*	histone deacetylase 7 [Source:HGNC Symbol;Acc:HGNC:14067]
**-0,588221597**	0,000123533	0,000556731	*LARP1B*	La ribonucleoprotein domain family member 1B [Source:HGNC Symbol;Acc:HGNC:24704]
**-0,587041485**	0,002070998	0,007320543	*SNAI1*	snail family transcriptional repressor 1 [Source:HGNC Symbol;Acc:HGNC:11128]
**-0,587008996**	3,77E-05	0,00018537	*SPRTN*	SprT-like N-terminal domain [Source:HGNC Symbol;Acc:HGNC:25356]
**-0,586924298**	3,92E-06	2,20E-05	*TNC*	tenascin C [Source:HGNC Symbol;Acc:HGNC:5318]
**-0,586672686**	0,000493079	0,001981916	*MAGOHB*	mago homolog B, exon junction complex subunit [Source:HGNC Symbol;Acc:HGNC:25504]
**-0,586331762**	0,000107903	0,000490506	*C16orf87*	chromosome 16 open reading frame 87 [Source:HGNC Symbol;Acc:HGNC:33754]
**-0,58611932**	0,000280813	0,001178262	*METTL1*	methyltransferase like 1 [Source:HGNC Symbol;Acc:HGNC:7030]
**-0,585695769**	1,57E-12	1,65E-11	*BZW2*	basic leucine zipper and W2 domains 2 [Source:HGNC Symbol;Acc:HGNC:18808]
**-0,585663085**	2,25E-05	0,000114271	*PRPS2*	phosphoribosyl pyrophosphate synthetase 2 [Source:HGNC Symbol;Acc:HGNC:9465]
**-0,585228322**	2,41E-14	2,84E-13	*WDR43*	WD repeat domain 43 [Source:HGNC Symbol;Acc:HGNC:28945]
**-0,585162241**	5,93E-09	4,56E-08	*NCAPH2*	non-SMC condensin II complex subunit H2 [Source:HGNC Symbol;Acc:HGNC:25071]
**-0,584927463**	2,13E-12	2,21E-11	*RRP1B*	ribosomal RNA processing 1B [Source:HGNC Symbol;Acc:HGNC:23818]
**-0,584024726**	1,06E-18	1,61E-17	*GPT2*	glutamic--pyruvic transaminase 2 [Source:HGNC Symbol;Acc:HGNC:18062]
**-0,583861853**	2,95E-22	5,22E-21	*RUSC2*	RUN and SH3 domain containing 2 [Source:HGNC Symbol;Acc:HGNC:23625]

**-0,583664656**	7,28E-08	5,04E-07	*SLIT2*	slit guidance ligand 2 [Source:HGNC Symbol;Acc:HGNC:11086]
**-0,582761239**	2,93E-08	2,11E-07	*EPHA5*	EPH receptor A5 [Source:HGNC Symbol;Acc:HGNC:3389]
**-0,582748761**	5,78E-10	4,86E-09	*SMIM13*	small integral membrane protein 13 [Source:HGNC Symbol;Acc:HGNC:27356]
**-0,582472637**	0,001153276	0,004305593	*TICRR*	TOPBP1 interacting checkpoint and replication regulator [Source:HGNC Symbol;Acc:HGNC:28704]
**-0,582228657**	4,66E-05	0,00022558	*COPZ2*	coatomer protein complex subunit zeta 2 [Source:HGNC Symbol;Acc:HGNC:19356]
**-0,582220759**	0,008234103	0,025069189	*CLDN2*	claudin 2 [Source:HGNC Symbol;Acc:HGNC:2041]
**-0,582117809**	1,60E-25	3,30E-24	*NCLN*	nicalin [Source:HGNC Symbol;Acc:HGNC:26923]
**-0,581810358**	0,002717954	0,00935188	*SPAG1*	sperm associated antigen 1 [Source:HGNC Symbol;Acc:HGNC:11212]
**-0,581226318**	9,44E-18	1,37E-16	*GPX8*	glutathione peroxidase 8 (putative) [Source:HGNC Symbol;Acc:HGNC:33100]
**-0,581177388**	3,27E-14	3,85E-13	*BGN*	biglycan [Source:HGNC Symbol;Acc:HGNC:1044]
**-0,580926522**	0,003333445	0,011224853	*SUV39H1*	suppressor of variegation 3-9 homolog 1 [Source:HGNC Symbol;Acc:HGNC:11479]
**-0,580198686**	2,22E-08	1,62E-07	*ARL6IP6*	ADP ribosylation factor like GTPase 6 interacting protein 6 [Source:HGNC Symbol;Acc:HGNC:24048]
**-0,579743977**	6,96E-12	6,84E-11	*USP1*	ubiquitin specific peptidase 1 [Source:HGNC Symbol;Acc:HGNC:12607]
**-0,578859599**	1,40E-05	7,27E-05	*C9orf64*	chromosome 9 open reading frame 64 [Source:HGNC Symbol;Acc:HGNC:28144]
**-0,578527624**	0,01175139	0,034220808	*BRICD5*	BRICHOS domain containing 5 [Source:HGNC Symbol;Acc:HGNC:28309]
**-0,578095315**	4,24E-06	2,37E-05	*CPNE7*	copine 7 [Source:HGNC Symbol;Acc:HGNC:2320]
**-0,577961875**	9,16E-26	1,90E-24	*AZIN1*	antizyme inhibitor 1 [Source:HGNC Symbol;Acc:HGNC:16432]
**-0,577058291**	1,82E-15	2,29E-14	*TCOF1*	treacle ribosome biogenesis factor 1 [Source:HGNC Symbol;Acc:HGNC:11654]
**-0,576064786**	3,92E-17	5,45E-16	*VAMP5*	vesicle associated membrane protein 5 [Source:HGNC Symbol;Acc:HGNC:12646]
**-0,575730303**	3,95E-32	1,05E-30	*EFEMP2*	EGF containing fibulin extracellular matrix protein 2 [Source:HGNC Symbol;Acc:HGNC:3219]
**-0,575056319**	3,73E-11	3,48E-10	*PDCD5*	programmed cell death 5 [Source:HGNC Symbol;Acc:HGNC:8764]
**-0,574937537**	8,58E-31	2,17E-29	*ANKRD52*	ankyrin repeat domain 52 [Source:HGNC Symbol;Acc:HGNC:26614]
**-0,574771411**	4,42E-16	5,75E-15	*MID1*	midline 1 [Source:HGNC Symbol;Acc:HGNC:7095]
**-0,57446578**	1,26E-08	9,38E-08	*CHML*	CHM like, Rab escort protein 2 [Source:HGNC Symbol;Acc:HGNC:1941]
**-0,574290652**	8,04E-13	8,65E-12	*RPS6KA4*	ribosomal protein S6 kinase A4 [Source:HGNC Symbol;Acc:HGNC:10433]
**-0,574248472**	6,70E-09	5,13E-08	*UMPS*	uridine monophosphate synthetase [Source:HGNC Symbol;Acc:HGNC:12563]
**-0,574220223**	1,11E-08	8,36E-08	*MFHAS1*	malignant fibrous histiocytoma amplified sequence 1 [Source:HGNC Symbol;Acc:HGNC:16982]
**-0,57334022**	3,11E-06	1,77E-05	*AVEN*	apoptosis and caspase activation inhibitor [Source:HGNC Symbol;Acc:HGNC:13509]
**-0,572665639**	1,67E-06	9,90E-06	*DDX31*	DEAD-box helicase 31 [Source:HGNC Symbol;Acc:HGNC:16715]
**-0,572637385**	8,91E-48	3,78E-46	*RTN4*	reticulon 4 [Source:HGNC Symbol;Acc:HGNC:14085]
**-0,57226095**	2,60E-05	0,000130895	*GSTCD*	glutathione S-transferase C-terminal domain containing [Source:HGNC Symbol;Acc:HGNC:25806]
**-0,571943537**	4,21E-09	3,29E-08	*DAAM1*	dishevelled associated activator of morphogenesis 1 [Source:HGNC Symbol;Acc:HGNC:18142]
**-0,571923486**	1,29E-14	1,55E-13	*ELMSAN1*	ELM2 and Myb/SANT domain containing 1 [Source:HGNC Symbol;Acc:HGNC:19853]
**-0,571320088**	0,015803403	0,044225842	*ZNF595*	zinc finger protein 595 [Source:HGNC Symbol;Acc:HGNC:27196]

**-0,570911882**	1,22E-08	9,12E-08	*RNASEH1*	ribonuclease H1 [Source:HGNC Symbol;Acc:HGNC:18466]
**-0,570021849**	8,17E-14	9,37E-13	*CHST10*	carbohydrate sulfotransferase 10 [Source:HGNC Symbol;Acc:HGNC:19650]
**-0,569858093**	1,93E-23	3,58E-22	*ALDH18A1*	aldehyde dehydrogenase 18 family member A1 [Source:HGNC Symbol;Acc:HGNC:9722]
**-0,569824486**	1,18E-06	7,14E-06	*KRBA1*	KRAB-A domain containing 1 [Source:HGNC Symbol;Acc:HGNC:22228]
**-0,569308852**	1,71E-09	1,38E-08	*RHPN2*	rhophilin Rho GTPase binding protein 2 [Source:HGNC Symbol;Acc:HGNC:19974]
**-0,569292961**	4,90E-14	5,69E-13	*PHACTR4*	phosphatase and actin regulator 4 [Source:HGNC Symbol;Acc:HGNC:25793]
**-0,568692365**	5,31E-28	1,20E-26	*PPP1R18*	protein phosphatase 1 regulatory subunit 18 [Source:HGNC Symbol;Acc:HGNC:29413]
**-0,568489546**	1,37E-12	1,44E-11	*OGFOD1*	2-oxoglutarate and iron dependent oxygenase domain containing 1 [Source:HGNC Symbol;Acc:HGNC:25585]
**-0,568380101**	1,12E-17	1,62E-16	*ROCK2*	Rho associated coiled-coil containing protein kinase 2 [Source:HGNC Symbol;Acc:HGNC:10252]
**-0,568045666**	9,66E-10	7,95E-09	*MDC1*	mediator of DNA damage checkpoint 1 [Source:HGNC Symbol;Acc:HGNC:21163]
**-0,567146225**	9,68E-12	9,42E-11	*NDEL1*	nudE neurodevelopment protein 1 like 1 [Source:HGNC Symbol;Acc:HGNC:17620]
**-0,566859815**	3,94E-19	6,13E-18	*THOP1*	thimet oligopeptidase 1 [Source:HGNC Symbol;Acc:HGNC:11793]
**-0,566580567**	9,43E-15	1,14E-13	*HDAC9*	histone deacetylase 9 [Source:HGNC Symbol;Acc:HGNC:14065]
**-0,565470202**	6,12E-07	3,81E-06	*EZH2*	enhancer of zeste 2 polycomb repressive complex 2 subunit [Source:HGNC Symbol;Acc:HGNC:3527]
**-0,565461424**	8,32E-08	5,72E-07	*NME1*	NME/NM23 nucleoside diphosphate kinase 1 [Source:HGNC Symbol;Acc:HGNC:7849]
**-0,565298098**	3,51E-19	5,48E-18	*NCAM1*	neural cell adhesion molecule 1 [Source:HGNC Symbol;Acc:HGNC:7656]
**-0,565202286**	0,000267052	0,001125143	*RFC5*	replication factor C subunit 5 [Source:HGNC Symbol;Acc:HGNC:9973]
**-0,564637582**	8,50E-16	1,09E-14	*MRTFA*	myocardin related transcription factor A [Source:HGNC Symbol;Acc:HGNC:14334]
**-0,563964663**	0,002351142	0,008209115	*SNCAIP*	synuclein alpha interacting protein [Source:HGNC Symbol;Acc:HGNC:11139]
**-0,56382806**	1,80E-22	3,22E-21	*LRRFIP1*	LRR binding FLII interacting protein 1 [Source:HGNC Symbol;Acc:HGNC:6702]
**-0,563759263**	3,69E-06	2,08E-05	*TNFRSF19*	TNF receptor superfamily member 19 [Source:HGNC Symbol;Acc:HGNC:11915]
**-0,563696459**	4,19E-10	3,56E-09	*RAC3*	Rac family small GTPase 3 [Source:HGNC Symbol;Acc:HGNC:9803]
**-0,5635394**	2,89E-06	1,66E-05	*NPAS2*	neuronal PAS domain protein 2 [Source:HGNC Symbol;Acc:HGNC:7895]
**-0,563465547**	7,84E-23	1,43E-21	*ST5*	suppression of tumorigenicity 5 [Source:HGNC Symbol;Acc:HGNC:11350]
**-0,562927374**	8,55E-46	3,41E-44	*EIF5A*	eukaryotic translation initiation factor 5A [Source:HGNC Symbol;Acc:HGNC:3300]
**-0,562828465**	0,000499764	0,002005463	*SAPCD2*	suppressor APC domain containing 2 [Source:HGNC Symbol;Acc:HGNC:28055]
**-0,562793177**	1,56E-25	3,22E-24	*OPTN*	optineurin [Source:HGNC Symbol;Acc:HGNC:17142]
**-0,562516201**	4,29E-05	0,000209026	*PMM2*	phosphomannomutase 2 [Source:HGNC Symbol;Acc:HGNC:9115]
**-0,562512326**	7,12E-13	7,69E-12	*ADAMTS15*	ADAM metallopeptidase with thrombospondin type 1 motif 15 [Source:HGNC Symbol;Acc:HGNC:16305]
**-0,562490422**	5,96E-33	1,63E-31	*MXRA7*	matrix remodeling associated 7 [Source:HGNC Symbol;Acc:HGNC:7541]
**-0,562432403**	0,000167694	0,000735596	*DUSP7*	dual specificity phosphatase 7 [Source:HGNC Symbol;Acc:HGNC:3073]

**-0,562018197**	3,25E-12	3,32E-11	*NFKB1*	nuclear factor kappa B subunit 1 [Source:HGNC Symbol;Acc:HGNC:7794]
**-0,561852201**	2,76E-07	1,79E-06	*MPV17L2*	MPV17 mitochondrial inner membrane protein like 2 [Source:HGNC Symbol;Acc:HGNC:28177]
**-0,561319475**	4,13E-08	2,93E-07	*TPST1*	tyrosylprotein sulfotransferase 1 [Source:HGNC Symbol;Acc:HGNC:12020]
**-0,561244238**	1,83E-07	1,21E-06	*POP1*	POP1 homolog, ribonuclease P/MRP subunit [Source:HGNC Symbol;Acc:HGNC:30129]
**-0,561111017**	3,47E-14	4,07E-13	*LTBP1*	latent transforming growth factor beta binding protein 1 [Source:HGNC Symbol;Acc:HGNC:6714]
**-0,559960198**	1,41E-15	1,78E-14	*ALYREF*	Aly/REF export factor [Source:HGNC Symbol;Acc:HGNC:19071]
**-0,559878762**	8,21E-10	6,81E-09	*DPH3*	diphthamide biosynthesis 3 [Source:HGNC Symbol;Acc:HGNC:27717]
**-0,559850277**	1,93E-08	1,41E-07	*PRKD1*	protein kinase D1 [Source:HGNC Symbol;Acc:HGNC:9407]
**-0,559636139**	1,34E-07	9,00E-07	*KLF10*	Kruppel like factor 10 [Source:HGNC Symbol;Acc:HGNC:11810]
**-0,559511881**	1,20E-11	1,16E-10	*DHX37*	DEAH-box helicase 37 [Source:HGNC Symbol;Acc:HGNC:17210]
**-0,558962972**	4,03E-08	2,86E-07	*PCSK5*	proprotein convertase subtilisin/kexin type 5 [Source:HGNC Symbol;Acc:HGNC:8747]
**-0,558320949**	0,001792094	0,006411758	*CGN*	cingulin [Source:HGNC Symbol;Acc:HGNC:17429]
**-0,557928753**	2,45E-19	3,84E-18	*IVNS1ABP*	influenza virus NS1A binding protein [Source:HGNC Symbol;Acc:HGNC:16951]
**-0,557203994**	0,000152834	0,000675138	*BX255925,3*	novel RING finger protein
**-0,556865534**	4,59E-17	6,37E-16	*PDCD11*	programmed cell death 11 [Source:HGNC Symbol;Acc:HGNC:13408]
**-0,556612756**	2,25E-26	4,78E-25	*MYOF*	myoferlin [Source:HGNC Symbol;Acc:HGNC:3656]
**-0,5565866**	3,30E-12	3,36E-11	*SETD3*	SET domain containing 3 [Source:HGNC Symbol;Acc:HGNC:20493]
**-0,556366442**	2,43E-13	2,70E-12	*MED22*	mediator complex subunit 22 [Source:HGNC Symbol;Acc:HGNC:11477]
**-0,555335754**	4,60E-11	4,23E-10	*GNL2*	G protein nucleolar 2 [Source:HGNC Symbol;Acc:HGNC:29925]
**-0,555201155**	0,00171967	0,006174267	*DNAJC6*	DnaJ heat shock protein family (Hsp40) member C6 [Source:HGNC Symbol;Acc:HGNC:15469]
**-0,554671238**	1,93E-32	5,21E-31	*LIMA1*	LIM domain and actin binding 1 [Source:HGNC Symbol;Acc:HGNC:24636]
**-0,553679693**	1,08E-18	1,64E-17	*POFUT1*	protein O-fucosyltransferase 1 [Source:HGNC Symbol;Acc:HGNC:14988]
**-0,552980814**	0,000412798	0,0016847	*PIGW*	phosphatidylinositol glycan anchor biosynthesis class W [Source:HGNC Symbol;Acc:HGNC:23213]
**-0,551760803**	3,25E-34	9,25E-33	*ACTR2*	ARP2 actin related protein 2 homolog [Source:HGNC Symbol;Acc:HGNC:169]
**-0,551400049**	4,93E-06	2,73E-05	*UPRT*	uracil phosphoribosyltransferase homolog [Source:HGNC Symbol;Acc:HGNC:28334]
**-0,551130948**	3,88E-13	4,26E-12	*NAA15*	N(alpha)-acetyltransferase 15, NatA auxiliary subunit [Source:HGNC Symbol;Acc:HGNC:30782]
**-0,551029545**	0,001440614	0,005268859	*CENPL*	centromere protein L [Source:HGNC Symbol;Acc:HGNC:17879]
**-0,550784504**	1,57E-09	1,27E-08	*CDH8*	cadherin 8 [Source:HGNC Symbol;Acc:HGNC:1767]
**-0,550594378**	3,55E-07	2,27E-06	*SLC25A24*	solute carrier family 25 member 24 [Source:HGNC Symbol;Acc:HGNC:20662]
**-0,55008321**	4,50E-12	4,52E-11	*IMP4*	IMP4, U3 small nucleolar ribonucleoprotein [Source:HGNC Symbol;Acc:HGNC:30856]
**-0,550052494**	1,64E-09	1,32E-08	*PGP*	phosphoglycolate phosphatase [Source:HGNC Symbol;Acc:HGNC:8909]
**-0,549880943**	6,09E-39	1,99E-37	*KPNB1*	karyopherin subunit beta 1 [Source:HGNC Symbol;Acc:HGNC:6400]
**-0,54925893**	3,43E-07	2,20E-06	*NDOR1*	NADPH dependent diflavin oxidoreductase 1 [Source:HGNC Symbol;Acc:HGNC:29838]

**-0,549056207**	3,93E-07	2,50E-06	*CENPT*	centromere protein T [Source:HGNC Symbol;Acc:HGNC:25787]
**-0,548615126**	1,40E-05	7,27E-05	*CNKSR3*	CNKSR family member 3 [Source:HGNC Symbol;Acc:HGNC:23034]
**-0,54841651**	0,001173818	0,004371352	*TKTL1*	transketolase like 1 [Source:HGNC Symbol;Acc:HGNC:11835]
**-0,54831437**	6,59E-25	1,32E-23	*PPA1*	pyrophosphatase (inorganic) 1 [Source:HGNC Symbol;Acc:HGNC:9226]
**-0,547589673**	2,64E-07	1,71E-06	*NUP85*	nucleoporin 85 [Source:HGNC Symbol;Acc:HGNC:8734]
**-0,547393294**	7,37E-06	4,01E-05	*PARP2*	poly(ADP-ribose) polymerase 2 [Source:HGNC Symbol;Acc:HGNC:272]
**-0,546874812**	0,009629889	0,028728305	*AMER1*	APC membrane recruitment protein 1 [Source:HGNC Symbol;Acc:HGNC:26837]
**-0,546724405**	0,002123389	0,00749345	*TMEM241*	transmembrane protein 241 [Source:HGNC Symbol;Acc:HGNC:31723]
**-0,546523958**	0,003182535	0,010770889	*RNF219*	ring finger protein 219 [Source:HGNC Symbol;Acc:HGNC:20308]
**-0,546198649**	3,22E-15	3,99E-14	*FEZ2*	fasciculation and elongation protein zeta 2 [Source:HGNC Symbol;Acc:HGNC:3660]
**-0,545930472**	0,002467624	0,008581538	*BAIAP2L1*	BAI1 associated protein 2 like 1 [Source:HGNC Symbol;Acc:HGNC:21649]
**-0,545597983**	0,017860644	0,049100957	*ZNF324B*	zinc finger protein 324B [Source:HGNC Symbol;Acc:HGNC:33107]
**-0,545360981**	1,52E-05	7,87E-05	*HABP4*	hyaluronan binding protein 4 [Source:HGNC Symbol;Acc:HGNC:17062]
**-0,545183657**	5,08E-12	5,08E-11	*WDR34*	WD repeat domain 34 [Source:HGNC Symbol;Acc:HGNC:28296]
**-0,544771164**	6,85E-27	1,48E-25	*ADAM19*	ADAM metallopeptidase domain 19 [Source:HGNC Symbol;Acc:HGNC:197]
**-0,544588101**	8,12E-10	6,73E-09	*QSOX2*	quiescin sulfhydryl oxidase 2 [Source:HGNC Symbol;Acc:HGNC:30249]
**-0,544385111**	0,001146371	0,004284761	*GINS4*	GINS complex subunit 4 [Source:HGNC Symbol;Acc:HGNC:28226]
**-0,54432003**	9,15E-06	4,90E-05	*PHLPP2*	PH domain and leucine rich repeat protein phosphatase 2 [Source:HGNC Symbol;Acc:HGNC:29149]
**-0,544140544**	0,00069179	0,002710455	*MXD3*	MAX dimerization protein 3 [Source:HGNC Symbol;Acc:HGNC:14008]
**-0,544128827**	2,05E-08	1,50E-07	*CFH*	complement factor H [Source:HGNC Symbol;Acc:HGNC:4883]
**-0,544058295**	1,15E-18	1,74E-17	*UGP2*	UDP-glucose pyrophosphorylase 2 [Source:HGNC Symbol;Acc:HGNC:12527]
**-0,543694018**	1,55E-09	1,25E-08	*MRAS*	muscle RAS oncogene homolog [Source:HGNC Symbol;Acc:HGNC:7227]
**-0,543531427**	8,40E-13	9,01E-12	*GNB4*	G protein subunit beta 4 [Source:HGNC Symbol;Acc:HGNC:20731]
**-0,543396445**	7,22E-20	1,16E-18	*FUBP1*	far upstream element binding protein 1 [Source:HGNC Symbol;Acc:HGNC:4004]
**-0,542683269**	0,000412451	0,001683639	*CRHBP*	corticotropin releasing hormone binding protein [Source:HGNC Symbol;Acc:HGNC:2356]
**-0,542646065**	2,47E-08	1,79E-07	*HAT1*	histone acetyltransferase 1 [Source:HGNC Symbol;Acc:HGNC:4821]
**-0,542411552**	0,000136924	0,000610981	*LRCH1*	leucine rich repeats and calponin homology domain containing 1 [Source:HGNC Symbol;Acc:HGNC:20309]
**-0,541907557**	8,04E-30	1,95E-28	*EHD2*	EH domain containing 2 [Source:HGNC Symbol;Acc:HGNC:3243]
**-0,541819142**	4,00E-06	2,25E-05	*UTP15*	UTP15, small subunit processome component [Source:HGNC Symbol;Acc:HGNC:25758]
**-0,54179474**	0,000703322	0,002752302	*SH2D5*	SH2 domain containing 5 [Source:HGNC Symbol;Acc:HGNC:28819]
**-0,541516137**	1,86E-22	3,32E-21	*IFITM3*	interferon induced transmembrane protein 3 [Source:HGNC Symbol;Acc:HGNC:5414]
**-0,541286813**	8,96E-16	1,14E-14	*ANO6*	anoctamin 6 [Source:HGNC Symbol;Acc:HGNC:25240]

**-0,54122469**	1,21E-06	7,29E-06	*TTF2*	transcription termination factor 2 [Source:HGNC Symbol;Acc:HGNC:12398]
**-0,541185244**	0,000152697	0,000674692	*LRRC45*	leucine rich repeat containing 45 [Source:HGNC Symbol;Acc:HGNC:28302]
**-0,540679234**	0,000737558	0,002874682	*USP45*	ubiquitin specific peptidase 45 [Source:HGNC Symbol;Acc:HGNC:20080]
**-0,540261029**	1,34E-07	9,00E-07	*CHUK*	conserved helix-loop-helix ubiquitous kinase [Source:HGNC Symbol;Acc:HGNC:1974]
**-0,540039848**	8,67E-05	0,000401648	*TRIB3*	tribbles pseudokinase 3 [Source:HGNC Symbol;Acc:HGNC:16228]
**-0,539990101**	3,50E-20	5,67E-19	*NEDD9*	neural precursor cell expressed, developmentally down-regulated 9 [Source:HGNC Symbol;Acc:HGNC:7733]
**-0,539873799**	5,65E-12	5,61E-11	*FTSJ1*	FtsJ RNA methyltransferase homolog 1 [Source:HGNC Symbol;Acc:HGNC:13254]
**-0,539451653**	1,01E-11	9,81E-11	*FH*	fumarate hydratase [Source:HGNC Symbol;Acc:HGNC:3700]
**-0,539112727**	9,08E-05	0,0004194	*SRP19*	signal recognition particle 19 [Source:HGNC Symbol;Acc:HGNC:11300]
**-0,538649201**	1,37E-18	2,06E-17	*B4GALT2*	beta-1,4-galactosyltransferase 2 [Source:HGNC Symbol;Acc:HGNC:925]
**-0,538551049**	2,58E-11	2,44E-10	*KAZN*	kazrin, periplakin interacting protein [Source:HGNC Symbol;Acc:HGNC:29173]
**-0,538228095**	1,22E-17	1,76E-16	*PLEKHG2*	pleckstrin homology and RhoGEF domain containing G2 [Source:HGNC Symbol;Acc:HGNC:29515]
**-0,537883592**	1,80E-10	1,58E-09	*LGMN*	legumain [Source:HGNC Symbol;Acc:HGNC:9472]
**-0,537763142**	6,18E-14	7,14E-13	*LETM1*	leucine zipper and EF-hand containing transmembrane protein 1 [Source:HGNC Symbol;Acc:HGNC:6556]
**-0,537231588**	6,13E-13	6,65E-12	*MESD*	mesoderm development LRP chaperone [Source:HGNC Symbol;Acc:HGNC:13520]
**-0,536920726**	3,64E-07	2,33E-06	*DDX20*	DEAD-box helicase 20 [Source:HGNC Symbol;Acc:HGNC:2743]
**-0,536477447**	8,27E-05	0,000384165	*DCK*	deoxycytidine kinase [Source:HGNC Symbol;Acc:HGNC:2704]
**-0,535744376**	1,61E-13	1,81E-12	*NSUN2*	NOP2/Sun RNA methyltransferase family member 2 [Source:HGNC Symbol;Acc:HGNC:25994]
**-0,53535228**	7,95E-19	1,22E-17	*DNMT1*	DNA methyltransferase 1 [Source:HGNC Symbol;Acc:HGNC:2976]
**-0,535185508**	5,89E-11	5,35E-10	*RASSF4*	Ras association domain family member 4 [Source:HGNC Symbol;Acc:HGNC:20793]
**-0,535065253**	3,51E-16	4,58E-15	*POFUT2*	protein O-fucosyltransferase 2 [Source:HGNC Symbol;Acc:HGNC:14683]
**-0,534624908**	1,80E-12	1,88E-11	*SLC35F1*	solute carrier family 35 member F1 [Source:HGNC Symbol;Acc:HGNC:21483]
**-0,534040317**	2,13E-05	0,000108066	*ANKRD33B*	ankyrin repeat domain 33B [Source:HGNC Symbol;Acc:HGNC:35240]
**-0,53390688**	1,28E-10	1,13E-09	*CCDC137*	coiled-coil domain containing 137 [Source:HGNC Symbol;Acc:HGNC:33451]
**-0,533734588**	0,004555931	0,014869223	*NAALADL2*	N-acetylated alpha-linked acidic dipeptidase like 2 [Source:HGNC Symbol;Acc:HGNC:23219]
**-0,532880975**	3,16E-22	5,57E-21	*RAF1*	Raf-1 proto-oncogene, serine/threonine kinase [Source:HGNC Symbol;Acc:HGNC:9829]
**-0,532866806**	5,32E-15	6,53E-14	*WLS*	wntless Wnt ligand secretion mediator [Source:HGNC Symbol;Acc:HGNC:30238]
**-0,532783026**	1,52E-09	1,23E-08	*BLVRA*	biliverdin reductase A [Source:HGNC Symbol;Acc:HGNC:1062]
**-0,53263609**	1,10E-07	7,43E-07	*NEMP1*	nuclear envelope integral membrane protein 1 [Source:HGNC Symbol;Acc:HGNC:29001]
**-0,532432134**	2,63E-10	2,28E-09	*RNF126*	ring finger protein 126 [Source:HGNC Symbol;Acc:HGNC:21151]
**-0,532360131**	3,68E-21	6,28E-20	*ERBB2*	erb-b2 receptor tyrosine kinase 2 [Source:HGNC Symbol;Acc:HGNC:3430]

**-0,532009354**	3,17E-05	0,00015746	*DONSON*	downstream neighbor of SON [Source:HGNC Symbol;Acc:HGNC:2993]
**-0,531906901**	4,54E-14	5,30E-13	*NIPA2*	NIPA magnesium transporter 2 [Source:HGNC Symbol;Acc:HGNC:17044]
**-0,531793559**	0,009429629	0,028226344	*ZNF141*	zinc finger protein 141 [Source:HGNC Symbol;Acc:HGNC:12926]
**-0,531598124**	2,79E-07	1,81E-06	*GPD1L*	glycerol-3-phosphate dehydrogenase 1 like [Source:HGNC Symbol;Acc:HGNC:28956]
**-0,531401559**	0,010942825	0,032136269	*LIN9*	lin-9 DREAM MuvB core complex component [Source:HGNC Symbol;Acc:HGNC:30830]
**-0,531370291**	2,82E-09	2,23E-08	*C19orf48*	chromosome 19 open reading frame 48 [Source:HGNC Symbol;Acc:HGNC:29667]
**-0,530640024**	0,003132365	0,010632619	*FAM167A*	family with sequence similarity 167 member A [Source:HGNC Symbol;Acc:HGNC:15549]
**-0,530628786**	1,11E-05	5,88E-05	*MSTO1*	misato 1, mitochondrial distribution and morphology regulator [Source:HGNC Symbol;Acc:HGNC:29678]
**-0,530488051**	9,94E-13	1,06E-11	*EML4*	echinoderm microtubule associated protein like 4 [Source:HGNC Symbol;Acc:HGNC:1316]
**-0,530195309**	1,11E-17	1,61E-16	*PPARD*	peroxisome proliferator activated receptor delta [Source:HGNC Symbol;Acc:HGNC:9235]
**-0,52963647**	7,68E-26	1,60E-24	*PLCD3*	phospholipase C delta 3 [Source:HGNC Symbol;Acc:HGNC:9061]
**-0,52926321**	2,09E-05	0,000106485	*TRAF2*	TNF receptor associated factor 2 [Source:HGNC Symbol;Acc:HGNC:12032]
**-0,529163855**	3,06E-06	1,74E-05	*TMOD2*	tropomodulin 2 [Source:HGNC Symbol;Acc:HGNC:11872]
**-0,528881219**	1,34E-12	1,41E-11	*EIF3J*	eukaryotic translation initiation factor 3 subunit J [Source:HGNC Symbol;Acc:HGNC:3270]
**-0,528621568**	0,005563303	0,017753897	*MMACHC*	methylmalonic aciduria (cobalamin deficiency) cblC type, with homocystinuria [Source:HGNC Symbol;Acc:HGNC:24525]
**-0,528548776**	2,47E-05	0,000124463	*NTMT1*	N-terminal Xaa-Pro-Lys N-methyltransferase 1 [Source:HGNC Symbol;Acc:HGNC:23373]
**-0,528467618**	2,10E-16	2,80E-15	*CREB3*	cAMP responsive element binding protein 3 [Source:HGNC Symbol;Acc:HGNC:2347]
**-0,52817755**	1,05E-12	1,12E-11	*LRP6*	LDL receptor related protein 6 [Source:HGNC Symbol;Acc:HGNC:6698]
**-0,528084158**	7,67E-16	9,83E-15	*PLA2G16*	phospholipase A2 group XVI [Source:HGNC Symbol;Acc:HGNC:17825]
**-0,527924359**	4,12E-26	8,66E-25	*CCT2*	chaperonin containing TCP1 subunit 2 [Source:HGNC Symbol;Acc:HGNC:1615]
**-0,527847712**	4,93E-05	0,000237757	*FAT4*	FAT atypical cadherin 4 [Source:HGNC Symbol;Acc:HGNC:23109]
**-0,527084825**	0,014326389	0,040603972	*ACKR3*	atypical chemokine receptor 3 [Source:HGNC Symbol;Acc:HGNC:23692]
**-0,527019546**	0,000962305	0,003650928	*MB21D2*	Mab-21 domain containing 2 [Source:HGNC Symbol;Acc:HGNC:30438]
**-0,526837958**	6,11E-12	6,05E-11	*MUS81*	MUS81 structure-specific endonuclease subunit [Source:HGNC Symbol;Acc:HGNC:29814]
**-0,526748319**	3,53E-36	1,07E-34	*CD151*	CD151 molecule (Raph blood group) [Source:HGNC Symbol;Acc:HGNC:1630]
**-0,5254839**	2,27E-07	1,49E-06	*NCAPD3*	non-SMC condensin II complex subunit D3 [Source:HGNC Symbol;Acc:HGNC:28952]
**-0,525166823**	7,59E-32	1,99E-30	*MARCKS*	myristoylated alanine rich protein kinase C substrate [Source:HGNC Symbol;Acc:HGNC:6759]
**-0,524891519**	3,70E-05	0,000181696	*DCLK2*	doublecortin like kinase 2 [Source:HGNC Symbol;Acc:HGNC:19002]
**-0,52468572**	2,37E-16	3,14E-15	*SLC39A13*	solute carrier family 39 member 13 [Source:HGNC Symbol;Acc:HGNC:20859]
**-0,524258861**	1,69E-14	2,01E-13	*P4HA1*	prolyl 4-hydroxylase subunit alpha 1 [Source:HGNC Symbol;Acc:HGNC:8546]
**-0,523606278**	7,91E-07	4,87E-06	*KATNBL1*	katanin regulatory subunit B1 like 1 [Source:HGNC Symbol;Acc:HGNC:26199]

**-0,523314183**	0,002450042	0,008526813	*LRRC8B*	leucine rich repeat containing 8 VRAC subunit B [Source:HGNC Symbol;Acc:HGNC:30692]
**-0,523126328**	1,15E-05	6,04E-05	*KNOP1*	lysine rich nucleolar protein 1 [Source:HGNC Symbol;Acc:HGNC:34404]
**-0,521572747**	0,000928849	0,003542	*WDHD1*	WD repeat and HMG-box DNA binding protein 1 [Source:HGNC Symbol;Acc:HGNC:23170]
**-0,521469726**	3,10E-23	5,72E-22	*VARS*	valyl-tRNA synthetase [Source:HGNC Symbol;Acc:HGNC:12651]
**-0,520937852**	0,00152873	0,005557613	*FANCE*	FA complementation group E [Source:HGNC Symbol;Acc:HGNC:3586]
**-0,520843471**	3,73E-15	4,62E-14	*EIF1AX*	eukaryotic translation initiation factor 1A X-linked [Source:HGNC Symbol;Acc:HGNC:3250]
**-0,520715942**	1,46E-10	1,29E-09	*ZNF503*	zinc finger protein 503 [Source:HGNC Symbol;Acc:HGNC:23589]
**-0,520192203**	3,07E-09	2,42E-08	*IFNGR1*	interferon gamma receptor 1 [Source:HGNC Symbol;Acc:HGNC:5439]
**-0,519863364**	0,000843369	0,003249655	*DPF1*	double PHD fingers 1 [Source:HGNC Symbol;Acc:HGNC:20225]
**-0,519582196**	2,43E-13	2,70E-12	*PLEKHJ1*	pleckstrin homology domain containing J1 [Source:HGNC Symbol;Acc:HGNC:18211]
**-0,519431911**	8,32E-09	6,31E-08	*TMEM150A*	transmembrane protein 150A [Source:HGNC Symbol;Acc:HGNC:24677]
**-0,51941551**	8,25E-08	5,68E-07	*HGH1*	HGH1 homolog [Source:HGNC Symbol;Acc:HGNC:24161]
**-0,51934774**	1,93E-32	5,21E-31	*PTBP1*	polypyrimidine tract binding protein 1 [Source:HGNC Symbol;Acc:HGNC:9583]
**-0,518721253**	1,17E-15	1,49E-14	*CNR1*	cannabinoid receptor 1 [Source:HGNC Symbol;Acc:HGNC:2159]
**-0,518138788**	7,21E-11	6,51E-10	*DHX33*	DEAH-box helicase 33 [Source:HGNC Symbol;Acc:HGNC:16718]
**-0,517725709**	3,00E-10	2,58E-09	*PRPF4*	pre-mRNA processing factor 4 [Source:HGNC Symbol;Acc:HGNC:17349]
**-0,517578841**	1,81E-15	2,28E-14	*YWHAH*	tyrosine 3-monooxygenase/tryptophan 5-monooxygenase activation protein eta [Source:HGNC Symbol;Acc:HGNC:12853]
**-0,517474568**	0,000232435	0,000990668	*NDUFAF4*	NADH:ubiquinone oxidoreductase complex assembly factor 4 [Source:HGNC Symbol;Acc:HGNC:21034]
**-0,517200744**	5,36E-09	4,14E-08	*STX2*	syntaxin 2 [Source:HGNC Symbol;Acc:HGNC:3403]
**-0,517033038**	4,20E-17	5,84E-16	*EIF2S1*	eukaryotic translation initiation factor 2 subunit alpha [Source:HGNC Symbol;Acc:HGNC:3265]
**-0,516217014**	1,65E-11	1,58E-10	*RBM14*	RNA binding motif protein 14 [Source:HGNC Symbol;Acc:HGNC:14219]
**-0,516176866**	2,58E-07	1,68E-06	*IGF2BP1*	insulin like growth factor 2 mRNA binding protein 1 [Source:HGNC Symbol;Acc:HGNC:28866]
**-0,516089838**	0,004761261	0,015453594	*PWP2*	PWP2, small subunit processome component [Source:HGNC Symbol;Acc:HGNC:9711]
**-0,515597036**	2,56E-34	7,31E-33	*PTTG1IP*	PTTG1 interacting protein [Source:HGNC Symbol;Acc:HGNC:13524]
**-0,515449482**	0,002162075	0,007616548	*SHISAL1*	shisa like 1 [Source:HGNC Symbol;Acc:HGNC:29335]
**-0,515171946**	6,74E-12	6,65E-11	*POLR1A*	RNA polymerase I subunit A [Source:HGNC Symbol;Acc:HGNC:17264]
**-0,514112226**	7,79E-07	4,80E-06	*PPAT*	phosphoribosyl pyrophosphate amidotransferase [Source:HGNC Symbol;Acc:HGNC:9238]
**-0,514070829**	3,97E-40	1,35E-38	*PFN1*	profilin 1 [Source:HGNC Symbol;Acc:HGNC:8881]
**-0,514010292**	3,04E-05	0,000151533	*PDE6D*	phosphodiesterase 6D [Source:HGNC Symbol;Acc:HGNC:8788]
**-0,513958348**	0,001404725	0,005153135	*NDUFAF2*	NADH:ubiquinone oxidoreductase complex assembly factor 2 [Source:HGNC Symbol;Acc:HGNC:28086]
**-0,513847197**	0,009065262	0,027291419	*GPR135*	G protein-coupled receptor 135 [Source:HGNC Symbol;Acc:HGNC:19991]
**-0,512744035**	2,33E-10	2,03E-09	*ZBTB47*	zinc finger and BTB domain containing 47 [Source:HGNC Symbol;Acc:HGNC:26955]

**-0,512660453**	5,31E-08	3,73E-07	*SNRPD1*	small nuclear ribonucleoprotein D1 polypeptide [Source:HGNC Symbol;Acc:HGNC:11158]
**-0,512054494**	0,006981914	0,021678565	*ADAT2*	adenosine deaminase, tRNA specific 2 [Source:HGNC Symbol;Acc:HGNC:21172]
**-0,511741562**	4,48E-11	4,14E-10	*STARD3NL*	STARD3 N-terminal like [Source:HGNC Symbol;Acc:HGNC:19169]
**-0,511740124**	0,001512365	0,005510506	*CCDC113*	coiled-coil domain containing 113 [Source:HGNC Symbol;Acc:HGNC:25002]
**-0,511004943**	2,87E-24	5,61E-23	*EFNB2*	ephrin B2 [Source:HGNC Symbol;Acc:HGNC:3227]
**-0,510861332**	5,20E-19	8,02E-18	*ARPC4*	actin related protein 2/3 complex subunit 4 [Source:HGNC Symbol;Acc:HGNC:707]
**-0,51082504**	0,000943856	0,00359076	*POLD3*	DNA polymerase delta 3, accessory subunit [Source:HGNC Symbol;Acc:HGNC:20932]
**-0,510471799**	2,95E-16	3,88E-15	*TFDP1*	transcription factor Dp-1 [Source:HGNC Symbol;Acc:HGNC:11749]
**-0,50989786**	6,38E-06	3,49E-05	*BST1*	bone marrow stromal cell antigen 1 [Source:HGNC Symbol;Acc:HGNC:1118]
**-0,509100274**	2,16E-12	2,24E-11	*HSPA1A*	heat shock protein family A (Hsp70) member 1A [Source:HGNC Symbol;Acc:HGNC:5232]
**-0,509076832**	1,76E-09	1,42E-08	*ACTL6A*	actin like 6A [Source:HGNC Symbol;Acc:HGNC:24124]
**-0,508824801**	0,000362046	0,001490108	*CTU1*	cytosolic thiouridylase subunit 1 [Source:HGNC Symbol;Acc:HGNC:29590]
**-0,508629168**	6,66E-09	5,11E-08	*NACAD*	NAC alpha domain containing [Source:HGNC Symbol;Acc:HGNC:22196]
**-0,508513526**	9,38E-13	1,00E-11	*DYNLT1*	dynein light chain Tctex-type 1 [Source:HGNC Symbol;Acc:HGNC:11697]
**-0,508402211**	6,44E-11	5,84E-10	*FOXD1*	forkhead box D1 [Source:HGNC Symbol;Acc:HGNC:3802]
**-0,508004078**	8,94E-11	8,01E-10	*KMT5A*	lysine methyltransferase 5A [Source:HGNC Symbol;Acc:HGNC:29489]
**-0,507526277**	6,64E-12	6,56E-11	*SNRPB*	small nuclear ribonucleoprotein polypeptides B and B1 [Source:HGNC Symbol;Acc:HGNC:11153]
**-0,507292302**	1,49E-05	7,75E-05	*OSBPL10*	oxysterol binding protein like 10 [Source:HGNC Symbol;Acc:HGNC:16395]
**-0,50649115**	0,017517856	0,048267998	*WNT3*	Wnt family member 3 [Source:HGNC Symbol;Acc:HGNC:12782]
**-0,506340148**	4,41E-10	3,75E-09	*PCGF5*	polycomb group ring finger 5 [Source:HGNC Symbol;Acc:HGNC:28264]
**-0,506177266**	4,29E-05	0,000208869	*AKTIP*	AKT interacting protein [Source:HGNC Symbol;Acc:HGNC:16710]
**-0,505613472**	0,00027104	0,001140707	*CA2*	carbonic anhydrase 2 [Source:HGNC Symbol;Acc:HGNC:1373]
**-0,50555941**	2,93E-18	4,34E-17	*ATP2C1*	ATPase secretory pathway Ca2+ transporting 1 [Source:HGNC Symbol;Acc:HGNC:13211]
**-0,505262798**	0,000258076	0,00108969	*MEIS3*	Meis homeobox 3 [Source:HGNC Symbol;Acc:HGNC:29537]
**-0,505218677**	1,06E-07	7,18E-07	*LIMK2*	LIM domain kinase 2 [Source:HGNC Symbol;Acc:HGNC:6614]
**-0,50493084**	2,09E-08	1,53E-07	*DOHH*	deoxyhypusine hydroxylase [Source:HGNC Symbol;Acc:HGNC:28662]
**-0,504929878**	0,004594542	0,014975089	*SGIP1*	SH3 domain GRB2 like endophilin interacting protein 1 [Source:HGNC Symbol;Acc:HGNC:25412]
**-0,504539101**	4,16E-11	3,86E-10	*KLF16*	Kruppel like factor 16 [Source:HGNC Symbol;Acc:HGNC:16857]
**-0,504289917**	0,000179944	0,000784894	*CMC2*	C-X9-C motif containing 2 [Source:HGNC Symbol;Acc:HGNC:24447]
**-0,504078808**	0,001825267	0,006519625	*TNFSF9*	TNF superfamily member 9 [Source:HGNC Symbol;Acc:HGNC:11939]
**-0,503566689**	1,85E-08	1,36E-07	*TOR1B*	torsin family 1 member B [Source:HGNC Symbol;Acc:HGNC:11995]
**-0,503345129**	1,83E-16	2,43E-15	*CCDC50*	coiled-coil domain containing 50 [Source:HGNC Symbol;Acc:HGNC:18111]

**-0,503043721**	0,000284641	0,001192003	*TMEM200B*	transmembrane protein 200B [Source:HGNC Symbol;Acc:HGNC:33785]
**-0,502717384**	0,004089608	0,013469608	*TEDC1*	tubulin epsilon and delta complex 1 [Source:HGNC Symbol;Acc:HGNC:20127]
**-0,502213717**	1,94E-19	3,06E-18	*MRC2*	mannose receptor C type 2 [Source:HGNC Symbol;Acc:HGNC:16875]
**-0,502081194**	1,38E-20	2,28E-19	*ERBIN*	erbb2 interacting protein [Source:HGNC Symbol;Acc:HGNC:15842]
**-0,501876658**	3,84E-10	3,27E-09	*PNN*	pinin, desmosome associated protein [Source:HGNC Symbol;Acc:HGNC:9162]
**-0,501775194**	3,50E-12	3,54E-11	*DUS1L*	dihydrouridine synthase 1 like [Source:HGNC Symbol;Acc:HGNC:30086]
**-0,501555006**	4,66E-18	6,85E-17	*PLXNA1*	plexin A1 [Source:HGNC Symbol;Acc:HGNC:9099]
**-0,501392034**	3,80E-09	2,98E-08	*SRPRB*	SRP receptor subunit beta [Source:HGNC Symbol;Acc:HGNC:24085]
**-0,501268853**	0,000205141	0,000882502	*PROSER3*	proline and serine rich 3 [Source:HGNC Symbol;Acc:HGNC:25204]
**-0,500980192**	0,013613308	0,038884287	*COL11A2*	collagen type XI alpha 2 chain [Source:HGNC Symbol;Acc:HGNC:2187]
**-0,500605445**	9,86E-05	0,000452463	*KRT7*	keratin 7 [Source:HGNC Symbol;Acc:HGNC:6445]
**-0,500560257**	1,45E-07	9,67E-07	*NIPSNAP3A*	nipsnap homolog 3A [Source:HGNC Symbol;Acc:HGNC:23619]
**-0,500105414**	4,07E-12	4,10E-11	*CHST14*	carbohydrate sulfotransferase 14 [Source:HGNC Symbol;Acc:HGNC:24464]
**-0,500062363**	3,66E-12	3,70E-11	*ATIC*	5-aminoimidazole-4-carboxamide ribonucleotide formyltransferase/IMP cyclohydrolase [Source:HGNC Symbol;Acc:HGNC:794]
**-0,499988668**	0,010133616	0,030060067	*GEMIN6*	gem nuclear organelle associated protein 6 [Source:HGNC Symbol;Acc:HGNC:20044]
**-0,499606917**	9,72E-09	7,34E-08	*LRP12*	LDL receptor related protein 12 [Source:HGNC Symbol;Acc:HGNC:31708]
**-0,499585793**	2,34E-05	0,000118594	*LYPD6*	LY6/PLAUR domain containing 6 [Source:HGNC Symbol;Acc:HGNC:28751]
**-0,499372005**	1,51E-06	8,98E-06	*RNF150*	ring finger protein 150 [Source:HGNC Symbol;Acc:HGNC:23138]
**-0,499360937**	3,69E-12	3,73E-11	*GTF3A*	general transcription factor IIIA [Source:HGNC Symbol;Acc:HGNC:4662]
**-0,49873136**	5,24E-10	4,42E-09	*GLIS2*	GLIS family zinc finger 2 [Source:HGNC Symbol;Acc:HGNC:29450]
**-0,498669035**	7,20E-05	0,000337775	*DNAL1*	dynein axonemal light chain 1 [Source:HGNC Symbol;Acc:HGNC:23247]
**-0,498501132**	1,46E-17	2,09E-16	*DEK*	DEK proto-oncogene [Source:HGNC Symbol;Acc:HGNC:2768]
**-0,49821985**	8,03E-06	4,34E-05	*ABHD5*	abhydrolase domain containing 5 [Source:HGNC Symbol;Acc:HGNC:21396]
**-0,498179676**	0,017190597	0,047494409	*CASP1*	caspase 1 [Source:HGNC Symbol;Acc:HGNC:1499]
**-0,497495558**	8,92E-06	4,78E-05	*DCUN1D3*	defective in cullin neddylation 1 domain containing 3 [Source:HGNC Symbol;Acc:HGNC:28734]
**-0,497445171**	2,28E-12	2,36E-11	*PNMA2*	PNMA family member 2 [Source:HGNC Symbol;Acc:HGNC:9159]
**-0,497377936**	0,001044867	0,003937221	*C1orf131*	chromosome 1 open reading frame 131 [Source:HGNC Symbol;Acc:HGNC:25332]
**-0,497286359**	3,49E-05	0,000172075	*RIOX2*	ribosomal oxygenase 2 [Source:HGNC Symbol;Acc:HGNC:19441]
**-0,497052541**	0,000147257	0,000652287	*ADAMTS9*	ADAM metallopeptidase with thrombospondin type 1 motif 9 [Source:HGNC Symbol;Acc:HGNC:13202]
**-0,496569776**	1,36E-17	1,95E-16	*IRS1*	insulin receptor substrate 1 [Source:HGNC Symbol;Acc:HGNC:6125]
**-0,496503704**	6,96E-08	4,83E-07	*C1QTNF6*	C1q and TNF related 6 [Source:HGNC Symbol;Acc:HGNC:14343]
**-0,495890864**	5,68E-19	8,76E-18	*PPP4R1*	protein phosphatase 4 regulatory subunit 1 [Source:HGNC Symbol;Acc:HGNC:9320]

**-0,495758948**	5,02E-07	3,16E-06	*COMMD2*	COMM domain containing 2 [Source:HGNC Symbol;Acc:HGNC:24993]
**-0,495672683**	1,07E-21	1,86E-20	*F2R*	coagulation factor II thrombin receptor [Source:HGNC Symbol;Acc:HGNC:3537]
**-0,495335967**	0,00652816	0,020495952	*CEP57L1*	centrosomal protein 57 like 1 [Source:HGNC Symbol;Acc:HGNC:21561]
**-0,494452094**	1,62E-05	8,36E-05	*RIN3*	Ras and Rab interactor 3 [Source:HGNC Symbol;Acc:HGNC:18751]
**-0,494315369**	4,40E-11	4,07E-10	*NCBP1*	nuclear cap binding protein subunit 1 [Source:HGNC Symbol;Acc:HGNC:7658]
**-0,494266001**	2,70E-18	4,01E-17	*YARS*	tyrosyl-tRNA synthetase [Source:HGNC Symbol;Acc:HGNC:12840]
**-0,494193912**	0,000893237	0,003418291	*FAT1*	FAT atypical cadherin 1 [Source:HGNC Symbol;Acc:HGNC:3595]
**-0,493398954**	6,35E-12	6,28E-11	*PLEKHO1*	pleckstrin homology domain containing O1 [Source:HGNC Symbol;Acc:HGNC:24310]
**-0,493335548**	6,89E-05	0,000324484	*HES4*	hes family bHLH transcription factor 4 [Source:HGNC Symbol;Acc:HGNC:24149]
**-0,492362426**	3,41E-10	2,92E-09	*MPP5*	membrane palmitoylated protein 5 [Source:HGNC Symbol;Acc:HGNC:18669]
**-0,491634448**	6,34E-14	7,31E-13	*ADAMTS12*	ADAM metallopeptidase with thrombospondin type 1 motif 12 [Source:HGNC Symbol;Acc:HGNC:14605]
**-0,491572758**	5,23E-06	2,89E-05	*MZT1*	mitotic spindle organizing protein 1 [Source:HGNC Symbol;Acc:HGNC:33830]
**-0,491427759**	8,41E-07	5,16E-06	*BAG2*	BCL2 associated athanogene 2 [Source:HGNC Symbol;Acc:HGNC:938]
**-0,491140129**	2,04E-18	3,05E-17	*EFR3A*	EFR3 homolog A [Source:HGNC Symbol;Acc:HGNC:28970]
**-0,490980625**	2,70E-16	3,56E-15	*CSE1L*	chromosome segregation 1 like [Source:HGNC Symbol;Acc:HGNC:2431]
**-0,490621483**	3,66E-26	7,71E-25	*CAV1*	caveolin 1 [Source:HGNC Symbol;Acc:HGNC:1527]
**-0,490519246**	2,92E-07	1,89E-06	*ZSWIM6*	zinc finger SWIM-type containing 6 [Source:HGNC Symbol;Acc:HGNC:29316]
**-0,490425241**	5,55E-15	6,77E-14	*FOXN3*	forkhead box N3 [Source:HGNC Symbol;Acc:HGNC:1928]
**-0,490281839**	4,51E-06	2,51E-05	*BNC2*	basonuclin 2 [Source:HGNC Symbol;Acc:HGNC:30988]
**-0,490193284**	0,00370608	0,012348934	*PLS1*	plastin 1 [Source:HGNC Symbol;Acc:HGNC:9090]
**-0,489812722**	0,000369245	0,001518129	*WRAP53*	WD repeat containing antisense to TP53 [Source:HGNC Symbol;Acc:HGNC:25522]
**-0,489119098**	6,91E-12	6,80E-11	*DESI1*	desumoylating isopeptidase 1 [Source:HGNC Symbol;Acc:HGNC:24577]
**-0,488552462**	9,39E-06	5,02E-05	*ZNF275*	zinc finger protein 275 [Source:HGNC Symbol;Acc:HGNC:13069]
**-0,488296344**	8,05E-09	6,11E-08	*CHRDL1*	chordin like 1 [Source:HGNC Symbol;Acc:HGNC:29861]
**-0,487989249**	0,005016646	0,016190426	*ARHGAP19*	Rho GTPase activating protein 19 [Source:HGNC Symbol;Acc:HGNC:23724]
**-0,487940266**	1,45E-14	1,73E-13	*WDR45B*	WD repeat domain 45B [Source:HGNC Symbol;Acc:HGNC:25072]
**-0,487918104**	2,95E-08	2,12E-07	*TNS1*	tensin 1 [Source:HGNC Symbol;Acc:HGNC:11973]
**-0,487504322**	0,008480143	0,025749594	*SHISA2*	shisa family member 2 [Source:HGNC Symbol;Acc:HGNC:20366]
**-0,487447716**	9,15E-19	1,40E-17	*SEC23A*	Sec23 homolog A, coat complex II component [Source:HGNC Symbol;Acc:HGNC:10701]
**-0,487303525**	4,05E-08	2,87E-07	*PDCL3*	phosducin like 3 [Source:HGNC Symbol;Acc:HGNC:28860]
**-0,48717129**	3,77E-10	3,22E-09	*SLC31A1*	solute carrier family 31 member 1 [Source:HGNC Symbol;Acc:HGNC:11016]
**-0,486397418**	5,55E-16	7,17E-15	*PALM*	paralemmin [Source:HGNC Symbol;Acc:HGNC:8594]
**-0,486341954**	1,16E-09	9,50E-09	*CEP89*	centrosomal protein 89 [Source:HGNC Symbol;Acc:HGNC:25907]
**-0,486275199**	3,09E-22	5,45E-21	*TUBA1C*	tubulin alpha 1c [Source:HGNC Symbol;Acc:HGNC:20768]

**-0,485923313**	5,11E-16	6,62E-15	*UBE2G2*	ubiquitin conjugating enzyme E2 G2 [Source:HGNC Symbol;Acc:HGNC:12483]
**-0,485909172**	4,62E-13	5,06E-12	*ABHD12*	abhydrolase domain containing 12 [Source:HGNC Symbol;Acc:HGNC:15868]
**-0,485774587**	2,96E-05	0,000147831	*FAM118A*	family with sequence similarity 118 member A [Source:HGNC Symbol;Acc:HGNC:1313]
**-0,485693941**	7,75E-08	5,35E-07	*BTN2A1*	butyrophilin subfamily 2 member A1 [Source:HGNC Symbol;Acc:HGNC:1136]
**-0,48559463**	0,001060226	0,003986581	*ATRIP*	ATR interacting protein [Source:HGNC Symbol;Acc:HGNC:33499]
**-0,484949814**	5,25E-09	4,06E-08	*XXYLT1*	xyloside xylosyltransferase 1 [Source:HGNC Symbol;Acc:HGNC:26639]
**-0,484861128**	0,006572178	0,02061414	*DUSP10*	dual specificity phosphatase 10 [Source:HGNC Symbol;Acc:HGNC:3065]
**-0,484860353**	8,43E-13	9,04E-12	*SMC1A*	structural maintenance of chromosomes 1A [Source:HGNC Symbol;Acc:HGNC:11111]
**-0,484722403**	0,0072699	0,022472075	*TMEM267*	transmembrane protein 267 [Source:HGNC Symbol;Acc:HGNC:26139]
**-0,484263755**	3,84E-18	5,68E-17	*KDELR2*	KDEL endoplasmic reticulum protein retention receptor 2 [Source:HGNC Symbol;Acc:HGNC:6305]
**-0,482890037**	0,007912506	0,024200172	*CENPH*	centromere protein H [Source:HGNC Symbol;Acc:HGNC:17268]
**-0,48275798**	3,40E-11	3,17E-10	*SLC35C1*	solute carrier family 35 member C1 [Source:HGNC Symbol;Acc:HGNC:20197]
**-0,482418013**	2,33E-06	1,35E-05	*TAP2*	transporter 2, ATP binding cassette subfamily B member [Source:HGNC Symbol;Acc:HGNC:44]
**-0,482393568**	6,00E-21	1,01E-19	*LRRN1*	leucine rich repeat neuronal 1 [Source:HGNC Symbol;Acc:HGNC:20980]
**-0,481999337**	7,56E-07	4,67E-06	*CCNYL1*	cyclin Y like 1 [Source:HGNC Symbol;Acc:HGNC:26868]
**-0,481983412**	0,001560731	0,005659108	*ADRA1B*	adrenoceptor alpha 1B [Source:HGNC Symbol;Acc:HGNC:278]
**-0,481287492**	3,85E-13	4,24E-12	*BOP1*	block of proliferation 1 [Source:HGNC Symbol;Acc:HGNC:15519]
**-0,481141369**	1,02E-16	1,38E-15	*CLUH*	clustered mitochondria homolog [Source:HGNC Symbol;Acc:HGNC:29094]
**-0,480910047**	1,52E-24	3,00E-23	*HSPD1*	heat shock protein family D (Hsp60) member 1 [Source:HGNC Symbol;Acc:HGNC:5261]
**-0,480770378**	5,91E-06	3,25E-05	*TWNK*	twinkle mtDNA helicase [Source:HGNC Symbol;Acc:HGNC:1160]
**-0,480525768**	0,005191623	0,016694057	*TUBE1*	tubulin epsilon 1 [Source:HGNC Symbol;Acc:HGNC:20775]
**-0,480400749**	3,16E-20	5,14E-19	*DHX15*	DEAH-box helicase 15 [Source:HGNC Symbol;Acc:HGNC:2738]
**-0,480331922**	3,71E-11	3,45E-10	*PHGDH*	phosphoglycerate dehydrogenase [Source:HGNC Symbol;Acc:HGNC:8923]
**-0,480231328**	8,07E-10	6,70E-09	*SMC4*	structural maintenance of chromosomes 4 [Source:HGNC Symbol;Acc:HGNC:14013]
**-0,48009991**	1,35E-06	8,08E-06	*TRMT61A*	tRNA methyltransferase 61A [Source:HGNC Symbol;Acc:HGNC:23790]
**-0,479950118**	7,40E-10	6,16E-09	*PHLPP1*	PH domain and leucine rich repeat protein phosphatase 1 [Source:HGNC Symbol;Acc:HGNC:20610]
**-0,479855095**	8,63E-06	4,65E-05	*TXNDC11*	thioredoxin domain containing 11 [Source:HGNC Symbol;Acc:HGNC:28030]
**-0,479392363**	2,56E-09	2,03E-08	*SELENOM*	selenoprotein M [Source:HGNC Symbol;Acc:HGNC:30397]
**-0,479248872**	2,28E-07	1,49E-06	*HSPA12A*	heat shock protein family A (Hsp70) member 12A [Source:HGNC Symbol;Acc:HGNC:19022]
**-0,47911828**	0,000108202	0,00049175	*SCNN1A*	sodium channel epithelial 1 alpha subunit [Source:HGNC Symbol;Acc:HGNC:10599]
**-0,478276446**	1,77E-18	2,66E-17	*PAICS*	phosphoribosylaminoimidazole carboxylase and phosphoribosylaminoimidazolesuccinocarboxamide synthase [Source:HGNC Symbol;Acc:HGNC:8587]

**-0,478142231**	0,000283279	0,001187069	*TMEM268*	transmembrane protein 268 [Source:HGNC Symbol;Acc:HGNC:24513]
**-0,477918449**	1,81E-06	1,07E-05	*CSGALNACT2*	chondroitin sulfate N-acetylgalactosaminyltransferase 2 [Source:HGNC Symbol;Acc:HGNC:24292]
**-0,477865331**	4,91E-05	0,000236989	*FOXL1*	forkhead box L1 [Source:HGNC Symbol;Acc:HGNC:3817]
**-0,477523829**	2,46E-05	0,000124335	*TRMU*	tRNA 5-methylaminomethyl-2-thiouridylate methyltransferase [Source:HGNC Symbol;Acc:HGNC:25481]
**-0,477391216**	2,39E-06	1,38E-05	*TOP3A*	DNA topoisomerase III alpha [Source:HGNC Symbol;Acc:HGNC:11992]
**-0,477250286**	1,66E-05	8,54E-05	*TDP1*	tyrosyl-DNA phosphodiesterase 1 [Source:HGNC Symbol;Acc:HGNC:18884]
**-0,476812279**	3,62E-06	2,05E-05	*DAGLB*	diacylglycerol lipase beta [Source:HGNC Symbol;Acc:HGNC:28923]
**-0,476785497**	2,41E-30	6,01E-29	*MAP4*	microtubule associated protein 4 [Source:HGNC Symbol;Acc:HGNC:6862]
**-0,47607709**	3,58E-12	3,62E-11	*BCL2L1*	BCL2 like 1 [Source:HGNC Symbol;Acc:HGNC:992]
**-0,476030275**	9,84E-08	6,72E-07	*ATP5MC1*	ATP synthase membrane subunit c locus 1 [Source:HGNC Symbol;Acc:HGNC:841]
**-0,475962281**	1,66E-08	1,23E-07	*CDR2L*	cerebellar degeneration related protein 2 like [Source:HGNC Symbol;Acc:HGNC:29999]
**-0,475854149**	3,04E-11	2,85E-10	*KLHL5*	kelch like family member 5 [Source:HGNC Symbol;Acc:HGNC:6356]
**-0,475240217**	0,00033152	0,00137233	*TTC27*	tetratricopeptide repeat domain 27 [Source:HGNC Symbol;Acc:HGNC:25986]
**-0,475037805**	1,99E-16	2,65E-15	*RBM3*	RNA binding motif protein 3 [Source:HGNC Symbol;Acc:HGNC:9900]
**-0,474496596**	2,44E-12	2,53E-11	*PA2G4*	proliferation-associated 2G4 [Source:HGNC Symbol;Acc:HGNC:8550]
**-0,474060341**	5,34E-09	4,12E-08	*NUP153*	nucleoporin 153 [Source:HGNC Symbol;Acc:HGNC:8062]
**-0,473857497**	7,67E-11	6,90E-10	*NUP205*	nucleoporin 205 [Source:HGNC Symbol;Acc:HGNC:18658]
**-0,473679271**	2,23E-06	1,30E-05	*RRP9*	ribosomal RNA processing 9, U3 small nucleolar RNA binding protein [Source:HGNC Symbol;Acc:HGNC:16829]
**-0,473444946**	1,61E-10	1,42E-09	*DDX18*	DEAD-box helicase 18 [Source:HGNC Symbol;Acc:HGNC:2741]
**-0,4732221**	0,00833202	0,025335503	*RNF144B*	ring finger protein 144B [Source:HGNC Symbol;Acc:HGNC:21578]
**-0,473018611**	0,01396113	0,03972576	*ARHGEF18*	Rho/Rac guanine nucleotide exchange factor 18 [Source:HGNC Symbol;Acc:HGNC:17090]
**-0,472922207**	4,03E-10	3,44E-09	*HMOX2*	heme oxygenase 2 [Source:HGNC Symbol;Acc:HGNC:5014]
**-0,472821647**	0,017194379	0,047498094	*ADSL*	adenylosuccinate lyase [Source:HGNC Symbol;Acc:HGNC:291]
**-0,472713849**	3,50E-16	4,58E-15	*PTPRM*	protein tyrosine phosphatase, receptor type M [Source:HGNC Symbol;Acc:HGNC:9675]
**-0,472234395**	5,59E-06	3,08E-05	*ENOPH1*	enolase-phosphatase 1 [Source:HGNC Symbol;Acc:HGNC:24599]
**-0,472173151**	3,15E-07	2,03E-06	*IGSF3*	immunoglobulin superfamily member 3 [Source:HGNC Symbol;Acc:HGNC:5950]
**-0,471931651**	1,48E-07	9,87E-07	*GRPEL1*	GrpE like 1, mitochondrial [Source:HGNC Symbol;Acc:HGNC:19696]
**-0,471764816**	0,007360844	0,022709784	*DBF4*	DBF4 zinc finger [Source:HGNC Symbol;Acc:HGNC:17364]
**-0,471611508**	4,14E-07	2,63E-06	*ROR1*	receptor tyrosine kinase like orphan receptor 1 [Source:HGNC Symbol;Acc:HGNC:10256]
**-0,471479808**	0,003739758	0,012441924	*RBMS3*	RNA binding motif single stranded interacting protein 3 [Source:HGNC Symbol;Acc:HGNC:13427]
**-0,471353322**	0,001033411	0,003898595	*ACHE*	acetylcholinesterase (Cartwright blood group) [Source:HGNC Symbol;Acc:HGNC:108]
**-0,471271503**	1,21E-28	2,81E-27	*MSN*	moesin [Source:HGNC Symbol;Acc:HGNC:7373]

**-0,471156288**	1,95E-10	1,71E-09	*HMGB3*	high mobility group box 3 [Source:HGNC Symbol;Acc:HGNC:5004]
**-0,470976495**	2,34E-12	2,43E-11	*FZD2*	frizzled class receptor 2 [Source:HGNC Symbol;Acc:HGNC:4040]
**-0,470603615**	1,45E-06	8,67E-06	*TMEM87B*	transmembrane protein 87B [Source:HGNC Symbol;Acc:HGNC:25913]
**-0,47036874**	0,00574918	0,018283936	*PRXL2C*	peroxiredoxin like 2C [Source:HGNC Symbol;Acc:HGNC:16881]
**-0,470340079**	1,65E-08	1,22E-07	*NOL11*	nucleolar protein 11 [Source:HGNC Symbol;Acc:HGNC:24557]
**-0,470204029**	6,54E-08	4,55E-07	*SELENOS*	selenoprotein S [Source:HGNC Symbol;Acc:HGNC:30396]
**-0,469411058**	1,25E-06	7,50E-06	*NEBL*	nebulette [Source:HGNC Symbol;Acc:HGNC:16932]
**-0,469329358**	8,82E-07	5,41E-06	*ODF2*	outer dense fiber of sperm tails 2 [Source:HGNC Symbol;Acc:HGNC:8114]
**-0,469287417**	0,000133739	0,000598554	*UCHL5*	ubiquitin C-terminal hydrolase L5 [Source:HGNC Symbol;Acc:HGNC:19678]
**-0,469266587**	0,005485301	0,017536696	*LRRC8C*	leucine rich repeat containing 8 VRAC subunit C [Source:HGNC Symbol;Acc:HGNC:25075]
**-0,469163164**	1,53E-10	1,35E-09	*SLC2A6*	solute carrier family 2 member 6 [Source:HGNC Symbol;Acc:HGNC:11011]
**-0,469106643**	6,87E-12	6,77E-11	*RBMS2*	RNA binding motif single stranded interacting protein 2 [Source:HGNC Symbol;Acc:HGNC:9909]
**-0,469035422**	7,87E-05	0,000367022	*RASL10B*	RAS like family 10 member B [Source:HGNC Symbol;Acc:HGNC:30295]
**-0,469033879**	4,30E-11	3,99E-10	*FAR1*	fatty acyl-CoA reductase 1 [Source:HGNC Symbol;Acc:HGNC:26222]
**-0,468834445**	1,61E-06	9,58E-06	*SLC35B4*	solute carrier family 35 member B4 [Source:HGNC Symbol;Acc:HGNC:20584]
**-0,468717724**	0,000696755	0,002729357	*DTD2*	D-tyrosyl-tRNA deacylase 2 (putative) [Source:HGNC Symbol;Acc:HGNC:20277]
**-0,468565823**	7,92E-23	1,44E-21	*CD24*	CD24 molecule [Source:HGNC Symbol;Acc:HGNC:1645]
**-0,468399664**	4,84E-38	1,55E-36	*CFL1*	cofilin 1 [Source:HGNC Symbol;Acc:HGNC:1874]
**-0,468318092**	0,000117889	0,000532657	*ARHGAP33*	Rho GTPase activating protein 33 [Source:HGNC Symbol;Acc:HGNC:23085]
**-0,468026216**	2,53E-24	4,96E-23	*ITGB8*	integrin subunit beta 8 [Source:HGNC Symbol;Acc:HGNC:6163]
**-0,467817964**	1,78E-07	1,18E-06	*EVI5*	ecotropic viral integration site 5 [Source:HGNC Symbol;Acc:HGNC:3501]
**-0,467081242**	7,73E-06	4,19E-05	*ANKRD13B*	ankyrin repeat domain 13B [Source:HGNC Symbol;Acc:HGNC:26363]
**-0,466844611**	2,24E-05	0,000113834	*PAPSS2*	3'-phosphoadenosine 5'-phosphosulfate synthase 2 [Source:HGNC Symbol;Acc:HGNC:8604]
**-0,466793115**	6,68E-16	8,60E-15	*SEPT8*	septin 8 [Source:HGNC Symbol;Acc:HGNC:16511]
**-0,465953551**	1,90E-07	1,25E-06	*PGAM5*	PGAM family member 5, mitochondrial serine/threonine protein phosphatase [Source:HGNC Symbol;Acc:HGNC:28763]
**-0,465847217**	1,22E-08	9,11E-08	*IKBIP*	IKBKB interacting protein [Source:HGNC Symbol;Acc:HGNC:26430]
**-0,464773801**	0,000145247	0,00064412	*TICAM1*	toll like receptor adaptor molecule 1 [Source:HGNC Symbol;Acc:HGNC:18348]
**-0,463519489**	2,74E-18	4,07E-17	*SYNCRIP*	synaptotagmin binding cytoplasmic RNA interacting protein [Source:HGNC Symbol;Acc:HGNC:16918]
**-0,463418521**	1,36E-05	7,08E-05	*ABCF2*	ATP binding cassette subfamily F member 2 [Source:HGNC Symbol;Acc:HGNC:71]
**-0,46326986**	9,94E-05	0,000455618	*ISOC1*	isochorismatase domain containing 1 [Source:HGNC Symbol;Acc:HGNC:24254]
**-0,462992182**	0,00948207	0,028357074	*HSPA14*	heat shock protein family A (Hsp70) member 14 [Source:HGNC Symbol;Acc:HGNC:29526]
**-0,462189324**	0,00123443	0,004575997	*COQ2*	coenzyme Q2, polyprenyltransferase [Source:HGNC Symbol;Acc:HGNC:25223]
**-0,461983996**	1,13E-05	5,96E-05	*DPH2*	DPH2 homolog [Source:HGNC Symbol;Acc:HGNC:3004]

**-0,461949369**	0,000252158	0,001067725	*BRIX1*	BRX1, biogenesis of ribosomes [Source:HGNC Symbol;Acc:HGNC:24170]
**-0,461279324**	0,002579504	0,008914726	*TXNDC5*	thioredoxin domain containing 5 [Source:HGNC Symbol;Acc:HGNC:21073]
**-0,461240104**	4,62E-12	4,63E-11	*HNRNPH1*	heterogeneous nuclear ribonucleoprotein H1 [Source:HGNC Symbol;Acc:HGNC:5041]
**-0,461098651**	1,45E-08	1,08E-07	*STK10*	serine/threonine kinase 10 [Source:HGNC Symbol;Acc:HGNC:11388]
**-0,460414871**	3,53E-09	2,77E-08	*RRM1*	ribonucleotide reductase catalytic subunit M1 [Source:HGNC Symbol;Acc:HGNC:10451]
**-0,460102365**	1,35E-10	1,20E-09	*PTBP3*	polypyrimidine tract binding protein 3 [Source:HGNC Symbol;Acc:HGNC:10253]
**-0,45957887**	4,14E-09	3,23E-08	*SKA2*	spindle and kinetochore associated complex subunit 2 [Source:HGNC Symbol;Acc:HGNC:28006]
**-0,459348528**	9,41E-05	0,000433385	*EGR1*	early growth response 1 [Source:HGNC Symbol;Acc:HGNC:3238]
**-0,458396658**	1,76E-08	1,30E-07	*CACYBP*	calcyclin binding protein [Source:HGNC Symbol;Acc:HGNC:30423]
**-0,458243791**	0,000333404	0,001379541	*PTTG1*	pituitary tumor-transforming 1 [Source:HGNC Symbol;Acc:HGNC:9690]
**-0,458215309**	1,22E-09	9,90E-09	*DDR2*	discoidin domain receptor tyrosine kinase 2 [Source:HGNC Symbol;Acc:HGNC:2731]
**-0,458085638**	0,005543968	0,017700927	*DENND1B*	DENN domain containing 1B [Source:HGNC Symbol;Acc:HGNC:28404]
**-0,457938389**	0,000134975	0,000603809	*CNEP1R1*	CTD nuclear envelope phosphatase 1 regulatory subunit 1 [Source:HGNC Symbol;Acc:HGNC:26759]
**-0,457805479**	6,19E-07	3,85E-06	*WDR77*	WD repeat domain 77 [Source:HGNC Symbol;Acc:HGNC:29652]
**-0,457160706**	0,001935222	0,006875807	*HOMER1*	homer scaffold protein 1 [Source:HGNC Symbol;Acc:HGNC:17512]
**-0,45712191**	7,86E-12	7,70E-11	*OSTC*	oligosaccharyltransferase complex non-catalytic subunit [Source:HGNC Symbol;Acc:HGNC:24448]
**-0,456770515**	0,000576171	0,002286073	*TMEM18*	transmembrane protein 18 [Source:HGNC Symbol;Acc:HGNC:25257]
**-0,456747033**	3,49E-12	3,54E-11	*DUSP3*	dual specificity phosphatase 3 [Source:HGNC Symbol;Acc:HGNC:3069]
**-0,456658053**	0,000165111	0,000724754	*MED21*	mediator complex subunit 21 [Source:HGNC Symbol;Acc:HGNC:11473]
**-0,456620744**	8,70E-08	5,97E-07	*SLC27A4*	solute carrier family 27 member 4 [Source:HGNC Symbol;Acc:HGNC:10998]
**-0,455877928**	0,000205032	0,00088223	*LCMT2*	leucine carboxyl methyltransferase 2 [Source:HGNC Symbol;Acc:HGNC:17558]
**-0,455864546**	3,67E-21	6,26E-20	*DAG1*	dystroglycan 1 [Source:HGNC Symbol;Acc:HGNC:2666]
**-0,455846121**	4,34E-17	6,02E-16	*PTPRF*	protein tyrosine phosphatase, receptor type F [Source:HGNC Symbol;Acc:HGNC:9670]
**-0,455703328**	0,000177023	0,000773021	*MAP7D3*	MAP7 domain containing 3 [Source:HGNC Symbol;Acc:HGNC:25742]
**-0,455414753**	1,32E-12	1,39E-11	*BICD2*	BICD cargo adaptor 2 [Source:HGNC Symbol;Acc:HGNC:17208]
**-0,455282137**	1,48E-07	9,87E-07	*LSG1*	large 60S subunit nuclear export GTPase 1 [Source:HGNC Symbol;Acc:HGNC:25652]
**-0,454835635**	0,002583132	0,008924383	*PDZD4*	PDZ domain containing 4 [Source:HGNC Symbol;Acc:HGNC:21167]
**-0,454616298**	1,06E-07	7,20E-07	*BLMH*	bleomycin hydrolase [Source:HGNC Symbol;Acc:HGNC:1059]
**-0,453906575**	2,85E-13	3,16E-12	*GSPT1*	G1 to S phase transition 1 [Source:HGNC Symbol;Acc:HGNC:4621]
**-0,453707375**	9,12E-08	6,25E-07	*ZNF598*	zinc finger protein 598 [Source:HGNC Symbol;Acc:HGNC:28079]
**-0,453599938**	4,12E-06	2,31E-05	*RTL10*	retrotransposon Gag like 10 [Source:HGNC Symbol;Acc:HGNC:26112]

**-0,453336659**	3,26E-06	1,85E-05	*DCUN1D5*	defective in cullin neddylation 1 domain containing 5 [Source:HGNC Symbol;Acc:HGNC:28409]
**-0,453300661**	1,21E-23	2,27E-22	*MCL1*	MCL1, BCL2 family apoptosis regulator [Source:HGNC Symbol;Acc:HGNC:6943]
**-0,453053896**	0,010027357	0,029776723	*MPP1*	membrane palmitoylated protein 1 [Source:HGNC Symbol;Acc:HGNC:7219]
**-0,453041635**	0,011636856	0,033953515	*PGBD1*	piggyBac transposable element derived 1 [Source:HGNC Symbol;Acc:HGNC:19398]
**-0,452648093**	4,60E-05	0,000223229	*POP7*	POP7 homolog, ribonuclease P/MRP subunit [Source:HGNC Symbol;Acc:HGNC:19949]
**-0,451993605**	9,11E-17	1,24E-15	*AXL*	AXL receptor tyrosine kinase [Source:HGNC Symbol;Acc:HGNC:905]
**-0,451981056**	0,001152034	0,004302611	*CBX2*	chromobox 2 [Source:HGNC Symbol;Acc:HGNC:1552]
**-0,451837264**	3,83E-07	2,44E-06	*PLA2G15*	phospholipase A2 group XV [Source:HGNC Symbol;Acc:HGNC:17163]
**-0,451647168**	5,70E-11	5,19E-10	*BIRC2*	baculoviral IAP repeat containing 2 [Source:HGNC Symbol;Acc:HGNC:590]
**-0,451350944**	0,000757817	0,002945362	*TSEN54*	tRNA splicing endonuclease subunit 54 [Source:HGNC Symbol;Acc:HGNC:27561]
**-0,451228141**	1,31E-05	6,86E-05	*CDK7*	cyclin dependent kinase 7 [Source:HGNC Symbol;Acc:HGNC:1778]
**-0,450993821**	6,59E-06	3,60E-05	*SNTA1*	syntrophin alpha 1 [Source:HGNC Symbol;Acc:HGNC:11167]
**-0,450954212**	1,60E-06	9,50E-06	*PLA2G12A*	phospholipase A2 group XIIA [Source:HGNC Symbol;Acc:HGNC:18554]
**-0,450832714**	9,87E-12	9,61E-11	*NUP93*	nucleoporin 93 [Source:HGNC Symbol;Acc:HGNC:28958]
**-0,450642423**	8,72E-05	0,000404069	*SSSCA1*	Sjogren syndrome/scleroderma autoantigen 1 [Source:HGNC Symbol;Acc:HGNC:11328]
**-0,450586548**	1,10E-26	2,37E-25	*ARPC2*	actin related protein 2/3 complex subunit 2 [Source:HGNC Symbol;Acc:HGNC:705]
**-0,4504562**	1,76E-05	9,05E-05	*C17orf51*	chromosome 17 open reading frame 51 [Source:HGNC Symbol;Acc:HGNC:27904]
**-0,450221078**	2,53E-20	4,14E-19	*SLC4A2*	solute carrier family 4 member 2 [Source:HGNC Symbol;Acc:HGNC:11028]
**-0,449537492**	2,15E-13	2,40E-12	*GPD2*	glycerol-3-phosphate dehydrogenase 2 [Source:HGNC Symbol;Acc:HGNC:4456]
**-0,449451653**	7,54E-06	4,09E-05	*FAM207A*	family with sequence similarity 207 member A [Source:HGNC Symbol;Acc:HGNC:15811]
**-0,449101198**	6,02E-08	4,20E-07	*CDH11*	cadherin 11 [Source:HGNC Symbol;Acc:HGNC:1750]
**-0,448457917**	4,65E-05	0,000225021	*TATDN2*	TatD DNase domain containing 2 [Source:HGNC Symbol;Acc:HGNC:28988]
**-0,448265786**	4,19E-06	2,34E-05	*FAM57A*	family with sequence similarity 57 member A [Source:HGNC Symbol;Acc:HGNC:29646]
**-0,447403353**	0,001393771	0,005117788	*GABPB1*	GA binding protein transcription factor subunit beta 1 [Source:HGNC Symbol;Acc:HGNC:4074]
**-0,447200959**	0,009318754	0,027924606	*SLC35G2*	solute carrier family 35 member G2 [Source:HGNC Symbol;Acc:HGNC:28480]
**-0,446782881**	5,55E-09	4,28E-08	*SLC35F6*	solute carrier family 35 member F6 [Source:HGNC Symbol;Acc:HGNC:26055]
**-0,446541879**	2,49E-17	3,51E-16	*ESYT1*	extended synaptotagmin 1 [Source:HGNC Symbol;Acc:HGNC:29534]
**-0,445460213**	1,46E-09	1,18E-08	*PGM3*	phosphoglucomutase 3 [Source:HGNC Symbol;Acc:HGNC:8907]
**-0,445370277**	0,00272537	0,009374074	*CCDC167*	coiled-coil domain containing 167 [Source:HGNC Symbol;Acc:HGNC:21239]
**-0,445365306**	9,28E-08	6,36E-07	*TBC1D2B*	TBC1 domain family member 2B [Source:HGNC Symbol;Acc:HGNC:29183]
**-0,445104677**	4,16E-13	4,57E-12	*PLXNA3*	plexin A3 [Source:HGNC Symbol;Acc:HGNC:9101]
**-0,444941443**	2,35E-06	1,36E-05	*PCDH9*	protocadherin 9 [Source:HGNC Symbol;Acc:HGNC:8661]
**-0,444710285**	0,001423499	0,005212156	*CYP2U1*	cytochrome P450 family 2 subfamily U member 1 [Source:HGNC Symbol;Acc:HGNC:20582]

**-0,444628981**	0,003404277	0,011441531	*MIS18A*	MIS18 kinetochore protein A [Source:HGNC Symbol;Acc:HGNC:1286]
**-0,443760295**	0,000147143	0,000651929	*GTPBP3*	GTP binding protein 3, mitochondrial [Source:HGNC Symbol;Acc:HGNC:14880]
**-0,443739314**	1,00E-07	6,84E-07	*DRAM1*	DNA damage regulated autophagy modulator 1 [Source:HGNC Symbol;Acc:HGNC:25645]
**-0,443646375**	7,67E-11	6,90E-10	*MFSD14B*	major facilitator superfamily domain containing 14B [Source:HGNC Symbol;Acc:HGNC:23376]
**-0,443630482**	0,000152868	0,000675138	*ZNF526*	zinc finger protein 526 [Source:HGNC Symbol;Acc:HGNC:29415]
**-0,443587088**	2,48E-12	2,57E-11	*PLP2*	proteolipid protein 2 [Source:HGNC Symbol;Acc:HGNC:9087]
**-0,443455804**	4,82E-06	2,68E-05	*MAP1LC3A*	microtubule associated protein 1 light chain 3 alpha [Source:HGNC Symbol;Acc:HGNC:6838]
**-0,44345252**	2,96E-09	2,34E-08	*TENM3*	teneurin transmembrane protein 3 [Source:HGNC Symbol;Acc:HGNC:29944]
**-0,44334829**	5,34E-06	2,95E-05	*BAK1*	BCL2 antagonist/killer 1 [Source:HGNC Symbol;Acc:HGNC:949]
**-0,443271645**	2,26E-07	1,48E-06	*TAF13*	TATA-box binding protein associated factor 13 [Source:HGNC Symbol;Acc:HGNC:11546]
**-0,442920731**	0,001513369	0,005512095	*MIPEP*	mitochondrial intermediate peptidase [Source:HGNC Symbol;Acc:HGNC:7104]
**-0,442307653**	0,009654988	0,028790184	*IFITM2*	interferon induced transmembrane protein 2 [Source:HGNC Symbol;Acc:HGNC:5413]
**-0,442299265**	7,52E-17	1,03E-15	*IL6ST*	interleukin 6 signal transducer [Source:HGNC Symbol;Acc:HGNC:6021]
**-0,442121383**	1,33E-11	1,28E-10	*MBNL1*	muscleblind like splicing regulator 1 [Source:HGNC Symbol;Acc:HGNC:6923]
**-0,442001328**	0,001430773	0,005237801	*CDK5R1*	cyclin dependent kinase 5 regulatory subunit 1 [Source:HGNC Symbol;Acc:HGNC:1775]
**-0,441994889**	1,90E-08	1,39E-07	*ANKRD40*	ankyrin repeat domain 40 [Source:HGNC Symbol;Acc:HGNC:28233]
**-0,441926574**	3,86E-06	2,17E-05	*HCCS*	holocytochrome c synthase [Source:HGNC Symbol;Acc:HGNC:4837]
**-0,441921964**	7,38E-09	5,63E-08	*OTUD4*	OTU deubiquitinase 4 [Source:HGNC Symbol;Acc:HGNC:24949]
**-0,441827547**	0,001360989	0,005004215	*SLC38A3*	solute carrier family 38 member 3 [Source:HGNC Symbol;Acc:HGNC:18044]
**-0,441650193**	2,08E-06	1,22E-05	*SNAPC4*	small nuclear RNA activating complex polypeptide 4 [Source:HGNC Symbol;Acc:HGNC:11137]
**-0,440941959**	0,001679631	0,00604169	*MRE11*	MRE11 homolog, double strand break repair nuclease [Source:HGNC Symbol;Acc:HGNC:7230]
**-0,440639097**	0,001359999	0,005002301	*CDK20*	cyclin dependent kinase 20 [Source:HGNC Symbol;Acc:HGNC:21420]
**-0,440173257**	1,08E-15	1,37E-14	*IPO9*	importin 9 [Source:HGNC Symbol;Acc:HGNC:19425]
**-0,440052457**	0,00513004	0,016512451	*SCML1*	Scm polycomb group protein like 1 [Source:HGNC Symbol;Acc:HGNC:10580]
**-0,439549291**	3,97E-08	2,82E-07	*POLE3*	DNA polymerase epsilon 3, accessory subunit [Source:HGNC Symbol;Acc:HGNC:13546]
**-0,439211178**	1,71E-18	2,57E-17	*RIC8A*	RIC8 guanine nucleotide exchange factor A [Source:HGNC Symbol;Acc:HGNC:29550]
**-0,438883091**	0,000138581	0,000616955	*TIMM21*	translocase of inner mitochondrial membrane 21 [Source:HGNC Symbol;Acc:HGNC:25010]
**-0,4388083**	1,29E-06	7,78E-06	*CLEC16A*	C-type lectin domain containing 16A [Source:HGNC Symbol;Acc:HGNC:29013]
**-0,438765707**	0,003859623	0,012803392	*BIN3*	bridging integrator 3 [Source:HGNC Symbol;Acc:HGNC:1054]
**-0,438660657**	0,000790405	0,00306343	*GNAZ*	G protein subunit alpha z [Source:HGNC Symbol;Acc:HGNC:4395]
**-0,438519252**	9,30E-05	0,000428954	*FAM19A5*	family with sequence similarity 19 member A5, C-C motif chemokine like [Source:HGNC Symbol;Acc:HGNC:21592]

**-0,438364515**	8,87E-15	1,07E-13	*RAP1B*	RAP1B, member of RAS oncogene family [Source:HGNC Symbol;Acc:HGNC:9857]
**-0,438085425**	0,000703009	0,002751633	*NOM1*	nucleolar protein with MIF4G domain 1 [Source:HGNC Symbol;Acc:HGNC:13244]
**-0,437371406**	2,55E-05	0,000128645	*SLC5A6*	solute carrier family 5 member 6 [Source:HGNC Symbol;Acc:HGNC:11041]
**-0,437366743**	0,000131755	0,000590494	*CRY1*	cryptochrome circadian regulator 1 [Source:HGNC Symbol;Acc:HGNC:2384]
**-0,437156384**	4,93E-12	4,93E-11	*ZDHHC7*	zinc finger DHHC-type containing 7 [Source:HGNC Symbol;Acc:HGNC:18459]
**-0,437113416**	3,17E-08	2,28E-07	*NEK7*	NIMA related kinase 7 [Source:HGNC Symbol;Acc:HGNC:13386]
**-0,437003393**	0,018094879	0,049632397	*SH3D21*	SH3 domain containing 21 [Source:HGNC Symbol;Acc:HGNC:26236]
**-0,436951029**	1,03E-07	6,99E-07	*ARPC5L*	actin related protein 2/3 complex subunit 5 like [Source:HGNC Symbol;Acc:HGNC:23366]
**-0,436468015**	0,000375314	0,001542102	*ANKS3*	ankyrin repeat and sterile alpha motif domain containing 3 [Source:HGNC Symbol;Acc:HGNC:29422]
**-0,436467495**	2,57E-05	0,000129429	*ORC3*	origin recognition complex subunit 3 [Source:HGNC Symbol;Acc:HGNC:8489]
**-0,435978329**	1,92E-13	2,15E-12	*TMEM43*	transmembrane protein 43 [Source:HGNC Symbol;Acc:HGNC:28472]
**-0,435771195**	0,00102532	0,003868824	*GAS7*	growth arrest specific 7 [Source:HGNC Symbol;Acc:HGNC:4169]
**-0,435733742**	0,002278901	0,007982737	*PDF*	peptide deformylase, mitochondrial [Source:HGNC Symbol;Acc:HGNC:30012]
**-0,435591667**	6,54E-20	1,05E-18	*RBMX*	RNA binding motif protein X-linked [Source:HGNC Symbol;Acc:HGNC:9910]
**-0,435418873**	2,26E-08	1,64E-07	*SMC5*	structural maintenance of chromosomes 5 [Source:HGNC Symbol;Acc:HGNC:20465]
**-0,435323108**	0,001143334	0,004275057	*DBR1*	debranching RNA lariats 1 [Source:HGNC Symbol;Acc:HGNC:15594]
**-0,435264002**	1,12E-06	6,76E-06	*SMIM12*	small integral membrane protein 12 [Source:HGNC Symbol;Acc:HGNC:25154]
**-0,434873795**	8,61E-24	1,64E-22	*TTYH3*	tweety family member 3 [Source:HGNC Symbol;Acc:HGNC:22222]
**-0,434610601**	4,19E-05	0,000204707	*ANKRD27*	ankyrin repeat domain 27 [Source:HGNC Symbol;Acc:HGNC:25310]
**-0,434451441**	1,44E-18	2,16E-17	*PTPN11*	protein tyrosine phosphatase, non-receptor type 11 [Source:HGNC Symbol;Acc:HGNC:9644]
**-0,434137212**	0,000148246	0,00065607	*SLC38A5*	solute carrier family 38 member 5 [Source:HGNC Symbol;Acc:HGNC:18070]
**-0,433841059**	0,004510346	0,014735317	*PRKDC*	protein kinase, DNA-activated, catalytic subunit [Source:HGNC Symbol;Acc:HGNC:9413]
**-0,4337919**	0,016271331	0,045368024	*FRMPD3*	FERM and PDZ domain containing 3 [Source:HGNC Symbol;Acc:HGNC:29382]
**-0,433742898**	0,005470705	0,017492914	*TIMM23B*	translocase of inner mitochondrial membrane 23 homolog B [Source:HGNC Symbol;Acc:HGNC:23581]
**-0,433452494**	1,05E-05	5,56E-05	*EIF2AK3*	eukaryotic translation initiation factor 2 alpha kinase 3 [Source:HGNC Symbol;Acc:HGNC:3255]
**-0,43342389**	0,007388224	0,022772535	*SFXN4*	sideroflexin 4 [Source:HGNC Symbol;Acc:HGNC:16088]
**-0,433014802**	0,008195274	0,024966639	*GTF2H2*	general transcription factor IIH subunit 2 [Source:HGNC Symbol;Acc:HGNC:4656]
**-0,432797333**	2,40E-10	2,09E-09	*ATL3*	atlastin GTPase 3 [Source:HGNC Symbol;Acc:HGNC:24526]
**-0,432254077**	5,10E-08	3,59E-07	*MCMBP*	minichromosome maintenance complex binding protein [Source:HGNC Symbol;Acc:HGNC:25782]
**-0,431738161**	1,19E-07	8,01E-07	*GCNT1*	glucosaminyl (N-acetyl) transferase 1, core 2 [Source:HGNC Symbol;Acc:HGNC:4203]
**-0,431589237**	3,18E-17	4,47E-16	*BCAR1*	BCAR1, Cas family scaffold protein [Source:HGNC Symbol;Acc:HGNC:971]

**-0,4315698**	2,81E-14	3,31E-13	*DNAJC10*	DnaJ heat shock protein family (Hsp40) member C10 [Source:HGNC Symbol;Acc:HGNC:24637]
**-0,431389427**	6,19E-07	3,86E-06	*GORASP1*	golgi reassembly stacking protein 1 [Source:HGNC Symbol;Acc:HGNC:16769]
**-0,431126892**	3,97E-10	3,39E-09	*NFE2L2*	nuclear factor, erythroid 2 like 2 [Source:HGNC Symbol;Acc:HGNC:7782]
**-0,430984227**	0,002975608	0,010149577	*C2orf69*	chromosome 2 open reading frame 69 [Source:HGNC Symbol;Acc:HGNC:26799]
**-0,430815711**	0,005008766	0,01617435	*BORCS8*	BLOC-1 related complex subunit 8 [Source:HGNC Symbol;Acc:HGNC:37247]
**-0,430560787**	0,000111298	0,000504991	*HEATR5A*	HEAT repeat containing 5A [Source:HGNC Symbol;Acc:HGNC:20276]
**-0,43038472**	0,000474033	0,001913287	*HAUS5*	HAUS augmin like complex subunit 5 [Source:HGNC Symbol;Acc:HGNC:29130]
**-0,430331318**	0,000105439	0,000480654	*ATR*	ATR serine/threonine kinase [Source:HGNC Symbol;Acc:HGNC:882]
**-0,4299942**	0,00080976	0,003130799	*PPFIA2*	PTPRF interacting protein alpha 2 [Source:HGNC Symbol;Acc:HGNC:9246]
**-0,429959672**	4,14E-05	0,000202577	*MAP3K6*	mitogen-activated protein kinase kinase kinase 6 [Source:HGNC Symbol;Acc:HGNC:6858]
**-0,429338992**	7,69E-09	5,86E-08	*GFPT1*	glutamine--fructose-6-phosphate transaminase 1 [Source:HGNC Symbol;Acc:HGNC:4241]
**-0,429314996**	1,72E-14	2,05E-13	*NUTF2*	nuclear transport factor 2 [Source:HGNC Symbol;Acc:HGNC:13722]
**-0,429294788**	0,001046067	0,003940214	*RIOK1*	RIO kinase 1 [Source:HGNC Symbol;Acc:HGNC:18656]
**-0,429111464**	3,34E-06	1,89E-05	*METTL3*	methyltransferase like 3 [Source:HGNC Symbol;Acc:HGNC:17563]
**-0,428713592**	3,42E-12	3,47E-11	*RBM12*	RNA binding motif protein 12 [Source:HGNC Symbol;Acc:HGNC:9898]
**-0,428232777**	0,000660157	0,002598054	*RPL26*	ribosomal protein L26 [Source:HGNC Symbol;Acc:HGNC:10327]
**-0,42819595**	1,56E-05	8,03E-05	*SOWAHC*	sosondowah ankyrin repeat domain family member C [Source:HGNC Symbol;Acc:HGNC:26149]
**-0,428115094**	0,003255136	0,010987852	*WASF3*	WAS protein family member 3 [Source:HGNC Symbol;Acc:HGNC:12734]
**-0,428017028**	3,69E-08	2,63E-07	*NOP58*	NOP58 ribonucleoprotein [Source:HGNC Symbol;Acc:HGNC:29926]
**-0,427718637**	6,54E-14	7,53E-13	*CLPTM1L*	CLPTM1 like [Source:HGNC Symbol;Acc:HGNC:24308]
**-0,427589391**	0,003529781	0,01182035	*TRIT1*	tRNA isopentenyltransferase 1 [Source:HGNC Symbol;Acc:HGNC:20286]
**-0,42722836**	0,001051505	0,003958389	*ZNF783*	zinc finger family member 783 [Source:HGNC Symbol;Acc:HGNC:27222]
**-0,426973411**	9,73E-09	7,34E-08	*TMEM9B*	TMEM9 domain family member B [Source:HGNC Symbol;Acc:HGNC:1168]
**-0,426574078**	5,36E-05	0,000257351	*GNPNAT1*	glucosamine-phosphate N-acetyltransferase 1 [Source:HGNC Symbol;Acc:HGNC:19980]
**-0,426312599**	3,37E-09	2,65E-08	*CELSR1*	cadherin EGF LAG seven-pass G-type receptor 1 [Source:HGNC Symbol;Acc:HGNC:1850]
**-0,426075168**	4,64E-10	3,93E-09	*GLIS3*	GLIS family zinc finger 3 [Source:HGNC Symbol;Acc:HGNC:28510]
**-0,425796698**	2,71E-11	2,55E-10	*SRSF10*	serine and arginine rich splicing factor 10 [Source:HGNC Symbol;Acc:HGNC:16713]
**-0,425677576**	1,06E-13	1,20E-12	*PRDX3*	peroxiredoxin 3 [Source:HGNC Symbol;Acc:HGNC:9354]
**-0,425593376**	2,05E-08	1,50E-07	*NUS1*	NUS1, dehydrodolichyl diphosphate synthase subunit [Source:HGNC Symbol;Acc:HGNC:21042]
**-0,42414998**	3,23E-07	2,08E-06	*UBTD2*	ubiquitin domain containing 2 [Source:HGNC Symbol;Acc:HGNC:24463]
**-0,424116772**	0,001183386	0,004402272	*C19orf47*	chromosome 19 open reading frame 47 [Source:HGNC Symbol;Acc:HGNC:26723]
**-0,424078828**	1,13E-05	5,94E-05	*GEMIN5*	gem nuclear organelle associated protein 5 [Source:HGNC Symbol;Acc:HGNC:20043]

**-0,423734217**	0,012618141	0,0363899	*ZNF473*	zinc finger protein 473 [Source:HGNC Symbol;Acc:HGNC:23239]
**-0,423234941**	6,80E-13	7,36E-12	*SORBS3*	sorbin and SH3 domain containing 3 [Source:HGNC Symbol;Acc:HGNC:30907]
**-0,423208834**	1,84E-05	9,42E-05	*LRRC40*	leucine rich repeat containing 40 [Source:HGNC Symbol;Acc:HGNC:26004]
**-0,423080483**	0,008891341	0,026821911	*MMP19*	matrix metallopeptidase 19 [Source:HGNC Symbol;Acc:HGNC:7165]
**-0,422719958**	1,50E-05	7,78E-05	*TTC7A*	tetratricopeptide repeat domain 7A [Source:HGNC Symbol;Acc:HGNC:19750]
**-0,422130853**	1,25E-07	8,44E-07	*MAPKBP1*	mitogen-activated protein kinase binding protein 1 [Source:HGNC Symbol;Acc:HGNC:29536]
**-0,421809727**	5,94E-15	7,24E-14	*NARS*	asparaginyl-tRNA synthetase [Source:HGNC Symbol;Acc:HGNC:7643]
**-0,421558846**	1,08E-06	6,56E-06	*SNRPF*	small nuclear ribonucleoprotein polypeptide F [Source:HGNC Symbol;Acc:HGNC:11162]
**-0,421396617**	0,000100156	0,000458726	*TWISTNB*	TWIST neighbor [Source:HGNC Symbol;Acc:HGNC:18027]
**-0,421334979**	0,000565608	0,002246457	*EIF2B3*	eukaryotic translation initiation factor 2B subunit gamma [Source:HGNC Symbol;Acc:HGNC:3259]
**-0,42123324**	2,35E-06	1,36E-05	*USP38*	ubiquitin specific peptidase 38 [Source:HGNC Symbol;Acc:HGNC:20067]
**-0,420918099**	0,002851532	0,009768196	*NARS2*	asparaginyl-tRNA synthetase 2, mitochondrial [Source:HGNC Symbol;Acc:HGNC:26274]
**-0,420811462**	0,009454782	0,028288544	*TLCD2*	TLC domain containing 2 [Source:HGNC Symbol;Acc:HGNC:33522]
**-0,420730487**	9,28E-11	8,30E-10	*NOMO2*	NODAL modulator 2 [Source:HGNC Symbol;Acc:HGNC:22652]
**-0,420562877**	1,59E-06	9,47E-06	*C1GALT1*	core 1 synthase, glycoprotein-N-acetylgalactosamine 3-beta-galactosyltransferase 1 [Source:HGNC Symbol;Acc:HGNC:24337]
**-0,420511624**	6,21E-09	4,77E-08	*POGK*	pogo transposable element derived with KRAB domain [Source:HGNC Symbol;Acc:HGNC:18800]
**-0,420375995**	2,03E-05	0,000103315	*SPHK1*	sphingosine kinase 1 [Source:HGNC Symbol;Acc:HGNC:11240]
**-0,420331919**	1,39E-07	9,33E-07	*MAP2K3*	mitogen-activated protein kinase kinase 3 [Source:HGNC Symbol;Acc:HGNC:6843]
**-0,420290179**	0,003355734	0,011297948	*TOMM40L*	translocase of outer mitochondrial membrane 40 like [Source:HGNC Symbol;Acc:HGNC:25756]
**-0,419691225**	0,000657602	0,002589573	*TENT4B*	terminal nucleotidyltransferase 4B [Source:HGNC Symbol;Acc:HGNC:30758]
**-0,419687595**	0,015165913	0,042585692	*SOD3*	superoxide dismutase 3 [Source:HGNC Symbol;Acc:HGNC:11181]
**-0,419643654**	1,26E-06	7,61E-06	*C18orf25*	chromosome 18 open reading frame 25 [Source:HGNC Symbol;Acc:HGNC:28172]
**-0,419539388**	9,23E-08	6,33E-07	*WDR47*	WD repeat domain 47 [Source:HGNC Symbol;Acc:HGNC:29141]
**-0,41945767**	7,66E-07	4,72E-06	*SLC52A2*	solute carrier family 52 member 2 [Source:HGNC Symbol;Acc:HGNC:30224]
**-0,419255979**	2,33E-08	1,69E-07	*TMEM33*	transmembrane protein 33 [Source:HGNC Symbol;Acc:HGNC:25541]
**-0,419003613**	5,43E-06	3,00E-05	*UTP18*	UTP18, small subunit processome component [Source:HGNC Symbol;Acc:HGNC:24274]
**-0,418916648**	6,93E-06	3,78E-05	*TONSL*	tonsoku like, DNA repair protein [Source:HGNC Symbol;Acc:HGNC:7801]
**-0,418913479**	1,00E-07	6,83E-07	*DAXX*	death domain associated protein [Source:HGNC Symbol;Acc:HGNC:2681]
**-0,418440703**	3,71E-10	3,17E-09	*SYNGR2*	synaptogyrin 2 [Source:HGNC Symbol;Acc:HGNC:11499]
**-0,418352017**	1,88E-12	1,96E-11	*PPP1R12A*	protein phosphatase 1 regulatory subunit 12A [Source:HGNC Symbol;Acc:HGNC:7618]
**-0,41824177**	2,17E-13	2,43E-12	*SNN*	stannin [Source:HGNC Symbol;Acc:HGNC:11149]

**-0,418219988**	0,003097171	0,010521822	*ZIK1*	zinc finger protein interacting with K protein 1 [Source:HGNC Symbol;Acc:HGNC:33104]
**-0,418011276**	2,23E-10	1,95E-09	*KPNA3*	karyopherin subunit alpha 3 [Source:HGNC Symbol;Acc:HGNC:6396]
**-0,417891118**	2,43E-11	2,30E-10	*CREB3L1*	cAMP responsive element binding protein 3 like 1 [Source:HGNC Symbol;Acc:HGNC:18856]
**-0,417751562**	1,54E-07	1,02E-06	*MSH6*	mutS homolog 6 [Source:HGNC Symbol;Acc:HGNC:7329]
**-0,41751453**	3,04E-16	3,99E-15	*PTGFRN*	prostaglandin F2 receptor inhibitor [Source:HGNC Symbol;Acc:HGNC:9601]
**-0,417480778**	0,006043367	0,019134864	*NFKBIE*	NFKB inhibitor epsilon [Source:HGNC Symbol;Acc:HGNC:7799]
**-0,4174237**	2,03E-12	2,11E-11	*CEP170B*	centrosomal protein 170B [Source:HGNC Symbol;Acc:HGNC:20362]
**-0,417330303**	1,58E-16	2,12E-15	*KLF6*	Kruppel like factor 6 [Source:HGNC Symbol;Acc:HGNC:2235]
**-0,417218714**	7,44E-05	0,000347841	*CASP2*	caspase 2 [Source:HGNC Symbol;Acc:HGNC:1503]
**-0,416735381**	0,0136965	0,039070157	*WDR89*	WD repeat domain 89 [Source:HGNC Symbol;Acc:HGNC:20489]
**-0,416363241**	0,001981749	0,007029395	*YRDC*	yrdC N6-threonylcarbamoyltransferase domain containing [Source:HGNC Symbol;Acc:HGNC:28905]
**-0,416278258**	0,002040354	0,007224068	*MAN1C1*	mannosidase alpha class 1C member 1 [Source:HGNC Symbol;Acc:HGNC:19080]
**-0,41613402**	9,73E-06	5,18E-05	*UACA*	uveal autoantigen with coiled-coil domains and ankyrin repeats [Source:HGNC Symbol;Acc:HGNC:15947]
**-0,41580237**	0,001867377	0,006655338	*PDLIM3*	PDZ and LIM domain 3 [Source:HGNC Symbol;Acc:HGNC:20767]
**-0,41562783**	5,83E-11	5,30E-10	*LEPROT*	leptin receptor overlapping transcript [Source:HGNC Symbol;Acc:HGNC:29477]
**-0,415547672**	0,000223624	0,000955236	*ZNF319*	zinc finger protein 319 [Source:HGNC Symbol;Acc:HGNC:13644]
**-0,415297157**	2,38E-08	1,73E-07	*SINHCAF*	SIN3-HDAC complex associated factor [Source:HGNC Symbol;Acc:HGNC:30702]
**-0,415273891**	6,68E-10	5,58E-09	*C6orf48*	chromosome 6 open reading frame 48 [Source:HGNC Symbol;Acc:HGNC:19078]
**-0,415229801**	0,006305497	0,019880499	*DHODH*	dihydroorotate dehydrogenase (quinone) [Source:HGNC Symbol;Acc:HGNC:2867]
**-0,415098632**	1,80E-06	1,06E-05	*EIF4EBP1*	eukaryotic translation initiation factor 4E binding protein 1 [Source:HGNC Symbol;Acc:HGNC:3288]
**-0,415057789**	0,00028458	0,001192003	*MAP3K9*	mitogen-activated protein kinase kinase kinase 9 [Source:HGNC Symbol;Acc:HGNC:6861]
**-0,414778824**	3,71E-08	2,65E-07	*TMED5*	transmembrane p24 trafficking protein 5 [Source:HGNC Symbol;Acc:HGNC:24251]
**-0,41471863**	7,97E-05	0,000371441	*RABL2B*	RAB, member of RAS oncogene family like 2B [Source:HGNC Symbol;Acc:HGNC:9800]
**-0,414448961**	2,88E-07	1,86E-06	*TANK*	TRAF family member associated NFKB activator [Source:HGNC Symbol;Acc:HGNC:11562]
**-0,413963292**	3,46E-12	3,51E-11	*PDHB*	pyruvate dehydrogenase E1 beta subunit [Source:HGNC Symbol;Acc:HGNC:8808]
**-0,413903002**	2,52E-07	1,64E-06	*DTYMK*	deoxythymidylate kinase [Source:HGNC Symbol;Acc:HGNC:3061]
**-0,413794517**	3,73E-09	2,92E-08	*CTSC*	cathepsin C [Source:HGNC Symbol;Acc:HGNC:2528]
**-0,413782631**	1,63E-10	1,43E-09	*DMWD*	DM1 locus, WD repeat containing [Source:HGNC Symbol;Acc:HGNC:2936]
**-0,413743394**	1,44E-08	1,07E-07	*CAPN15*	calpain 15 [Source:HGNC Symbol;Acc:HGNC:11182]
**-0,413732844**	0,003249376	0,010973711	*MRPL12*	mitochondrial ribosomal protein L12 [Source:HGNC Symbol;Acc:HGNC:10378]
**-0,413600384**	0,015946754	0,044578322	*GRK4*	G protein-coupled receptor kinase 4 [Source:HGNC Symbol;Acc:HGNC:4543]
**-0,413509296**	1,27E-05	6,68E-05	*UGGT2*	UDP-glucose glycoprotein glucosyltransferase 2 [Source:HGNC Symbol;Acc:HGNC:15664]
**-0,413321167**	1,47E-09	1,19E-08	*ZBTB18*	zinc finger and BTB domain containing 18 [Source:HGNC Symbol;Acc:HGNC:13030]

**-0,41303696**	9,39E-10	7,74E-09	*MAP4K5*	mitogen-activated protein kinase kinase kinase kinase 5 [Source:HGNC Symbol;Acc:HGNC:6867]
**-0,412958719**	0,002760451	0,009472911	*BMPR1B*	bone morphogenetic protein receptor type 1B [Source:HGNC Symbol;Acc:HGNC:1077]
**-0,412726601**	0,001469931	0,005366977	*ITGAE*	integrin subunit alpha E [Source:HGNC Symbol;Acc:HGNC:6147]
**-0,412423181**	8,94E-21	1,49E-19	*EIF4G1*	eukaryotic translation initiation factor 4 gamma 1 [Source:HGNC Symbol;Acc:HGNC:3296]
**-0,412386191**	1,88E-07	1,24E-06	*NR2E1*	nuclear receptor subfamily 2 group E member 1 [Source:HGNC Symbol;Acc:HGNC:7973]
**-0,412231855**	0,000245093	0,001039621	*C12orf4*	chromosome 12 open reading frame 4 [Source:HGNC Symbol;Acc:HGNC:1184]
**-0,412112941**	0,016916546	0,046870716	*FAM216A*	family with sequence similarity 216 member A [Source:HGNC Symbol;Acc:HGNC:30180]
**-0,412041927**	1,17E-10	1,04E-09	*NUP188*	nucleoporin 188 [Source:HGNC Symbol;Acc:HGNC:17859]
**-0,412011361**	0,001556471	0,005645773	*EBF4*	early B cell factor 4 [Source:HGNC Symbol;Acc:HGNC:29278]
**-0,41186418**	0,00220675	0,007758006	*RNF138*	ring finger protein 138 [Source:HGNC Symbol;Acc:HGNC:17765]
**-0,411602594**	0,002257608	0,007916722	*BUD13*	BUD13 homolog [Source:HGNC Symbol;Acc:HGNC:28199]
**-0,411253492**	1,35E-07	9,07E-07	*GOSR2*	golgi SNAP receptor complex member 2 [Source:HGNC Symbol;Acc:HGNC:4431]
**-0,411198482**	2,45E-05	0,000123466	*LRRC42*	leucine rich repeat containing 42 [Source:HGNC Symbol;Acc:HGNC:28792]
**-0,411102029**	6,94E-12	6,82E-11	*EIF2S2*	eukaryotic translation initiation factor 2 subunit beta [Source:HGNC Symbol;Acc:HGNC:3266]
**-0,411010761**	3,83E-05	0,000187836	*NLGN1*	neuroligin 1 [Source:HGNC Symbol;Acc:HGNC:14291]
**-0,410724897**	1,88E-08	1,38E-07	*CLTB*	clathrin light chain B [Source:HGNC Symbol;Acc:HGNC:2091]
**-0,410616817**	6,02E-05	0,000286774	*NFKBIB*	NFKB inhibitor beta [Source:HGNC Symbol;Acc:HGNC:7798]
**-0,410374483**	4,73E-19	7,31E-18	*SLC38A2*	solute carrier family 38 member 2 [Source:HGNC Symbol;Acc:HGNC:13448]
**-0,410239587**	5,26E-14	6,08E-13	*MAPKAPK2*	mitogen-activated protein kinase-activated protein kinase 2 [Source:HGNC Symbol;Acc:HGNC:6887]
**-0,410219837**	0,012829346	0,03688936	*S1PR2*	sphingosine-1-phosphate receptor 2 [Source:HGNC Symbol;Acc:HGNC:3169]
**-0,409865141**	0,000253707	0,001073114	*NFYB*	nuclear transcription factor Y subunit beta [Source:HGNC Symbol;Acc:HGNC:7805]
**-0,409706603**	0,00126762	0,004690073	*CCNQ*	cyclin Q [Source:HGNC Symbol;Acc:HGNC:28434]
**-0,40913503**	0,000494338	0,001985741	*SH3RF3*	SH3 domain containing ring finger 3 [Source:HGNC Symbol;Acc:HGNC:24699]
**-0,409039491**	0,003283851	0,011077076	*TNFSF4*	TNF superfamily member 4 [Source:HGNC Symbol;Acc:HGNC:11934]
**-0,408842157**	0,002429271	0,008466674	*FANCG*	FA complementation group G [Source:HGNC Symbol;Acc:HGNC:3588]
**-0,408789365**	1,57E-08	1,16E-07	*KIF1BP*	KIF1 binding protein [Source:HGNC Symbol;Acc:HGNC:23419]
**-0,408698419**	0,005547168	0,017708231	*FBXW9*	F-box and WD repeat domain containing 9 [Source:HGNC Symbol;Acc:HGNC:28136]
**-0,408594456**	2,64E-15	3,29E-14	*TPD52L2*	tumor protein D52 like 2 [Source:HGNC Symbol;Acc:HGNC:12007]
**-0,408499839**	2,53E-21	4,34E-20	*TPM3*	tropomyosin 3 [Source:HGNC Symbol;Acc:HGNC:12012]
**-0,408340882**	0,016664642	0,04629836	*C8orf58*	chromosome 8 open reading frame 58 [Source:HGNC Symbol;Acc:HGNC:32233]
**-0,408211713**	7,12E-08	4,94E-07	*WDR46*	WD repeat domain 46 [Source:HGNC Symbol;Acc:HGNC:13923]
**-0,407985078**	6,74E-11	6,10E-10	*MFSD12*	major facilitator superfamily domain containing 12 [Source:HGNC Symbol;Acc:HGNC:28299]

**-0,407906817**	2,52E-10	2,18E-09	*ELAC2*	elaC ribonuclease Z 2 [Source:HGNC Symbol;Acc:HGNC:14198]
**-0,40782773**	2,26E-14	2,67E-13	*UBE3C*	ubiquitin protein ligase E3C [Source:HGNC Symbol;Acc:HGNC:16803]
**-0,40755659**	3,06E-06	1,74E-05	*SPINDOC*	spindlin interactor and repressor of chromatin binding [Source:HGNC Symbol;Acc:HGNC:25115]
**-0,406808453**	6,28E-24	1,20E-22	*ANXA2*	annexin A2 [Source:HGNC Symbol;Acc:HGNC:537]
**-0,40675916**	3,65E-08	2,61E-07	*GOPC*	golgi associated PDZ and coiled-coil motif containing [Source:HGNC Symbol;Acc:HGNC:17643]
**-0,406470702**	6,37E-10	5,33E-09	*U2SURP*	U2 snRNP associated SURP domain containing [Source:HGNC Symbol;Acc:HGNC:30855]
**-0,406356302**	3,76E-19	5,85E-18	*ARF4*	ADP ribosylation factor 4 [Source:HGNC Symbol;Acc:HGNC:655]
**-0,406182291**	0,000240341	0,001020806	*ATG101*	autophagy related 101 [Source:HGNC Symbol;Acc:HGNC:25679]
**-0,406085666**	0,005397566	0,017293243	*WDR90*	WD repeat domain 90 [Source:HGNC Symbol;Acc:HGNC:26960]
**-0,405979167**	2,42E-10	2,10E-09	*AGAP3*	ArfGAP with GTPase domain, ankyrin repeat and PH domain 3 [Source:HGNC Symbol;Acc:HGNC:16923]
**-0,405575665**	4,16E-10	3,54E-09	*GNAQ*	G protein subunit alpha q [Source:HGNC Symbol;Acc:HGNC:4390]
**-0,405243513**	1,30E-18	1,96E-17	*TMEM123*	transmembrane protein 123 [Source:HGNC Symbol;Acc:HGNC:30138]
**-0,405161176**	1,07E-18	1,62E-17	*SF1*	splicing factor 1 [Source:HGNC Symbol;Acc:HGNC:12950]
**-0,405139268**	1,14E-13	1,30E-12	*STK25*	serine/threonine kinase 25 [Source:HGNC Symbol;Acc:HGNC:11404]
**-0,404987755**	9,73E-07	5,93E-06	*PRKAB2*	protein kinase AMP-activated non-catalytic subunit beta 2 [Source:HGNC Symbol;Acc:HGNC:9379]
**-0,404821245**	3,73E-06	2,10E-05	*GOLT1B*	golgi transport 1B [Source:HGNC Symbol;Acc:HGNC:20175]
**-0,404504297**	9,29E-07	5,68E-06	*WDR3*	WD repeat domain 3 [Source:HGNC Symbol;Acc:HGNC:12755]
**-0,404286951**	0,00868914	0,026293691	*GTDC1*	glycosyltransferase like domain containing 1 [Source:HGNC Symbol;Acc:HGNC:20887]
**-0,403928174**	0,016588842	0,046147107	*HAUS3*	HAUS augmin like complex subunit 3 [Source:HGNC Symbol;Acc:HGNC:28719]
**-0,40392544**	1,45E-11	1,39E-10	*HARS*	histidyl-tRNA synthetase [Source:HGNC Symbol;Acc:HGNC:4816]
**-0,403591219**	2,42E-07	1,58E-06	*CLDN11*	claudin 11 [Source:HGNC Symbol;Acc:HGNC:8514]
**-0,402857688**	1,92E-07	1,27E-06	*SEPT10*	septin 10 [Source:HGNC Symbol;Acc:HGNC:14349]
**-0,402806143**	0,000161734	0,000711221	*RAB30*	RAB30, member RAS oncogene family [Source:HGNC Symbol;Acc:HGNC:9770]
**-0,402728666**	2,56E-12	2,64E-11	*IPO7*	importin 7 [Source:HGNC Symbol;Acc:HGNC:9852]
**-0,402576357**	3,37E-09	2,66E-08	*RIPOR1*	RHO family interacting cell polarization regulator 1 [Source:HGNC Symbol;Acc:HGNC:25836]
**-0,401597698**	4,89E-14	5,68E-13	*MGAT1*	mannosyl (alpha-1,3-)-glycoprotein beta-1,2-N-acetylglucosaminyltransferase [Source:HGNC Symbol;Acc:HGNC:7044]
**-0,401186601**	0,008547751	0,025938647	*RPUSD2*	RNA pseudouridylate synthase domain containing 2 [Source:HGNC Symbol;Acc:HGNC:24180]
**-0,401150345**	1,05E-11	1,02E-10	*P3H1*	prolyl 3-hydroxylase 1 [Source:HGNC Symbol;Acc:HGNC:19316]
**-0,40101636**	6,64E-14	7,63E-13	*ILF2*	interleukin enhancer binding factor 2 [Source:HGNC Symbol;Acc:HGNC:6037]
**-0,400985316**	6,51E-06	3,56E-05	*ARMC6*	armadillo repeat containing 6 [Source:HGNC Symbol;Acc:HGNC:25049]
**-0,400864515**	1,49E-06	8,86E-06	*MICA*	MHC class I polypeptide-related sequence A [Source:HGNC Symbol;Acc:HGNC:7090]
**-0,400862803**	3,48E-08	2,49E-07	*TSR1*	TSR1, ribosome maturation factor [Source:HGNC Symbol;Acc:HGNC:25542]

**-0,40062121**	1,08E-08	8,15E-08	*DFFA*	DNA fragmentation factor subunit alpha [Source:HGNC Symbol;Acc:HGNC:2772]
**-0,40034408**	5,05E-14	5,85E-13	*VDAC1*	voltage dependent anion channel 1 [Source:HGNC Symbol;Acc:HGNC:12669]
**-0,400309785**	9,87E-05	0,000452835	*CCDC9*	coiled-coil domain containing 9 [Source:HGNC Symbol;Acc:HGNC:24560]

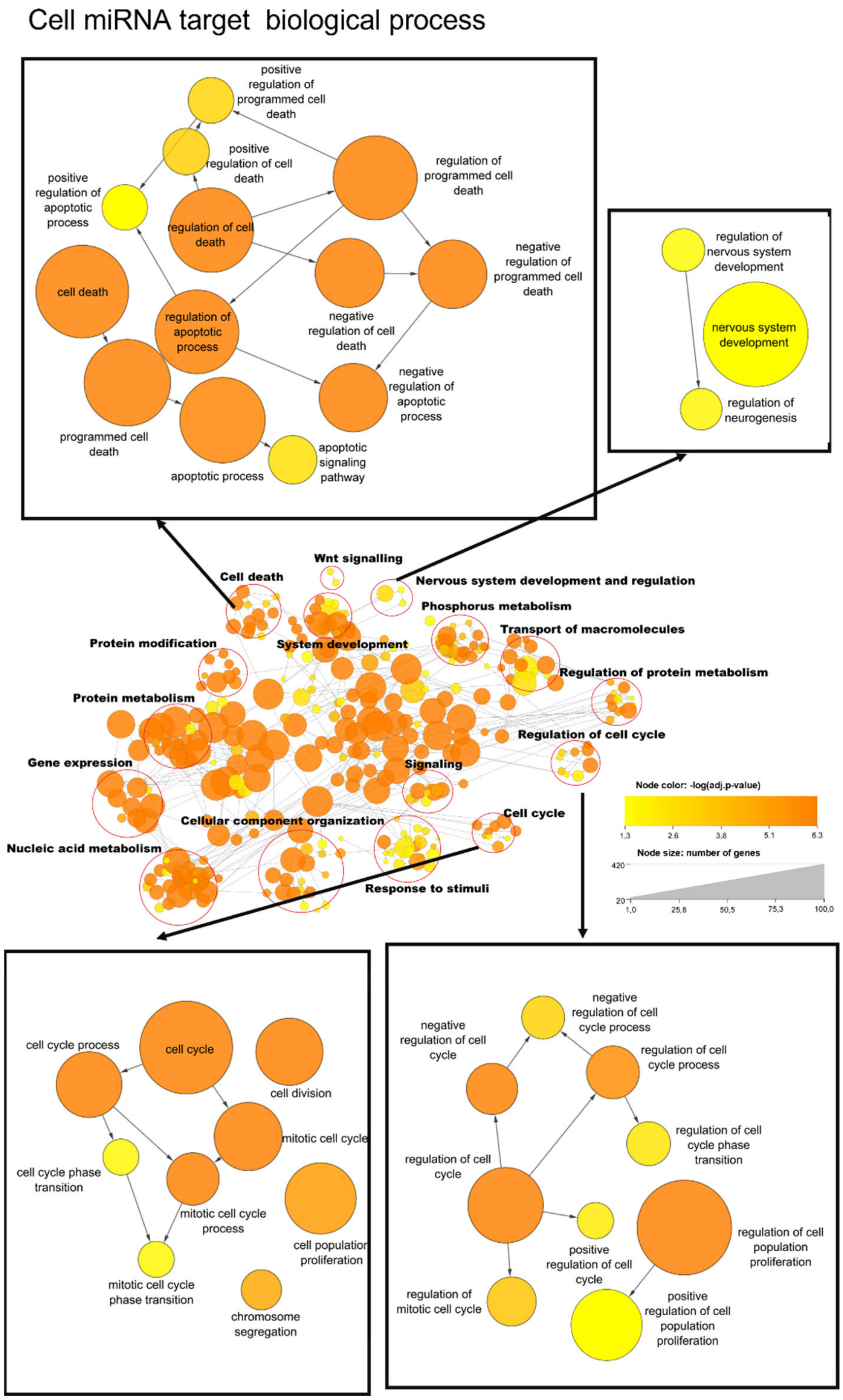

Figure S4. GO biological process of cellular miRNA targets.

Table S2. miRNA Identified in astrocytes: cellular (group1), EV-enriched (group2) and Proteinase K Rnase A/T1 treated EVs (group 3). A: significantly different between at least 2 groups (order from most abundant in cells). B: miRNA not significantly different between groups. Only miRNA represented in all samples with read count >5 are listed.

	p Value			Group1 :Cellular		Group 2: EV		Group 3: EV/ProteinaseK/ RNase A/T1	
**A**	1 vs 2	1 vs 3	2 vs 3	mean	SD	Mean	SD	Mean	SD
**hsa-let-7a-5p**	5,83E-09	1,22E-08	7,30E-01	529093,3	79765,6	139857,4	38543,4	147506,1	16764,8
**hsa-let-7b-5p**	1,21E-02	3,17E-02	6,63E-01	472859,8	126401,0	212171,8	46930,1	241446,2	34655,4
**hsa-miR-125b-5p**	1,45E-06	1,73E-08	5,80E-02	319087,6	14680,1	94938,8	11305,9	64977,1	39435,5
**hsa-let-7f-5p**	3,06E-07	6,50E-08	4,95E-01	264282,2	49473,9	71993,5	28249,3	63320,4	7203,6
**hsa-miR-125a-5p**	1,21E-04	4,86E-06	1,83E-01	156748,4	40381,4	42697,6	6905,0	29103,6	17366,3
**hsa-miR-21-5p**	4,29E-02	6,01E-02	8,70E-01	124441,8	48925,7	68901,0	31859,7	72126,8	19283,1
**hsa-let-7i-5p**	2,63E-02	6,53E-04	1,33E-01	111401,0	13359,8	66078,8	23112,2	46871,5	16231,5
**hsa-miR-9-5p**	2,05E-03	9,43E-06	4,29E-02	75573,1	25632,3	39619,9	8592,5	26560,8	7953,5
**hsa-let-7c-5p**	1,41E-04	8,59E-05	8,43E-01	69692,5	27534,0	24635,8	5157,6	23533,6	3101,8
**hsa-let-7e-5p**	1,42E-04	1,34E-05	3,34E-01	64413,8	10642,7	19035,4	5293,4	14631,6	4079,7
**hsa-miR-16-5p**	2,10E-03	9,51E-03	5,34E-01	59081,0	9385,1	27564,5	11780,5	31688,8	5533,6
**hsa-miR-30a-5p**	2,57E-02	1,33E-02	7,69E-01	54005,7	21507,9	30566,2	9554,5	28467,3	11094,4
**hsa-miR-221-3p**	3,12E-01	4,54E-05	6,31E-04	38967,4	9867,9	32565,1	1863,6	16469,9	1647,0
**hsa-miR-29a-3p**	9,44E-01	1,52E-03	1,80E-03	27723,2	6760,4	27171,2	6834,6	9860,5	6266,4
**hsa-miR-127-3p**	4,78E-01	7,10E-03	3,51E-02	25280,3	6237,8	20770,9	1214,4	11263,9	5171,6
**hsa-miR-5701**	1,26E-03	1,03E-05	4,21E-02	24280,6	11388,8	5983,5	4906,1	2657,1	960,8
**hsa-miR-99b-5p**	3,60E-03	4,76E-04	4,21E-01	20894,3	4077,9	10705,0	2112,1	9030,7	3764,8
**hsa-miR-191-5p**	3,81E-02	1,50E-03	1,74E-01	19803,5	4049,2	11828,0	771,8	8503,3	4741,3
**hsa-miR-31-5p**	1,14E-01	6,65E-03	1,89E-01	19435,5	6663,5	11579,1	695,4	7556,1	5367,7
**hsa-miR-432-5p**	5,03E-02	3,76E-02	8,88E-01	18655,3	4168,3	10851,8	2684,8	10452,5	1212,2
**hsa-miR-181a-5p**	5,70E-02	7,21E-05	1,01E-02	18287,2	1595,9	12258,9	642,7	7009,5	2056,9
**hsa-miR-26a-5p**	4,16E-03	1,67E-03	7,08E-01	18210,9	5627,8	9024,0	2513,7	8292,4	2714,5
**hsa-miR-10a-5p**	5,25E-03	6,48E-05	8,66E-02	14036,5	4479,4	6903,2	1719,8	4567,8	1295,2
**hsa-miR-34a-5p**	6,21E-02	8,50E-05	8,73E-03	14017,5	4038,1	7588,1	616,6	3067,5	2179,7
**hsa-miR-30d-5p**	2,04E-03	3,01E-04	4,38E-01	12473,2	596,6	5033,3	2021,5	4092,6	2103,3
**hsa-miR-151a-3p**	1,32E-02	1,66E-02	9,22E-01	11823,7	2825,5	6597,2	2217,5	6739,2	1057,9
**hsa-miR-409-3p**	1,30E-01	8,38E-03	1,90E-01	11160,4	916,0	5990,6	871,8	3503,2	1113,7
**hsa-miR-222-3p**	2,55E-02	2,61E-03	3,33E-01	10942,3	1406,4	6227,5	2143,2	4934,5	1916,3
**hsa-miR-27b-3p**	3,56E-02	1,14E-01	5,61E-01	10782,0	970,9	5677,7	842,3	6720,4	3611,2
**hsa-miR-103b**	1,20E-01	1,28E-04	6,84E-03	10578,0	2875,0	7452,5	2397,0	3915,6	656,5
**hsa-miR-103a-3p**	1,13E-01	2,21E-04	1,22E-02	10515,6	2827,4	7346,5	2603,3	4064,0	591,1
**hsa-let-7g-5p**	2,32E-03	1,44E-03	8,36E-01	9579,6	1456,0	3019,6	372,8	2818,1	1059,7
**hsa-miR-24-3p**	1,84E-01	4,40E-03	8,62E-02	8299,5	1846,1	5844,5	1572,2	3692,0	1347,6
**hsa-miR-99a-5p**	5,14E-01	1,52E-02	3,39E-03	7256,5	1703,9	9036,5	2991,5	3023,3	774,1
**hsa-miR-1248**	6,95E-06	4,84E-08	6,54E-03	6444,3	4129,7	426,2	119,7	102,3	54,5
**hsa-let-7d-5p**	7,06E-02	9,57E-04	5,81E-02	5644,1	1107,1	2505,8	928,9	1062,5	802,1
**hsa-miR-708-5p**	1,88E-01	9,45E-03	1,47E-01	4905,9	1862,6	3006,7	947,6	1746,0	88,5
**hsa-miR-206**	7,40E-03	6,03E-01	2,32E-02	4433,0	1423,1	15834,4	10163,1	5544,3	1322,5
**hsa-miR-320a-3p**	2,51E-01	1,00E-02	1,16E-01	4043,8	195,3	2967,0	252,2	1925,5	478,5
**hsa-miR-1291**	2,48E-02	8,59E-04	1,38E-01	3965,5	2730,6	1428,3	864,4	746,4	430,7
**hsa-miR-30e-5p**	1,04E-02	3,07E-01	9,34E-02	3952,4	1608,1	1550,6	618,4	2789,4	881,0
**hsa-miR-744-5p**	3,26E-01	6,60E-03	5,66E-02	2936,8	563,1	2089,0	1083,7	1050,6	131,5
**hsa-miR-128-3p**	1,56E-02	1,67E-05	9,76E-03	2873,8	499,4	1607,3	245,9	851,3	282,5
**hsa-miR-221-5p**	3,01E-01	1,39E-02	1,08E-01	2873,2	609,1	1641,5	310,8	674,6	841,9
**hsa-miR-29b-3p**	7,60E-02	1,60E-02	4,29E-01	2836,7	2153,1	1002,4	576,4	638,5	399,5
**hsa-miR-148b-3p**	2,74E-03	1,35E-04	2,18E-01	2736,3	228,3	1089,2	277,6	767,1	458,7
**hsa-miR-423-3p**	5,72E-01	1,35E-02	4,66E-02	2730,1	140,2	2381,1	506,2	1440,1	539,3
**hsa-miR-12136**	2,21E-03	3,89E-03	8,00E-01	2674,0	799,1	13600,6	2087,0	12101,3	14566,1
**hsa-miR-574-5p**	4,22E-02	2,81E-02	8,30E-01	2536,9	493,5	765,6	518,9	678,3	577,3
**hsa-miR-361-5p**	7,71E-02	9,73E-04	6,02E-02	1955,3	319,9	1069,9	461,3	553,6	288,5
**hsa-miR-149-5p**	1,15E-01	3,17E-02	5,15E-01	1932,7	345,2	1147,1	225,2	928,9	132,7
**hsa-miR-92b-5p**	8,66E-01	3,12E-02	4,45E-02	1888,4	491,1	1779,5	273,6	843,6	291,9
**hsa-miR-1246**	8,35E-05	2,49E-03	1,51E-01	1836,4	581,3	12459,7	3800,9	6997,8	2801,1
**hsa-miR-574-3p**	1,76E-01	3,01E-02	3,27E-01	1806,8	837,4	672,1	47,5	328,6	428,5

**hsa-miR-4791**	4,42E-02	1,50E-06	1,10E-04	1744,0	297,1	752,0	411,1	103,0	65,5
**hsa-miR-4497**	2,45E-05	3,45E-06	3,18E-01	1486,1	1445,9	17683,5	4737,7	27691,6	11979,7
**hsa-miR-182-5p**	2,58E-02	9,29E-02	5,46E-01	1440,5	202,0	732,8	241,8	872,4	580,4
**hsa-miR-4284**	1,41E-01	3,28E-05	5,68E-04	1424,3	814,2	602,2	430,2	47,5	31,4
**hsa-miR-106b-3p**	4,54E-01	1,21E-02	6,08E-02	1389,9	346,6	1117,3	135,7	625,6	421,5
**hsa-miR-197-3p**	8,50E-02	9,59E-06	5,59E-04	935,3	183,6	513,7	131,4	125,1	14,7
**hsa-miR-1275**	2,87E-03	1,82E-04	1,85E-01	915,6	262,5	214,2	122,9	118,7	47,8
**hsa-miR-183-5p**	1,31E-01	4,49E-02	1,25E-03	904,3	168,4	636,8	156,3	1456,1	408,2
**hsa-miR-532-5p**	1,55E-01	7,28E-03	1,44E-01	789,5	125,3	465,0	134,5	259,5	262,8
**hsa-miR-15b-5p**	1,27E-02	7,47E-02	4,14E-01	757,4	40,3	214,0	145,8	316,0	377,9
**hsa-miR-185-5p**	1,35E-02	3,25E-03	4,96E-01	707,2	271,7	224,1	113,0	162,3	108,9
**hsa-miR-130a-3p**	3,35E-01	3,83E-02	2,22E-01	650,5	295,4	376,8	29,7	182,4	171,3
**hsa-miR-19b-3p**	7,51E-01	5,50E-04	2,68E-04	628,1	279,3	697,5	194,7	156,6	41,8
**hsa-miR-769-5p**	9,17E-01	5,34E-04	4,35E-04	600,0	163,4	630,9	438,6	82,3	101,9
**hsa-miR-625-3p**	9,57E-03	4,11E-01	1,52E-03	569,8	80,5	182,5	22,0	795,8	728,3
**hsa-miR-151a-5p**	5,18E-01	3,37E-02	1,15E-01	566,3	214,8	396,2	49,0	153,3	202,0
**hsa-miR-296-3p**	2,20E-01	2,50E-02	2,47E-01	425,4	29,4	237,1	173,6	126,2	179,8
**hsa-miR-1180-3p**	5,74E-01	2,47E-04	9,21E-04	409,4	41,8	335,7	48,4	76,9	58,9
**hsa-miR-107**	3,66E-01	3,90E-04	3,13E-03	403,2	183,9	287,1	70,8	78,7	18,5
**hsa-miR-5585-3p**	1,84E-01	1,20E-04	1,70E-03	376,4	82,1	866,4	46,1	8608,8	11229,4
**hsa-miR-3195**	3,08E-04	3,42E-05	2,99E-01	342,1	195,9	2659,3	705,8	4340,8	3783,2
**hsa-miR-4485-3p**	3,29E-02	2,15E-03	1,99E-01	335,0	210,3	1217,2	601,2	2554,6	3692,2
**hsa-miR-760**	3,88E-02	5,06E-02	9,53E-01	267,0	61,8	106,1	25,0	106,4	39,6
**hsa-miR-25-5p**	1,29E-02	1,47E-02	9,50E-01	253,5	88,4	83,2	69,9	80,2	49,4
**hsa-miR-7704**	6,39E-10	4,85E-11	4,96E-02	212,4	53,8	8732,5	2130,8	17263,6	6224,7
**hsa-miR-4448**	9,14E-01	4,65E-03	5,83E-03	187,7	29,8	175,6	144,2	17,4	6,2
**hsa-miR-192-5p**	5,64E-02	2,70E-02	6,43E-01	182,8	39,5	82,4	53,9	69,2	15,3
**hsa-miR-339-3p**	7,61E-01	9,72E-04	5,53E-04	147,5	21,7	175,6	88,0	11,5	5,4
**hsa-miR-320b**	3,78E-01	4,61E-02	2,33E-01	142,8	18,4	207,5	18,7	350,6	157,3
**hsa-miR-4488**	8,34E-08	7,06E-09	1,12E-01	126,9	44,7	3694,3	552,9	7084,3	5297,8
**hsa-miR-10395-5p**	2,58E-02	4,17E-01	5,53E-03	117,7	67,0	451,8	236,0	68,3	73,3
**hsa-miR-320c**	5,93E-02	1,47E-02	4,88E-01	90,7	44,5	217,7	50,6	299,5	56,7
**hsa-miR-4516**	1,78E-06	1,90E-07	1,55E-01	83,0	26,4	3167,3	102,0	6614,2	6458,7
**hsa-miR-219a-2-3p**	1,19E-02	9,17E-05	4,19E-02	79,2	37,6	243,6	15,1	593,8	345,5
**hsa-miR-484**	3,92E-01	1,28E-02	2,63E-03	73,3	7,3	119,5	102,1	11,5	5,4
**hsa-miR-4508**	1,71E-05	1,15E-05	8,10E-01	71,8	60,6	2086,3	377,8	2384,9	429,1
**hsa-miR-122-5p**	6,27E-06	3,03E-07	1,41E-01	68,6	4,8	565,8	197,9	962,9	235,3
**hsa-miR-1304-3p**	1,87E-01	6,78E-02	5,27E-03	66,4	30,4	118,4	32,0	28,5	19,4
**hsa-miR-320d**	2,80E-02	1,71E-03	2,07E-01	62,5	23,8	183,4	62,0	335,8	68,8
**hsa-miR-122b-3p**	7,43E-05	1,34E-06	6,79E-02	62,1	7,5	397,4	123,8	815,3	186,1
**hsa-miR-3182**	3,88E-03	2,75E-02	2,81E-01	58,1	49,5	1449,8	2038,1	541,5	843,3
**hsa-miR-1307-5p**	6,41E-02	1,01E-01	2,76E-03	51,5	24,3	156,6	71,3	17,4	6,2
**hsa-miR-4492**	6,94E-07	1,26E-06	7,02E-01	47,4	42,5	3135,9	1309,0	2561,7	518,3
**hsa-miR-203a-3p**	2,10E-03	1,25E-05	4,31E-03	35,5	26,2	942,3	173,7	17248,8	21195,2
**hsa-miR-8485**	2,13E-02	6,59E-03	5,44E-01	33,5	23,8	181,2	153,4	275,9	271,7
**hsa-miR-219b-3p**	3,46E-01	4,35E-02	2,32E-01	19,8	9,5	38,8	14,2	94,4	76,3
**hsa-miR-196a-5p**	7,10E-03	1,15E-04	8,24E-02	19,5	8,6	50,1	30,6	97,3	46,8
**hsa-miR-219a-5p**	1,10E-01	2,03E-02	3,69E-01	14,6	5,2	43,8	10,4	82,1	58,8
**hsa-miR-203b-5p**	3,68E-04	4,39E-06	5,23E-03	10,4	8,1	879,7	33,4	16387,7	20067,6
**hsa-miR-205-5p**	1,85E-04	2,22E-05	1,22E-01	7,2	7,9	1242,0	1463,1	5374,7	6033,5
**hsa-miR-1269a**	1,11E-01	6,22E-03	1,38E-01	5,2	1,4	19,8	12,3	69,9	54,4
				**miRNA not significanty different**					
		**p Value**		**Cellular**		**EV**		**EV/ProteinaseK/Rnase A/T1**	
(b)	1 vs 2	1 vs 3	2 vs 3	mean	SD	mean	SD	mean	SD
**hsa-miR-100-5p**	6,40E-01	3,27E-01	1,54E-01	42245,5	21385,5	47892,0	4389,6	32398,2	6672,9
**hsa-miR-92a-3p**	2,79E-01	8,79E-01	2,20E-01	14085,7	371,8	11360,0	2628,0	14518,7	8103,1
**hsa-miR-155-5p**	1,85E-01	8,49E-02	6,62E-01	20070,4	5918,6	11339,5	5008,2	9422,3	4966,9
**hsa-miR-143-3p**	9,56E-01	8,88E-01	9,32E-01	8451,9	3773,9	8247,2	3319,3	7945,2	975,7
**hsa-miR-93-5p**	2,41E-01	2,02E-01	9,13E-01	8733,2	2112,3	7057,9	1197,3	6921,5	439,4
**hsa-miR-382-5p**	3,04E-01	5,15E-01	6,99E-01	7187,5	177,8	4891,3	480,1	5639,1	4319,3
**hsa-miR-23b-3p**	1,87E-01	5,58E-01	4,50E-01	7441,2	1788,8	3905,6	387,8	5624,8	3615,9

**hsa-miR-23a-3p**	8,33E-01	6,25E-01	7,80E-01	6942,6	1027,7	6248,3	902,5	5434,7	2752,6
**hsa-miR-379-5p**	6,86E-02	9,78E-02	8,54E-01	8810,9	1922,2	4027,4	2155,5	4346,7	545,9
**hsa-miR-25-3p**	1,40E-01	4,31E-01	4,78E-01	4944,9	1073,1	3731,8	349,6	4262,4	1820,7
**hsa-miR-26b-5p**	6,10E-02	6,78E-02	9,60E-01	7094,1	3075,3	4064,2	310,9	4121,8	2217,8
**hsa-miR-486-5p**	8,36E-01	2,50E-01	3,42E-01	2469,0	402,5	2678,9	704,3	3914,6	735,3
**hsa-miR-342-3p**	1,30E-01	7,38E-02	7,61E-01	6060,3	1468,5	4179,3	738,0	3887,9	1837,1
**hsa-miR-1307-3p**	7,29E-01	3,86E-01	5,99E-01	2915,4	345,9	3183,7	581,8	3647,7	2276,6
**hsa-miR-654-3p**	8,69E-01	9,85E-01	8,54E-01	3303,1	535,0	3076,7	1398,2	3324,2	2119,1
**hsa-miR-10395-3p**	6,06E-02	2,58E-01	4,22E-01	3985,4	2231,5	1817,4	1206,0	2508,8	1113,9
**hsa-miR-181b-5p**	9,63E-01	9,65E-02	1,05E-01	4113,9	67,6	4056,0	851,6	2437,1	1646,5
**hsa-miR-92b-3p**	1,38E-01	6,95E-02	7,13E-01	4031,7	162,6	2585,9	811,5	2328,8	1423,9
**hsa-miR-328-3p**	6,94E-01	7,05E-01	4,44E-01	1972,3	398,8	1662,7	1049,0	2328,0	1760,3
**hsa-miR-370-3p**	5,98E-01	5,25E-01	2,52E-01	2917,8	593,3	3821,4	1313,1	2098,8	2353,9
**hsa-miR-28-3p**	3,77E-01	5,17E-01	8,13E-01	2555,7	313,2	1924,1	336,2	2075,5	271,5
**hsa-miR-146b-5p**	8,15E-02	5,11E-01	2,62E-01	2368,6	601,5	1361,8	233,1	1935,2	452,0
**hsa-miR-3184-3p**	2,30E-01	7,56E-01	1,37E-01	1621,6	243,4	1156,0	164,1	1772,6	699,3
**hsa-miR-423-5p**	2,67E-01	7,76E-01	1,70E-01	1607,5	219,4	1171,1	70,2	1746,1	762,8
**hsa-miR-181a-2-3p**	6,97E-02	3,42E-01	3,52E-01	2605,0	1532,0	920,4	252,1	1538,0	787,6
**hsa-miR-140-3p**	1,20E-01	8,35E-01	1,76E-01	1593,2	10,6	977,3	230,2	1500,3	756,4
**hsa-miR-30c-5p**	2,35E-01	2,40E-01	9,93E-01	2607,3	1092,6	1455,4	358,5	1459,2	1294,7
**hsa-miR-101-3p**	4,70E-02	9,42E-01	4,14E-02	1389,6	787,4	573,5	386,8	1432,4	461,2
**hsa-miR-148a-3p**	1,79E-01	3,65E-01	3,08E-02	1130,3	119,6	801,4	60,9	1426,4	546,7
**hsa-miR-22-3p**	2,78E-01	3,01E-01	9,62E-01	2720,3	1356,7	1370,8	423,1	1422,4	2148,8
**hsa-miR-425-5p**	8,71E-01	2,01E-01	2,62E-01	2000,2	680,4	1889,0	1047,1	1264,6	881,7
**hsa-miR-99b-3p**	3,18E-01	7,15E-01	1,81E-01	1011,0	117,2	632,4	264,3	1198,7	549,4
**hsa-miR-186-5p**	9,61E-01	6,02E-01	5,69E-01	1358,5	303,7	1383,0	349,0	1116,0	1057,3
**hsa-miR-27a-3p**	3,08E-01	2,32E-01	8,48E-01	2344,7	260,9	1214,3	222,0	1075,0	897,4
**hsa-miR-3615**	6,62E-01	4,49E-01	7,44E-01	1281,5	523,1	1113,4	305,6	1003,4	568,6
**hsa-miR-671-5p**	6,72E-01	2,67E-01	4,80E-01	1770,1	645,8	1388,6	708,3	922,9	890,4
**hsa-miR-7-5p**	7,05E-01	1,79E-01	3,26E-01	573,4	68,1	638,0	252,5	858,4	397,8
**hsa-miR-339-5p**	2,17E-01	2,20E-01	9,99E-01	1210,6	288,0	812,5	97,4	807,2	452,4
**hsa-miR-3529-3p**	7,79E-01	2,44E-01	3,71E-01	553,4	65,1	600,7	281,8	797,7	384,6
**hsa-miR-218-5p**	8,75E-01	2,31E-01	2,96E-01	482,8	309,7	507,5	160,7	717,8	477,8
**hsa-miR-199b-3p**	6,02E-01	7,29E-01	8,61E-01	565,9	237,3	774,8	556,9	694,6	504,9
**hsa-miR-135b-5p**	1,18E-01	1,45E-01	9,11E-01	1608,6	751,6	647,9	273,3	689,9	503,8
**hsa-miR-199a-3p**	6,34E-01	7,84E-01	8,40E-01	555,8	241,6	772,0	520,5	669,5	408,4
**hsa-miR-20a-5p**	1,18E-01	8,17E-01	1,85E-01	698,6	312,6	393,4	90,8	650,6	544,8
**hsa-miR-4454**	6,82E-01	2,85E-01	1,49E-01	318,4	183,3	251,6	28,5	604,2	900,1
**hsa-miR-941**	3,39E-01	9,81E-01	3,57E-01	569,2	109,7	383,3	162,5	565,9	478,7
**hsa-miR-126-3p**	4,30E-01	3,99E-01	9,52E-01	844,5	464,1	593,4	58,3	565,1	752,7
**hsa-miR-454-3p**	3,23E-01	4,52E-01	8,14E-01	772,9	327,1	500,7	371,5	552,7	366,6
**hsa-miR-500a-3p**	7,38E-01	3,98E-01	6,03E-01	317,6	84,2	389,8	44,9	543,6	455,7
**hsa-miR-340-5p**	5,04E-01	4,18E-01	1,55E-01	290,6	217,9	178,8	115,2	529,7	811,9
**hsa-miR-93-3p**	9,67E-01	9,42E-02	1,04E-01	176,3	19,8	181,2	141,3	484,8	652,2
**hsa-miR-374a-5p**	1,20E-01	6,69E-01	2,48E-01	611,4	176,5	230,2	78,9	469,8	452,9
**hsa-miR-34c-5p**	7,44E-01	6,96E-01	4,80E-01	315,9	84,0	236,3	77,7	453,6	713,8
**hsa-miR-361-3p**	2,71E-01	1,17E-01	6,03E-01	1019,0	227,8	563,5	135,6	431,1	516,2
**hsa-miR-501-3p**	9,30E-01	9,35E-01	9,96E-01	408,3	263,2	383,4	228,2	385,4	613,4
**hsa-miR-660-5p**	9,73E-01	4,68E-01	4,49E-01	448,4	120,8	455,8	49,4	303,6	405,9
**hsa-miR-3184-5p**	9,94E-01	9,37E-01	9,32E-01	275,8	52,5	274,3	176,3	288,3	416,9
**hsa-miR-576-5p**	7,20E-02	4,80E-01	2,64E-01	333,0	33,8	155,4	71,4	244,5	187,4
**hsa-miR-1296-5p**	9,89E-01	6,87E-01	6,78E-01	184,4	34,2	181,9	166,2	240,6	343,3
**hsa-miR-598-3p**	1,53E-01	2,86E-01	7,23E-01	387,3	138,0	195,7	73,6	237,7	268,4
**hsa-miR-17-5p**	2,12E-01	8,36E-01	1,57E-01	188,5	48,6	91,4	109,1	204,2	223,6
**hsa-miR-345-5p**	7,05E-01	6,19E-01	3,89E-01	264,5	117,6	322,1	53,6	200,3	183,2
**hsa-miR-589-5p**	3,02E-02	6,95E-01	7,15E-02	167,0	126,5	502,6	339,5	198,3	134,2
**hsa-miR-378a-3p**	3,59E-01	6,68E-01	6,47E-01	223,7	16,7	171,1	118,9	197,7	38,5
**hsa-miR-125b-2-3p**	5,68E-01	1,21E-01	3,02E-01	555,0	47,3	382,4	139,7	191,8	312,7
**hsa-miR-363-3p**	3,99E-01	3,72E-01	9,68E-02	96,6	49,6	55,6	26,1	173,7	22,4
**hsa-miR-619-5p**	4,34E-02	7,65E-01	8,43E-02	146,9	11,3	399,0	404,9	161,7	199,8
**hsa-miR-629-5p**	8,70E-01	4,39E-01	5,38E-01	188,4	121,2	176,5	140,1	138,8	14,0

**hsa-miR-20b-5p**	7,37E-01	6,60E-02	1,24E-01	33,2	17,5	41,1	14,4	117,0	66,8
**hsa-miR-10b-5p**	3,40E-02	1,27E-01	5,92E-01	58,0	27,3	123,4	42,7	102,9	44,7
**hsa-miR-324-3p**	9,37E-02	5,83E-02	7,80E-01	277,9	82,2	122,7	13,1	100,9	136,0
**hsa-miR-130b-3p**	1,82E-01	4,18E-01	5,95E-01	175,8	18,4	73,7	70,9	100,8	100,4
**hsa-miR-29c-3p**	3,14E-01	9,77E-02	4,66E-01	262,5	212,9	152,0	124,4	100,0	40,2
**hsa-miR-1306-5p**	7,52E-01	3,88E-01	5,76E-01	148,7	32,3	126,7	88,5	92,0	52,6
**hsa-miR-1247-5p**	4,88E-01	1,12E-01	3,38E-01	13,6	11,4	23,8	13,7	53,3	25,8
**hsa-miR-425-3p**	7,28E-01	8,69E-01	6,18E-01	62,7	10,3	81,5	22,3	52,6	46,6
**hsa-miR-301a-3p**	6,78E-02	3,74E-01	3,50E-01	85,3	66,3	19,8	22,4	45,1	47,5
**hsa-miR-19a-3p**	5,16E-01	5,34E-01	2,33E-01	60,8	39,0	88,9	14,5	44,1	33,3
**hsa-miR-18a-3p**	8,81E-01	2,21E-01	2,79E-01	76,9	18,2	72,2	87,4	40,0	24,6
**hsa-miR-216a-5p**	8,04E-01	3,16E-01	4,39E-01	85,7	63,6	69,5	70,7	35,5	41,0

Table S3. number of targets identified for miRNA target analysis for top 15 cellular miRNAs.

cellular miRNA	Database	Number of hits
**hsa-let-7a-5p**	Tarbase v8	4923
**hsa-let-7b-5p**	Tarbase v8	7981
**hsa-let-7f-5p**	Tarbase v8	4005
**hsa-let-7i-5p**	Tarbase v8	3912
**hsa-mir-9-5p**	Tarbase v8	2091
**hsa-mir-125a-5p**	Tarbase v8	1411
**hsa-mir-125b-5p**	Tarbase v8	1517
**hsa-let-7e-5p**	Tarbase v8	3432
**hsa-let-7c-5p**	Tarbase v8	3390
**hsa-mir-16-5p**	Tarbase v8	11381
**hsa-mir-21-5p**	Tarbase v8	2318
**hsa-mir-29a-3p**	Tarbase v8	2751
**hsa-mir-30a-5p**	Tarbase v8	3431
**hsa-mir-100-5p**	Tarbase v8	1198
**hsa-mir-221-3p**	Tarbase v8	1297
**Total number hits without duplicates**		10280

Table S4. number of targets identified for miRNA target analysis for top 15 preferentially secreted miRNAs.

secreted miRNA	Database	Number of hits
**hsa-mir-203a-3p**	Tarbase v8	2075
**hsa-mir-203b-5p**	Targetscan and mirDB	250
**hsa-mir-205-5p**	Tarbase v8	1140
**hsa-mir-4497**	Targetscan and miRDB	67
**hsa-mir-4516**	Tarbase v8	23
**hsa-mir-5585-3p**	Tarbase v8	32
**hsa-mir-183-5p**	Tarbase v8	1078
**hsa-mir-7704**	Targetscan and miRDB	58
**hsa-mir-12136**	miRDB	2631
**hsa-mir-1246**	Tarbase	377
**hsa-mir-3195**	Targetscan	423
**hsa-mir-4485-3p**	Tarbase v8	30
**hsa-mir-4488**	Tarbase v8	10
**hsa-mir-4492**	Targetscan	5621
**hsa-mir-4508**	Tarbase v8	6
**Total number of hits without duplicates**		9616

Table S5. SNORD significantly different between at least 2 groups : cells (Group1), EV (Group2), EV Proteinase K/ Rnase A/T1 (group3).

GeneName	1VS2	1VS3	2VS3	MeanGroup1	SDGroup1	MeanGroup2	SDGroup2	MeanGroup3	SDGroup3
**SNORD113-6**	1,19E-10	2,24E-18	2,03E-06	75803,46014	2762,667401	2702,299917	1043,029667	178,2800073	30,04840296
**SNORD12B**	7,46E-18	5,64E-23	0,000728	61179,44814	2229,693297	743,4438775	286,9533522	132,4908578	22,33081962
**SNORD69**	5,81E-08	2,18E-14	0,000152	45967,94573	1675,308025	4150,169759	1601,876296	529,9976832	89,32905148
**SNORD113-8**	4,04E-07	8,29E-17	9,19E-09	40174,50933	1464,165449	2616,812223	1010,033255	54,84998083	9,244751282
**SNORD100**	1,44E-08	5,86E-14	0,000983	40002,3507	1457,891104	2774,185409	1070,775922	429,8544184	72,45029307
**SNORD114-22**	1,03E-07	3,68E-14	2,74E-05	37838,53828	1379,030667	1851,195946	714,5218336	120,125224	20,24664005
**SNORD114-12**	1,79E-05	6,89E-13	5,01E-06	37775,13591	1376,719959	4410,134886	1702,217246	240,8845466	40,60015496
**SNORD113-7**	7,17E-10	9,34E-17	3,9E-06	35093,40369	1278,983864	743,5640885	286,9997511	33,60864406	5,664606451
**SNORD59A**	1,43E-10	4,58E-16	0,000428	29734,8884	1083,692046	1017,823342	392,8579262	131,424927	22,15116111
**SNORD113-9**	1,73E-07	3,49E-12	0,001415	28282,14817	1030,746732	1385,573371	534,8015305	165,8422748	27,95207144
**SNORD114-3**	6,05E-05	4,8E-11	7,75E-05	26739,75238	974,5339072	3326,130414	1283,814826	243,5801637	41,05449076
**SNORD3A**	0,00041	3,58E-09	0,00111	23992,59065	874,413225	5036,611842	1944,023881	696,8800242	117,456422
**SNORD114-20**	4,56E-09	8,88E-16	3,89E-06	22824,08548	831,8268952	452,7123058	174,7372165	15,94813858	2,68799683
**SNORD12C**	2,18E-10	4,31E-17	2,29E-05	22315,22992	813,2815852	768,3471407	296,5654764	64,78028298	10,91846515
**SNORD41**	5,74E-07	4,56E-11	0,011107	21024,61637	766,2449991	2197,353393	848,1311653	486,0460016	81,92116622
**SNORD114-25**	6,09E-07	1,17E-13	7,42E-06	20830,80048	759,1813528	959,2004125	370,2307359	38,2136675	6,440765271
**SNORA46**	2,93E-09	1,07E-13	0,003382	18369,57099	669,4815095	725,059455	279,8573604	117,3537292	19,77951536
**SNORD104**	1,7E-06	9,05E-11	0,004766	17931,84562	653,5285492	1478,246247	570,5712684	239,0328523	40,28805906
**SNORD6**	5,08E-05	5,68E-09	0,008043	16568,08931	603,8262651	2203,937658	850,6725501	386,5958961	65,15923711
**SNORD127**	3,67E-07	4,65E-12	0,001776	16175,34434	589,5126216	1266,376604	488,7941415	187,2999824	31,56868473
**SNORD114-21**	1,62E-05	4,09E-11	0,000102	15485,14604	564,3582506	1063,121835	410,3421707	58,54606467	9,867711862
**SNORD101**	4,05E-13	2,81E-18	0,000573	15430,80473	562,3777741	270,5614693	104,4309099	36,94638774	6,227170188
**SNORD114-9**	1,65E-05	1,49E-10	0,000313	15018,65166	547,3567996	942,0563454	363,6134946	60,27530235	10,15916816
**SNORD114-1**	0,003929	4,99E-08	0,000476	13473,90149	491,0581698	2836,895033	1094,980488	218,3441741	36,8010627
**SNORD19C**	2,11E-08	7,2E-17	7,24E-07	13209,47133	481,4209766	920,9994941	355,4860027	54,95761119	9,262891961
**SNORD99**	2,29E-06	1,14E-09	0,026222	12851,58301	468,3776882	1086,689388	419,4387395	241,9129493	40,77348824
**SNORD14E**	4,51E-08	3,74E-10	0,149898	12218,19835	445,2938984	565,1735064	218,1448219	187,7948136	31,65208661
**SNORD111B**	6,21E-08	7,06E-18	1,47E-08	11069,54185	403,4309563	837,0633782	323,0884667	29,48255944	4,969170911
**SNORD82**	4,54E-09	6,8E-13	0,008672	10298,2424	375,3208429	346,724347	133,8281432	61,58676311	10,38020978
**SNORD3B-1**	0,000104	5,21E-08	0,022472	10281,86329	374,7239041	1442,339181	556,7119127	300,4066157	50,63236857
**SNORD71**	9,82E-05	2,54E-08	0,010839	10266,8233	374,1757697	1186,638872	458,0170914	191,8576689	32,3368651
**SNORD3C**	0,004204	8,87E-07	0,008033	9484,630774	345,6686568	2451,827494	946,3526971	406,3586382	68,49017055
**SNORD116-15**	8,39E-06	1,55E-08	0,027088	8745,052681	318,7146327	321,7294523	124,1806512	48,95486707	8,251152749
**SNORD113-5**	2,17E-08	1,85E-13	7,94E-05	8661,064061	315,6536561	87,50545852	33,77522557	2,802893016	0,472416734
**SNORD105B**	6,43E-06	4,7E-10	0,003777	8557,369913	311,8745088	614,7756966	237,2902009	79,69194435	13,43176777
**SNORD116-19**	1,19E-05	3,66E-08	0,040766	8347,31117	304,2188893	324,1360762	125,1095563	55,2012119	9,303949915
**SNORD83A**	2,06E-05	9,78E-10	0,00419	8314,516705	303,0236906	974,0344573	375,9563582	152,2651161	25,66369406
**SNORD46**	1,09E-08	1,16E-13	0,000897	8149,775948	297,0196913	321,3823098	124,0466617	38,55563345	6,498402304
**SNORD114-17**	0,000506	6,01E-09	0,00027	8052,34117	293,4686676	803,7060322	310,2132483	38,68672737	6,520497675
**SNORD1B**	7,13E-06	3,79E-13	3,3E-06	7904,094496	288,0657975	631,6625491	243,8081629	24,5838144	4,143506457
**SNORA74B**	2,95E-06	2,49E-10	0,003661	7650,336402	278,8175493	435,7176313	168,1776375	53,33402323	8,989242515
**SNORD126**	1,24E-05	3,08E-09	0,012514	7552,280152	275,2438759	686,0122517	264,7859795	120,9432382	20,38451316
**SNORD116-8**	0,001853	5,96E-05	0,19399	7362,250366	268,3182146	855,2313926	330,1009296	237,6643815	40,05740863
**SNORD53B**	9,89E-08	5,24E-09	0,374408	7215,991311	262,9877834	368,0095939	142,0437908	161,0465811	27,14377589
**SNORD3B-2**	0,000313	3,98E-07	0,034777	6386,177453	232,7451047	904,3903475	349,0752293	189,7327503	31,97871832
**SNORD116-17**	4,8E-05	2,68E-07	0,053829	6094,809634	222,1261649	244,211509	94,26039179	40,81066937	6,878479851
**SNORD90**	2,24E-06	8,71E-11	0,000877	5880,368737	214,3108373	201,6902419	77,84809693	13,9079535	2,344131556
**SNORD114-13**	9,75E-08	5,95E-11	0,005273	5805,291157	211,5746247	46,70193762	18,0259438	3,520187927	0,593314006
**SNORD66**	0,000981	9,19E-08	0,002926	5690,658686	207,3968287	819,8079039	316,4282246	84,54613623	14,24992296
**SNORA20**	9,59E-07	1,2E-10	0,00728	5118,328469	186,5381762	319,9586243	123,4971497	51,20053008	8,629650529
**SNORA28**	1,11E-05	3,18E-10	0,000703	5008,214493	182,5250574	239,6470346	92,49860282	15,67242556	2,641526472
**SNORD117**	9,53E-07	1,04E-12	3,14E-05	4772,309431	173,9274654	187,3970927	72,33124865	7,63989403	1,287674472
**SNORD91A**	5,37E-08	2,31E-10	0,084471	4741,22336	172,7945293	159,3632138	61,51077412	42,35708333	7,139121919
**SNORD72**	8,29E-09	5,24E-14	0,000138	4544,380032	165,6205475	77,95097028	30,08739854	4,245078572	0,715491509
**SNORA80E**	7,07E-07	2,03E-11	0,00089	4418,086272	161,0177543	157,7553328	60,89016665	12,6329588	2,129236152
**SNORA65**	3,08E-05	9,53E-09	0,009553	4356,507154	158,7734949	325,8451446	125,76922	45,28728511	7,632996052
**SNORD105**	7,57E-06	3,21E-09	0,010018	4267,328733	155,5233753	203,7131861	78,62890991	26,32338934	4,436705057

**SNORD70**	1,29E-08	3,02E-09	0,635315	4094,185899	149,2131612	105,2723557	40,6328659	55,45332274	9,346442218
**SNORD114-28**	0,004178	2,26E-07	0,000524	4057,769136	147,8859473	450,6062115	173,9243094	14,4072877	2,428292398
**SNORD116-16**	0,000434	1,6E-05	0,18321	4029,655415	146,8613389	206,1063715	79,55262801	49,39523825	8,325375601
**SNORD121A**	6,62E-08	5,69E-13	0,000178	3859,863515	140,6732501	80,10202143	30,91765804	4,117729343	0,694027291
**SNORA13**	7,19E-09	1,3E-11	0,033158	3712,026376	135,2853054	61,37849805	23,69078058	11,62216521	1,958870817
**SNORD93**	0,006488	3,52E-09	2,91E-05	3676,972698	134,0077694	977,9058294	377,4506247	54,6316489	9,207952282
**SNORD116-25**	0,000913	6,12E-06	0,051663	3647,349497	132,9281478	250,500592	96,68784262	36,57845769	6,165157006
**SNORD116-24**	6,52E-05	3,55E-06	0,239503	3537,883999	128,9386629	105,6515431	40,77922409	28,94729759	4,878954605
**SNORD116-29**	0,017172	2,05E-06	0,000947	3535,761858	128,8613211	575,9069006	222,2876813	19,52024544	3,29006157
**SNORD113-3**	9,11E-05	2,39E-08	0,002131	3411,428136	124,3299618	108,0382526	41,70044262	4,814573333	0,81147764
**SNORD116-5**	0,003597	4,84E-05	0,080693	3242,767887	118,1831161	301,1425195	116,2345378	49,02512885	8,262995098
**SNORD116-3**	0,005365	5,65E-05	0,065293	3102,31317	113,0642248	329,8463812	127,3136113	49,23626669	8,298581562
**SNORD116-9**	0,006288	3,76E-05	0,040618	3083,507896	112,3788641	348,4638917	134,49957	43,32969878	7,303052477
**SNORD15B**	3,46E-06	4,14E-09	0,027138	3055,968952	111,3752035	144,3379101	55,71132994	25,44915151	4,289355665
**SNORA73B**	0,003601	2,03E-09	2,21E-05	3029,053346	110,3942606	623,2499712	240,5610886	25,82737361	4,353103532
**SNORD114-15**	0,009893	3,32E-05	0,024701	2715,312375	98,95992829	369,5493593	142,6381072	39,03226371	6,578736483
**SNORD114-14**	0,001783	0,000481	0,571846	2375,496036	86,57527569	155,5756446	60,04885384	66,75938849	11,2520357
**SNORD116-2**	0,016465	0,001684	0,312033	2083,542019	75,93497188	296,9888782	114,6313216	87,27399681	14,70969327
**SNORA71D**	0,001769	2,21E-06	0,028341	2080,611568	75,82817118	382,1865329	147,5157845	72,94033005	12,2938094
**SNORA66**	0,001276	1,74E-09	5,78E-05	2061,092979	75,11681356	338,5274544	130,6643189	16,50967998	2,782642453
**SNORA71B**	0,000452	2,41E-08	0,001535	2038,146356	74,28052078	227,4477477	87,78994037	18,43338887	3,106876116
**SNORD62A**	0,004765	5,57E-06	0,021431	2027,796748	73,9033279	412,4957135	159,2144765	67,88149274	11,44116201
**SNORD62B**	0,00433	7,6E-06	0,029701	2002,726347	72,98963374	397,9753163	153,6099154	71,19147167	11,99904611
**SNORD123**	0,001564	6,62E-09	1,91E-05	1922,629389	70,0704892	109,1037803	42,1117134	0,348920007	0,058809112
**SNORD116-7**	0,005533	0,000146	0,12262	1894,422627	69,04248993	173,5011951	66,96773094	31,26037377	5,268814612
**SNORD94**	0,001865	2,59E-07	0,002214	1888,32677	68,82032562	219,9313614	84,88877685	14,75523824	2,48693811
**SNORD91B**	9,27E-06	2,92E-08	0,04649	1861,928816	67,85824858	80,32083199	31,00211421	14,73583568	2,483667883
**SNORA74A**	0,002015	1,96E-06	0,014913	1833,794679	66,83289613	238,6258494	92,10444729	29,60044324	4,989039768
**SNORA3B**	0,002515	3,55E-06	0,017557	1828,612065	66,64401508	223,6371448	86,31912958	26,6657801	4,494413691
**SNORA23**	0,002427	3,65E-06	0,022448	1821,358364	66,37965294	260,7812511	100,655956	37,67592913	6,350131556
**SNORA77**	0,000263	3,62E-05	0,40201	1750,477878	63,79640402	64,63528784	24,94783141	22,319658	3,761891686
**SNORD17**	1,33E-05	6,15E-09	0,005747	1746,696885	63,65860521	52,05539869	20,09226468	4,06811499	0,685664985
**SNORD116-6**	0,020473	0,001861	0,272522	1653,630401	60,26678458	221,8488309	85,62887886	57,09281927	9,622772993
**SNORD116-14**	0,001893	8,06E-05	0,165732	1637,229501	59,66905154	86,08555072	33,22717169	16,87339287	2,843944847
**SNORD116-1**	0,012859	0,000832	0,215372	1626,878532	59,29180906	173,3041377	66,89167102	38,46672445	6,483417037
**SNORA58**	5,62E-05	1,33E-09	0,000279	1608,379439	58,6176071	67,02030299	25,86839598	2,025150204	0,341331203
**SNORD38A**	0,021724	7,91E-05	0,040922	1554,10318	56,63950146	451,2428504	174,1700383	87,3677201	14,72548997
**SNORA53**	0,000548	3,31E-09	5,23E-05	1391,008319	50,6954871	86,34712074	33,32813209	1,082595019	0,182467187
**SNORD114-23**	0,008407	2,12E-05	0,012189	1376,499275	50,16670304	115,1115148	44,43056974	6,049673231	1,019648932
**SNORA26**	0,009363	5,92E-07	0,000683	1211,407277	44,14990275	187,4570906	72,35440654	7,03734206	1,186116678
**SNORD67**	0,000946	3,61E-07	0,004257	1177,270706	42,90579079	90,75502013	35,02948649	5,824308812	0,981664634
**SNORA54**	0,132252	8,43E-08	4,2E-06	1175,768494	42,85104246	476,9010741	184,073561	4,78878954	0,807131882
**SNORA11**	0,005998	2,19E-05	0,018134	1170,859178	42,67212179	88,22343268	34,05234816	5,66478828	0,954778068
**SNORD111**	4,32E-07	1,76E-11	0,001172	1159,093938	42,24333602	37,05136215	14,30102916	2,795427956	0,471158527
**SNORD14A**	1,41E-05	2,79E-06	0,537433	1140,561099	41,56790417	40,54071611	15,64784477	18,12025388	3,054098429
**SNORA14B**	0,024817	0,00095	0,176799	1096,861191	39,97525507	242,1495441	93,46451766	61,47321313	10,36107137
**SNORD109B**	0,067134	0,001125	0,058507	1077,592174	39,27299315	209,5633026	80,88692909	21,25746455	3,582863105
**SNORA55**	0,000158	5,48E-08	0,004126	1006,144304	36,66906582	50,88101321	19,63897713	3,269086451	0,550991827
**SNORD11**	7,98E-07	6,96E-11	0,001295	962,5446065	35,08006892	21,10895159	8,147601461	1,063608834	0,179267139
**SNORD116-23**	0,013803	7,96E-05	0,021821	931,889491	33,96283907	76,55566174	29,54883944	4,140941129	0,697939548
**SNORA7B**	0,007873	1,57E-05	0,01538	927,3746215	33,79829404	114,8456469	44,32795044	9,843710595	1,659119197
**SNORD88A**	0,001809	0,000887	0,784993	914,376089	33,32456076	127,6643587	49,2756976	73,00453315	12,30463058
**SNORD114-11**	0,001198	1,26E-06	0,003592	859,020938	31,30713476	23,87859719	9,216625112	0,34169169	0,057590808
**SNORD53**	0,01574	4,12E-07	0,000301	788,8796588	28,75082631	152,535885	58,87557199	4,883206229	0,823045448
**SNORA37**	0,006607	0,000457	0,22761	781,5242198	28,48275634	62,48592477	24,11822348	14,09531008	2,375709781
**SNORD92**	0,005194	5,32E-05	0,071778	725,4550737	26,43930869	89,64097942	34,59949073	15,00414985	2,528891194
**SNORA2A**	0,004231	0,000122	0,140004	713,464369	26,00230583	67,61090275	26,09635479	13,56153768	2,285744516
**SNORD124**	0,015384	4,58E-07	0,000237	652,739151	23,78916701	103,7336645	40,03896417	1,986897526	0,334883863
**SNORD48**	0,015015	1,92E-05	0,004268	543,8825952	19,82187505	42,37173595	16,35457906	0,705648954	0,118934391
**SNORD63**	0,000168	2,04E-05	0,343916	518,8798698	18,91064733	12,2957477	4,745894247	3,568141817	0,601396448
**SNORD30**	0,038911	0,001303	0,129861	499,010115	18,18649142	95,83840637	36,99156428	17,44180469	2,939748452

**SNORA60**	0,026668	0,002796	0,285081	449,9847027	16,39975361	59,89002398	23,11626159	14,94152275	2,51833564
**SNORD116-13**	0,006119	0,020495	0,523282	436,8319822	15,92040092	14,85415382	5,733384004	20,38795725	3,436311027
**SNORA80B**	0,030936	0,000515	0,07143	391,0892161	14,2532996	62,98723237	24,31171743	8,049209607	1,356663023
**SNORA5C**	0,024179	0,001647	0,226487	358,1857799	13,0541294	55,97389668	21,60472065	13,3262216	2,246082905
**SNORA2B**	0,138227	3,33E-06	9,41E-05	353,4520663	12,88160856	113,0750467	43,64453684	0,702791277	0,118452741
**SNORD20**	0,012526	3,6E-05	0,019124	339,824851	12,38496285	45,46113434	17,54702042	3,731776447	0,628976429
**SNORA79B**	0,108896	0,000399	0,016617	328,6102122	11,97624382	99,86876696	38,54719681	7,549326855	1,272409727
**SNORD115-17**	0,029871	0,002141	0,169378	322,4195202	11,75062321	25,92451853	10,00630676	3,388570751	0,571130442
**SNORD115-19**	0,041218	0,00243	0,13952	317,2543834	11,56237909	30,21507079	11,66236769	3,388677972	0,571148514
**SNORD89**	0,143661	1,51E-07	5,08E-06	289,1185035	10,53696313	108,8535521	42,01513069	0,348144968	0,058678483
**SNORA71A**	0,110468	0,000966	0,04437	263,8201019	9,614959448	98,72438424	38,10548969	14,75900777	2,487573449
**SNORD115-18**	0,033397	0,005785	0,331099	247,6717239	9,026429619	18,29241185	7,060477679	3,77476949	0,63622274
**SNORD115-32**	0,044839	0,00497	0,225013	239,7771085	8,738709287	20,6276045	7,961811833	3,009650955	0,507264982
**SNORA74D**	0,001125	3,5E-05	0,104171	213,407585	7,777668421	6,556883327	2,530815987	0,690016469	0,116299596
**SNORA12**	0,005574	0,002577	0,691932	188,8899542	6,884120035	10,64908785	4,11031895	4,882140811	0,822865876
**SNORD115-15**	0,123211	0,001435	0,02318	148,9448672	5,428315915	22,68550681	8,756117877	0,355378982	0,059897747
**SNORA75**	6,12E-05	1,84E-07	0,023628	145,4960283	5,302622512	4,960693958	1,91472121	0,318486616	0,053679682
**SNORD115-39**	0,060323	0,002057	0,064084	141,8234233	5,168773924	11,36367256	4,386133282	0,355812434	0,059970804
**SNORA30**	0,01273	0,000777	0,223397	132,2602589	4,820243101	18,53722562	7,154970528	4,506343034	0,75952662
**SNORA38B**	0,001144	9,31E-05	0,189825	131,6458456	4,797850724	2,968137751	1,145637355	0,351055642	0,059169065
**SNORA63C**	0,057338	0,000787	0,038327	123,1487807	4,488174039	16,15108708	6,233972356	0,706938914	0,119151809
**SNORA49**	0,01587	0,00767	0,66003	86,14916972	3,139718191	4,651805494	1,795496904	1,859890736	0,313477361
**SNORA84**	0,310498	0,000251	2,39E-05	33,10222798	1,206415195	92,73655902	35,7943179	0,340683235	0,057420836
**SNORA9B**	0,174369	0,001167	0,020018	30,08125733	1,096315509	9,68013305	3,736323229	0,341369681	0,057536534
**SNORD73B**	0,00079	0,008462	0,221879	23,1242673	0,842767063	0	0	0,342628299	0,057748669
**SNORA22**	0,003696	0,027598	0,240913	21,9493498	0,799947035	0	0	0,357239118	0,060211266
